# Topology-Aware Generation and Activity-Based Filtering: A Computational-Experimental Framework for Data-Scarce Quaternary Ammonium Compound Discovery

**DOI:** 10.1021/acs.jcim.6c00390

**Published:** 2026-03-05

**Authors:** Shiva Ghaemi, Amanda Consylman, Bo Pan, Alice Wu, Ashley Petersen, Gabe Chang, Diana McDonough, Mark Forman, Elise L. Bezold, William M. Wuest, Amarda Shehu, Liang Zhao, Kevin P. C. Minbiole

**Affiliations:** † Department of Computer Science, 3298 George Mason University, Fairfax, Virginia 22030, United States; ‡ Department of Chemistry and Biochemistry, 8210 Villanova University, Villanova, Pennsylvania 19085, United States; § Department of Computer Science, 1371 Emory University, Atlanta, Georgia 30322, United States; ∥ Department of Chemistry, St. Joseph’s University, Philadelphia, Pennsylvania 19131 United States; ⊥ Department of Chemistry, Emory University, Atlanta, Georgia 30322, United States

## Abstract

Quaternary ammonium compounds (QACs) are widely used antimicrobial disinfectants whose efficacy is threatened by increased bacterial resistance. Artificial intelligence-guided development of novel QACs is constrained by historically sparse structure–activity data and methods to generate novel chemical entities with bioactivity. This paper presents a comparative experimental study of two computational workflows designed to accelerate QAC discovery under data-limited conditions. Both workflows employ a topology-aware variational autoencoder to generate novel candidates. In Workflow 1, generated QAC structures were directly subjected to expert evaluation within a fixed time constraint through the systematic application of chemistry-domain decision criteria. In Workflow 2, generated candidates were first computationally filtered using predictive models trained to anticipate antimicrobial activity, advancing only molecules projected to be highly active against at least one bacterial strain for expert evaluation. This predictive filtering enabled the assessment of a larger, higher-quality candidate pool within the same time constraint. Comparative assessment of the compound sets resulting from the two workflows revealed substantial improvements in candidate quality: compounds deemed synthesis-worthy increased from 9% to 38%, while invalid outputs decreased from 21% to 0%. Experimental characterization of 29 selected compounds across both workflows yielded 11 novel QACs with experimentally validated minimum inhibitory concentrations of 1–32 μM against four bacterial pathogens. These results demonstrate that topology-aware generation coupled with computational prefiltering enables systematic navigation of data-scarce chemical spaces while respecting practical constraints on expert evaluation time.

## Graphical abstract

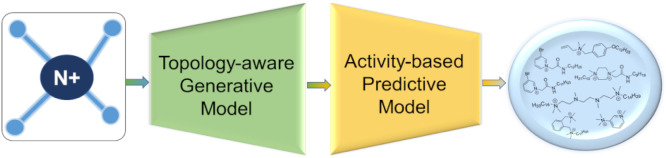

## Introduction

Quaternary ammonium compounds (QACs) constitute a high-use class of antimicrobial disinfectants. Ubiquitous in household cleaning products, it is estimated that over 10 billion pounds of QACs are produced annually.^1^ Surprisingly, commercialized QACs have a relatively uniform structure, which have resulted in the development of bacterial resistance;^1–3^ this has motivated research toward the structural variation of current active ingredients.

The application of AI toward the discovery of novel QACs is currently constrained by sparse experimental data in the literature. To date, well under 10,000 unique QACs have been both developed and fully tested such that structure–activity relationships could be realized, with many of these originating from systematic investigations by our wet laboratories over the past few years.^4–13^ Other data sets are severely limited in the breadth of microbes tested for inhibition and/or toxicity data. The scarcity of consistent analysis and information stands in stark contrast to typical drug discovery data sets containing upward of a million small molecule compounds.^14–16^

QACs are amphiphilic molecules and feature a permanently charged quaternary ammonium core (N^+^) with four covalent bonds to hydrophobic substituents, typically comprising alkyl chains (C_4_–C_18_) and aromatic groups such as benzyl or phenyl moieties. This topology, illustrated in Figure 1, enables membrane disruption through electrostatic interaction with negatively charged phospholipid headgroups and subsequent membrane insertion via hydrophobic tails. This distinctive topology is also poorly represented in existing molecular databases, precluding the direct application of generative deep learning-based models trained over these databases^17–25^ for QAC discovery.

**Figure 1:**
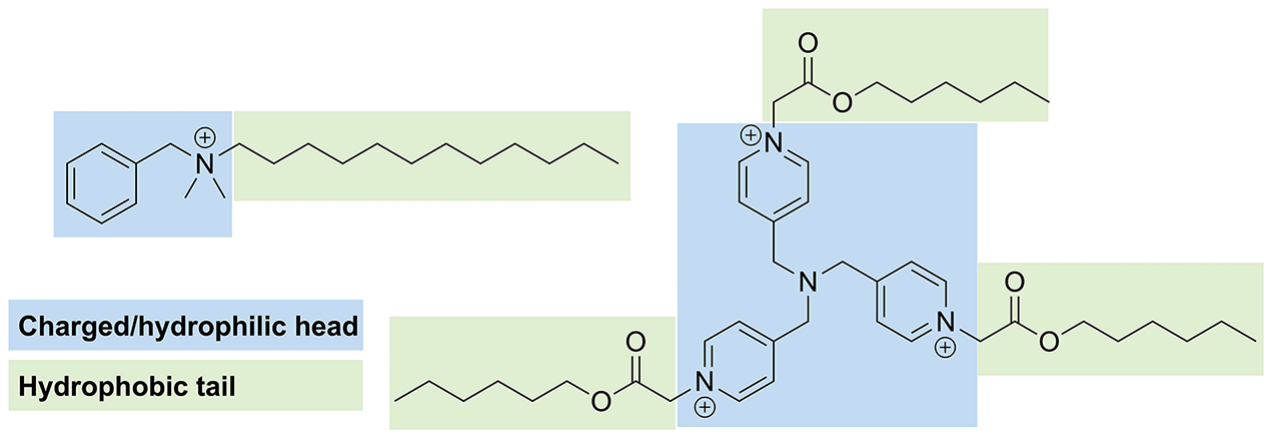
QACs have a unique topology, a central core fragment to which an arbitrary number of tails can attach themselves.

Data scarcity presents challenges that has been recognized and addressed in other domains, such as antimicrobial peptide (AMP) discovery. Pioneers in this field have built predictive machine learning models that leveraged a few thousand known AMPs to train a data set for mapping between molecular structure and antimicrobial activity,^26,27^ and more recent work has entered the discovery space.^20,28–30^ For instance, de la Fuente-Nunez and colleagues have mined the proteomes of all available extinct organisms, guided by AMP structure–activity predictive models, and report 69 peptides with experimentally confirmed activity against bacterial pathogens.^29^ However, AMPs benefit from larger characterized data sets and leverage natural sequence databases, which are unavailable for QACs. Fortunately, generative (deep learning-based) models provide opportunities to address such gaps with *de novo molecular design*, though novel architectures are required to capture the distinctive, hierarchical QAC topology. Moreover, this topology leads to a combinatorially vast design space; generative models that capture QAC topology alone are likely to produce many compounds lacking desirable properties, the most important of which is antimicrobial potency, measurable through minimum inhibitory concentration (MIC) against pathogens of interest. However, the practical challenge extends beyond computational generation to efficient expert evaluation: chemistry domain experts must assess generated candidates for synthetic feasibility, structural novelty, and potential bioactivity, a time-intensive process that becomes prohibitive as the number of generated molecules scales into the thousands.

This paper addresses a fundamental question in molecular discovery under resource constraints: Does predictive filtering of computationally generated molecules improve the efficiency of expert-guided candidate selection? To investigate this question, two distinct computational workflows were designed and evaluated experimentally. Both workflows employed a topology-aware variational autoencoder specifically designed to capture QAC’s distinctive hierarchical structure, a central charged core with variable hydrophobic tails but differed in their approach to candidate prioritization prior to expert evaluation. Both workflows concluded with wet-laboratory synthesis and biological characterization of selected candidates. The resulting experimental data enabled direct comparison of workflow effectiveness across multiple metrics: proportion of synthesis-worthy candidates, chemical validity rates, structural novelty, and ultimately, the number and potency of experimentally validated antimicrobial compounds.

Herein we demonstrate that 1) topology-aware generative models can produce chemically valid QACs at high rates when trained on limited data sets, addressing the challenge of generating molecules conforming to underrepresented topologies; 2) the model provides experimental evidence that computational prefiltering enables more efficient use of limited expert evaluation time, demonstrating a practical workflow design for resource-constrained discovery settings, increasing the proportion of synthesis-worthy candidates from 9% to 38% and eliminating invalid outputs entirely; and 3) 11 compounds were proposed by the model and validated, to represent novel QACs with MICs of 1–32 μM against multiple bacterial pathogens, demonstrating the first example of AI-generated disinfectant compound discovery.

## Methods

### QAC Structure and Topology

As shown in Figure 1, QACs exhibit a distinctive molecular architecture that enhances their affinity for phospholipid membranes. QACs feature a permanently charged quaternary ammonium core (N^+^) with four covalent bonds to organic substituents, typically comprising hydrophobic alkyl chains (C_4_–C_18_) and oftentimes aromatic groups such as benzyl or phenyl rings. The nitrogen atom itself is frequently incorporated into an aromatic ring, as in the pyridinium structures in Figure 1.

### Data Set

The computational workflows utilized two related data sets derived from ongoing experimental characterization efforts across collaborating laboratories (W.M.W. and K.P.C.M.) since 2012.^11,13^

#### Complete Data Set

The complete data set comprised 603 characterized QACs with structural information. This data set was used to train the generative model, providing maximum structural diversity for learning the QAC topology.

#### Curated Data Set

For the predictive model, the complete data set was curated to retain only compounds with valid, numeric MIC measurements. Salt components were not separated prior to featurization; SMILES were used as provided (salts intact). Compounds were excluded due to missing MIC values (17 compounds), non-numeric annotations (e.g., “insol.” for insoluble or “NT” for not tested; 12 compounds), or MIC values reported only with inequality qualifiers (e.g., “>”, “≥”, “≤”; 153 compounds) that prevent direct numeric comparison. This curation yielded 421 compounds with valid MIC measurements against four bacterial strains: *Staphylococcus aureus*, *Enterococcus faecalis*, *Escherichia coli*, and *Pseudomonas aeruginosa*.

For binary classification, compounds were labeled as active (1) if MIC ≤ 8 μM and inactive (0) otherwise. This threshold was applied consistently across all four bacterial strains. The 8 μM cutoff was selected to focus on strong lead compounds with single-digit micromolar activity as a minimum requirement for further development. The curated data set was split (80/20%) into a training portion and a held-out test set, and model evaluation and selection were performed using 5-fold cross-validation on the 80% training portion, with final performance reported on the held-out 20% test set.

### Study Design: Comparative Workflow Evaluation

This study employed a comparative experimental design to evaluate whether predictive filtering of computationally generated molecules improves the efficiency of expert-guided candidate selection. Two workflows were conducted sequentially, both utilizing the same topology-aware generative model architecture but differing in their approach to candidate prioritization prior to expert evaluation.

Workflow 1 generated QAC candidates using the topology-aware variational autoencoder trained on the complete data set of 603 compounds. Generated candidates were directly submitted for expert evaluation under a practical time constraint of approximately 4 h. Domain experts sampled candidates and applied systematic chemistry decision criteria to assess chemical validity, QAC topology conformance, synthetic feasibility, structural novelty, and predicted biological relevance.

Workflow 2 introduced predictive filtering as an intermediate computational step. Generated candidates were evaluated using a random forest classifier trained on the curated data set of 421 compounds. The classifier predicted binary activity (MIC ≤ 8 μM) for each of the four bacterial strains. Generated candidates were ranked by the number of strains against which they were predicted active (range: 0–4), and only those predicted highly active against at least one strain were advanced for expert evaluation. This filtering enabled more comprehensive expert assessment within the same 4 h constraint.

Both workflows concluded with wet-laboratory synthesis and biological characterization of selected candidates, enabling direct comparison of workflow effectiveness. They are graphically summarized in Figure 2.

**Figure 2:**
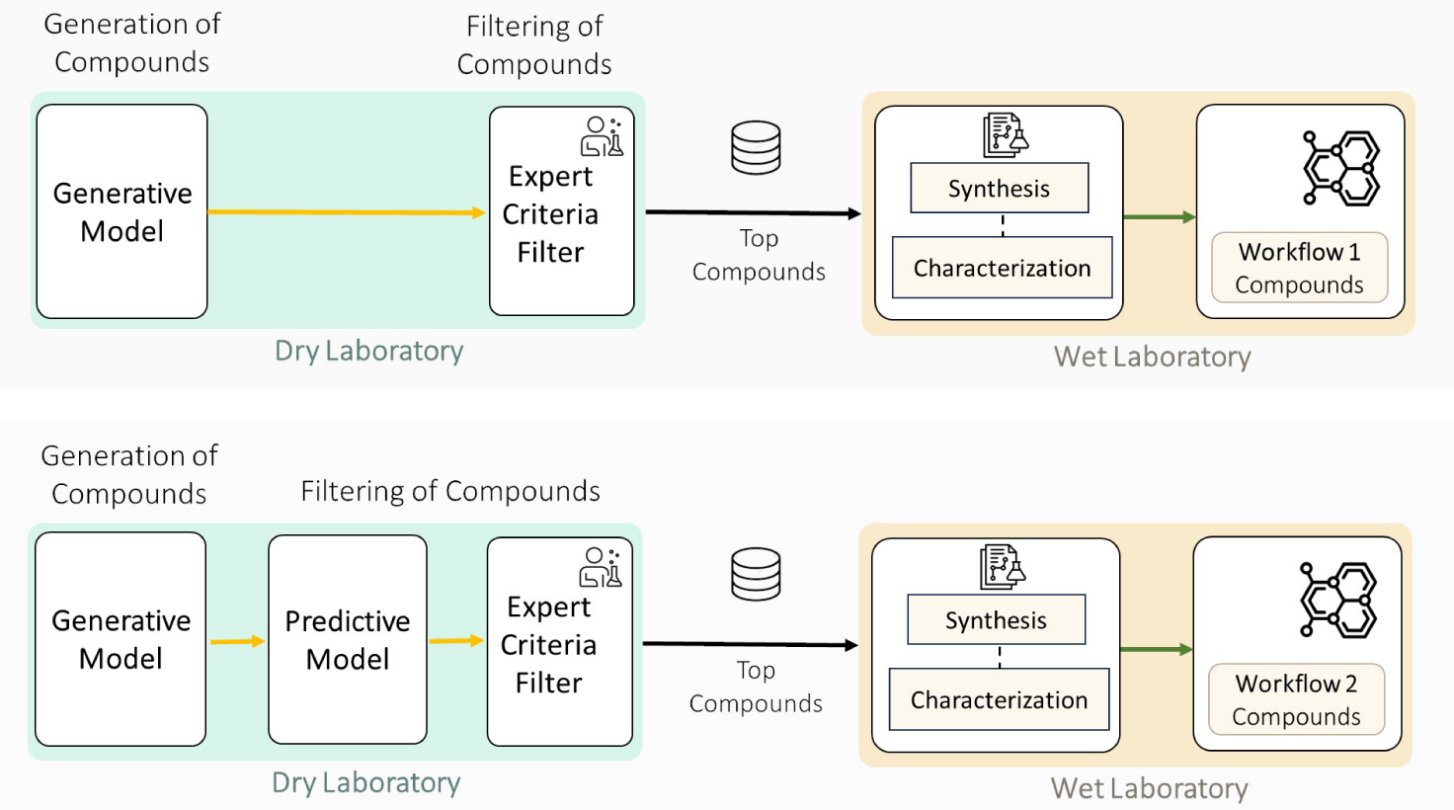
Schematic of the overall workflows.

## Computational Components

### Generator Model

The generator model is shown schematically in Figure 3. The model is based on the hierarchical variational autoencoder (VAE) architecture and has dedicated encoder and decoder components for the core and tail fragments of a QAC. We implement these components with HierVAE.^31^ We pretrain the core and tail encoder–decoder modules separately on the sets of cores and tails extracted from our QAC data set. We also include a GRU-based sequence module with an encoder and a decoder that models the sequence of components.

**Figure 3:**
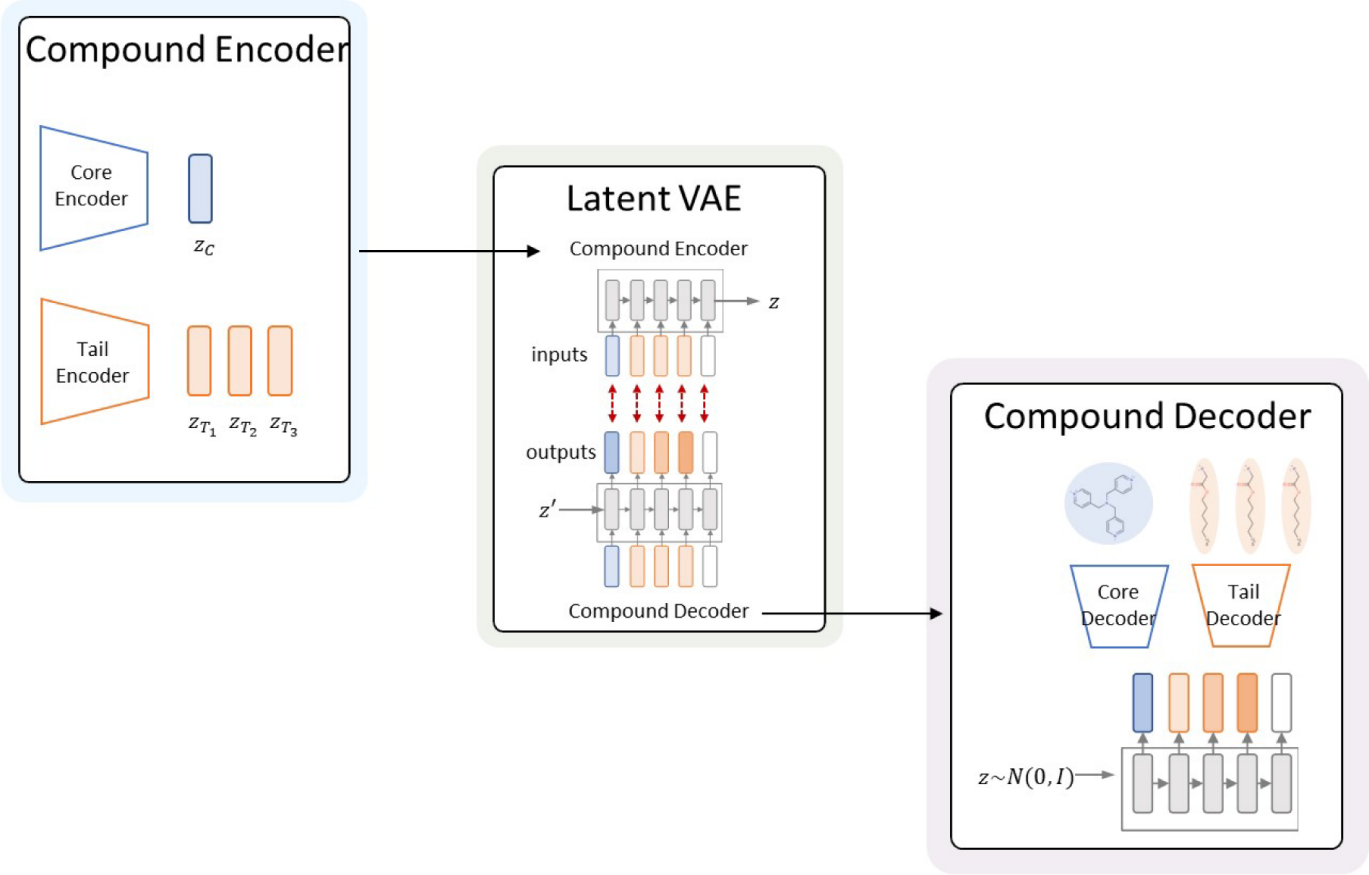
Generator model is a hierarchical VAE, with separate encoder and decoder components for the core and the head fragments of QACs.

#### Encoding Stage

Each QAC molecular graph *G* is decomposed into a core subgraph *G*_core_ and a set of tail subgraphs ${\{G_{tail}^{i}\}}_{i=1}^{k}$ , where *k* denotes the number of identified tails. This decomposition is performed using a rule-based method that detects alkyl or aryl chains (tails) connected to the positively charged nitrogen atom (N^+^). A fragment is cut as a tail only if it is attached to exactly one N^+^ and contains at least six consecutive carbon atoms; this procedure is applied iteratively until there is no remaining N^+^. The remaining portion of the molecule, excluding these chains, is defined as the core.

To capture the topological features of QACs, the core and each tail are encoded separately:

$$h_{core}={\mathrm{Enc}}_{\mathrm{core}}\left(G_{core}\right),h_{tail}^{i}={\mathrm{Enc}}_{tail}\left(G_{tail}^{i}\right),i=1,...,k$$

where *h*_core_ ∈ R^*d*^ and *h* ^*i*^_tail_ ∈ R^*d*^ are the *d*-dimensional latent representations of the core and the *i*th tail, respectively.

These representations are then integrated using a Gated Recurrent Unit (GRU) encoder to form a unified molecular embedding:

$$h={\mathrm{GRU}}_{E}\left(h_{core},\left[h_{tail}^{1},...,h_{tail}^{k}\right],h_{\langle \mathrm{EOS}\rangle }\right)$$

where *h*_⟨EOS⟩_ is a fixed embedding denoting the end-of-sequence token. We adopt an autoregressive GRU at the global level because the tails attached to a given core are not independently distributed and the number of tails varies across QACs. The ⟨EOS⟩ token provides a natural stopping rule for deciding how many tails to generate.

Finally, variational sampling is performed on the aggregated embedding *h*. Two separate multilayer perceptrons (MLPs) are used to compute the mean μ_*h*_ and log-variance log σ_*h*_, from which a latent vector *z* is sampled:

$$Z∼N\left(\mu_{h},\sigma_{h}\right)$$

#### Decoding Stage

The decoding stage reconstructs the molecular graph from the latent representation *z* obtained during the encoding phase.

First, component separation is needed. A decoder GRU is used to expand the latent vector into individual component embeddings:

$$\left[{ĥ}_{0},{ĥ}_{1},...,{ĥ}_{k+1}\right]={\mathrm{GRU}}_{D}\left(z\right)$$

Here, *ĥ*_0_ corresponds to the core embedding, while *ĥ*_*i*_ for *i* = 1, ..., *k* correspond to the *k* molecular tails. *ĥ*_*k*+1_ represents the end-of-sequence token.

##### Core and Tail Reconstruction

The core and tail need to be reconstructed next. So, separate decoders generate the graph structure for the core and each tail:

$${Ĝ}_{core}={\mathrm{Dec}}_{\mathrm{core}}\left({ĥ}_{0}\right),{Ĝ}_{\mathrm{tail}}^{i}={\mathrm{Dec}}_{\mathrm{tail}}\left({ĥ}_{i}\right),i=1,...,k$$

During generation, tail order is determined autoregressively by the GRU: at each step it selects the next tail conditioned on the core and the previously generated tails and stops when the ⟨EOS⟩ token is produced. Finally, the core and tail(s) need to be assembled together in a molecular graph *Ĝ* which is reconstructed by assembling the predicted core and tail subgraphs:

$$Ĝ=\mathrm{Assemble}\left({Ĝ}_{core},{\{{Ĝ}_{tail}^{i}\}}_{i=1}^{k}\right)$$

During decomposition, the N^+^ atom is retained in both core and tail fragments; at generation time, they are rejoined by merging their N^+^ atoms. Assembly is deterministic and order-insensitive: given the same core and the same multiset of tails, this rule yields the same molecule regardless of the order in which tails are generated.

### Training Objective

The model is trained by minimizing a VAE loss, which combines a reconstruction loss and a regularization term on the latent space:

$$L=L_{\mathrm{recon}}+\lambda L_{\mathrm{latent}}$$

Here, *L*_recon_ denotes the reconstruction loss, computed as the sum of graph matching errors for the core and tail components, along with the loss associated with the end-of-sequence token:

$$L_{\mathrm{recon}}=\left|\left|G_{\mathrm{core}}-{Ĝ}_{\mathrm{core}}\right|\right|+\overset{k}{\underset{i=1}{\sum }}\left|\right|G_{\mathrm{tail}}^{i}-{Ĝ}_{\mathrm{tail}}^{i}\left|\left|+\right|\right|{ĥ}_{k+1}-h_{\langle \mathrm{EOS}\rangle }\left|\right|$$

The term *L*_latent_ encourages the learned posterior distribution to align with a prior distribution using the Kullback–Leibler divergence:

$$L_{\mathrm{latent}}=D_{\mathrm{KL}}\left(q(z\left|G_{\mathrm{core}},{\{G_{\mathrm{tail}}^{i}\}}_{i=1}^{k}\right)||p\left(z\right)\right)$$

In this formulation, $q\left(z|G_{\mathrm{core}},{\{G_{\mathrm{tail}}^{i}\}}_{i=1}^{k}\right)$ is the posterior distribution learned by the encoder, and *p*(*z*) is the assumed prior, typically a standard Gaussian.

To mitigate the tail ordering ambiguity, data augmentation via random permutation of tail orderings is applied during training.^32^ This reflects the fact that tail arrangement in QACs is structurally unordered with respect to the reconstruction objective. This augmentation reduces sequence-order bias during training; moreover, because the assembly rule is order-insensitive (core and tails are merged at the shared quaternary nitrogen attachment site), the final assembled molecule is invariant to any permutation of tail order.

### Property Prediction Model

For bioactivity prediction, we employ feature-based molecular representations in conjunction with shallow machine learning models that are more suitable in the presence of limited training/input data. The feature-based representation uses 35 molecular descriptors identified as important through a feature-based analysis over a total of 56 descriptors^33^ identified as important for QACs in our wet laboratories over the years.

#### Molecular Descriptors

The 35 molecular descriptors include the number of aromatic rings, the number of tails in a QAC, the number of heavy atoms, the molecular weight, and other physicochemical characteristics that can be calculated from the SMILE string corresponding to a given QAC via SwissADME.^34^ If we were to categorize them, the descriptors span six major categories: physicochemical properties, lipophilicity, water solubility, pharmacokinetics, druglikeness, and medicinal chemistry. It is worth noting that descriptors related to lipophilicity (e.g., Consensus LogP and SILICOS-IT LogP) and polarity (e.g., TPSA and hydrogen bonding capacity) are particularly informative for distinguishing active compounds and guiding the design of novel QACs.

#### Target Predicted Value

Using the feature-based representation, each QAC molecule is then described as a vector *x* of 35 molecular descriptors. The target value to predict given a vector *x* is the associated MIC value of the corresponding QAC (represented by *x*) against a particular bacterium. Four separate predictive models were trained on the curated data set, each trained to predict MIC value against one of the four bacterial strains, *Staphylococcus aureus* (SA), *Enterococcus faecalis* (EF), *Escherichia coli* (EC), and *Pseudomonas aeruginosa* (PA), respectively. The MIC values for the QACs in our training data set have been obtained via wet laboratory tests.^7–10,12,13,35–40^

#### Model Training and Selection

Using the feature-based representation, each QAC molecule in the curated data set is described as a vector *x* of 35 molecular descriptors with an associated binary label *y* ∈ {0,1} indicating activity status. Multiple classification models were evaluated using an 80/20 setup with 5-fold cross-validation on the 80% training portion and a held-out 20% test set: K-nearest neighbors (KNN), decision tree (DT), random forest (RF),^41^ stochastic gradient descent (SGD), support vector machine (SVM), extreme gradient boosting (XGB), linear regression (LR), Naïve Bayes (NB), and gradient boosting (GB). These models were implemented through Python’s scikit-learn library with default hyperparameters. No additional dimensionality reduction was performed.

Models were evaluated using Area Under the Receiver Operating Characteristic Curve (AUROC) and Area Under the Precision-Recall Curve (AUPRC). Table 1 summarizes cross-validation performance on the 80% training portion (mean ± standard deviation across 5 folds), showing that the RF model outperformed all others in terms of both AUROC (0.8036) and AUPRC (0.9672). Consequently, the RF model was selected as the predictive filter for Workflow 2. Its final performance on the held-out 20% test set was AUROC (0.5592) and AUPRC (0.9121).

**Table 1:** Performance of Classification Models under 5-Fold Cross-Validation on the 80% Training Portion (Mean ± Standard Deviation across Folds)^a^

Model	AUROC (mean ± std)	AUPRC (mean ± std)
RF	**0.8036** ± **0.0552**	**0.9672** ± **0.0079**
GB	0.7648 ± 0.0897	0.9604 ± 0.0165
XGB	0.7636 ± 0.0735	0.9606 ± 0.0130
LR	0.6740 ± 0.1279	0.9300 ± 0.0415
KNN	0.6691 ± 0.0354	0.9256 ± 0.0118
NB	0.6343 ± 0.1116	0.9245 ± 0.0368
DT	0.6241 ± 0.0779	0.9151 ± 0.0171
SVM	0.5678 ± 0.1555	0.9030 ± 0.0409
SGD	0.5077 ± 0.0747	0.8849 ± 0.0325

a The Random Forest (RF) model was selected based on superior AUROC and AUPRC metrics.

#### Interpretation of AUROC vs AUPRC

The divergence between AUROC and AUPRC on the held-out test set is informative in our setting, where the active class is relatively rare and the practical goal is enrichment (prioritizing a small subset of candidates for synthesis/testing). In such cases, AUPRC more directly reflects performance on the positive class (precision–recall behavior), whereas AUROC reflects overall ranking/separation across both classes and can be less informative and more sensitive to split composition in a small data set. Thus, the lower AUROC suggests weaker global ranking on this particular held-out split, while the high AUPRC indicates the model can still prioritize an enriched subset of likely active compounds, which is the intended use case. Finally, the experimental validation of selected compounds provides an external, prospective test of the workflow’s practical utility.

## Implementation Details

The models were implemented in Python using PyTorch for neural network components and scikit-learn for the property prediction model. All experiments were conducted on a system with NVIDIA GeForce RTX 3090 GPUs.

### Generator Hyperparameters

The core and tail modules use hierarchical VAEs with hidden size 100, embedding size 100, and latent size 8. The global sequence module is a GRU encoder/decoder with hidden size 100, 3 layers, and latent size 8; an ⟨EOS⟩ token marks sequence termination. Core/tail VAEs are pretrained with learning rate 1e^–3^, batch size 50, for 400 epochs; the GRU-VAE is trained with learning rate 5e^–4^, batch size 32, for 300 epochs. The loss weight λ in the training objective is set to 1e^–6^.

## Expert Evaluation Protocol

Both workflows imposed a practical constraint of approximately 4 h of expert chemistry evaluation time per campaign. This constraint reflects realistic resource allocation in discovery settings where domain experts must balance computational candidate review with ongoing synthesis and characterization activities.

### Expertise

For the evaluation, we used the efforts of one expert (KPCM) with a PhD in organic synthesis and ∼95 peer-reviewed publications in the field of organic and medicinal chemistry, with >50 of these publications in the general field of quaternary ammonium compounds over a ∼15 year span, with a focus on structure–activity analysis.

### Decision Criteria

Expert evaluation applied systematic chemistry-domain criteria organized as a decision tree. Candidates were classified into six categories: (1) “Not A Compound” for chemically invalid structures, (2) “Not A QAC” for molecules lacking a charged nitrogen core, (3) “Already Made” for structures present in the training data set, (4) “Too Simple/Complex” for structures offering insufficient novelty within the QAC domain or excessive synthetic complexity, (5) “Incremental Change” for minor variations of known QACs, such as adding one or two carbons in a chain, or making existing structures slightly asymmetric, and (6) “Worth Making” for novel, synthetically accessible candidates with predicted biological relevance.

### Time Allocation

Approximately 4 h were spent by the expert on evaluating 300 generated compounds. A substantial fraction of the time window was spent on rapid triage and placement within categories 1–5, with categories 3–5 requiring repeated checking versus the training set. Deeper deliberation was reserved for placement into category 6, “Worth Making,” which involves structural assessment for both synthetic accessibility and anticipated antimicrobial activity.

#### Workflow 1: Sampling Strategy

Generated candidates were sampled for evaluation starting from a random position in the generated set. Expert proceeded sequentially through candidates, applying decision criteria to each molecule until the 4 h time budget was exhausted. This approach enabled evaluation of 300 candidates from the generated pool.

#### Workflow 2: Ranking Strategy

Generated candidates were first filtered by the RF classifiers (one per bacterial strain) to retain only those predicted highly active (MIC ≤ 8 μM) against at least one of the four bacterial strains. Retained candidates were ranked by the number of strains against which they were predicted active (range: 1–4 strains). Expert evaluated candidates in descending order of this ranking, applying the same decision criteria as Workflow 1 until the 4 h budget was exhausted. This approach again enabled evaluation of 300 candidates selected from approximately 800 top-ranked molecules in the filtered pool.

## Wet-Laboratory Synthesis and Characterization

Selected candidates from both workflows were synthesized using standard organic chemistry techniques and characterized for antimicrobial activity through minimum inhibitory concentration (MIC) assays against bacterial pathogens. Detailed synthetic protocols and biological assay procedures are provided in the Supporting Information.

## Results and Discussion

We report here on the results from two independent experimental workflows designed to evaluate the impact of predictive filtering on expert-guided molecular selection. We refer to the QACs synthesized and evaluated in the wet laboratory for each workflow as Workflow 1 and Workflow 2.

### Workflow 1: Direct Expert Evaluation

Of the compounds generated by the topology-aware VAE in Workflow 1 (May 13, 2022), 300 were analyzed to generate statistics regarding the types of compounds proposed by the computational module. We relate these statistics in Figure 4.

**Figure 4:**
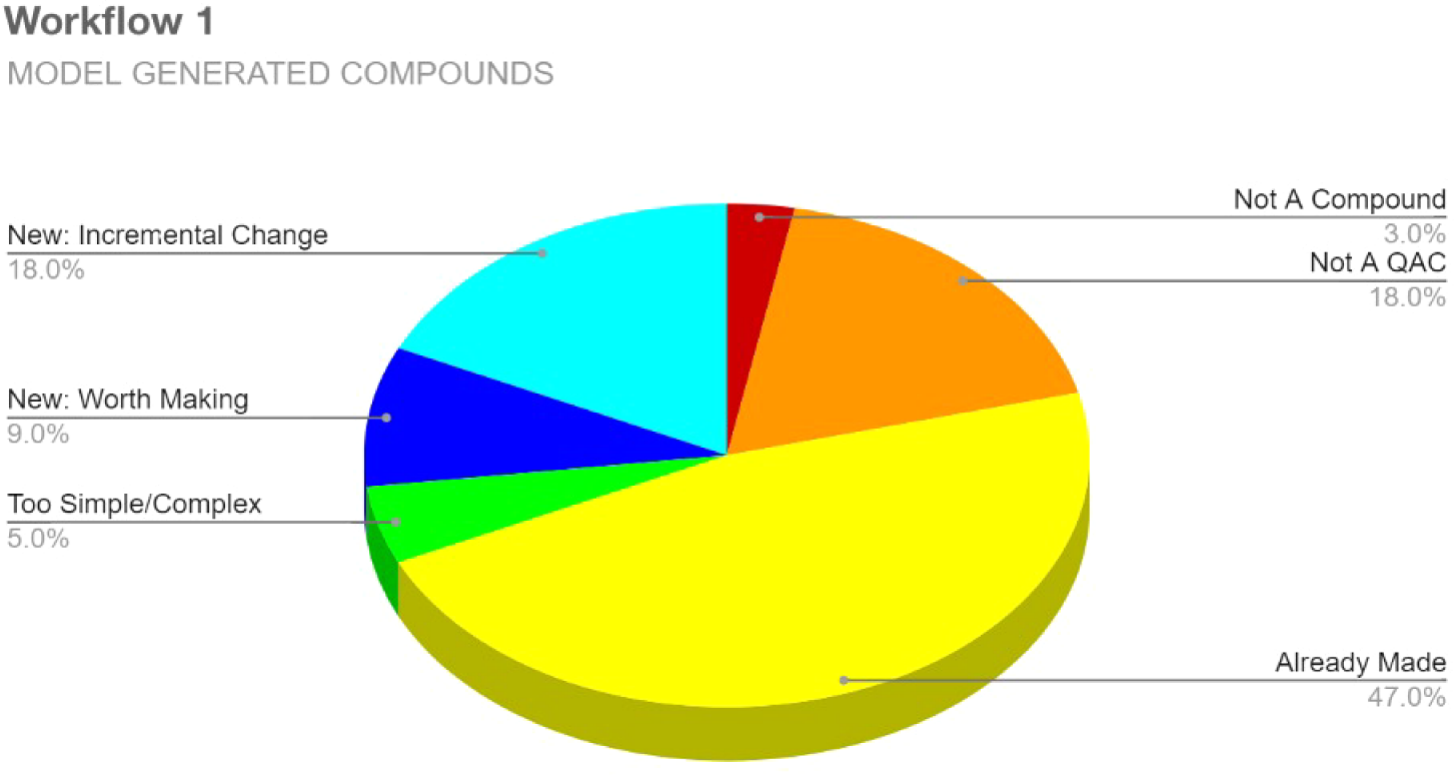
Expert evaluation statistics for Workflow 1 candidates. Analysis of 300 compounds sampled from generated molecules within a 4 h expert evaluation constraint.

As Figure 4 shows, we employed six categories to classify each compound, including “Not A Compound” for those that were chemically invalid (i.e., nonsensical) and “Not A QAC” for those that did not possess a charged nitrogen core; in this batch, simple compounds like decane and toluene appeared repeatedly. These categories were defined and applied by K.P.C.M. based on expertise in organic chemistry and QAC structure–activity relationships. These two categories collectively constituted 21% of the evaluated compounds in Workflow 1. The largest percentage at 47% was the “Already Made” category, which consisted of compounds from the training data set. The “Too Simple/Complex” category had a modest 5% and included simple tetraalkyl QACs. The total percentage of novel structures was 27%, with 18% being compounds deemed to represent only incremental changes, such as the addition of extra carbons to the side chains of known QACs, and 9% being the desired category of compounds “Worth Making.”

Drawing from Workflow 1, a total of ten QACs, illustrated in Table 2, were chosen for synthesis using traditional synthetic organic chemistry techniques, as employed in previous publications.^40,42^ These were prioritized as relatively straightforward to synthesize and anticipated—based on expert review—to exhibit significant biological activity. Importantly, nine of these ten were unreported in the chemical literature, as indicated by a CAS SciFinder search; one structure was known and is so designated in Table 2. After wet laboratory preparation, which is described in detail in the Supporting Information, these ten compounds were tested for bioactivity at Emory University (W.M.W.) against a range of bacterial pathogens to determine their minimum inhibitory concentration (MIC) values in μM. In addition to the ten synthesized compounds, three simple non-QAC compounds generated by the model were also tested, as was one control QAC compound—benzalkonium chloride (a commercially used active disinfectant). The commercially purchased compounds (decane, toluene, and undecylamine) were included to provide baseline data on non-QAC structures, and are designated as “Control” in Table 2.

**Table 2:** Antibacterial Activity for Workflow 1 Synthesized and Characterized Compounds^a^

Minimum Inhibitory Concentration (MIC) (μM)								
Compound name	MSSA	CA-MRSA	HA-MRSA	*E. faecalis*	*E. coli*	*A. baumannii*	*P. aeruginosa*	Lysis20
**Control compounds**								
BAC	2	2	16	32	32	16	125	32
Toluene	>250	>250	>250	>250	>250	>250	>250	NA
Decane	>250	>250	>250	>250	>250	>250	>250	NA
Undecylamine	250	125	125	250	250	250	>250	NA
**Known structures**								
16,12	32	8	16	16	16	>250	>250	NA
**Novel structures**								
Allyl,pO12Bn	1	1	16	32	32	32	250	16
PIP-10,12A	1	1	16	16	16	>250	16	125
4N-16,14	4	8	16	16	16	125	32	8
mBrPyr-15A	4	4	16	16	16	>250	>250	NA
mBrPyr-14A	16	16	63	63	63	63	250	125
OX-11,1	16	16	63	63	63	125	250	250
ACP-10	16	16	63	125	125	125	>250	250
Bn-11E	>250	>250	>250	>250	>250	>250	>250	>250
mN1-Pyr-1	>250	>250	>250	>250	>250	>250	>250	250

a BAC is benzalkonium chloride, a known disinfectant serving as a control compound. NA means not available (not tested)

The most potent compounds from this workflow had MIC values between 1–32 μM for most bacterial strains, with the occasional exception being one of the three Gram-negative bacteria tested. This outcome is expected, as Gram-negative bacteria are inherently more difficult to eliminate due to their protective outer membrane. Additionally, consistent with previous observations, the compounds most effective against bacteria were also effective at lysing red blood cells. For the four most potent compounds, their Lysis_20_ values were 125 μM or below, with as low as 8 μM for 4N-16,14, suggesting moderate to strong cytotoxicity. The three purchased non-QAC compounds displayed poor MIC values, with most exceeding 250 μM, as expected for simple molecules lacking the QAC topology.

### Workflow 2: Predictive Filtering Prior to Expert Evaluation

For Workflow 2, the trained generator model was used to produce approximately 2,000 candidate molecules (April 18, 2024).

Several computational enhancements were implemented in this workflow. First, a structure validity check was performed to retain only chemically valid generated molecules. Second, the property prediction model (Random Forest classifier) was used to classify generated compounds as active (1) or inactive (0) based on a threshold of ≤ 8 μM for each of the four bacterial strains. Of the approximately 2,000 generated compounds, 800 met the active criterion for at least one strain. From these, the top 300 candidates, ranked by the number of strains against which they were predicted active, were presented to the wet laboratory for expert evaluation within the same 4 h constraint as Workflow 1. The resulting filtered set was delivered as SMILES files.

As with Workflow 1, a statistical analysis was performed on the 300 expert-evaluated compounds from Workflow 2 after thorough inspection applying the same decision criteria. The analysis is presented in Figure 5.

**Figure 5:**
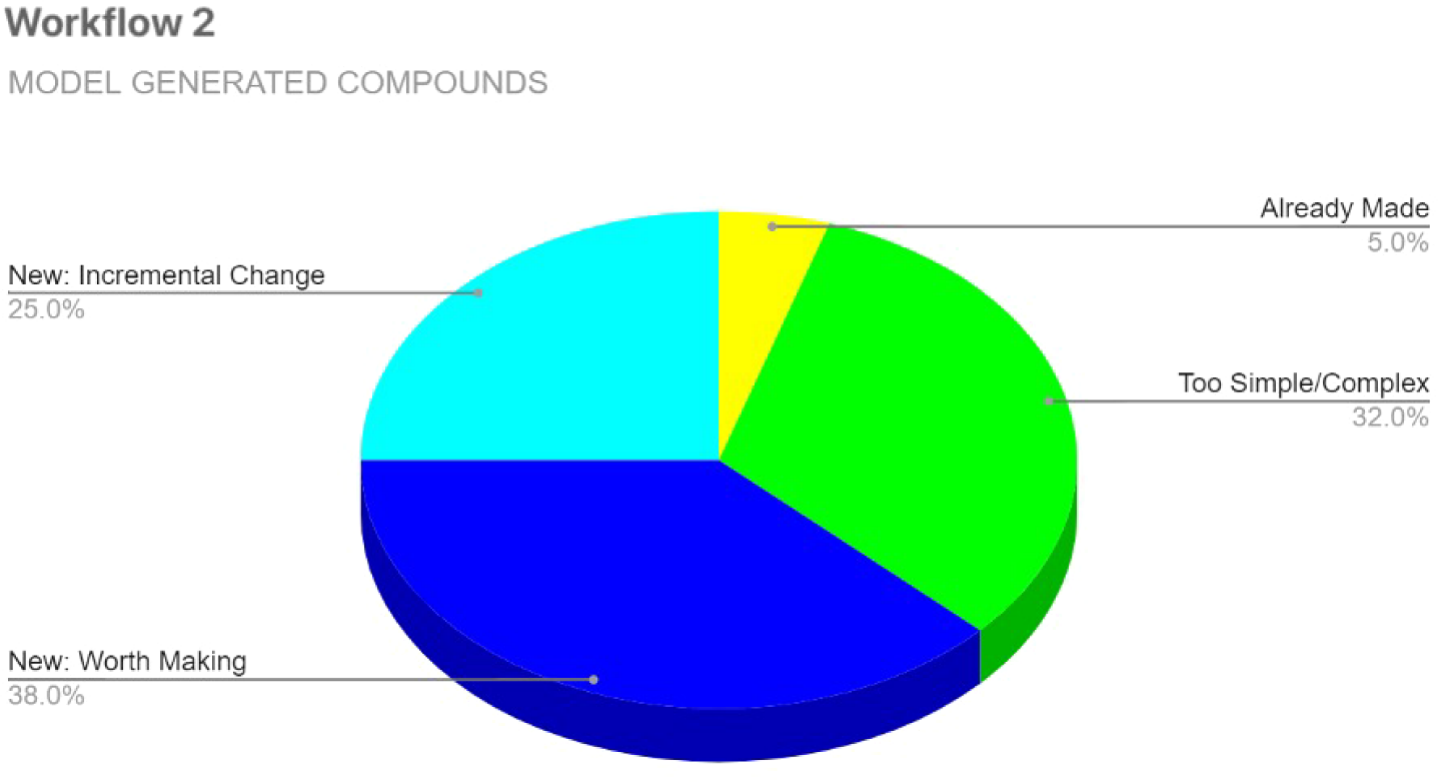
Expert evaluation statistics for Workflow 2 candidates. Analysis of 300 top ranked compounds from the predictively filtered pool of approximately 800 molecules, evaluated within the same 4 h expert evaluation constraint.

Gratifyingly, there was clear improvement in Workflow 2. Notably, the number of undesirable outputs categorized as “Not a Compound” or “Not a QAC” dropped to 0%, due in part to the structure validity check on generated compounds and the filtering effect of bioactivity-based selection, which favored QAC-like molecules. The percentage of compounds labeled “Already Made” also decreased significantly, from 47% in Workflow 1 (Figure 4) to just 5% in Workflow 2, reflecting the effect of bioactivity-based selection in promoting structural novelty. Most significantly, the proportion of novel QACs increased from 27% to 63%, and those considered worth synthesizing jumped from 9% to 38%.

From Workflow 2, a total of 19 QACs were selected for synthesis using standard techniques, as specified in the Supporting Information, and tested for biological activity. Three of the compounds were designated as “known” in light of a CAS SciFinder search, the remaining 16 were unreported in the chemical literature. The results are shown in Table 3, and further compound-by-compound novelty explanation is provided in the Supporting Information.

**Table 3:** Antibacterial Activity for Workflow 2 Synthesized and Characterized Compounds

Minimum Inhibitory Concentration(MIC) (μM)								
Compound name	MSSA	CA-MRSA	HA-MRSA	*E. faecalis*	*E. coli*	*A. baumannii*	*P. aeruginosa*	Lysis20
**Control compounds**								
BAC	2	2	16	32	32	16	125	32
**Known structures**								
pCl-Bn-10	16	16	125	63	63	125	250	250
oCl-Bn-10	16	16	125	250	250	250	>250	>250
Paraquat-4,4	>250	>250	>250	>250	>250	>250	>250	250
**Novel structures**								
oOC9-Bn-10	1	2	4	8	8	8	32	16
mOC6-Bn-8	2	2	16	32	32	16	125	63
Bn-pOC9-Bn	2	2	32	32	32	16	125	63
pOC6-Bn-8	2	2	16	32	32	16	125	63
DPE-11E,11E	2	2	32	32	32	16	125	63
Paraquat-11-mOC1-Bn	4	8	32	16	16	250	125	250
Pyr-mOC9	4	4	32	125	63	32	250	125
DPA-9,9E	8	16	32	32	32	125	125	125
EtBn-pOC9Bn	16	16	63	125	250	125	250	250
8(3)0(3)8	16	32	125	125	125	>250	250	250
3,5-methoxy-Bn-10	16	32	125	250	>250	250	>250	250
DPA-8,mOC6Bn	32	8	32	32	32	125	125	250
2,3-methoxy-Bn10	32	32	250	>250	>250	>250	>250	250
oX-8,8	32	63	250	250	250	>250	>250	250
3,4-methoxy-Bn10	125	125	>250	>250	>250	>250	>250	250
Urea^–9^,pClPh	>250	>250	>250	>250	>250	>250	>250	250

The most effective compounds were mOC6-Bn-8, Bn-pOC9-Bn, oOC9-Bn-10, pOC6-Bn-6, DPE-11E,11E, Paraquat-11,3-mOC1-Bn, and Pyr-mOC9, as shown in Table 2. These QACs showed MIC values ranging from approximately 1 to 32 μM against the majority of bacterial strains. We note that 14 of the 19 tested compounds had MICs below 16 μM against MSSA (methicillin-susceptible *S. aureus*). Somewhat surprisingly, only 4 of these retained activity against HA-MRSA, demonstrating that further work is needed to overcome the challenges presented by this resistant and pathogenic strain.^43,44^ In addition, their activity was less impressive against Gram-negative species, as observed in Workflow 1, which is consistent with the known resistance of Gram-negative bacteria due to their additional outer membrane. As seen in Workflow 1, the strongest antibacterial compounds also tended to exhibit higher cytotoxicity, with lower Lysis_20_ values indicating a greater tendency to lyse red blood cells. For the top seven performers, Lysis_20_ values were all at or below 125 μM, except for Paraquat-11,3-mOC1-Bn, which had a value of 250 μM, indicating lower cytotoxicity.

## Comparative Analysis

Figure 6 shows the molecular structures of all QAC compounds synthesized and characterized from Workflow 1 and Workflow 2. The most bioactive compounds (deemed “Top Compounds”) for each workflow are boxed in green and illustrates the improvement achieved through predictive filtering, with more compounds populating the “Top Compounds” category in Workflow 2.

**Figure 6:**
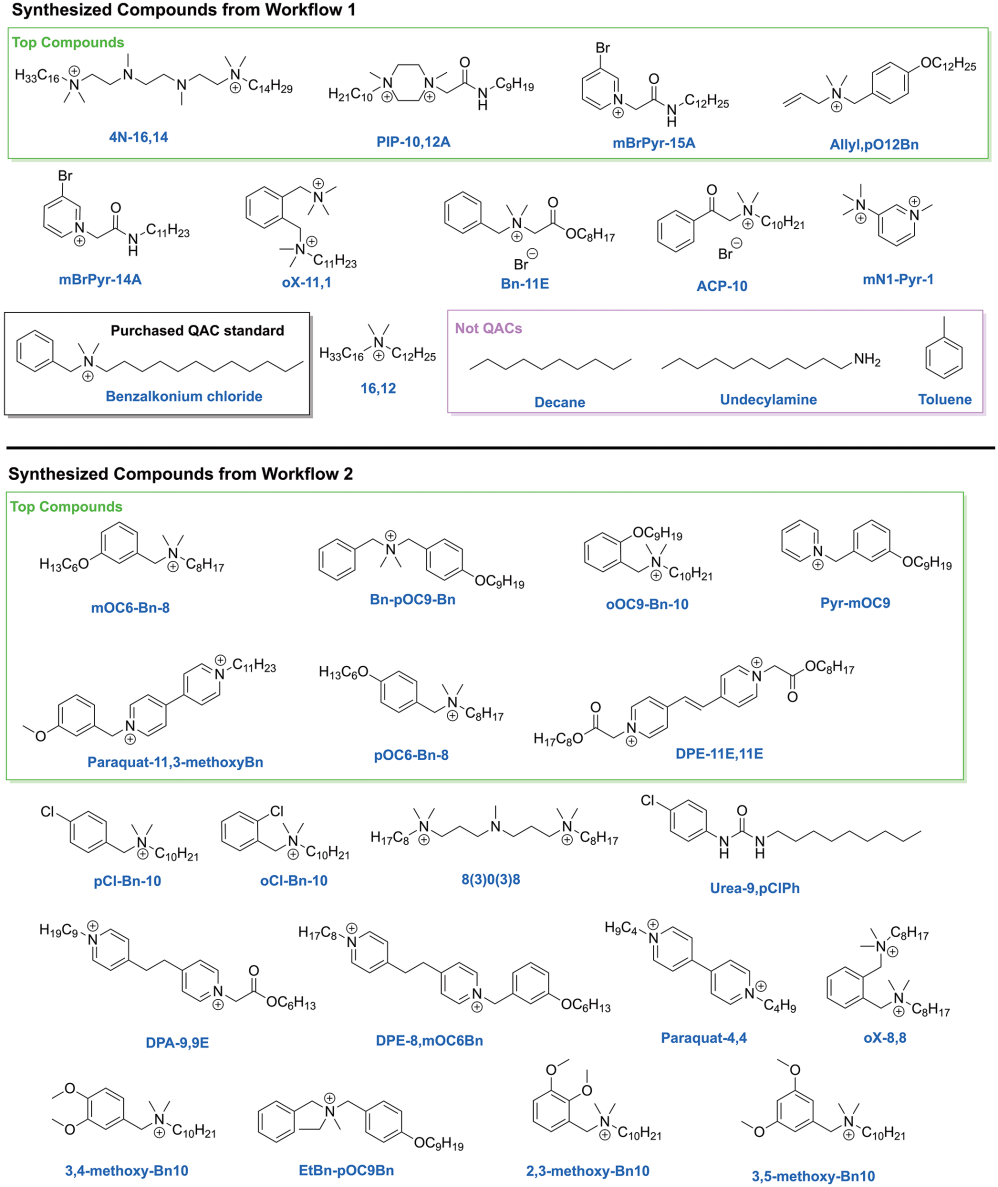
All computationally suggested QAC compounds synthesized in the two experimental workflows. The most bioactive compounds for each workflow are boxed in green and titled “Top Compounds”, and the additional non-QAC structures tested in Workflow 1 are highlighted in a pink box.

The comparative evaluation reveals substantial improvements in candidate quality when predictive filtering is incorporated into the workflow. Synthesis-worthy candidates increased from 9% to 38%, while chemically invalid outputs decreased from 21% to 0%. Redundant structures from the training data set decreased from 47% to 5%. Overall, novel QACs increased from 27% to 63% of evaluated candidates. From Workflow 1, 4 of 10 synthesized compounds (40%) exhibited potent antimicrobial activity (MIC 1–32 μM), while Workflow 2 yielded 7 of 19 compounds (37%) with potent activity. In total, 11 novel QACs with significant antibacterial activity were discovered across both workflows.

These results must be interpreted considering several factors. The dramatic reduction in invalid outputs (21% to 0%) and already-synthesized structures (47% to 5%) suggests that computational prefiltering, even with a relatively simple Random Forest classifier, substantially improves the efficiency of expert evaluation within fixed time constraints. The 4 h evaluation window enabled assessment of 300 compounds sampled from thousands in Workflow 1 (approximately 10% coverage of generated molecules) versus 300 top-ranked compounds from approximately 800 filtered candidates in Workflow 2 (approximately 37.5% coverage of the filtered set). In addition, there are only a few training compounds bearing a chloride, and we built three aryl chlorides in our set. The methoxy compounds and 8(3)­0(3)­8 mimic some compounds in the training set with slight modifications but DPA-9,9E and Paraquat-1,3methoxyBn are chemically distinct. This improved coverage, combined with activity-based ranking, enabled experts to focus evaluation time on higher-quality candidates.

## Conclusions

This work presents a comparative experimental evaluation of two computational workflows for quaternary ammonium compound discovery under data-scarce conditions and practical resource constraints. Both workflows employed a topology-aware variational autoencoder capable of generating chemically valid QACs while respecting their distinctive hierarchical architecture, a permanently charged nitrogen core with variable hydrophobic tails. The workflows differed in their approach to candidate prioritization: Workflow 1 submitted generated candidates directly for expert evaluation, while Workflow 2 introduced predictive filtering using a Random Forest classifier trained to identify compounds with predicted antimicrobial activity. Under fixed time constraints (approximately 4 h of expert evaluation per workflow), predictive filtering demonstrated substantial benefits. The proportion of synthesis-worthy candidates increased from 9% to 38%, chemically invalid outputs decreased from 21% to 0%, and redundant structures decreased from 47% to 5%. Overall, novel QACs increased from 27% to 63% of evaluated candidates, enabling more efficient use of limited expert time.

Wet-laboratory validation provided experimental confirmation of workflow effectiveness. From Workflow 1, 10 candidates were synthesized and characterized, yielding 4 compounds with potent antimicrobial activity (MIC 1–32 μM against most bacterial strains). From Workflow 2, 19 candidates were synthesized, yielding 7 potent antimicrobials. In total, 11 novel QACs with significant antibacterial activity were discovered across both workflows, expanding the characterized chemical space and providing new lead compounds for further development. The dramatic improvements in candidate quality in Workflow 2, particularly the elimination of invalid outputs and substantial reduction in redundant structures, suggest that computational prefiltering substantially improves expert evaluation efficiency within practical time budgets. This finding has important implications for molecular discovery in data-scarce domains where expert evaluation represents a critical bottleneck.

Future work may extend this framework in several directions. Expanding predictive models to simultaneously predict activity against resistant pathogens and toxicity to human cells would enable multiobjective optimization of therapeutic indices. Incorporating synthetic accessibility prediction alongside activity prediction could further improve expert evaluation efficiency by filtering synthetically intractable candidates. Systematic investigation of the relationship between training data set size, predictive model performance, and workflow effectiveness would clarify the conditions under which predictive filtering provides maximum benefit. The experimental comparison presented here demonstrates that thoughtful integration of predictive modeling into expert-guided workflows can accelerate molecular discovery under realistic resource constraints, offering a practical strategy for navigating vast chemical spaces in the search for novel therapeutics.

## Supplementary Material





## Notes

Detailed wet-laboratory protocols, additional figures, tables, and raw data can be found in the Supporting Information­(PDF)
Dataset (XLSX)
