# Supplementary material for: Topology-Aware Generation and Activity-Based Filtering: A Computational-Experimental Framework for Data-Scarce Quaternary Ammonium Compound Discovery
Source: J Chem Inf Model. 2026 Mar 5;66(6):3117–28. doi: 10.1021/acs.jcim.6c00390 (PMC13014459; doi:10.1021/acs.jcim.6c00390)
Supplement: Supplementary file 1 [file ci6c00390_si_001.pdf]

Supporting Information

Topology-Aware Generation and Activity Based Filtering: A Computational-Experimental Framework for Data-Scarce Quaternary Ammonium Compound Discovery

Shiva Ghaemi,[a] Amanda J. Consylman,[b] Bo Pan,[c] Alice Wu, [b] Ashley Petersen, [b] Gabe Chang,[b] Diana McDonough, [b] Mark Forman, [d] Elise L. Bezold,[e] William M. Wuest,*[e] Amarda Shehu, * [a] Liang Zhao,*[c] and Kevin P. C. Minbiole *[b].

[a] *Department of Computer Science, George Mason University, Fairfax, VA, USA*

[b] *Department of Chemistry & Biochemistry, Villanova University, Villanova, PA, USA*

[c] *Department of Computer Science, Emory University, Atlanta, GA, USA*

[d] *Department of Chemistry, St. Joseph's University, Philadelphia, PA, USA*

[e] *Department of Chemistry, Emory University, Atlanta, GA, USA*

**Corresponding authors*

*Email: kevin.minbiole@villanova.edu; wwuest@emory.edu; amarda@gmu.edu; liang.zhao@gmu.edu

Table of Contents

I. General Information S2
II. Statistical Analysis S2
III. Table S1: Smiles Files of Novel Compounds S5
IV. Biological Assays S5
V. Synthetic Procedures S7
VI. NMR Spectroscopic Data: ^{1}H and ^{13}C NMR S111
VII References........ S111

I. General Information

All reagents and solvents were from Sigma-Aldrich, TCI Chemicals, Ambeed, Oakwood Chemical, and ThermoFisher Scientific and were used without further purification. Reactions were run on a pie block (Chemglass) with reagent grade solvents and magnetic stirring. All yields refer to spectroscopically pure compounds. ^{1}H and ^{13}C NMR spectra were measured with a 400 MHz or 500 MHz JEOL spectrophotometer, and chemical shifts were reported on a δ-scale (ppm) downfield from TMS. Coupling constants were calculated in hertz (Hz). The solvent used for NMR spectroscopy was chloroform-D ($CDCl_3$) with chemical shifts internally referenced to the residual solvent peak of 7.26 ppm (^{1}H NMR) and 77.16 ppm (^{13}C NMR). Accurate mass spectrometry data was acquired on an AB Sciex 5600 TripleTOF using electrospray ionization in positive mode.

II. Statistical Analysis

To determine whether observed workflow differences reflect more than sampling variability, we model each expert-inspected candidate as a Bernoulli trial for each endpoint (e.g., "Worth Making" vs. not) within a fixed sample of n = 300 candidates per workflow. For each endpoint, we estimate the workflow-specific proportion $\hat{p} = k/n$ (with k successes) and report a 95% confidence interval (CI) using the Wilson score method, which inverts the score test for a binomial proportion and is well-behaved for finite samples and near-boundary proportions.[52,53] As effect sizes, we report the absolute risk difference $\Delta p = \hat{p}\text{w2} - \hat{p}\text{w1}$(percentage-point change), the risk ratio RR $= \hat{p}\text{w2}/\hat{p}\text{w1}$ (multiplicative change in probability), and the odds ratio $\text{OR} = \frac{\hat{p}\text{w2}/(1-\hat{p}\text{w2})}{\hat{p}\text{w1}/(1-\hat{p}\text{w1})}$ (multiplicative change in odds). Statistical significance is assessed using a two-sided Fisher exact test on the corresponding 2 × 2 contingency table (Workflow 1 vs. Workflow 2 by "in category" vs. "not in category"), yielding an exact p-value for the null

hypothesis of equal proportions without relying on large-sample approximations.[54] When a cell count is zero, we compute OR using the standard Haldane–Anscombe 0.5 continuity correction to avoid division by zero.[55]

1) "Worth Making" **Rates:** 9% → 38%.

Counts: W1 27/300, W2 114/300. W1 $\hat{p}$ = 0.090; 95% CI [0.063, 0.128].

W2 $\hat{p}$ = 0.380; 95% CI [0.327, 0.436].

Δp = +0.290 (absolute +29.0 percentage points).

RR = 4.22.

OR ≈ 6.20.

Fisher $p = 1.70 \times 10^{-17}$.

Interpretation: Workflow 2 yields a large, statistically decisive increase in "Worth Making" candidates under the same 300-candidate inspection budget.

2) "Already Made" **Rates:** 47% → 5%.

Counts: W1 141/300, W2 15/300.

W1 $\hat{p}$ = 0.470; 95% CI [0.414, 0.526].

W2 $\hat{p}$ = 0.050; 95% CI [0.031, 0.081].

Δp = −0.420 (absolute −42.0 percentage points).

RR = 0.106.

OR ≈ 0.059.

Fisher $p = 1.14 \times 10^{-34}$.

Interpretation: Workflow 2 dramatically reduces redundancy ("Already Made") among inspected candidates.

3) "Not a Compound" OR "Not a QAC" **Rates:** 21% → 0%.

Counts: W1 63/300, W2 0/300.

W1 $\hat{p}$ = 0.210; 95% CI [0.168, 0.260].

W2 $\hat{p} = 0.000$; 95% CI [0.000, 0.0126].

$\Delta p = -0.210$.

RR = 0.0.

OR ≈ 0.0062 (continuity-corrected).

Fisher $p = 5.68 \times 10^{-21}$.

Interpretation: Workflow 2 shows a complete elimination (within 300) of the invalid-output classes, consistent with the reported filtering/validity checks.

4) Novel QACs **Rates:** 27% → 63%.

Counts: W1 81/300, W2 189/300.

W1 $\hat{p} = 0.270$; 95% CI [0.223, 0.323].

W2 $\hat{p} = 0.630$; 95% CI [0.574, 0.683].

$\Delta p = +0.360$.

RR = 2.33.

OR ≈ 4.60.

Fisher $p = 6.55 \times 10^{-19}$.

Interpretation: Workflow 2 produces a large shift toward novelty among inspected candidates.

III. Table S1: Smiles Files of Novel Compounds

WORKFLOW 1

SMILES	Source/notebook	Compound name	New to us?	New to SciFinder?	Tested?	Reference
CC1=CC=CC=C1	Purchased	Toluene	No/control	No		
CCCCCCCCCC	Purchased	Decane	No/control	No		
CCCCCCCCCCCN	Purchased	Undecylamine	No/control	No		
C[N+](CCN(C)CCN(C)CC[N+](C)(C)CCCCCCCCCCCCCC)(C)CCCCCCCCCCCCCCCC	AAP149	4N-16,14	Yes	Yes		
C[N+]1(CC(NCCCCCCCCCC)=O)CC[N+](C)(CCCCCCCCCC)CC1	AAP152	PIP-10,12A	Yes	Yes		
BrC1=CC=C[N+](CC(NCCCCCCCCCCCCCCC)=O)=C1	AW39	mBrPyr-15A	Yes	Yes		
C[N+](C)(CCCCCCCCCCCCCCCC)CCCCCCCCCCCC	AW42	16,12	Yes	No	No	J. Colloid and Interface Science (2006), 297(1), 284.
BrC1=CC=C[N+](CC(NCCCCCCCCCCCCCC)=O)=C1	AC6	mBrPyr-14A	Yes	Yes		
C[N+](C)(C)CC1=CC=CC=C1C[N+](C)(CCCCCCCCCCC)C	AW51	oX-11,1	Yes	Yes		
O=C(OCCCCCCCCCCC)C[N+](C)(C)CC1=CC=CC=C1	AW50	Bn-11E	Yes	Yes		
O=C(C[N+](C)(C)CCCCCCCCCC)C1=CC=CC=C1	AC17	ACP-10	Yes	Yes		
C[N+]1=CC([N+](C)(C)C)=CC=C1	DR-1-051	mN1-Pyr-1	Yes	Yes		
C=CC[N+](C)(CC1=CC=C(OCCCCCCCCCCCC)C=C1)C	AC32	Allyl,pO12Bn	Yes	Yes		

WORKFLOW 2

SMILES	Source/notebook	Compound name	New to us?	New to SciFinder?	Tested?	Reference
C[N+](C)(Cc1ccc(Cl)cc1)CCCCCCCCCC	AC39	pCl-Bn-10	Yes	No	Yes	J. Colloid Science (1953), 8, 385.
C[N+](C)(Cc1cc(OCCCCCC)ccc1)CCCCCCCC	AC40	mOC6-Bn-8	Yes	Yes		
C[N+](C)(Cc1ccccc1)Cc2ccc(OCCCCCCCCC)cc2	AC41	Bn-pOC9-Bn	Yes	Yes		
C[N+](C)(CCCN(CCC[N+](C)(CCCCCCCC)C)C)CCCCCCCC	AC42	8(3)0(3)8	Yes	Yes		
C[N+](C)(Cc1c(C[N+](C)(CCCCCCCC)C)cccc1)CCCCCCCC	AC44	oX-8,8	Yes	Yes		
C[N+](C)(Cc1c(OCCCCCCCCC)cccc1)CCCCCCCCCC	AC46	oOC9-Bn-10	Yes	Yes		
C[N+](C)(Cc1c(Cl)cccc1)CCCCCCCCCC	MAF-009	oCl-Bn-10	Yes	No	Yes	J. Colloid Science (1953), 8, 385.
C[N+](C)(Cc1ccc(OCCCCCC)cc1)CCCCCCCC	MAF-010	pOC6-Bn-8	Yes	Yes		
O=C(OCCCCCCCCC)C[n+]1ccc(CCc2cc[n+](CCCCCCCCC)cc2)cc1	MAF-012	DPA-9,9E	Yes	Yes		
C[N+](C)(Cc1c(OC)c(OC)ccc1)CCCCCCCCCC	AC-52	2,3-methoxy-Bn10	Yes	Yes		
C[N+](C)(Cc1cc(OC)c(OC)cc1)CCCCCCCCCC	AC-60	3,4-methoxy-Bn10	Yes	Yes		
O=C(C[n+]1ccc(C=Cc2cc[n+](CC(OCCCCCCCCCCC)=O)cc2)cc1)OCCCCCCCCCCC	AC-59	DPE-11E,11E	Yes	Yes		
C[N+](CC)(Cc1ccccc1)Cc2ccc(OCCCCCCCCC)cc2	AC-73	EtBn-pOC9Bn	Yes	Yes		
CCCCCCCCCNC(Nc1ccc(Cl)cc1)=O	MAF-019	Urea-9,pClPh	Yes	Yes		
COC1=CC=CC(C[N+]2=CC=C(C=C2)C3=CC=[N](C=C3)CCCCCCCCCCC)=C1	AC80(AC71)	Paraquat-11-mOC1-Bn	Yes	Yes		
CCCCCCOC1=CC(C[N+]2=CC=C(C=C2)CCC3=CC=[N+](CCCCCCCC)C=C3)=CC=C1	AC81	DPA-8,mOC6Bn	Yes	Yes		
CCCC[N+]1=CC=C(C=C1)C2=CC=[N+](C=C2)CCCC	AC87	Paraquat-4,4	Never published	No	Yes	Drug and Chemical Toxicology (1977) (1979), 2(3), 193.
COC1=CC(OC)=CC(C[N+](C)(C)CCCCCCCCCC)=C1	AC89	3,5-methoxy-Bn-10	Never published	Yes		
CCCCCCCCCOC1=CC(C[N+]2=CC=CC=C2)=CC=C1	AC92	Pyr-mOC9	Yes	Yes		

IV. Biological Assays

For all biological assays, laboratory strains of methicillin-susceptible *Staphylococcus aureus* MSSA (SH1000), *Enterococcus faecalis* (OG1RF), *Escherichia coli* (MC4100), *Pseudomonas aeruginosa* (PAO1), *Acinetobacter baumannii* (ATCC 17978), community-acquired methicillin-resistant *Staphylococcus aureus* CA-MRSA (USA300-0114), and hospital-acquired methicillin-resistant *Staphylococcus aureus* HA-MRSA (ATCC 33591) were grown with shaking at 37 ºC overnight from freezer stocks in 5 mL of the indicated media: SH1000, OG1RF, MC4100, USA300-0114, and PAO1 were grown in BD™ Mueller-Hinton broth (MHB), whereas ATCC 33591 was grown in BD™ tryptic soy broth (TSB). Optical density (OD) measurements were obtained using a SpectraMax iD3 plate reader (Molecular Devices, United States).

Minimum Inhibitory Concentration (MIC)

Compounds were serially diluted two-fold from stock solutions (1.0 mM) to yield twelve 100 μL test concentrations, wherein the starting concentration of dimethyl sulfoxide (DMSO) was 2.5%. Overnight *S. aureus, E. faecalis, E. coli, P. aeruginosa, A. baumannii*, USA300-0114 (CA-MRSA), and ATCC 33591 (HA-MRSA) cultures were diluted to ca. 10^6 CFU/mL in MHB or TSB and regrown to mid-exponential phase, as determined by optical density recorded at 600 nm (OD600). All cultures were then diluted again to ca. 10^6 CFU/mL and 100 μL were inoculated into each well of a U-bottom 96-well plate containing 100 μL of compound solution. Plates were incubated statically at 37 ºC for 72 h upon which wells were evaluated visually for bacterial growth. The MIC was determined as the lowest concentration of compound resulting in no bacterial growth visible to the naked eye, based on the highest value in three independent experiments. Aqueous DMSO controls were conducted as appropriate for each compound.

Red Blood Cell (RBC) Lysis Assay (Lysis$_{20}$)

RBC lysis assays were performed on mechanically defibrinated sheep blood (Hemostat Labs: DSB030). An aliquot of 1.5 mL blood was placed into a microcentrifuge tube and centrifuged at 3,800 rpm for ten min. The supernatant was removed, and the cells were resuspended with 1 mL of phosphate-buffered saline (PBS). The suspension was centrifuged as described above, the supernatant was removed, and cells were resuspended 4 additional times in 1 mL PBS. The final cell suspension was diluted twenty-fold with PBS. Compounds were serially diluted with PBS two-fold from stock solutions (1.0 mM) to yield 100 μL of twelve test concentrations on a flat-bottom 96-well plate (Corning, 351172), wherein the starting concentration of DMSO was 2.5%. To each of the wells, 100 μL of the twenty-fold suspension dilution was then inoculated. The concentration of DMSO in the first well was 2.5%, resulting in DMSO-induced lysis at all

concentrations >63 μM. TritonX (1% by volume) served as a positive control (100% lysis marker) and sterile PBS served as a negative control (0% lysis marker). Samples were then placed in an incubator at 37 ºC and shaken at 200 rpm. After 1 hour, the samples were centrifuged at 3,800 rpm for ten minutes. The absorbance of the supernatant was measured with a UV spectrometer at a 540 nm wavelength. The concentration inducing 20% RBC lysis was then calculated for each compound based upon the absorbances of the TritonX and PBS controls. Aqueous DMSO controls were conducted as appropriate for each compound.

V. Synthetic Procedures

Preparation of 4N-16, 14 (AAP149)

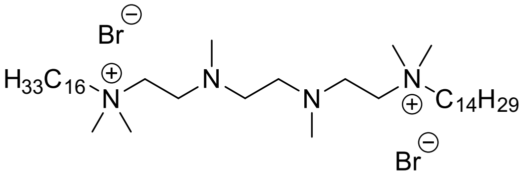

In a 25 mL round-bottom flask equipped with a stir bar, **4N-14** [1] (0.254 g, 0.500 mmol) was dissolved in acetonitrile (1 mL). 1-bromohexadecane (0.150 mL, 0.500 mmol) was added to the reaction flask via micropipette and the reaction was stirred at 80 ºC for 24 h. The resulting pale-yellow solution was removed from heat and allowed to cool to room temperature. The pale-yellow solution was then concentrated in vacuo resulting in a biphasic mixture of a white solid and a yellow oil. The reaction contents were triturated with hexanes for 1 hour and concentrated in vacuo affording **4N-16, 14** as a pale-yellow wax (0.469 g, >99%); ^{1}H NMR (500 MHz, CD_3OD) δ 3.48 (t, J = 6.5 Hz, 4H), 3.41 – 3.34 (m, 5H), 3.14 (s, 13H), 2.87 (t, J = 6.5 Hz, 4H), 2.63 (s, 4H), 2.32 (s, 6H), 1.77 (m, 7H), 1.26 (m, 41H), 0.87 (t, J = 6.9 Hz, 8H). ^{13}C{^{1}H} NMR

(126 MHz, $CDCl_3$) δ 65.2, 61.0, 54.6, 51.6, 51.4, 42.9, 32.0, 29.8, 29.7, 29.6, 29.5, 29.4, 29.3, 26.4, 22.8, 22.7, 14.2. HRMS (ESI+): Found 312.3494, $C_{42}H_{92}N_4[M\text{-}2Br^-]^{2+}$ requires 312.3499.

Preparation of PIP-10, 12A (AAP152)

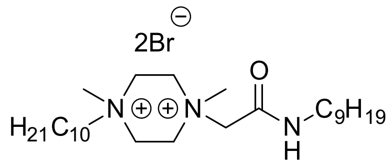

In a 25 mL round-bottom flask equipped with a stir bar, 1-decyl-1,4-dimethylpiperazinium bromide[2] (0.390 g, 1.10 mmol) was dissolved in acetonitrile (10 mL). 2-bromo-*N*-nonyl-acetamide (0.349 g, 1.30 mmol) was added to the reaction flask. The reaction mixture was stirred at 80 °C overnight. Upon completion, the resulting gold solution with white precipitate was removed from heat and allowed to cool to room temperature. Excess solvent was removed from the reaction contents in vacuo. The crude product was vacuum filtered and washed with cyclohexane to afford pure product **PIP-10, 12A** as a white solid (0.299 g, 45%). ^1H NMR (500 MHz, CD_3OD) δ 4.52 (d, 1H), 4.39 (m, 1H), 4.16 – 3.89 (m, 8H), 3.71 (m, 2H), 3.56 (d, J = 10.6 Hz, 3H), 3.35 (dd, J = 8.6 Hz, 3H), 3.23 (t, J = 7.1 Hz, 2H), 1.82 (s, 2H), 1.52 (q, J = 6.9 Hz, 2H), 1.42 (m, 5H), 1.33 – 1.21 (m, 26H), 0.88 (t, J = 6.8 Hz, 6H). $^{13}C\{^1H\}$ NMR (101 MHz, $CDCl_3$) δ 162.5, 54.6, 53.7, 39.4, 31.7, 29.3, 29.3, 29.3, 29.1, 29.1, 28.9, 28.8, 28.7, 26.6, 25.9, 25.9, 22.4, 21.6, 13.2. HRMS (ESI+): Found 219.7253, $C_{27}H_{57}N_3O[M\text{-}2Br^-]^{2+}$ requires 219.7246.

Preparation of mBrPyr-15A (AW39)

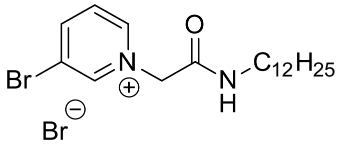

To a small round-bottom flask equipped with a stir bar, 3-bromopyridine (0.200 mL, 2.10 mmol), 2-bromo-*N*-dodecylacetamide[3] (0.912 g, 3.10 mmol), and acetonitrile (5 mL) were added. The reaction mixture was heated to 80 °C for 72 h. The crude product was dried using rotary evaporation to give **mBrPyr-15A** as a white powder (1.13 g, >99%). ^{1}H NMR (400 MHz, $CDCl_3$) δ 9.55 (dt, *J* = 6.1, 1.2 Hz, 1H), 9.34 (t, *J* = 1.6 Hz, 1H), 8.88 (t, *J* = 5.6 Hz, 1H), 8.53 (dt, *J* = 8.4, 1.5 Hz, 1H), 7.95 (dd, *J* = 8.4, 6.1 Hz, 1H), 6.00 (s, 2H), 3.87 (s, 0.3H) 3.31 – 3.18 (m, 3H), 1.56 (dt, J = 15.7, 7.6 Hz, 2H), 1.23 (m, 20H), 0.86 (t, *J* = 6.8 Hz, 4H).; ^{13}C{^{1}H} NMR (126 MHz, DMSO-d_6) δ 166.3, 149.0, 147.8, 146.0, 128.8, 121.7, 62.0, 31.8, 30.1, 29.6, 29.5, 29.5, 29.4, 29.3, 29.2, 26.9, 26.8, 22.6, 14.5. HRMS (ESI+): Found 383.1706, $C_{19}H_{32}BrN_2O[M-Br^-]^+$ requires 383.1693.

Preparation of 16, 12 (AW42)

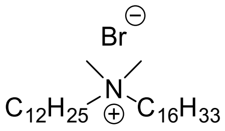

Dodecylhexadecyldimethylammonium bromide (16, 12). In an S_N2 reaction, *N*-*N*-dimethylhexadecylamine (0.400 mL, 1.20 mmol) and 1-bromododecane (0.240 mL, 1.00 mmol) reacted in acetonitrile (5 mL). The product afforded **16, 12** as a pale-yellow powder (0.489 g, 94%). Spectral data were in accordance with the literature.[4]

Preparation of mBrPyr-14A (AC6)

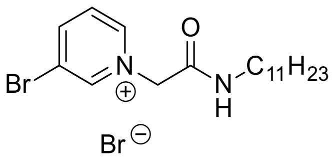

To a small round-bottom flask equipped with a stir bar, 3-bromopyridine (0.20 mL, 2.1 mmol), 2-bromo-*N*-undecylacetamide[3] (0.912 g, 3.10 mmol), and acetonitrile (5 mL) were added. The reaction was stirred at 80 °C for 87 h. After cooling to room temperature, the reaction mixture was concentrated via rotary evaporation. The resulting tan precipitate was triturated with hexanes three times. The product was dried using rotary evaporation to give **mBrPyr-14A** as a white powder (0.792 g, 84%). ^{1}H NMR (400 MHz, $CDCl_3$) δ 9.52 (dt, *J* = 6.1, 1.3 Hz, 1H), 9.34 (t, *J* = 1.6 Hz, 1H), 8.86 (t, *J* = 5.7 Hz, 1H), 8.53 (ddd, *J* = 8.4, 2.0, 1.2 Hz, 1H), 7.96 (dd, *J* = 8.4, 6.1 Hz, 1H), 6.01 (s, 2H), 3.23 (td, *J* = 7.6, 5.7 Hz, 2H), 1.63 – 1.52 (m, 2H), 1.27 (m, 16H), 0.86 (t, *J* = 6.8 Hz, 3H). ^{13}C{^{1}H} NMR (126 MHz, $CDCl_3$) δ 163.1, 148.1, 146.8, 145.1, 128.5, 122.6, 62.6, 40.5, 31.9, 29.7, 29.6, 29.4, 29.3, 29.1, 27.1, 22.7, 14.2. HRMS (ESI+): Found 369.1534, $C_{18}H_{30}BrN_2O[M-Br^-]^+$ requires 369.1536.

Preparation of oX-11, 1 (AW51)

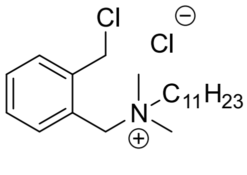

To a small round-bottom flask equipped with a stir bar, α,α′-dichloro-o-xylene (0.175 g, 1.00 mmol), *N,N*-dimethylundecylamine (0.213 g, 1.10 mmol), and acetonitrile (5 mL) were added. The solution was heated for 12 h under reflux. After cooling to room temperature, the rection mixture was concentrated via rotary evaporation. The crude product was purified by flash

column chromatography (silica, 8% methanol in dichloromethane by volume). Fractions containing product were collected and volatiles were removed by rotary evaporation affording the intermediate **AW47** as a solid (0.049 g, 13%). ^{1}H NMR (400 MHz, $CDCl_3$) δ 7.68 (dd, *J* = 7.8, 1.4 Hz, 1H), 7.59 (dd, *J* = 7.9, 1.4 Hz, 1H), 7.50 (td, *J* = 7.6, 1.4 Hz, 1H), 7.41 (td, *J* = 7.6, 1.5 Hz, 1H), 5.30 (d, *J* = 5.7 Hz, 2H), 4.99 (s, 2H), 3.70 – 3.61 (m, 2H), 3.31 (s, 6H), 1.76 (m, 2H), 1.33 (m, 6H), 1.23 (s, 10H), 0.87 (t, *J* = 6.8 Hz, 3H).

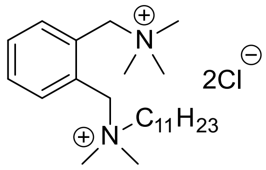

In a small round-bottom flask equipped with a stir bar, the intermediate **AW47** (0.049 g, 0.137 mmol) and trimethylamine 25% solution in ethanol (66.0 µl, 0.197 mmol) were dissolved in acetonitrile (5 mL). The reaction was heated under reflux overnight. The mixture was evaporated in vacuo to afford **oX-11, 1** as a white solid (0.026 g, 46%). ^{1}H NMR (400 MHz, $CDCl_3$) δ 8.23 (d, *J* = 7.3 Hz, 1H), 7.85 (d, *J* = 6.8 Hz, 1H), 7.68 (m, 2H), 5.69 (s, 2H), 5.48 (s, 2H), 3.76 – 3.67 (m, 2H), 3.42 (s, 10H), 3.22 (s, 7H), 1.75 (m, 3H), 1.35 (m, 7H), 1.24 (s, 14H), 0.87 (t, *J* = 6.8 Hz, 5H). $^{13}C\{^1H\}$ NMR (126 MHz, $CDCl_3$) δ 136.4, 135.8, 131.4, 131.2, 130.3, 129.5, 64.2, 64.0, 63.3, 52.5, 49.0, 31.9, 29.8, 29.6, 29.5, 29.4, 26.5, 23.0, 22.7, 14.2. HRMS (ESI+): Found 397.3346, $C_{24}H_{46}N_2Cl[M\text{-}Cl^-]^+$ requires 397.3339.

Preparation of Bn-8E (AW50)

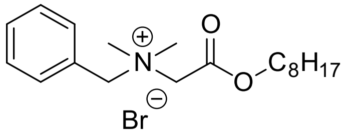

To a small round-bottom flask equipped with a stir bar, 1-octyl bromoacetate [5] (**AW49**) (0.301 g, 1.2 mmol), *N*-*N*-dimethylbenzylamine (0.120 mL, 0.180 g, 1.32 mmol), and acetonitrile (5 mL) were added. The reaction was heated to 80 °C overnight. After cooling to room temperature, the reaction mixture was rotary evaporated, resulting in **Bn-8E** as a pale-yellow solid (0.390 g, 84%). ^{1}H NMR (400 MHz, $CDCl_3$) δ 7.68 – 7.64 (m, 1H), 7.63 – 7.59 (m, 2H), 7.55 – 7.42 (m, 2H), 5.23 (s, 2H), 5.16 (s, 1H), 4.84 (s, 2H), 4.21 – 4.12 (m, 2H), 3.54 (s, 6H), 3.13 (s, 2H), 2.00 (s, 2H), 1.62 (m, 5H), 1.27 (m, 12H), 0.91 – 0.83 (m, 4H). ^{13}C NMR (101 MHz, $CDCl_3$) δ 165.0, 133.5, 130.7, 129.2, 127.5, 67.8, 67.5, 66.9, 60.9, 60.6, 58.7, 54.2, 50.3, 48.2, 44.5, 31.8, 29.2, 28.5, 28.2, 25.8, 25.7, 25.6, 22.6, 14.1. HRMS (ESI+): Found 306.2426, $C_{19}H_{32}NO_2[M-Br^-]^+$ requires 306.2428.

Preparation of ACP-10 (AC17)

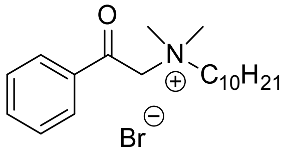

To a small round-bottom flask equipped with a stir bar, phenacyl bromide (0.199 g, 1.00 mmol), *N,N*-dimethyldecylamine (0.350 mL, 0.270 g, 1.50 mmol), and ethanol (5 mL) were added. The reaction was stirred for 4 days at room temperature.[6] The solvent was rotary evaporated to afford **ACP-10** as a pale-yellow crystalline solid (0.107 g, 35%). ^{1}H NMR (400 MHz, $CDCl_3$) δ 8.14 – 8.07 (m, 2H), 7.61 – 7.53 (m, 1H), 7.44 (t, *J* = 7.7 Hz, 2H), 5.85 (s, 2H), 3.89 – 3.81 (m,

2H), 3.63 (s, 6H), 1.68 (td, *J* = 9.6, 5.6 Hz, 2H), 1.34 – 1.19 (m, 6H), 1.20 (s, 8H), 0.83 (t, *J* = 6.8 Hz, 3H). ^{13}C NMR (126 MHz, $CDCl_3$) δ 191.4, 134.9, 134.3, 129.2, 128.6, 66.1, 64.7, 52.1, 31.8, 29.4, 29.3, 29.2, 29.1, 26.2, 23.0, 22.6, 14.1. HRMS (ESI+): Found 304.2643, $C_{20}H_{34}NO[M-Br^-]^+$ requires 304.2635.

Preparation of Pyr-3 (DR-1-051)

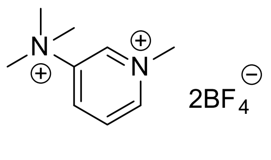

To a 20-mL reaction vial equipped with a stir bar and a pressure relieving septum cap were added *N,N*-dimethylpyridin-3-amine (94.0 µl, 0.100 g, 0.820 mmol), trimethyloxonium tetrafluoroborate (0.278 g, 1.88 mmol), and DCM (1 mL). The reaction vial was stirred at room temperature for 16 h. Upon completion, the reaction mixture was concentrated via rotary evaporation. The resulting tan solid was washed with diethyl ether, vacuum filtered, and dried via rotary evaporation. The pure product **Pyr-3** was isolated as a pale-yellow solid (0.111 g, 42 %). ^1H NMR (400 MHz, DMSO-d_6) δ 9.74 (d, *J* = 2.6 Hz, 1H), 9.21 – 9.12 (m, 2H), 8.40 (dd, *J* = 8.8, 6.1 Hz, 1H), 4.40 (s, 3H), 3.69 (s, 9H). $^{13}C\{^1H\}$ NMR (101 MHz, DMSO-d_6) δ 147.46, 145.65, 141.81, 138.61, 128.78, 57.40, 49.39. HRMS (ESI+): Found 152.1226 $C_9H_{16}N_2[M-2BF_4^-]^{2+}$ requires 152.1303.

Preparation of Allyl,pO12Bn (AC32)

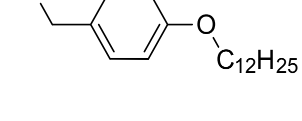

To a 40 mL vial equipped with a stir bar and a pressure relieving septum cap, 4-hydroxymethyl phenol (0.124 g, 1.00 mmol), 1-bromododecane (0.240 mL, 0.250 g, 1.00 mmol), potassium carbonate (0.701 g, 5.10 mmol), and acetone (7 mL) were added. The reaction vial was placed into a reaction pie block preheated on a stir plate to 65 °C for 24 h. The crude product was vacuum filtered, and the filtrate was concentrated via rotary evaporation to afford (4-dodecoxyphenyl)methanol (**AC27**) as a white crystalline solid (0.381 g, >99%).[7] ^{1}H NMR (400 MHz, $CDCl_3$) δ ppm?(d, *J* = 8.6 Hz, 2H), 6.91 – 6.78 (m, 2H), 4.60 (d, *J* = 5.8 Hz, 2H), 3.94 (t, *J* = 6.6 Hz, 2H), 2.16 (s, 1H), 1.88 – 1.70 (m, 2H), 1.56 – 1.38 (m, 2H), 1.31 – 1.20 (m, 18H), 0.91 – 0.83 (m, 3H).

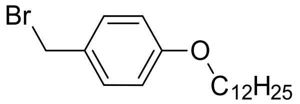

To a 40 mL vial equipped with a stir bar and a pressure relief septum cap, (4-dodecoxyphenyl)methanol (**AC27**) (0.380 g, 1.10 mmol), phosphorus tribromide (0.250 mL, 0.710 g, 2.6 mmol), and dichloromethane (10 mL) were added. The reaction vial was placed into a reaction pie block preheated on a stir plate to 36 °C for 1.5 h. Upon completion, saturated $NaHCO_3$ was added slowly and the reaction mixture was extracted with dichloromethane (15 x 2-5 mL). The combined organic layers were dried with magnesium sulfate, and rotary evaporated to form 4-(dodecyloxy)benzyl bromide (**AC26**) as a tan solid (0.042 g, 11%).[8] ^{1}H NMR (400 MHz, $CDCl_3$) δ 7.33 – 7.25 (m, 2H), 6.88 – 6.80 (m, 2H), 4.49 (s, 2H), 3.93 (t, *J* = 6.5 Hz, 2H), 1.88 – 1.70 (m, 2H), 1.42 (p, *J* = 7.2 Hz, 2H), 1.26 (m, 18H), 0.91 – 0.83 (m, 3H).

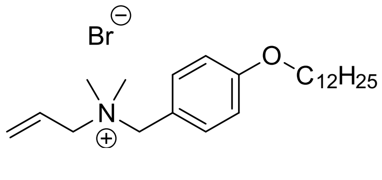

To a 40 mL vial equipped with a stir bar and a pressure relief septum cap, 4-(dodecyloxy)benzyl bromide (**AC26**) (0.042 g, 0.120 mmol), *N, N*-dimethylallylamine (28.0 µL, 0.020 g, 0.240 mmol), and a 1:1 solution of diethyl ether and ethanol (4 mL) were added. The reaction vial was placed into a reaction pie block preheated on a stir plate at 25 °C for 24 h. The product was rotary evaporated for 3 h in a 35 °C water bath to afford **Allyl,pO12Bn** as a pale yellow wax (0.045 g, 87%).[9] ^{1}H NMR (400 MHz, $CDCl_3$) δ 7.57 – 7.51 (m, 2H), 6.93 – 6.85 (m, 2H), 6.01 (ddt, J = 17.1, 10.0, 7.3 Hz, 1H), 5.80 (d, *J* = 16.8 Hz, 1H), 5.71 (d, *J* = 10.0 Hz, 1H), 4.96 (s, 2H), 4.33 (d, *J* = 7.3 Hz, 2H), 3.92 (t, *J* = 6.5 Hz, 2H), 3.18 (s, 6H), 1.78 (m, 2H), 1.49 – 1.37 (m, 2H), 1.34 – 1.16 (m, 22H), 0.85 (t, *J* = 6.7 Hz, 3H). $^{13}C\{^1H\}$ NMR (126 MHz, $CDCl_3$) δ 161.0, 134.7, 130.1, 124.5, 118.8, 115.1, 72.5, 68.3, 67.3, 65.6, 60.1, 58.4, 48.8, 34.1, 32.9, 31.9, 29.7, 29.6, 29.5, 29.4, 29.3, 29.2, 28.8, 28.2, 26.1, 22.7, 18.5, 14.2. HRMS (ESI+): Found 360.3258, $C_{24}H_{42}NO[M-Br^-]^+$ requires 360.3261.

Preparation of pCl-Bn-8 (AC39)

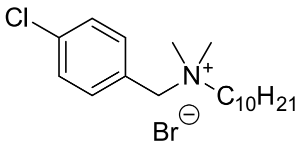

To a 20 mL vial equipped with a stir bar and a pressure relieving septum cap, 4-chlorobenzyl bromide (0.204 g, 0.991 mmol), dimethyldecylamine (0.340 mL, 1.83 mmol), and acetonitrile (5 mL) were added. The reaction mixture was heated to 80 °C while stirring for 6 h. The crude product was dried using rotary evaporation to give a clear pale-yellow oil. The oil was triturated with hexanes twice to give **pCl-Bn-8** as a clear colorless oil (0.351 g, 91%). ^{1}H NMR (400 MHz, $CDCl_3$) δ 7.64 (d, J = 8.5 Hz, 2H), 7.36 (d, *J* = 8.5 Hz, 2H), 5.13 (s, 1H), 3.51 – 3.42 (m, 2H), 3.24 (s, 6H), 1.74 (s, 2H), 1.20 (s, 19H), 0.89 – 0.78 (m, 3H). ^{13}C NMR (101 MHz,

CDCl3) δ 137.2 134.7, 129.5, 126.0, 66.4, 63.9, 49.5, 31.9, 29.4, 29.29, 29.26, 26.4, 23.3, 22.9, 22.7, 14.2. HRMS (ESI+): Found 310.2288, $C_{19}H_{33}BrClN[M-Br^-]^+$ requires 310.2296.

Preparation of mOC6-Bn-8 (AC40)

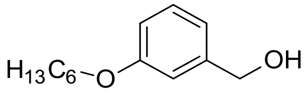

To a 40 mL vial equipped with a stir bar and a pressure relieving septum cap, 3-hydroxymethyl phenol (0.124 g, 1.00 mmol), 1-bromohexane (0.140 mL, 1.00 mmol), potassium carbonate (0.735 g, 5.24 mmol), and acetone (7 mL) were added. The reaction vial was placed into a reaction pie block preheated on a stir plate to 65 °C for 24 h. The crude product was vacuum filtered, and the filtrate was concentrated via rotary evaporation to afford (4-hexylphenyl)methanol (**AC33**) as a pale-yellow oil (0.308 g, >99%).[10] ^{1}H NMR (400 MHz, $CDCl_3$) δ 7.24 (td, *J* = 8.2 Hz, 1H), 6.91 (dd, *J* = 5.5 Hz, 2H), 6.80 (d, *J* = 8.2 Hz, 1H), 4.64 (d, *J* = 5.5 Hz, 2H), 3.95 (t, *J* = 7.4 Hz, 2H), 1.77 (q, *J* = 7.5 Hz, 3H), 1.46 (m, *J* = 7.5 Hz, 2H), 1.23 (m, *J* = 7.0 Hz, 4H), 0.88 (t, *J* = 7.1 Hz, 3H).

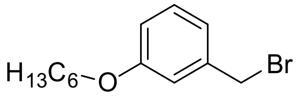

To a 40 mL vial equipped with a stir bar and a pressure relief septum cap, (4-hexyphenyl)methanol (**AC33**) (0.300 g, 1.44 mmol), phosphorus tribromide (0.137 mL, 1.44 mmol), and dichloromethane (10 mL) were added. The reaction vial was placed into a reaction pie block preheated on a stir plate to 36 °C for 1.5 h. Upon completion, saturated $NaHCO_3$ was added slowly and the reaction mixture was extracted with dichloromethane (15 x 2-5 mL). The combined organic layers were dried with magnesium sulfate, and rotary evaporated to form 4-

(hexyloxy)benzyl bromide (**AC36**) as a clear orange oil (0.023 g, 39%).[10] ^{1}H NMR (400 MHz, $CDCl_3$) δ 7.27 – 7.18 (ddd, 1H), 6.98 – 6.89 (m, 2H), 6.82 (dd, *J* = 7.8, 3.1 Hz, 1H), 4.45 (s, 2H), 3.94 (t, *J* = 6.5 Hz, 2H), 1.92 – 1.71 (m, 2H), 1.50 – 1.40 (m, 2H), 1.34 (d, *J* = 7.3 Hz, 4H), 0.97 – 0.84 (m, 3H).

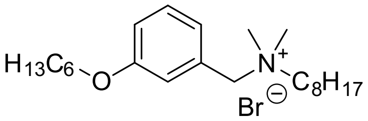

To a 40 mL vial equipped with a stir bar and a pressure relief septum cap, 4-(hexyloxy)benzyl bromide (**AC36**) (0.023 g, 0.083 mmol), dimethyloctylamine (17.0 µL, 0.080 mmol), and acetonitrile (3 mL) were added. The reaction vial was placed into a reaction pie block preheated on a stir plate 80°C for 24 h. The product was rotary evaporated to afford **mOC6-Bn-8** as a clear yellow oil (0.026 g, 73%). ^{1}H NMR (400 MHz, $CDCl_3$) δ 7.42 – 7.28 (m, 1H), 7.20 – 7.13 (m, 2H), 6.98 (ddd, *J* = 8.3, 2.4, 1.0 Hz, 1H), 4.97 (s, 2H), 3.96 (t, *J* = 6.4 Hz, 2H), 3.57 – 3.45 (m, 2H), 3.30 (s, 6H), 1.77 (s, 4H), 1.63 (s, 3H), 1.50 – 1.38 (m, 2H), 1.36 – 1.26 (m, 16H), 0.87 (dt, *J* = 11.5, 7.0 Hz, 6H). ^{13}C (126 MHz, $CDCl_3$) δ 159.6 130.3, 128.5, 125.2, 119.2, 116.8, 68.5, 67.4, 63.9, 49.9, 31.69, 31.67, 29.28, 29.24, 29.10, 26.4, 25.8, 23.0, 22.6, 14.1. HRMS (ESI+): Found 348.3269, $C_{23}H_{42}BrNO[M-Br^-]^+$ requires 348.3261.

Preparation of Bn-pOC9-Bn (AC41)

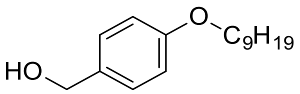

To a 40 mL vial equipped with a stir bar and a pressure relieving septum cap, 4-hydroxymethyl phenol (0.626 g, 5.04 mmol), 1-bromononane (0.950 mL, 5.00 mmol), potassium carbonate (2.10 g, 14.9 mmol), and acetone (15 mL) were added. The reaction vial was placed into

a reaction pie block preheated on a stir plate to 65 °C for 24 h. The crude product was vacuum filtered, and the filtrate was recrystallized with hexanes and concentrated via rotary evaporation to afford (4-nonylphenyl)methanol (**AC35**) as a tan flaky solid (0.806 g, 64%).[11] ^{1}H NMR (400 MHz, $CDCl_3$) δ 7.31 – 7.22 (m, 3H), 6.91 – 6.83 (m, 2H), 4.60 (d, *J* = 5.6 Hz, 2H), 3.94 (t, *J* = 6.6 Hz, 2H), 1.76 (dt, *J* = 14.7, 6.7 Hz, 2H), 1.50 – 1.38 (m, 2H), 1.38 – 1.23 (m, 10H), 0.91 – 0.83 (m, 3H).

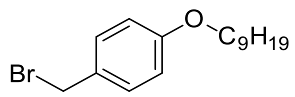

To a 40 mL vial equipped with a stir bar and a pressure relief septum cap, (4-nonylphenyl)methanol (**AC35**) (0.500 g, 1.99 mmol), phosphorus tribromide (0.190 mL, 1.99 mmol), and dichloromethane (10 mL) were added. The reaction vial was placed into a reaction pie block preheated on a stir plate to 36 °C for 1.5 h. Upon completion, saturated $NaHCO_3$ was added slowly and the reaction mixture was extracted with dichloromethane (10 x 5 mL). The combined organic layers were dried with magnesium sulfate, and rotary evaporated to form 4-(nonyloxy)benzyl bromide (**AC38**) as a clear yellow oil (0.158 g, 43%).[11] ^{1}H NMR (500 MHz, $CDCl_3$) δ 7.32 – 7.18 (m, 2H), 6.95 – 6.80 (m, 2H), 4.49 (s, 2H), 3.93 (t, *J* = 6.6 Hz, 2H), 1.76 (s, 2H), 1.48 – 1.39 (m, 2H), 1.39 – 1.18 (m, 10H), 0.93 – 0.77 (m, 3H).

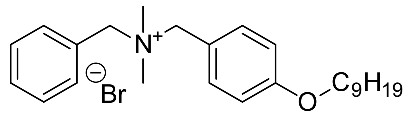

To a 40 mL vial equipped with a stir bar and a pressure relief septum cap, 4-(nonyloxy)benzyl bromide (**AC38**) (0.0790 g, 0.252 mmol), dimethylbenzylamine (37.0 µL, 0.252 mmol), and acetonitrile (3 mL) were added. The reaction vial was placed into a reaction pie block

preheated on a stir plate 80 °C for 24 h. The product was rotary evaporated to afford **Bn-pOC9-Bn** as a clear yellow oil (0.076 g, 69%). ^{1}H NMR (400 MHz, $CDCl_3$) δ 7.69 – 7.59 (m, 2H), 7.58 – 7.44 (m, 2H), 7.46 – 7.33 (m, 3H), 6.95 – 6.78 (m, 2H), 5.08 (d, *J* = 5.4 Hz, 4H), 3.91 (t, *J* = 6.5 Hz, 2H), 3.07 (s, 6H), 1.85 – 1.65 (m, 4H), 1.52 – 1.38 (m, 2H), 1.36 – 1.13 (m, 16H), 1.01 – 0.76 (t, 3H). ^{13}C NMR (126 MHz, $CDCl_3$) δ 160.94, 134.86, 133.46, 130.62, 129.19, 127.60, 118.96, 114.99, 68.24, 67.53, 67.18, 47.92, 31.93, 29.59, 29.46, 29.33, 29.21, 26.09, 22.59, 14.19. HRMS (ESI+): Found 368.2969, $C_{25}H_{38}BrO[M\text{-}Br^-]^+$ requires 368.2948.

Preparation of 8(3)0(3)8 (AC42)

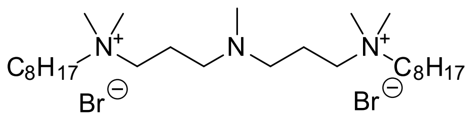

To a 20 mL vial equipped with a stir bar and a pressure relieving septum cap, 2,6,10-trimethyl-2,6,10-triazaundecane (0.510 mL, 2.20 mmol), 1-bromooctane (0.770 mL, 4.50 mmol), and acetonitrile (1 mL) were added. The reaction mixture was heated to 80 °C for 19 h while stirring. The crude product was dried using rotary evaporation to give a clear orange-yellow oil. The oil was triturated with hexanes and dried in the vacuum desiccator to give **8(3)0(3)8** as a thick clear orange-yellow oil (0.699 g, 47%). ^{1}H NMR (500 MHz, $CDCl_3$) δ 3.95 – 3.77 (m, 4H), 3.45 – 3.36 (m, 4H), 3.30 (s, 12H), 2.91 (d, *J* = 8.0 Hz, 4H), 2.52 (t, *J* = 6.2 Hz, 3H), 2.19 (s, 2H), 1.97 – 1.83 (m, 3H), 1.68 (dq, *J* = 12.5, 5.9 Hz, 4H), 1.39 – 1.11 (m, 20H), 0.82 (t, *J* = 6.9 Hz, 6H). ^{13}C NMR (126 MHz, CDCl3) δ 64.2 63.3, 53.9, 51.1, 42.0, 31.7, 29.2, 29.1, 26.4, 22.95, 22.90, 20.8, 14.2. HRMS (ESI+): Found 213.7434, $C_{27}H_{61}Br_2N_3[M\text{-}2Br^-]^+$ requires 427.4855.

Preparation of oX-8,8 (AC44)

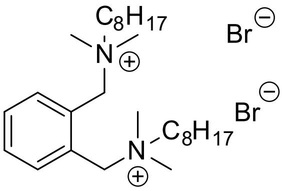

To a 20 mL vial equipped with a stir bar and a pressure relieving septum cap, 1,2-bis(bromomethyl)-benzene (0.525 g, 1.99 mmol), dimethyloctylamine (0.820 mL, 4.01 mmol), and acetonitrile (5 mL) were added. The reaction mixture was heated to 80 °C for 24 h while stirring. The product was dried using rotary evaporation and two days of vacuum desiccation to afford **oX-8,8** as an orange solid (1.14 g, 99%). ^{1}H NMR (500 MHz, $CDCl_3$) δ 8.08 (dd, *J* = 5.8, 3.5 Hz, 2H), 7.65 (dd, *J* = 5.8, 3.4 Hz, 2H), 5.48 (s, 4H), 3.93 – 3.70 (m, 4H), 3.25 (s, 12H), 1.71 (s, 4H), 1.40 – 1.13 (m, 20H), 0.91 – 0.75 (m, 6H). ^{13}C NMR (126 MHz, $CDCl_3$) δ 136.4, 131.2, 129.6, 64.0, 63.5, 48.5, 31.7, 29.3, 28.9, 26.3, 22.9, 22.5, 22.4, 14.0. HRMS (ESI+): Found 209.2144, $C_{28}H_{54}Br_2N_2[M\text{-}2Br^-]^+$ requires 418.4276.

Preparation of oOC9-Bn-10 (AC46)

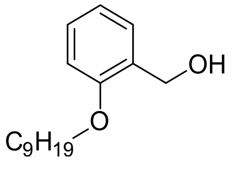

To a 40 mL vial equipped with a stir bar and a pressure relieving septum cap, 2-hydroxymethyl phenol (0.626 g, 5.04 mmol), 1-bromononane (0.950 mL, 5.00 mmol), potassium carbonate (2.16 g, 15.4 mmol), and acetone (15 mL) were added. The reaction vial was placed into a reaction pie block preheated on a stir plate to 65 °C for 24 h. The product was concentrated via

rotary evaporation to afford (2-nonylphenyl)methanol (**AC43**) as a clear orange oil (0.952 g, 75%). ^{1}H NMR (400 MHz, $CDCl_3$) δ 7.35 – 7.19 (m, 2H), 6.93 (td, *J* = 7.4, 1.1 Hz, 1H), 6.86 (dd, *J* = 8.2, 1.1 Hz, 1H), 4.70 (d, *J* = 11.8 Hz, 2H), 3.99 (t, *J* = 6.5 Hz, 2H), 1.91 – 1.73 (m, 2H), 1.53 – 1.43 (m, 2H), 1.40 – 1.19 (m, 10H), 1.01 – 0.83 (m, 3H).

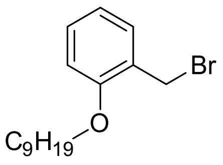

To a 40 mL vial equipped with a stir bar and a pressure relief septum cap, (2-nonylphenyl)methanol (**AC43**) (0.888 g, 3.55 mmol), phosphorus tribromide (0.36 mL, 3.80 mmol), and dichloromethane (5 mL) were added. The reaction vial was placed in an ice bath on a stir plate for 2 h under an Argon balloon. Upon completion, the reaction was quenched with 5 mL of H_2O, saturated $NaHCO_3$ was added slowly, and the reaction mixture was extracted with dichloromethane (3 x 10 mL). The combined organic layers were dried with sodium sulfate, and rotary evaporated to form 2-(nonyloxy)benzyl bromide (**AC45**) as a cloudy yellow oil (1.11 g, 63%). ^{1}H NMR (400 MHz, $CDCl_3$) δ 7.42 – 7.19 (m, 2H), 7.04 – 6.81 (m, 2H), 4.59 (s, 2H), 4.04 (t, *J* = 6.4 Hz, 2H), 1.95 – 1.74 (m, 2H), 1.62 – 1.47 (m, 2H), 1.43 – 1.13 (m, 10H), 1.01 – 0.83 (m, 3H).

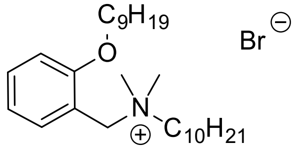

To a 40 mL vial equipped with a stir bar and a pressure relief septum cap, 2-(nonyloxy)benzyl bromide (**AC45**) (0.350 g, 1.12 mmol), dimethyldecylamine (0.261 mL, 1.12 mmol), and acetonitrile (4 mL) were added. The reaction vial was placed into a reaction pie block

preheated on a stir plate to 80 °C for 24 h. The crude product was rotary evaporated to give a clear yellow oil. The crude product was triturated with hexanes and dried in the vacuum desiccator to afford **oOC9-Bn-10** as a clear yellow oil (0.361 g, 64%). ^{1}H NMR (500 MHz, $CDCl_3$) δ 7.73 (dd, *J* = 7.6, 1.7 Hz, 1H), 7.41 (ddd, *J* = 8.8, 7.4, 1.7 Hz, 1H), 7.02 (td, *J* = 7.5, 1.0 Hz, 1H), 6.93 (dd, *J* = 8.4, 1.0 Hz, 1H), 4.79 (s, 2H), 3.99 (t, *J* = 6.8 Hz, 2H), 3.62 – 3.50 (m, 2H), 3.23 (s, 6H), 1.78 (tt, *J* = 14.3, 7.1 Hz, 5H), 1.48 – 1.06 (m, 43H), 0.94 – 0.77 (m, 6H). ^{13}C NMR (126 MHz, $CDCl_3$) δ 158.19, 135.70, 132.71, 121.26, 115.68, 112.21, 68.73, 64.94, 62.16, 50.03, 31.93, 29.64, 29.58, 29.41, 29.38, 29.30, 29.23, 26.52, 26.20, 23.20, 22.69, 14.14. HRMS (ESI+): Found 418.4042, $C_{28}H_{52}BrNO$ $[M-Br^-]^+$ requires 418.4043.

Preparation of 2,3methoxy-Bn10 (AC52)

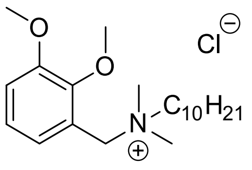

To a 20 mL vial equipped with a stir bar and a pressure relieving septum cap, 2,3-dimethoxybenzyl chloride (0.187 g, 1.00 mmol), dimethyldecylamine (0.234 mL, 1.03 mmol), and acetonitrile (3 mL) were added. The reaction mixture was heated to 80 °C for 24 h while stirring. The product was dried using rotary evaporation and three days of vacuum desiccation to afford **2,3methoxy-Bn10** as a thick clear yellow oil (0.419 g, >99%). ^{1}H NMR (400 MHz, $CDCl_3$) δ 7.29 (dd, *J* = 7.7, 1.5 Hz, 1H), 7.07 (t, *J* = 8.0 Hz, 1H), 6.98 (dd, *J* = 8.3, 1.5 Hz, 1H), 4.77 (s, 2H), 3.82 (d, *J* = 1.5 Hz, 6H), 3.47 – 3.27 (m, 2H), 3.16 (s, 6H), 1.73 (ddt, *J* = 16.1, 11.7, 5.6 Hz, 2H), 1.23 (d, *J* = 35.5 Hz, 15H), 0.80 (t, *J* = 6.7 Hz, 3H). ^{13}C NMR (126 MHz, $CDCl_3$) δ 152.59, 148.95, 126.44, 124.49, 121.03, 115.07, 64.50, 62.12, 61.25, 55.85, 50.01, 31.80, 29.38, 29.30, 29.20,

26.39, 23.17, 22.48, 13.95. HRMS (ESI+): Found 336.2898, $C_{21}H_{38}ON_2Cl$ [M-Br$^-$]$^+$ requires 336.2897.

Preparation of 3,4methoxy-Bn10 (AC60)

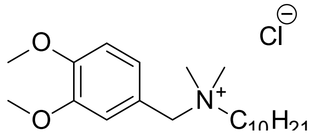

To a 20 mL vial equipped with a stir bar and a pressure relieving septum cap, 3,4-dimethoxybenzyl chloride (0.188 g, 1.01 mmol), dimethyldecylamine (0.230 mL, 1.00 mmol), and acetonitrile (3 mL) were added. The reaction mixture was heated to 80 °C for 48 h while stirring. The crude product was dried using rotary evaporation to give a clear orange oil. The product was triturated in 3:1 hexanes ethyl ether and dried via vacuum desiccation to afford **2,3methoxy-Bn10** as a light tan solid (0.274 g, 73%). ^{1}H NMR (500 MHz, $CDCl_3$) δ 7.29 (d, *J* = 2.1 Hz, 1H), 7.08 (dd, *J* = 8.2, 2.0 Hz, 1H), 6.87 – 6.73 (m, 1H), 4.91 (s, 2H), 4.00 – 3.70 (m, 7H), 3.49 – 3.32 (m, 2H), 3.21 (s, 6H), 1.72 (tq, *J* = 11.3, 5.3 Hz, 2H), 1.21 (d, *J* = 42.1 Hz, 17H), 0.80 (t, *J* = 6.9 Hz, 4H). ^{13}C NMR (126 MHz, CDCl3) δ 150.77, 149.2, 126.09, 119.76, 116.14, 111.06, 67.61, 63.44, 56.53, 55.92, 49.54, 31.85, 29.43, 29.25, 26.43, 22.95, 22.67, 14.14. HRMS (ESI+): Found 336.2897, $C_{21}H_{38}ON_2Cl$ [M-Br$^-$]$^+$ requires 336.2893.

Preparation of DPE-11E,11E (AC59)

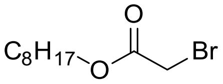

To a small round-bottom flask equipped with a stir bar and secured under an argon balloon, 1-octanol (2.05 mL, 13.0 mmol) and dichloromethane (10 mL) were added. In a vial, bromoacetyl

bromide (1.13 mL, 13.0 mmol) and dichloromethane (2.50 mL) were added. The solution in the vial was added dropwise over 20 minutes to the round bottom flask. The reaction was stirred at room temperature for 24 h. Upon completion, saturated $NaHCO_3$ was added slowly, and the reaction mixture was extracted with dichloromethane (3 x 7 mL). The combined organic layers were dried with sodium sulfate, and rotary evaporated to form **AC56** as a clear colorless oil (0.826 g, 26%). ^1H NMR (500 MHz, $CDCl_3$) δ 4.11 (t, J = 6.7 Hz, 2H), 3.78 (s, 2H), 1.61 (dq, J = 8.1, 6.7 Hz, 2H), 1.39 – 1.15 (m, 10H), 0.91 – 0.77 (m, 3H).

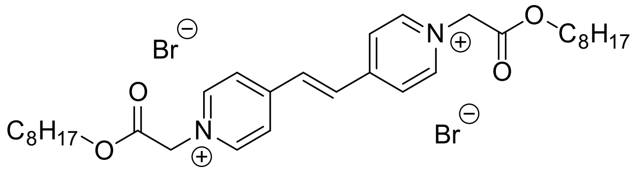

To a 20 mL vial equipped with a stir bar and a pressure relieving septum cap, (E)-1,2-di(pyridine-4-yl)ethene (0.146 g, 0.802 mmol), AC56 (0.4014 mL, 1.59 mmol), and acetonitrile (5 mL) were added. The reaction mixture was heated to 80 °C for 24 h while stirring. The crude product was dried using rotary evaporation to give an orange-yellow solid. The product was recrystallized in methanol and ethyl ether and dried via rotary evaporation to afford **DPE-11E,11E** as a yellow powder (0.025 g, 5%). ^1H NMR (500 MHz, CD_3OD) δ 9.09 – 8.91 (m, 4H), 8.48 – 8.38 (m, 4H), 8.15 (s, 2H), 5.58 (s, 4H), 4.26 (t, J = 6.7 Hz, 4H), 1.76 – 1.56 (m, 4H), 1.48 – 1.17 (m, 21H), 0.94 – 0.79 (m, 6H). ^{13}C NMR (126 MHz, CD_3OD) δ 166.04, 152.33, 146.47, 134.56, 125.57, 66.89, 60.12, 31.63, 28.99, 28.20, 25.56, 22.38, 13.09. HRMS (ESI+): Found 524.3601, $C_{32}H_{48}Br_2N_2O_4$ $[M\text{-}Br^-]^+$ requires 524.3603.

Preparation of EtBn-pOC9Bn (AC73)

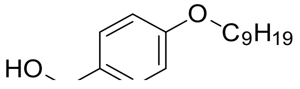

To a 40 mL vial equipped with a stir bar and a pressure relieving septum cap, 4-hydroxybenzyl alcohol (0.622 g, 5.01 mmol), 1-bromononane (0.950 mL, 5.00 mmol), potassium carbonate (2.20 g, 15.7 mmol), and acetone (15 mL) were added. The reaction vial was placed into a reaction pie block preheated on a stir plate to 65 °C for 24 h. The crude product was vacuum filtered, and the filtrate was recrystallized with hexanes and concentrated via rotary evaporation to afford (4-nonylphenyl)methanol (**AC70**) as a tan flaky solid (0.763 g, 61%).[11] ^{1}H NMR (500 MHz, $CDCl_3$) δ 7.33 – 7.18 (m, 2H), 6.95 – 6.86 (m, 2H), 4.60 (d, *J* = 5.6 Hz, 2H), 3.94 (t, *J* = 6.6 Hz, 2H), 1.76 (dt, *J* = 14.8, 6.7 Hz, 2H), 1.55 (s, 1H), 1.51 (t, *J* = 5.8 Hz, 1H), 1.43 (ddd, *J* = 12.2, 8.3, 6.1 Hz, 2H), 1.37 – 1.19 (m, 10H), 0.87 (t, *J* = 6.8 Hz, 3H).

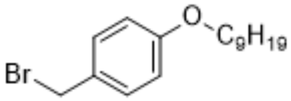

To a 40 mL vial equipped with a stir bar and a pressure relief septum cap, (4-nonylphenyl)methanol (**AC70**) (0.763 g, 3.05 mmol), phosphorus tribromide (0.290 mL, 3.00 mmol), and dichloromethane (13 mL) were added. The reaction vial was placed in an ice bath on a stir plate for 1.5 h under an argon balloon. Upon completion, the reaction was quenched with 5 mL of H_2O, saturated $NaHCO_3$ was added slowly, and the reaction mixture was extracted with dichloromethane (3 x 10 mL). The combined organic layers were dried with sodium sulfate, and rotary evaporated to form 4-(nonyloxy)benzyl bromide (**AC72**) as a pale-yellow oil (0.9434 g,

47%).[11] ^{1}H NMR (500 MHz, $CDCl_3$) δ 7.36 – 7.27 (m, 2H), 6.95 – 6.75 (m, 2H), 4.49 (s, 2H), 3.94 (t, J = 6.5 Hz, 2H), 1.76 (dq, J = 8.0, 6.6 Hz, 2H), 1.51 – 1.39 (m, 2H), 1.35 – 1.17 (m, 10H), 0.94 – 0.78 (m, 3H).

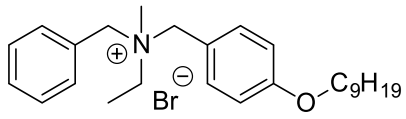

To a 40 mL vial equipped with a stir bar and a pressure relief septum cap, 4-(nonyloxy)benzyl bromide (**AC72**) (0.209 g, 0.638 mmol), *N*-benzyl-*N*-methyl-ethanamine (0.103 mL, 0.640 mmol), and acetonitrile (3 mL) were added. The reaction vial was placed into a reaction pie block preheated on a stir plate 80 °C for 24 h. The crude product was rotary evaporated to give a cloudy white oil. The product was triturated in hexanes and dried via rotary evaporation to afford **EtBn-pOC9Bn** as a tacky white solid (0.201 g, 65%). ^{1}H NMR (500 MHz, $CDCl_3$) δ 7.63 – 7.58 (m, 2H), 7.55 – 7.49 (m, 2H), 7.38 (ddd, J = 14.3, 7.8, 6.1 Hz, 3H), 6.90 – 6.80 (m, 2H), 5.04 – 4.80 (m, 4H), 3.89 (t, J = 6.5 Hz, 2H), 3.35 (qd, J = 7.4, 3.6 Hz, 2H), 3.00 (s, 3H), 2.08 (s, 1H), 1.73 (p, J = 6.7 Hz, 2H), 1.51 (t, J = 7.2 Hz, 3H), 1.45 – 1.38 (m, 2H), 1.35 – 1.15 (m, 11H), 0.94 – 0.79 (m, 3H). ^{13}C NMR (101 MHz, $CDCl_3$) δ 160.9, 134.70, 133.31, 130.59, 129.25, 127.44, 118.69, 115.04, 68.22, 64.29, 64.14, 54.75, 45.69, 31.90, 29.56, 29.43, 29.30, 29.18, 26.06, 22.71, 14.18, 9.11. HRMS (ESI+): Found 382.3107, $C_{26}H_{40}NBrO$ $[M-Br^{-}]^{+}$ requires 382.3104.

Preparation of Paraquat-11,3methoxyBn (AC80)

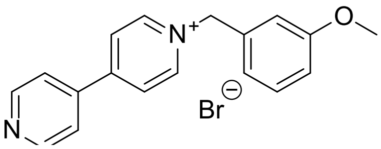

To a 20 mL vial equipped with a stir bar and a pressure relieving septum cap, 4-4'-dipyridyl (0.319 g, 2.04 mmol), 3-methoxybenzyl bromide (0.280 mL, 1.96 mmol), and acetonitrile (7 mL) were added. The reaction mixture was heated to 80 °C for 24 h while stirring. The crude product was dried using rotary evaporation to give a yellow-orange oil. The product was recrystallized in methanol and ethyl ether three times and dried via rotary evaporation to afford **AC58** as a dark orange oil (0.211 g, 29%). ^{1}H NMR (500 MHz, CD_3OD) δ 9.37 – 9.26 (m, 1H), 9.24 – 9.12 (m, 1H), 8.85 – 8.78 (m, 1H), 8.72 – 8.61 (m, 1H), 8.57 – 8.43 (m, 1H), 8.03 – 7.90 (m, 1H), 7.87 – 7.77 (m, 1H), 7.37 (ddd, *J* = 8.3, 7.6, 1.9 Hz, 1H), 7.23 – 7.07 (m, 2H), 7.01 (ddd, *J* = 8.4, 2.6, 0.9 Hz, 1H), 5.89 (d, *J* = 29.2 Hz, 2H), 3.80 (d, *J* = 2.3 Hz, 3H).

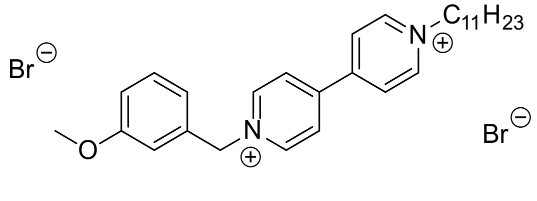

To a 20 mL vial equipped with a stir bar and a pressure relieving septum cap, AC58 (0.100 g, 0.280 mmol), 1-bromoundecane (0.0630 mL, 0.280 mmol), and acetonitrile (1 mL) were added. The reaction mixture was heated to 80°C while stirring for 72 h. The crude product was dried using rotary evaporation to give a yellow-orange oil. The product was recrystallized in methanol and ethyl ether and dried via rotary evaporation to afford **Paraquat-11,3methoxyBn** as a yellow solid (0.016 g, 11%).^{1}H NMR (400 MHz, CD_3OD) δ 9.37 – 9.29 (m, 2H), 9.28 – 9.23 (m, 1H), 8.66 (td, *J* = 6.9, 2.1 Hz, 3H), 7.47 – 7.35 (m, 1H), 7.22 – 7.04 (m, 2H), 7.02 (dtd, *J* = 8.4, 2.7, 0.9 Hz, 1H), 5.91 (d, *J* = 3.9 Hz, 2H), 4.72 (t, *J* = 7.6 Hz, 1H), 3.80 (d, *J* = 2.8 Hz, 3H), 2.05 (q, *J* = 7.3 Hz, 1H), 1.49 – 1.37 (m, 3H), 1.27 (s, 8H), 0.98 – 0.79 (m, 2H). ^{13}C NMR (101 MHz, CD_3OD) δ 160.7, 150.39, 149.85, 145.68, 134.20, 130.63, 127.01, 121.05, 115.37, 114.66, 64.47, 61.98, 54.69,

31.73, 31.27, 29.37, 28.84, 25.91, 22.40, 13.11. HRMS (ESI+): Found 432.3122, $C_{29}H_{40}Br_2N_2O$ $[M-2Br^-]^+$ requires 432.213.

Preparation of DPA-8,mOC6Bn (AC81)

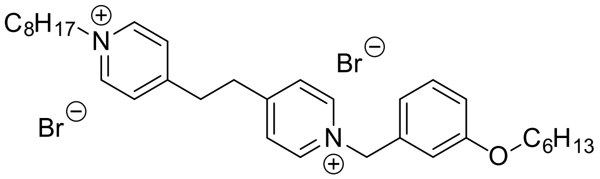

To a 20 mL vial equipped with a stir bar and a pressure relieving septum cap, MAF-017 (0.034 g, 0.075 mmol), 1-bromooctane (0.013 mL, 0.075 mmol), and acetonitrile (1 mL) were added. The reaction mixture was heated to 80 °C for 72 h while stirring. The product was dried using rotary evaporation to afford **DPA-8,mOC6Bn** as a light orange-pink powder (0.048 g, 71%). ^{1}H NMR (400 MHz, CD_3OD) δ 9.02 – 8.84 (m, 1H), 8.06 (t, *J* = 6.4 Hz, 1H), 7.16 – 6.90 (m, 1H), 3.97 (t, *J* = 6.4 Hz, 1H), 1.83 – 1.67 (m, 1H), 1.45 (h, *J* = 7.0 Hz, 1H), 1.38 – 1.19 (m, 3H), 0.89 (dt, *J* = 8.8, 6.9 Hz, 1H). ^{13}C NMR (101 MHz, CD_3OD) δ 161.2, 160.60, 160.12, 144.13, 134.60, 130.52, 128.08, 120.60, 115.49, 114.99, 67.92, 63.72, 61.12, 34.25, 34.15, 31.54, 31.42, 31.09, 28.97, 28.86, 28.76, 25.87, 25.52, 22.33, 13.05. HRMS (ESI+): Found 488.3733, $C_{33}H_{48}Br_2N_2O$ $[M-2Br^-]^+$ requires 488.7589.

Preparation of Paraquat-4,4 (AC87)

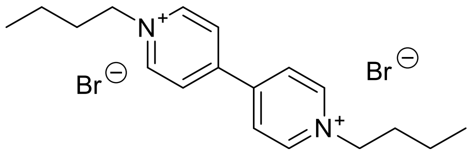

To a 20 mL vial equipped with a stir bar and a pressure relieving septum cap, 4-4'-dipyridyl (0.155 g, 0.999 mmol), 1-bromobutane (0.540 mL, 4.90 mmol), and acetonitrile (3 mL) were added. The reaction mixture was heated to 80 °C for 72 h while stirring. The crude product was dried using rotary evaporation to give a dark yellow solid. The product was recrystallized in methanol and ethyl ether and dried via vacuum desiccation to afford **Paraquat-4,4** as a yellow powder (0.1739 g, 41%). ^{1}H NMR (500 MHz, CD_3OD) δ 9.37 – 9.19 (m, 4H), 8.69 (d, *J* = 6.2 Hz, 4H), 4.82 (s, 1H), 4.76 (t, *J* = 7.6 Hz, 4H), 3.33 (s, 1H), 3.28 (p, *J* = 1.7 Hz, 1H), 2.07 (tt, *J* = 9.3, 6.8 Hz, 4H), 1.57 – 1.39 (m, 4H), 1.02 (t, *J* = 7.4 Hz, 6H). ^{13}C NMR (126 MHz, CD_3OD) δ 149.9, 145.79, 127.27, 61.75, 33.15, 19.16, 12.50. HRMS (ESI+): Found 270.2077, $C_{18}H_{26}Br_2N_2$ [M-2Br$^-$]$^+$ requires 270.4189.

Preparation of 3,5methoxy-Bn-10 (AC89)

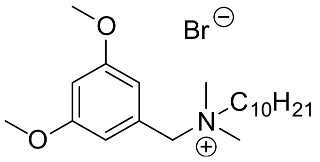

To a 20 mL vial equipped with a stir bar and a pressure relieving septum cap, 3,5-dimethoxybenzyl bromide (0.231 g, 0.999 mmol), dimethyldecylamine (0.240 mL, 1.02 mmol), and acetonitrile (3 mL) were added. The reaction mixture was heated to 80 °C for 24 h while stirring. The crude product was dried using rotary evaporation to give a clear orange oil. The product was triturated in hexanes twice and dried via rotary evaporation to afford **3,5methoxy-Bn-10** as a light tan solid (0.366 g, 88%). ^{1}H NMR (500 MHz, $CDCl_3$) δ 6.79 (d, *J* = 2.3 Hz, 2H), 6.45 (t, *J* = 2.2 Hz, 1H), 4.91 (s, 2H), 3.75 (s, 6H), 3.55 – 3.40 (m, 2H), 3.26 (s, 6H), 1.74 (s, 2H), 1.38 – 1.11 (m, 14H), 0.82 (t, *J* = 7.0 Hz, 3H). ^{13}C NMR (126 MHz, $CDCl_3$) δ 161.1, 129.30,

111.18, 102.31, 67.26, 64.10, 55.81, 50.01, 31.83, 29.42, 29.29, 29.24, 26.35, 23.01, 22.79, 14.12. HRMS (ESI+): Found 336.2896, $C_{21}H_{38}BrNO_2$ $[M-Br^-]^+$ requires 336.2897.

Preparation of Pyr-mOC9 (AC92)

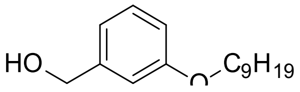

To a 40 mL vial equipped with a stir bar and a pressure relieving septum cap, 3-hydroxybenzyl alcohol (0.622 g, 5.01 mmol), 1-bromononane (0.710 mL, 4.98 mmol), potassium carbonate (2.10 g, 14.9 mmol), and acetone (15 mL) were added. The reaction vial was placed into a reaction pie block preheated on a stir plate to 65 °C for 24 h. The crude product was vacuum filtered, and the filtrate was recrystallized with hexanes and concentrated via rotary evaporation to afford (3-nonylphenyl)methanol (**AC88**) as a white powder (0.137 g, 11%). ^1H NMR (500 MHz, CD_3OD) δ 7.29 – 7.14 (m, 1H), 6.96 – 6.82 (m, 2H), 6.76 (ddd, *J* = 8.2, 2.6, 1.0 Hz, 1H), 4.54 (d, *J* = 0.7 Hz, 2H), 3.93 (t, *J* = 6.5 Hz, 2H), 1.73 (ddt, *J* = 9.0, 7.9, 6.4 Hz, 2H), 1.51 – 1.38 (m, 2H), 1.39 – 1.21 (m, 10H), 0.96 – 0.79 (m, 3H).

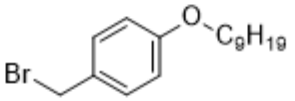

To a 40 mL vial equipped with a stir bar and a pressure relief septum cap, (3-nonylphenyl)methanol (**AC88**) (0.137 g, 0.548 mmol), phosphorus tribromide (0.052 mL, 0.550 mmol), and dichloromethane (5 mL) were added. The reaction vial was placed in an ice bath on a stirrer for 2 h under an argon balloon. Upon completion, the reaction was quenched with 5 mL

dH_2O, saturated $NaHCO_3$ was added slowly, and the reaction mixture was extracted with dichloromethane (3 x 10 mL). The combined organic layers were dried with sodium sulfate, and rotary evaporated to form 3-(nonyloxy)benzyl bromide (**AC90**) as a clear orange oil (0.079 g, 46%). ^1H NMR (500 MHz, $CDCl_3$) δ 7.22 (d, J = 7.9 Hz, 1H), 7.02 – 6.90 (m, 2H), 6.86 – 6.68 (m, 1H), 4.45 (s, 2H), 3.95 (t, J = 6.5 Hz, 2H), 1.91 – 1.67 (m, 2H), 1.57 – 1.39 (m, 2H), 1.39 – 1.12 (m, 10H), 0.89 (t, J = 6.8 Hz, 3H).

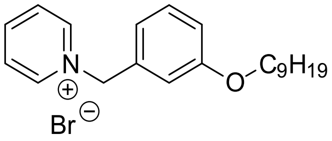

To a 20 mL vial equipped with a stir bar and a pressure relief septum cap, 3-(nonyloxy)benzyl bromide (**AC90**) (0.040 g, 0.127 mmol), pyridine (0.00960 mL, 0.127 mmol), and dichloromethane (2 mL) were added. The reaction vial was placed into a reaction pie block preheated on a stir plate at 35 °C for 24 h. The crude product was rotary evaporated to give a cloudy yellow oil. The product was recrystallized with methanol and ethyl ether and dried via rotary evaporation to afford **Pyr-mOC9** as a light tan solid (0.013 g, 27%). ^1H NMR (400 MHz, $CDCl_3$) δ 9.49 (d, J = 6.0 Hz, 2H), 8.40 (t, J = 7.7 Hz, 1H), 8.07 – 7.90 (m, 2H), 7.25 (s, 2H), 7.19 – 7.04 (m, 2H), 6.99 – 6.78 (m, 1H), 6.24 (s, 2H), 3.94 (t, J = 6.5 Hz, 2H), 1.91 (s, 3H), 1.80 – 1.65 (m, 2H), 1.41 (td, J = 9.2, 4.7 Hz, 2H), 1.37 – 1.13 (m, 10H), 0.92 – 0.80 (m, 3H). ^{13}C NMR (126 MHz, $CDCl_3$) δ 160.1, 145.13, 134.02, 130.84, 128.23, 121.55, 116.39, 115.47, 68.50, 64.43, 31.95, 29.60, 29.51, 29.34, 29.29, 27.96, 26.12, 22.75, 14.20. HRMS (ESI+): Found 312.232, $C_{21}H_{30}BrNO$ $[M\text{-}Br^-]^+$ requires 312.2322.

Preparation of oCl-Bn-10 (MAF-009)

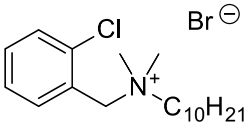

To a 20 mL reaction vial with pressure relieving septum cap were added *N,N*-dimethyldecylamine (0.130 mL, 0.206 g, 1.00 mmol), *o*-chlorobenzylbromide (0.262 mL, 0.204 g, 1.10 mmol), and acetonitrile (3 mL). The mixture was heated to 80 °C with stirring and held for 21 h. After cooling to room temperature, the solvent was removed using rotary evaporation. The resulting oil was triturated with hexane (2 x ~2 mL) to yield **oCl-Bn-10** as a pale yellow oil (0.360 g, 92%): ^{1}H NMR (400 MHz, $CDCl_3$) δ 7.91 (d, *J* = 7.5 Hz, 1H), 7.41 – 7.13 (m, 3H), 4.95 (s, 2H), 3.63 – 3.47 (m, 2H), 3.17 (s, 6H), 1.71 (s, 2H), 1.14 (d, *J* = 45.2 Hz, 14H), 0.72 (t, *J* = 6.9 Hz, 3H). ^{13}C NMR (101 MHz, $CDCl_3$) δ 136.6, 136.31, 132.42, 130.61, 127.84, 125.64, 65.36, 63.70, 49.98, 31.79, 29.37, 29.19, 26.34, 23.13, 22.61, 14.09. HRMS (ESI+): Found 310.2303, $C_{20}H_{35}BrClN$ $[M-Br^{-}]^{+}$ requires 310.2296.

Preparation of pOC6-Bn-8 (MAF-010)

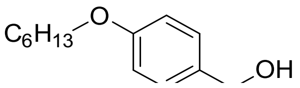

To a 40 mL reaction vial with pressure relieving septum cap were added *p*-hydroxybenzyl alcohol (0.621 g, 5.00 mmol), 1-bromohexane (0.825 g, 5.00 mmol), potassium carbonate (3.45 g, 5 equiv.) and acetone (5 mL). The mixture was heated to 65 °C with stirring and held for 23 h. After cooling to room temperature, the resulting mixture was vacuum filtered and the filtrate removed using rotary evaporation to furnish a light pink solid as **MAF-004** (1.04 g, 91%); ^{1}H NMR (400 MHz, $CDCl_3$) δ 7.26 (dq, *J* = 9.6, 2.3 Hz, 2H), 6.91 – 6.84 (m, 2H), 4.60 (dd, *J* = 5.9, 2.2 Hz,

2H), 3.94 (td, *J* = 6.6, 3.0 Hz, 2H), 1.81 – 1.71 (m, 2H), 1.63 – 1.52 (m, 1H), 1.44 (tp, *J* = 10.1, 3.3 Hz, 2H), 1.38 – 1.27 (m, *J* = 3.4, 2.8 Hz, 4H), 0.89 (dtt, *J* = 5.6, 3.9, 1.6 Hz, 3H).

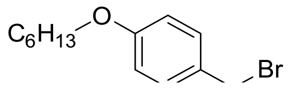

To a 25 mL round bottom flask was added **MAF-004** and dichloromethane (3 mL). The solution was placed under argon and cooled to 0 °C. With stirring, phosphorus tribromide (0.207 mL, 0.302 g, 2.17 mmol) was added dropwise over 10 minutes. The mixture was allowed to warm to room temperature and held for 2 h. Water (5 mL) was added dropwise over several minutes followed by dichloromethane (10 mL). The organic layer was washed with saturated sodium bicarbonate (3 x 10 mL) and dried over anhydrous magnesium sulfate. Removal of the solvent via rotary evaporation to furnish a colorless liquid as **MAF-007** (0.383 g, 97%). ^{1}H NMR (400 MHz, $CDCl_3$) δ 7.34 – 7.24 (m, 2H), 6.88 – 6.78 (m, 2H), 4.49 (s, 2H), 3.94 (t, *J* = 6.6 Hz, 2H), 1.76 (dq, *J* = 7.9, 6.6 Hz, 2H), 1.45 (ddt, *J* = 9.4, 6.9, 3.3 Hz, 2H), 1.43 – 1.31 (m, 2H), 1.35 – 1.28 (m, 2H), 0.95 – 0.85 (m, 3H).

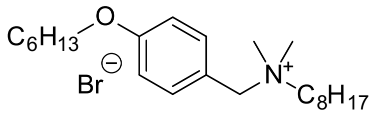

To a 20 mL reaction vial with pressure relieving septum cap were added **MAF-007** (0.140 g, 0.516 mmol), *N,N*-dimethyloctylamine (0.0890 g, 0.567 mmol, 1.10 mmol), and acetonitrile (3 mL). The mixture was heated to 80 °C with stirring and held for 22 h. After cooling to room temperature, the solvent was removed using rotary evaporation to yield **pOC6-Bn-8** (0.149 g, 68%); ^{1}H NMR (400 MHz, $CDCl_3$) δ 7.53 (dd, *J* = 8.8, 2.2 Hz, 2H), 6.91 (ddd, *J* = 10.5, 7.5, 1.8 Hz, 2H), 4.94 (d, *J* = 15.4 Hz, 2H), 3.94 (q, *J* = 6.4 Hz, 2H), 3.49 – 3.42 (m, 2H), 3.24 (s, 6H),

2.29 (d, J = 7.2 Hz, 2H), 1.99 (d, J = 1.5 Hz, 4H), 1.81 – 1.73 (m, 2H), 1.76 (s, 4H), 1.44 (s, 2H), 1.36 – 1.32 (m, 2H), 1.35 – 1.26 (m, 8H), 1.24 (s, 6H), 0.93 – 0.82 (m, 6H). ^{13}C NMR (101 MHz, $CDCl_3$) δ 160.90, 134.66, 118.94, 114.95, 77.55, 77.44, 77.23, 76.91, 68.20, 68.02, 67.23, 63.39, 49.36, 31.77, 31.66, 31.59, 29.72, 29.30, 29.25, 29.16, 29.13, 29.09, 27.06, 26.38, 25.88, 25.72, 22.95, 22.61, 22.43, 14.11, 14.08. HRMS (ESI+): Found 348.3255, $C_{23}H_{42}BrNO$ $[M-Br^{-}]^{+}$ requires 348.3261.

Preparation of Urea-9,pClPh (MAF-019)

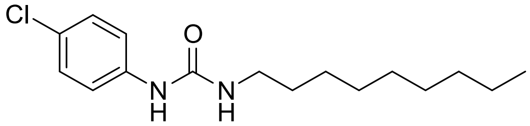

To a 20 mL reaction vial with pressure relieving septum cap was added nonylamine (0.287 g, 0.200 mol) and 2 mL dimethylformamide. A solution of *p*-chloroisocyanate (0.308 g, 0.002 mol) dissolved in dimethylformamide (2 mL) was added over 10 minutes. The mixture was stirred at room temperature for 1.5 h during which time a white precipitate formed. The reaction vial was then heated for 2-3 minutes at 40 °C until the white precipitate dissolved. The reaction was then allowed to cool to room temperature over 30 minutes during which time a white precipitate reformed. Water (5 mL) was added dropwise and the white precipitate vacuum filtered and washed well with water (0.481 g, 81%); ^{1}H NMR (400 MHz, DMSO-D_6) δ 7.41 – 7.32 (m, 2H), 7.25 – 7.15 (m, 2H), 6.10 (t, J = 5.7 Hz, 1H), 3.01 (td, J = 6.9, 5.6 Hz, 2H), 1.37 (p, J = 6.8 Hz, 2H), 1.22 (p, J = 5.4 Hz, 12H), 0.86 – 0.77 (m, 3H). ^{13}C NMR (101 MHz, DMSO-D_6) δ 155.55, 155.48, 140.11, 140.01, 128.94, 124.84, 119.53, 119.43, 40.59, 40.38, 40.17, 39.96, 39.75, 39.54, 39.43, 39.33, 31.82, 30.22, 30.20, 29.54, 29.32, 29.21, 26.89, 22.64, 14.47. HRMS (ESI+): Found 297.1733, $C_{16}H_{26}ClN_2O$ $[M-Br^{-}]^{+}$ requires 296.8390.

Preparation of DPA-9E,9E (MAF-012)

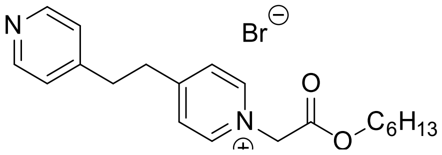

To a 20 mL reaction vial with pressure relieving septum cap were added hexyl 2-bromoacetate (**MAF-005)** (0.293 g, 1.00 mmol), 1,2-bis(4-pyridyl)ethane (0.184 g, 1.00 mmol), and acetonitrile (3 mL).[12] The mixture was heated to 80 °C with stirring and held for 20 h. After cooling to room temperature, the solvent was removed using rotary evaporation to yield **MAF-011** as a dark brown solid (0.477 g, 91%); ^{1}H NMR (500 MHz, CD_3OD) δ 8.85 (dd, *J* = 22.0, 6.9 Hz, 2H), 8.60 – 8.41 (m, 1H), 8.10 (dd, *J* = 42.2, 8.4 Hz, 2H), 7.71 – 7.40 (m, 1H), 5.55 (d, *J* = 15.8 Hz, 1H), 4.24 (td, *J* = 6.7, 3.7 Hz, 2H), 3.58 – 3.10 (m, 4H), 1.85 – 1.59 (m, 2H), 1.49 – 1.18 (m, 6H), 0.89 (tdd, *J* = 8.5, 5.5, 2.5 Hz, 3H).

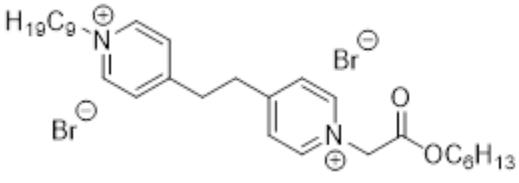

To a 20 mL reaction vial with pressure relieving septum cap were added MAF-011 (0.1845 g, 0.387 mmol), 1-bromononane (0.160 g, 0.774 mmol), and acetonitrile (3 mL).[12] The mixture was heated to 80 °C with stirring and held for 22 h. After cooling to room temperature, the solvent was removed using rotary evaporation and triturated in hexanes to yield **DPA-9E,9E (MAF-012)** as a dark brown solid (0.134 g, 56%); ^{1}H NMR (400 MHz, CD_3OD) δ 8.97 – 8.66 (m, 4H), 8.24 – 7.89 (m, 4H), 5.57 (s, 2H), 4.58 (t, *J* = 7.6 Hz, 1H), 4.24 (t, *J* = 6.7 Hz, 2H), 3.46 (t, *J* = 12.7 Hz, 4H),

1.99 (s, 1H), 1.81 – 1.59 (m, 3H), 1.47 – 1.15 (m, 18H), 0.89 (d, J = 6.3 Hz, 6H). ^{13}C NMR (126 MHz, CD_3OD) δ 166.19, 145.75, 144.17, 128.13, 127.81, 127.22, 66.81, 63.40, 61.14, 59.92, 34.41, 34.16, 31.65, 31.22, 31.10, 29.15, 28.98, 28.80, 28.16, 25.87, 25.23, 23.93, 22.37, 22.27, 13.10, 13.02. HRMS (ESI+): Found 454.3500, $C_{29}H_{46}BrN_2O_2$ $[M-Br^-]^+$ requires 454.3548.

VI. NMR Spectroscopic Data: ^{1}H and ^{13}C NMR

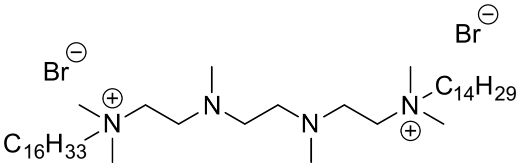

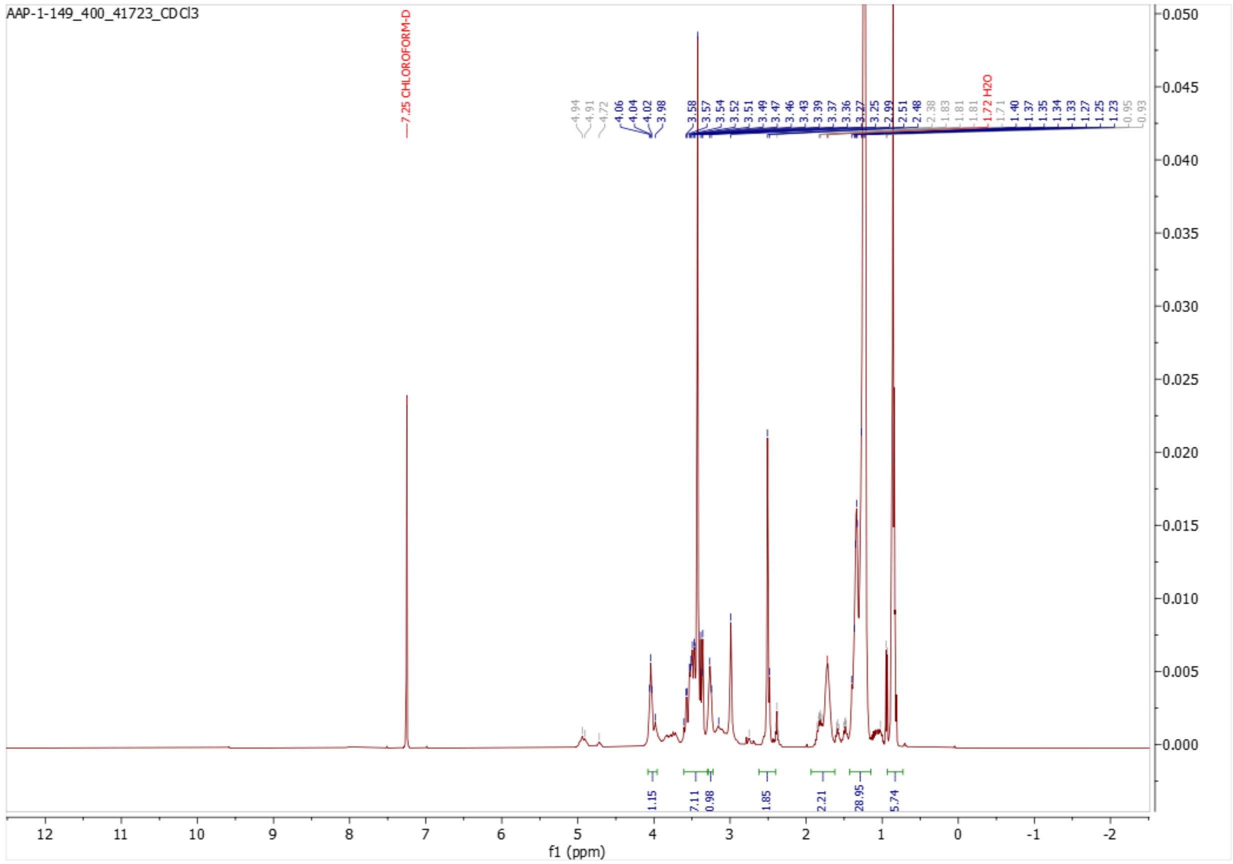

Figure S1: ^{1}H NMR of **4N-16, 14 (AAP149)** in $CDCl_3$.

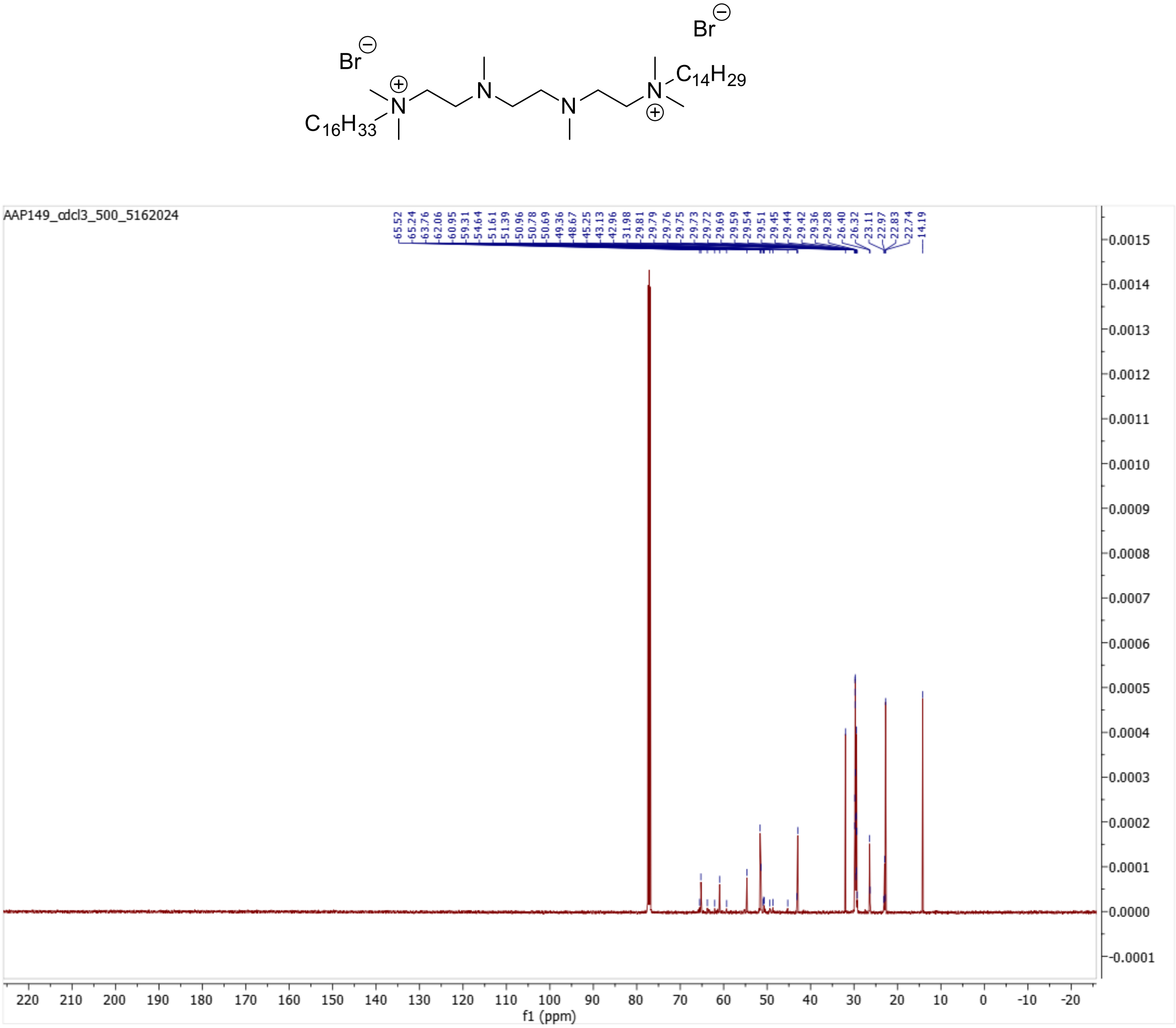

Figure S2: ^{13}C NMR of **4N-16, 14 (AAP149)** in $CDCl_3$.

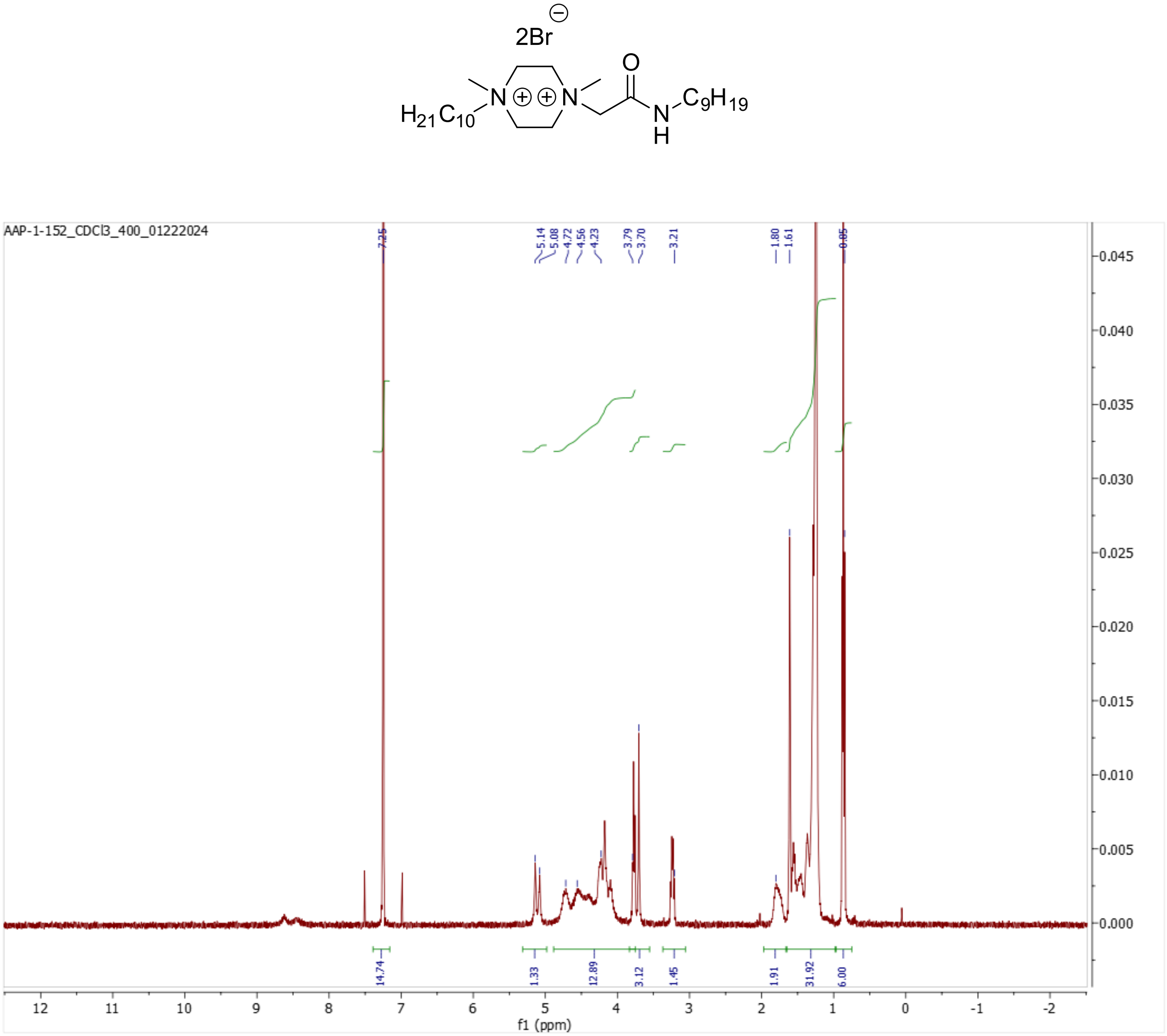

Figure S3: ^{1}H NMR of **PIP-10, 12A (AAP152)** in $CDCl_3$.

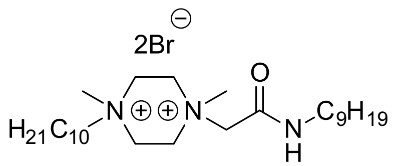

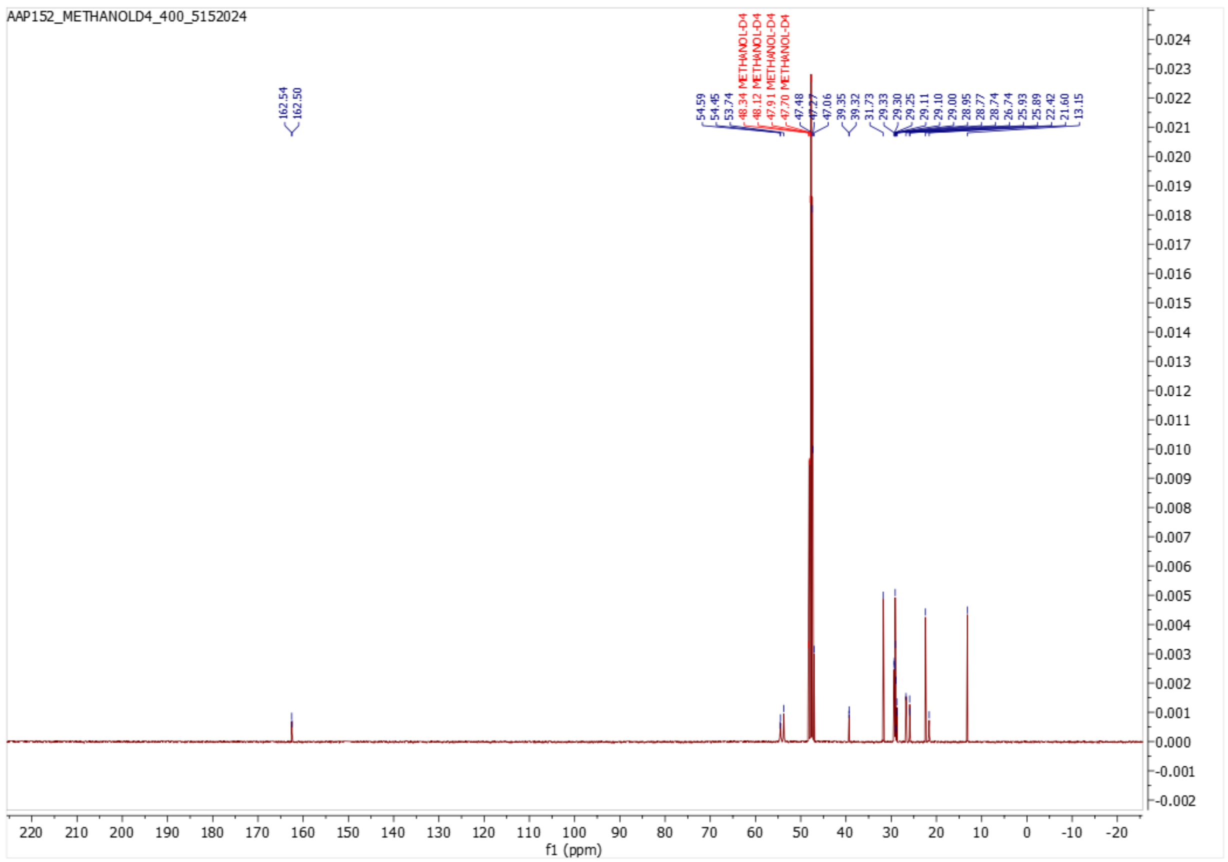

Figure S4: ^{13}C NMR of **PIP-10, 12A (AAP152)** in $CDCl_3$.

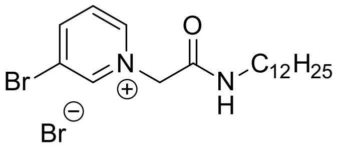

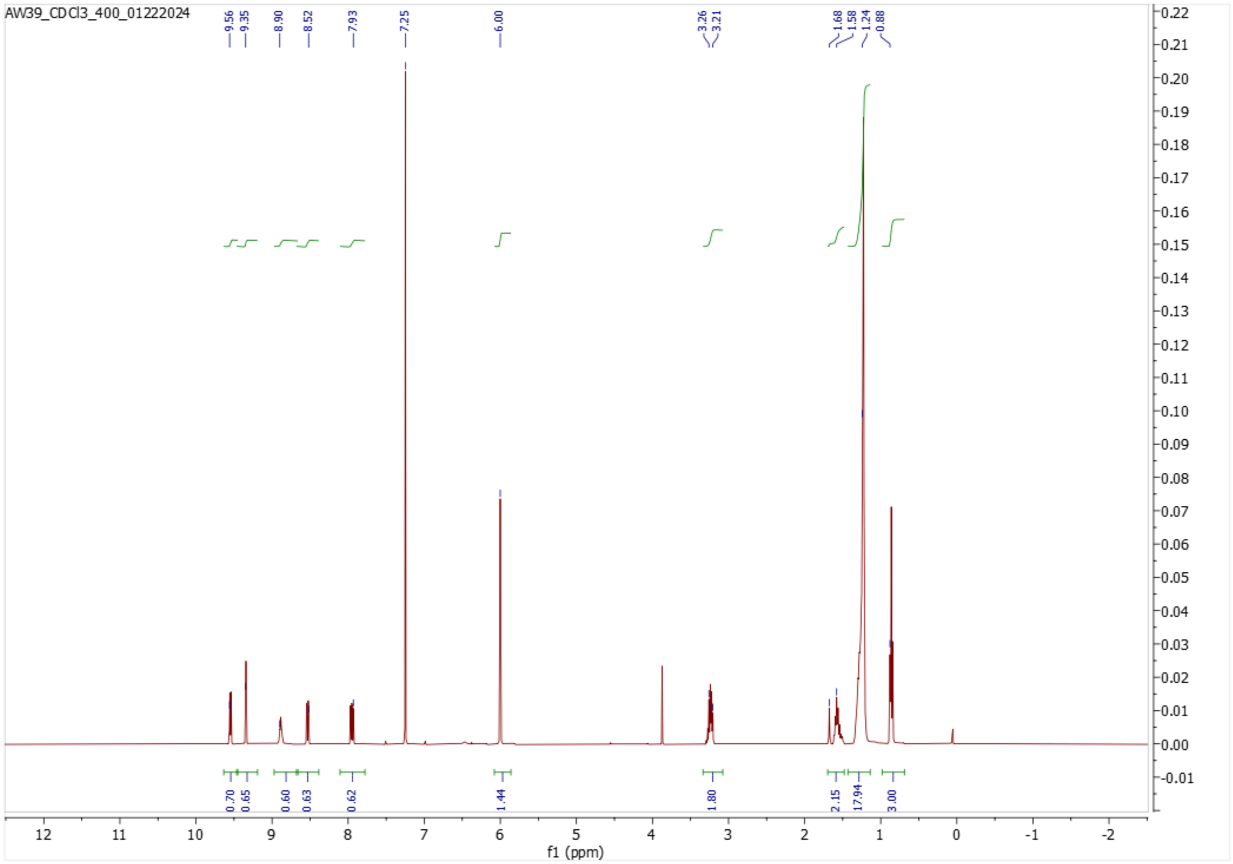

Figure S5: ^{1}H NMR of **mBrPyr-15A (AW39)** in $CDCl_3$.

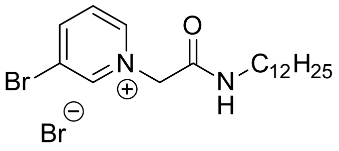

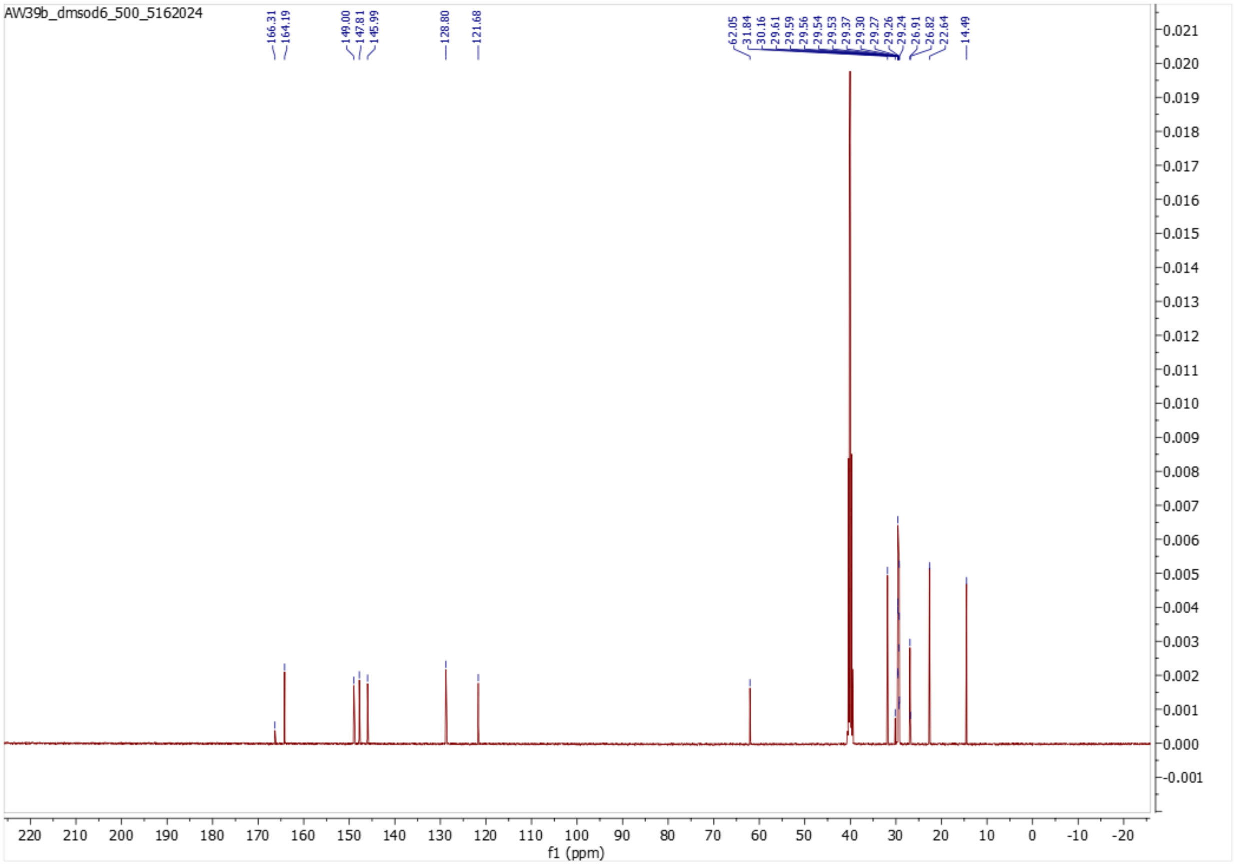

Figure S6: ^{13}C NMR of **mBrPyr-15A (AW39)** in DMSO-d_6.

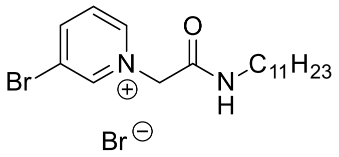

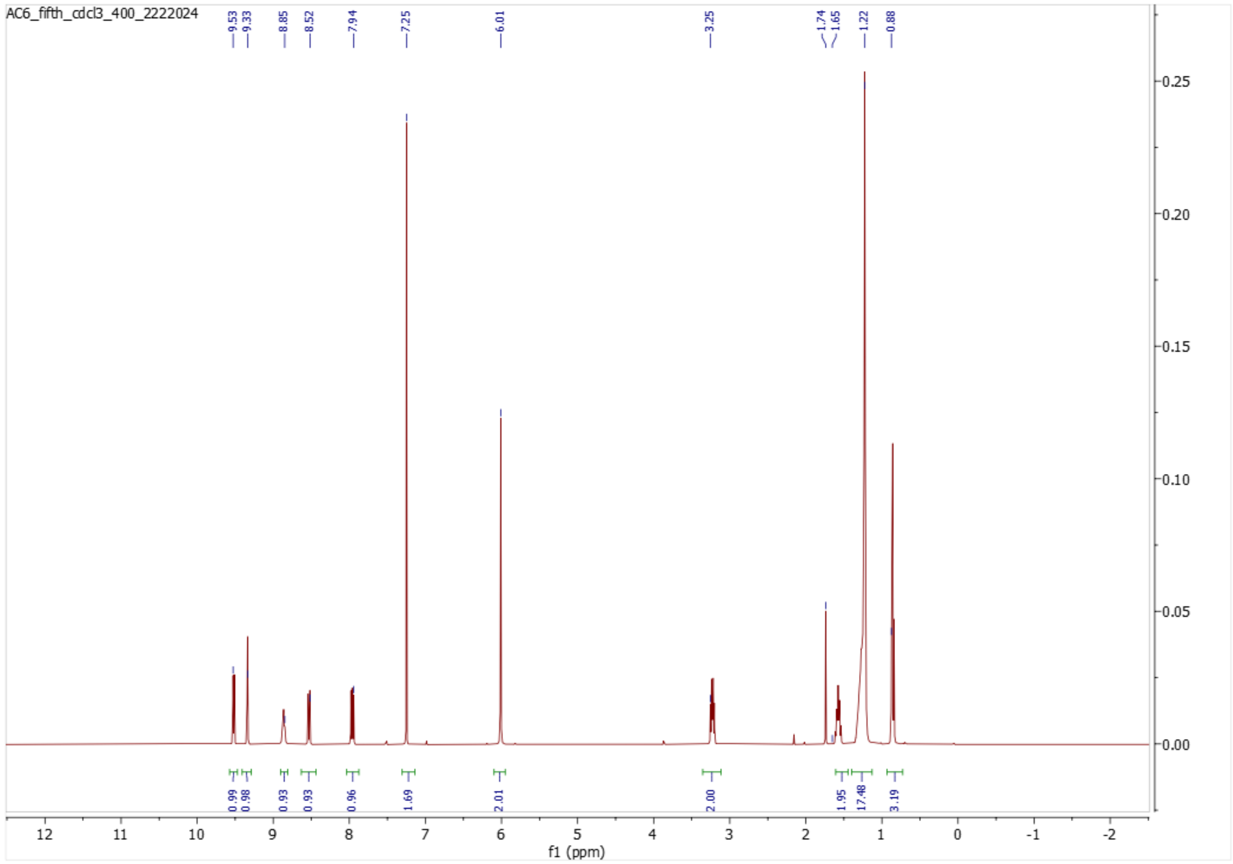

Figure S7: ^{1}H NMR of **mBrPyr-14A (AC6)** in $CDCl_3$.

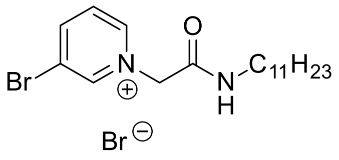

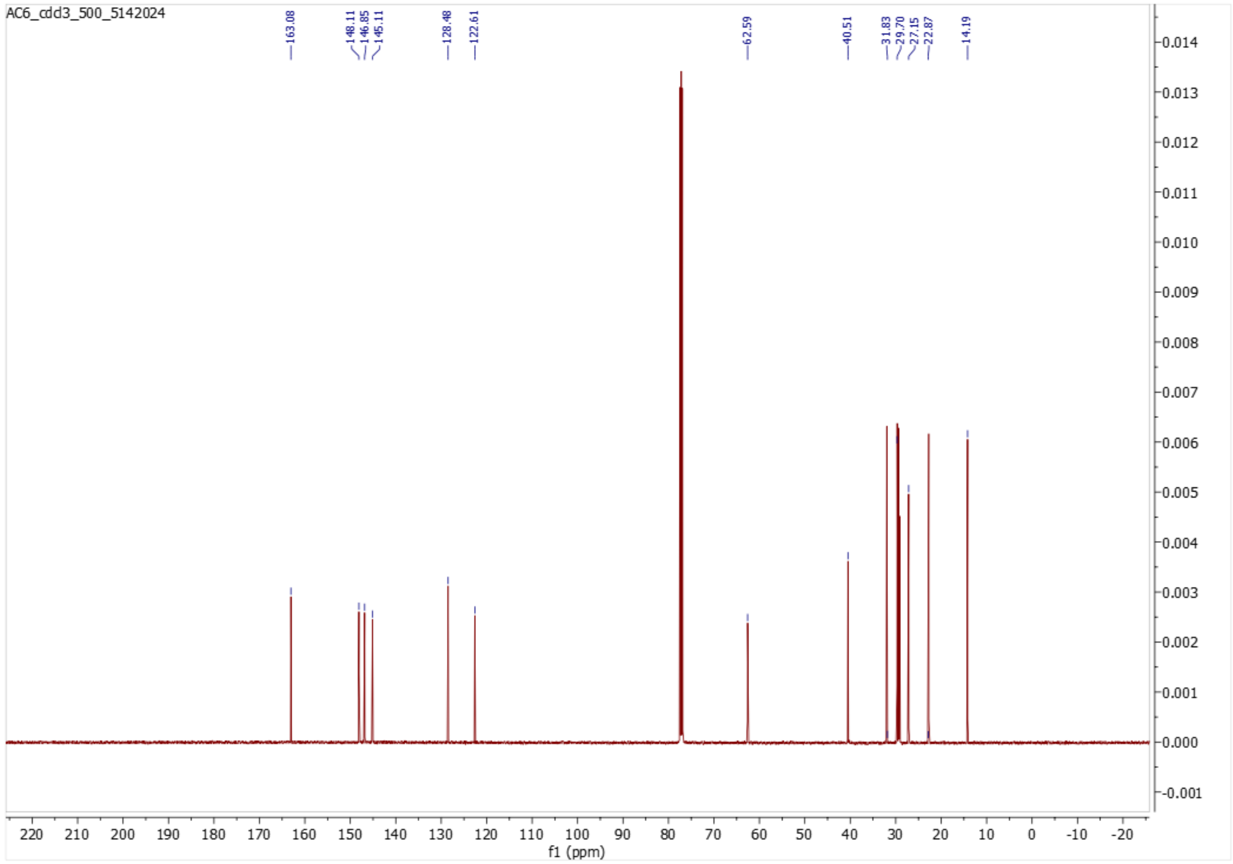

Figure S8: ^{13}C NMR of **mBrPyr-14A (AC6)** in $CDCl_3$.

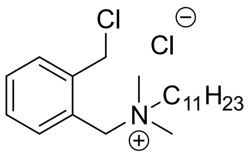

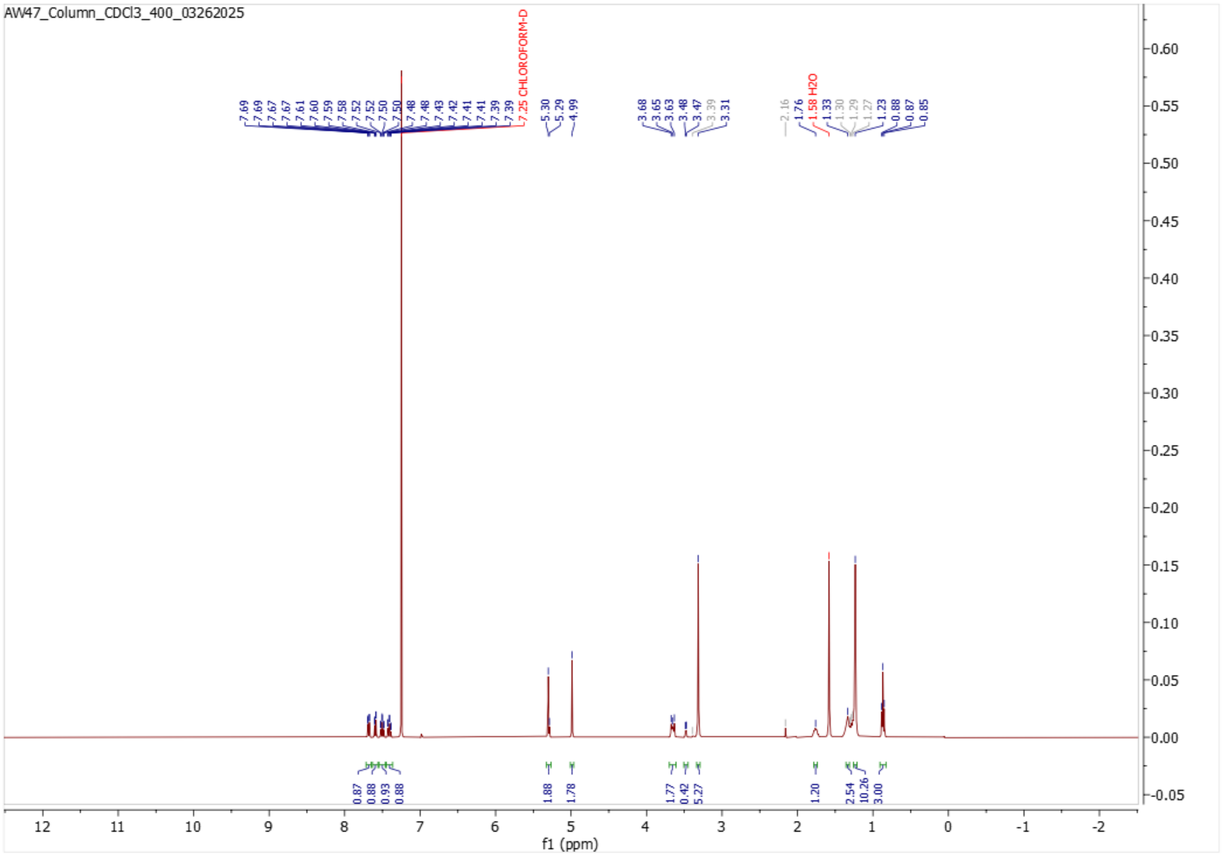

Figure S9: ^{1}H NMR of **AW47** in $CDCl_3$.

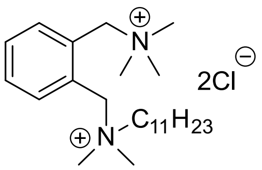

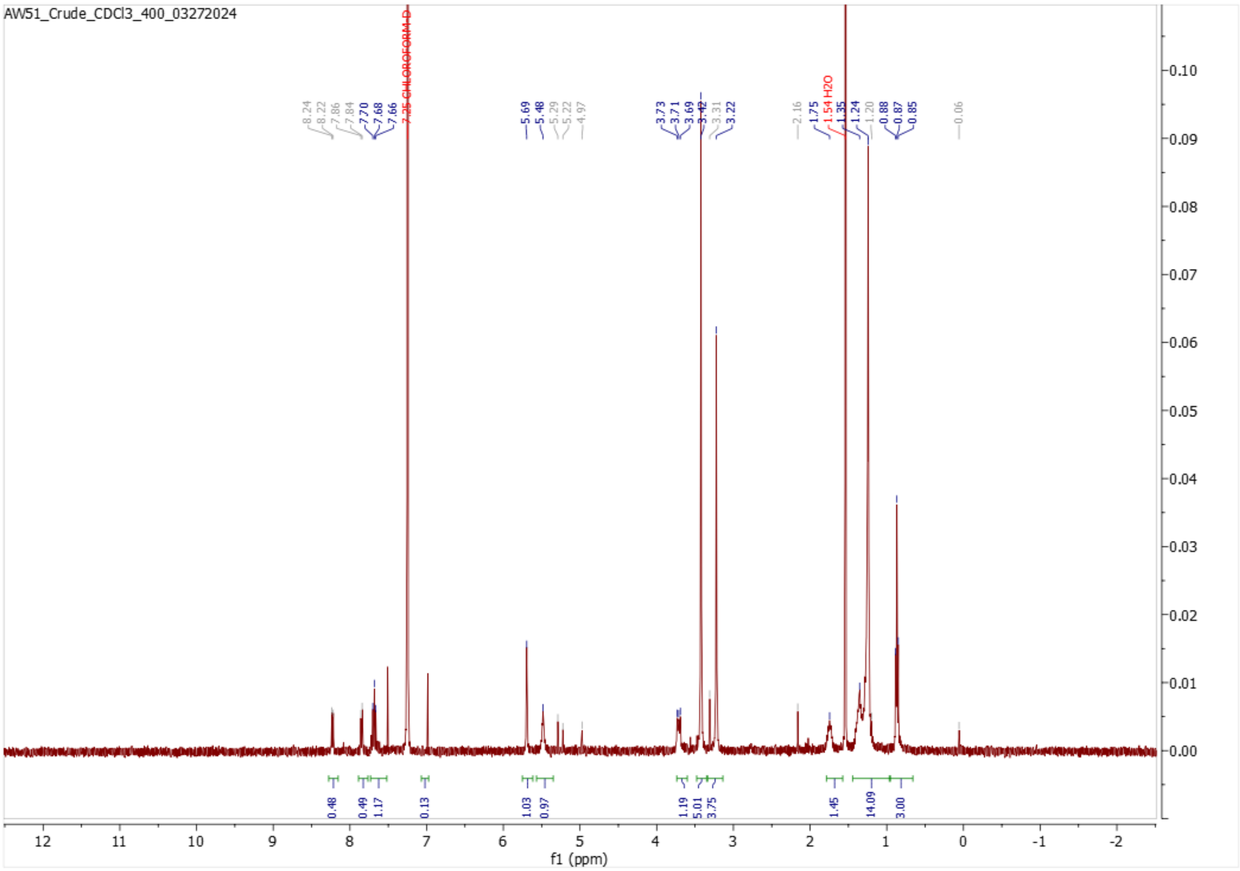

Figure S10: ^{1}H NMR of **oX-11, 1 (AW51)** in $CDCl_3$.

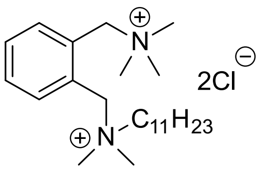

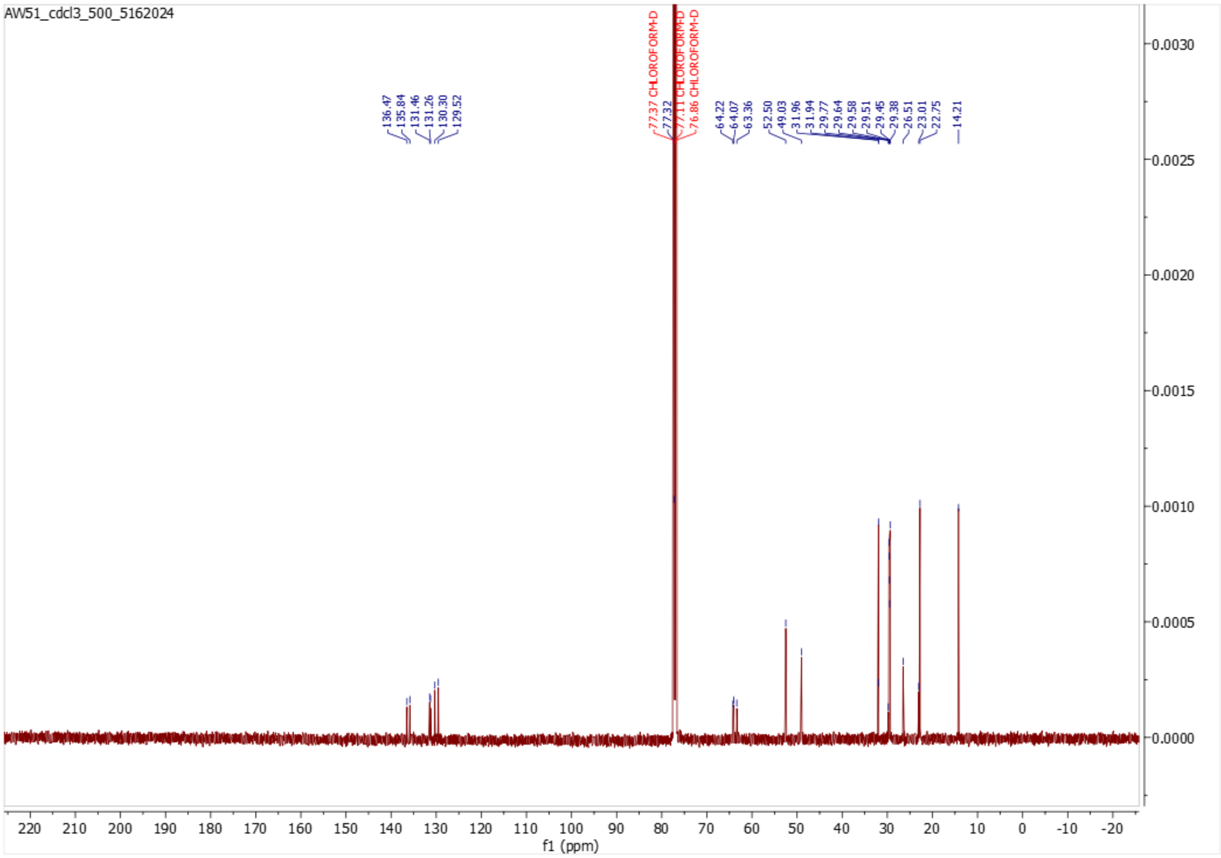

Figure S11: ^{13}C NMR of **oX-11, 1 (AW51)** in $CDCl_3$.

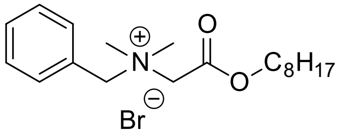

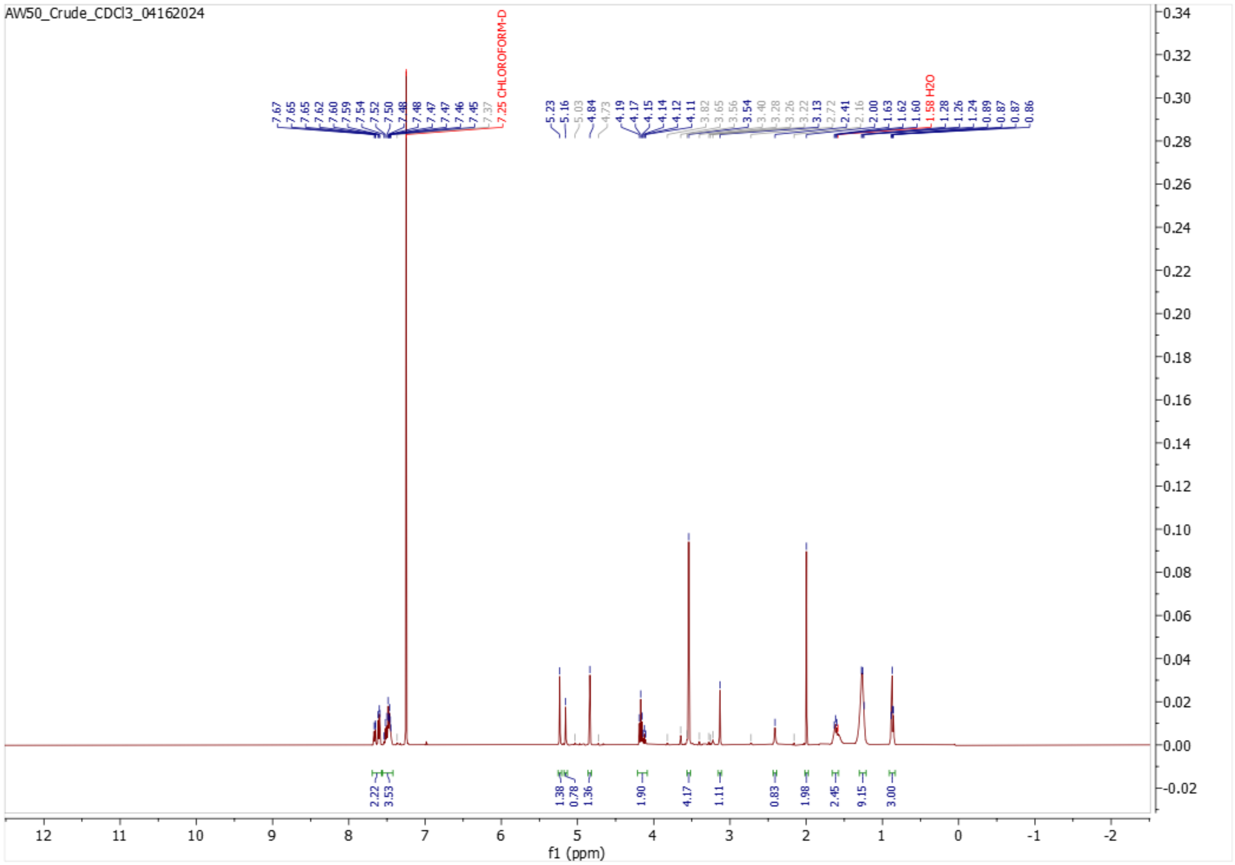

Figure S12: ^{1}H NMR of **Bn-8E (AW50)** in $CDCl_3$.

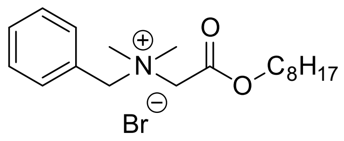

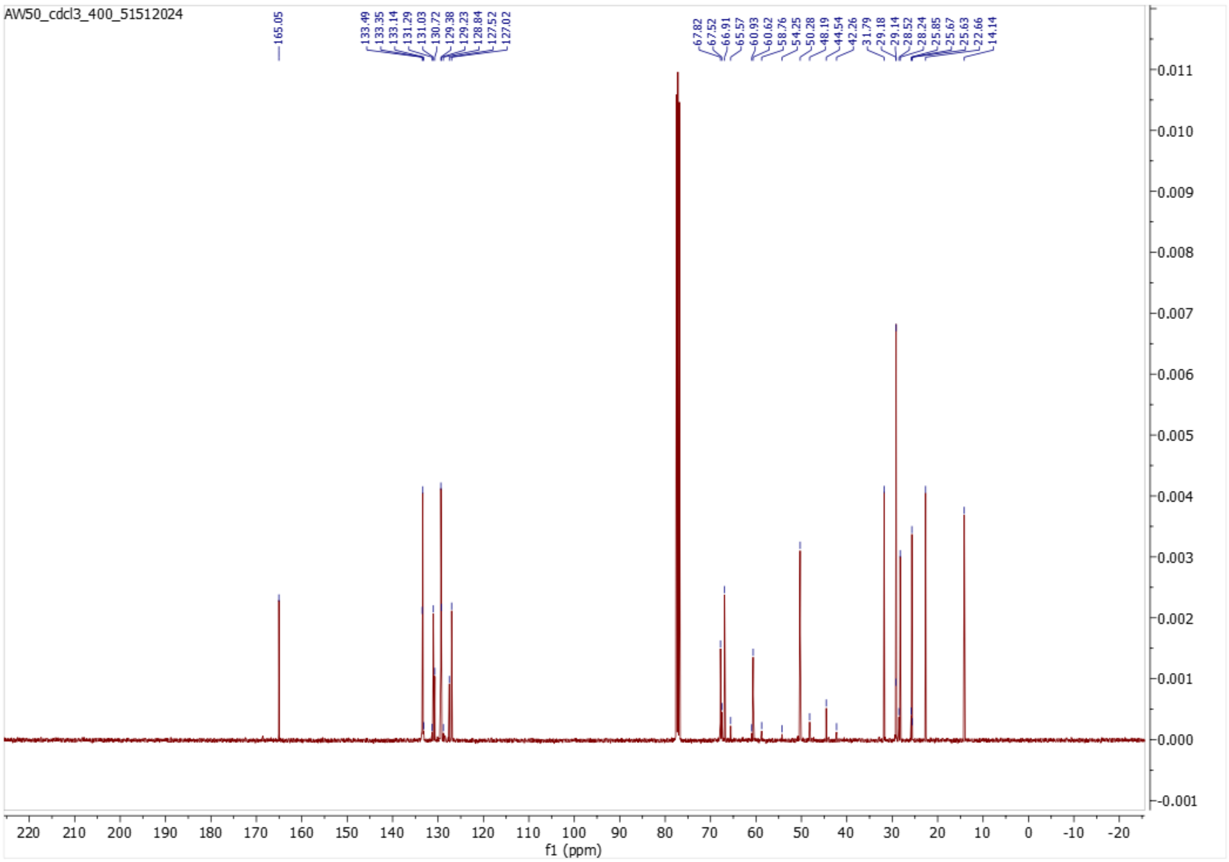

Figure S13: ^{13}C NMR of **Bn-8E (AW50)** in $CDCl_3$.

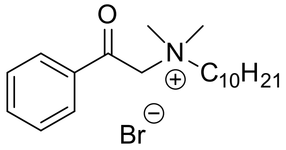

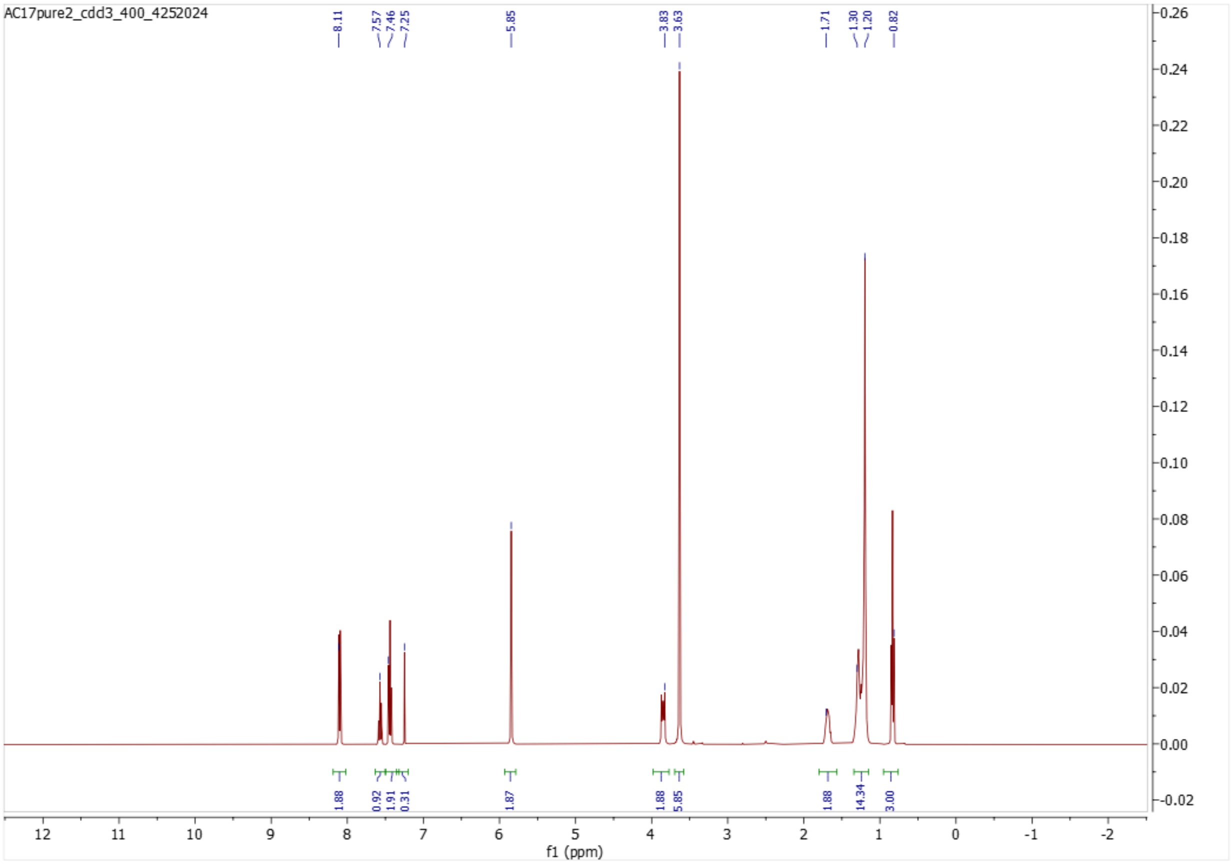

Figure S14: ^{1}H NMR of **ACP-10 (AC17)** in $CDCl_3$.

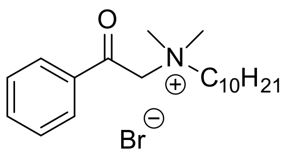

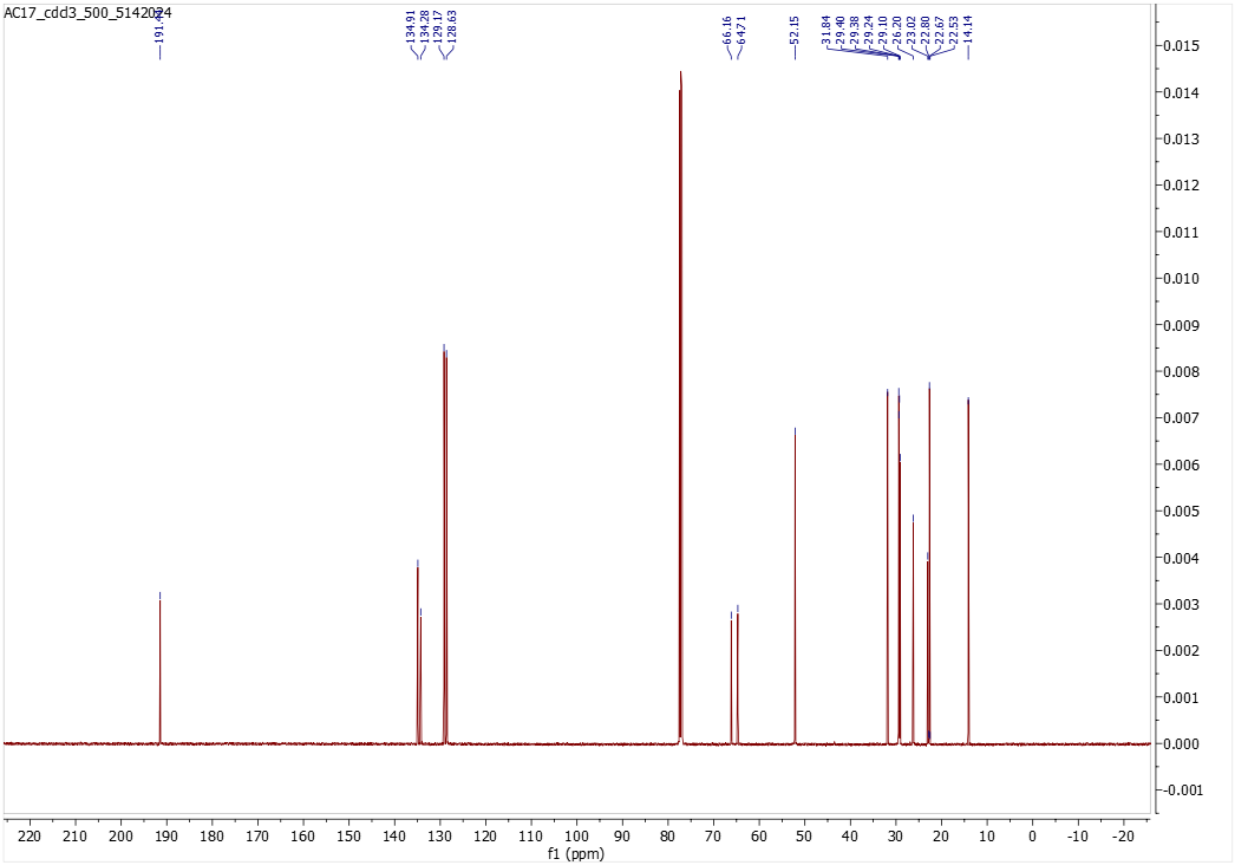

Figure S15: ^{13}C NMR of **ACP-10 (AC17)** in $CDCl_3$.

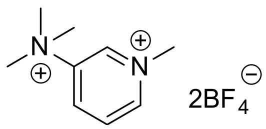

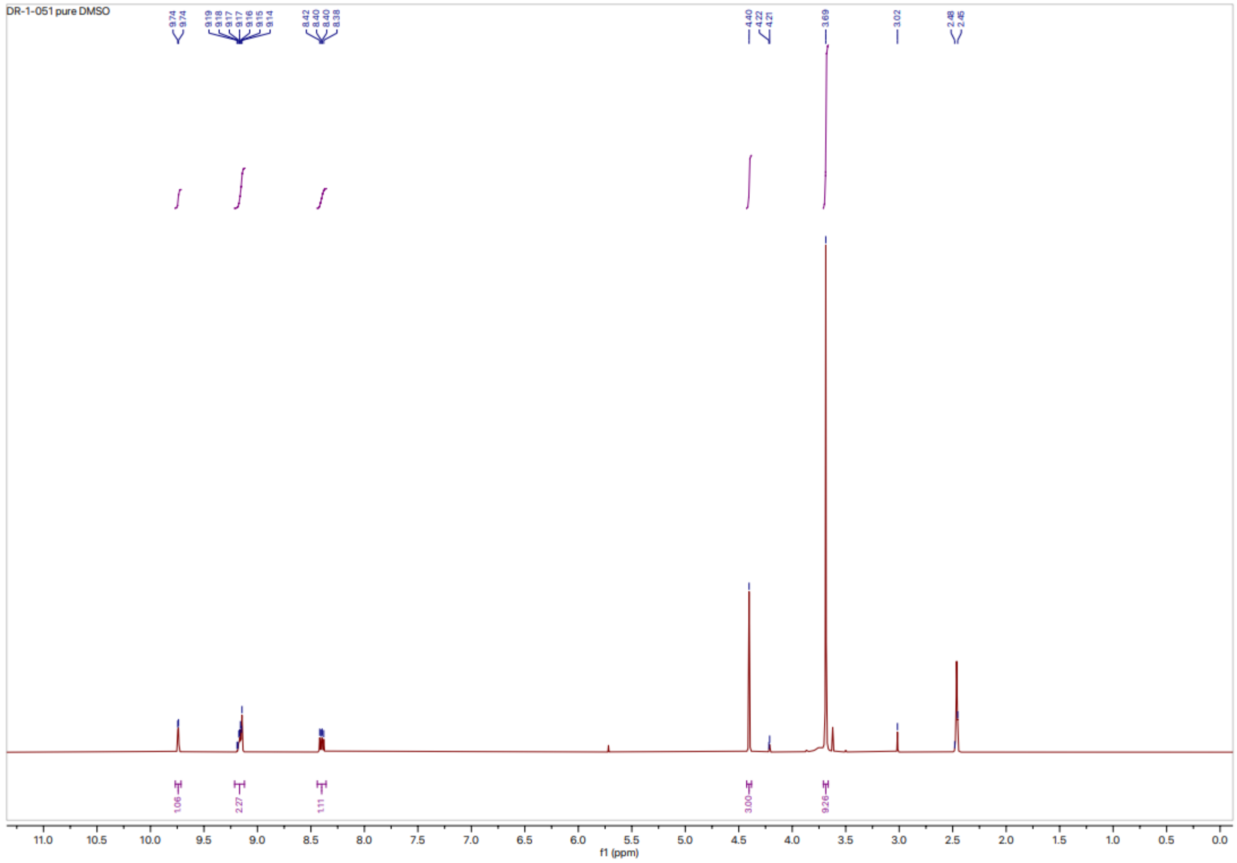

Figure S16: ^{1}H NMR of **Pyr-3 (DR-1-051)** in $CDCl_3$.

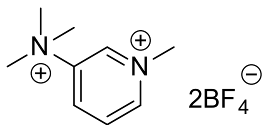

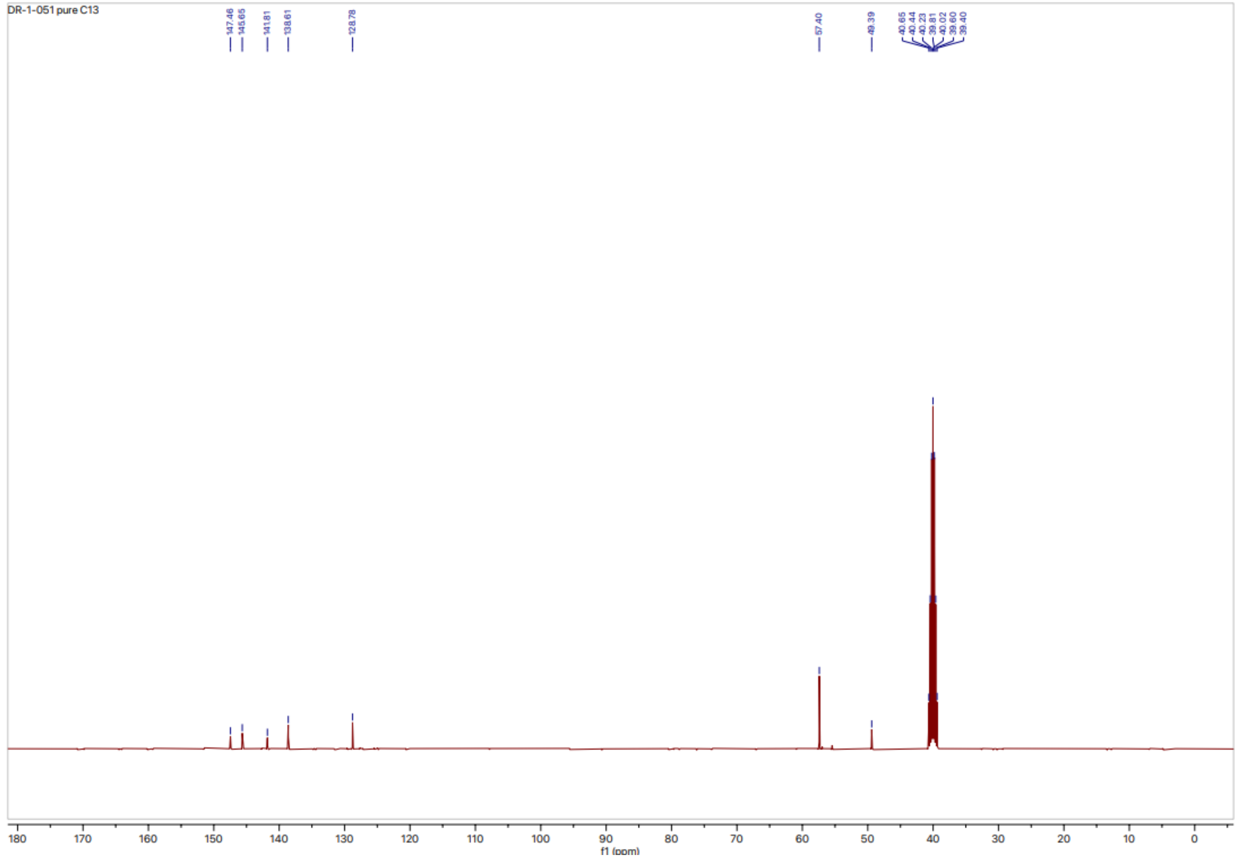

Figure S17: ^{13}C NMR of **Pyr-3 (DR-1-051)** in $CDCl_3$.

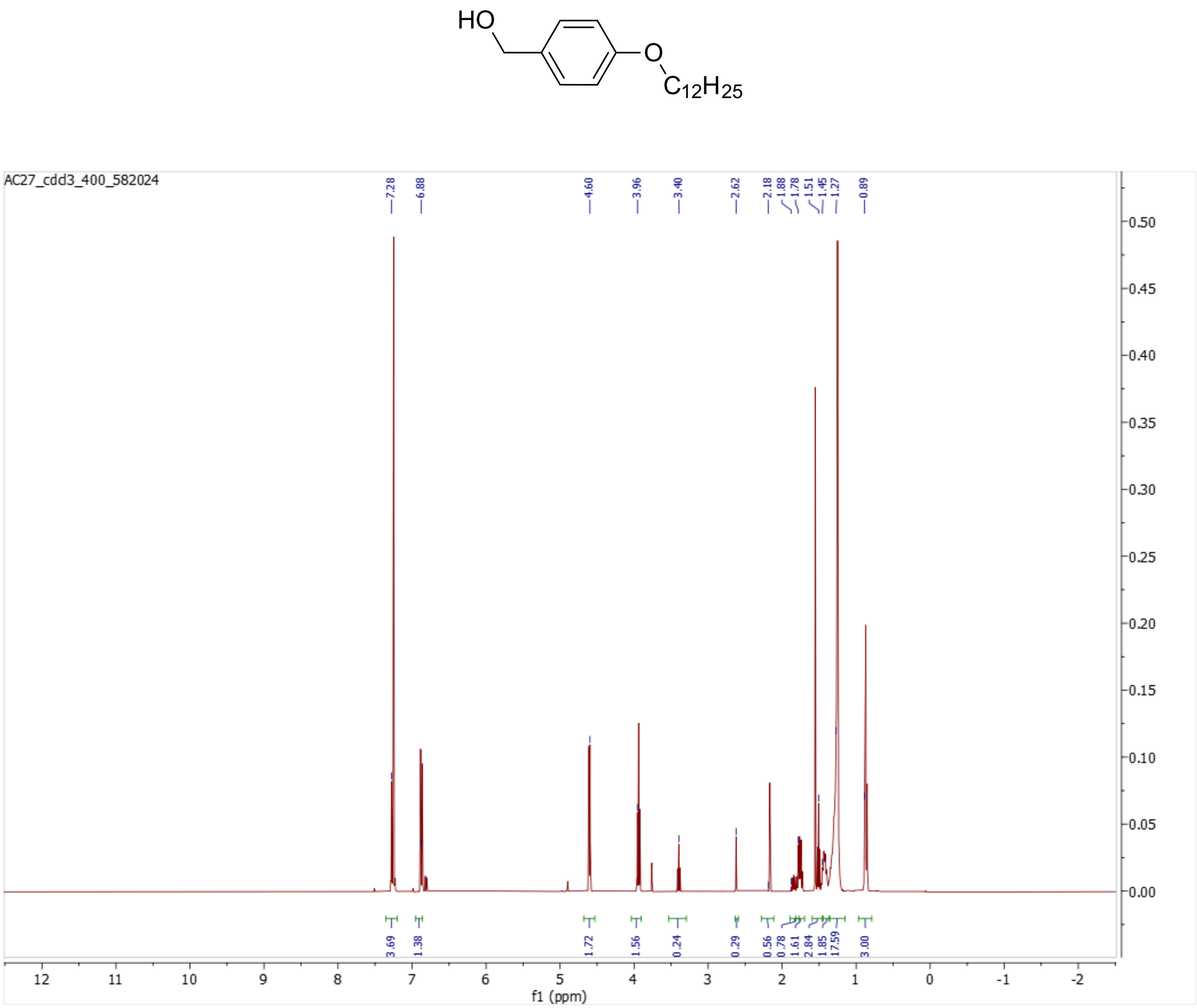

Figure S18: ^{1}H NMR (400 MHz) of **(4-dodecoxyphenyl)methanol** (**AC27**) in $CDCl_3$.

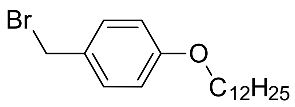

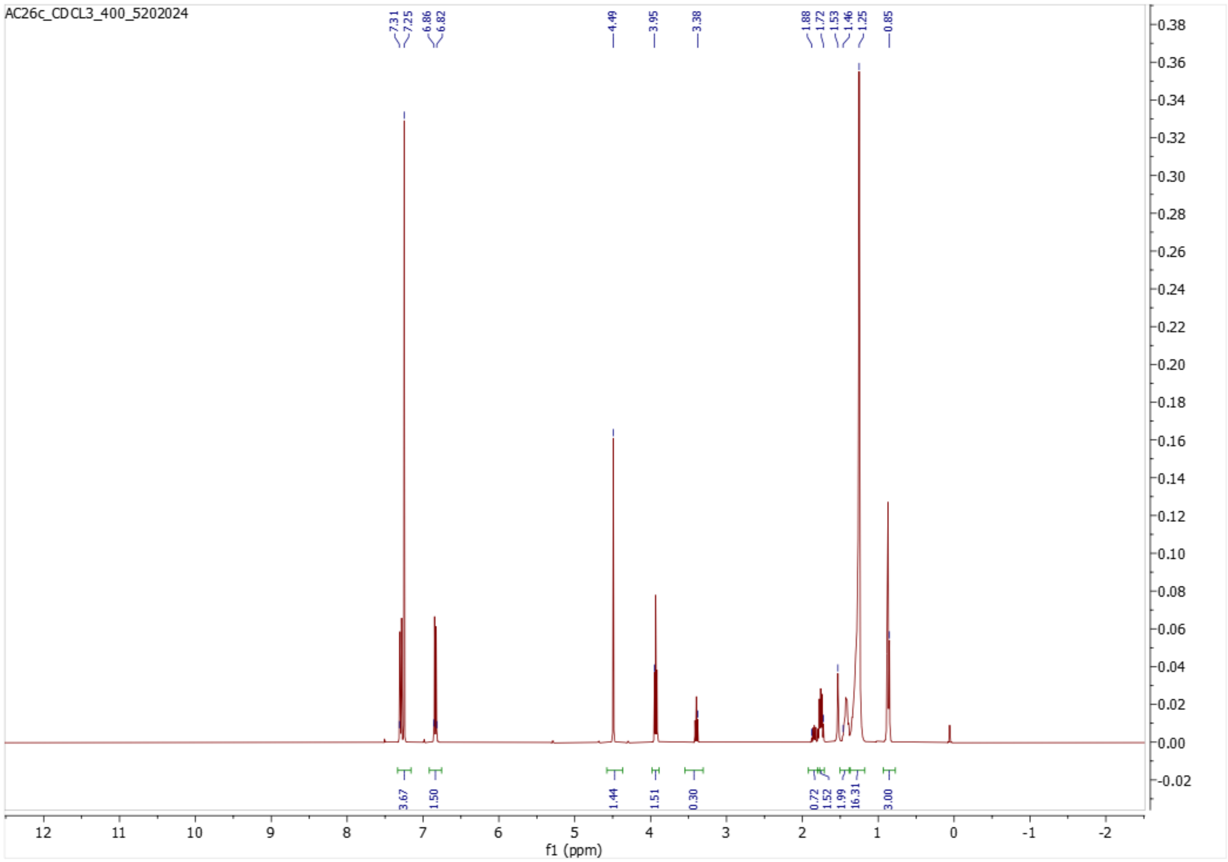

Figure S19: ^{1}H NMR (400 MHz) of **4-(dodecyloxy)benzyl bromide (AC26)** in $CDCl_3$.

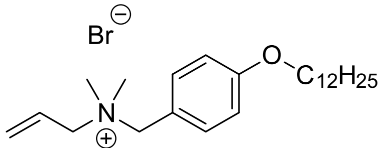

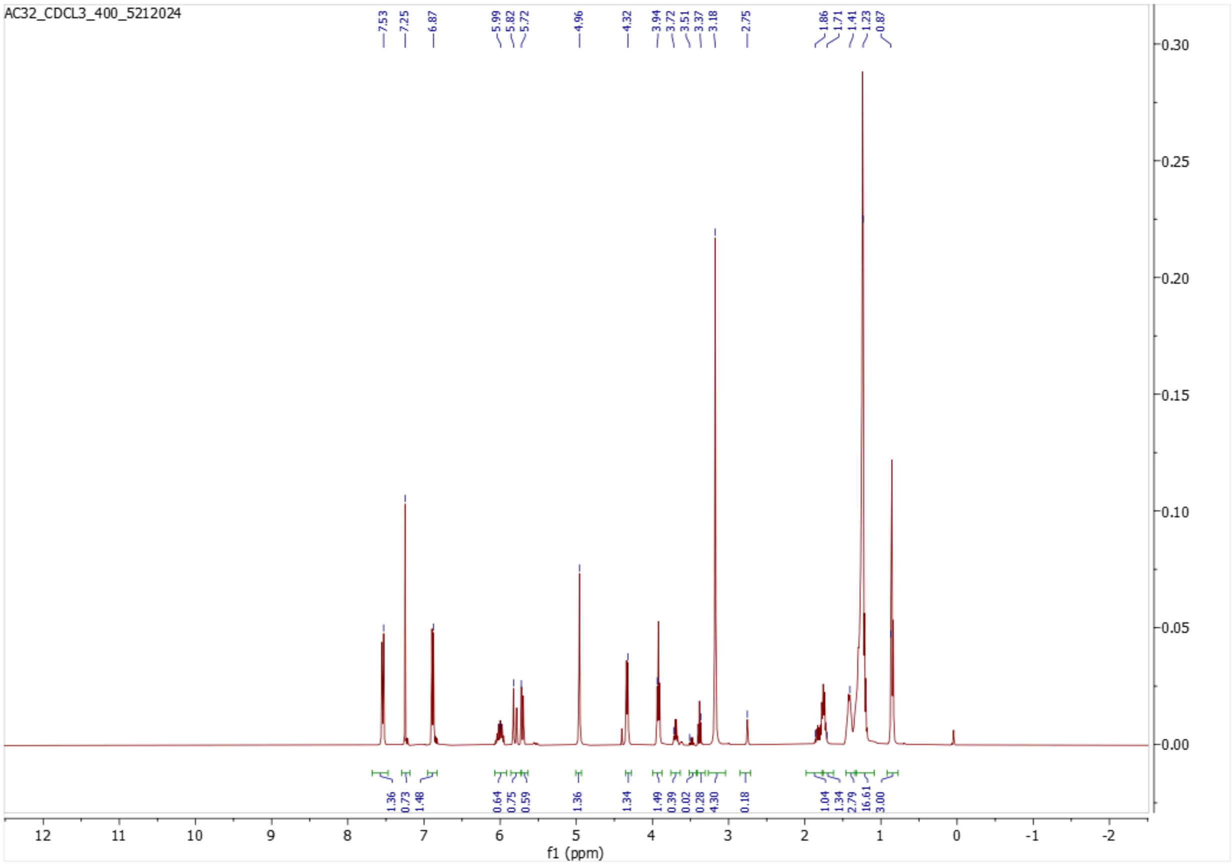

Figure S20: ^{1}H NMR (400 MHz) of **Allyl-pOC12Bn (AC32)** in $CDCl_3$.

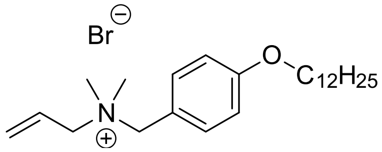

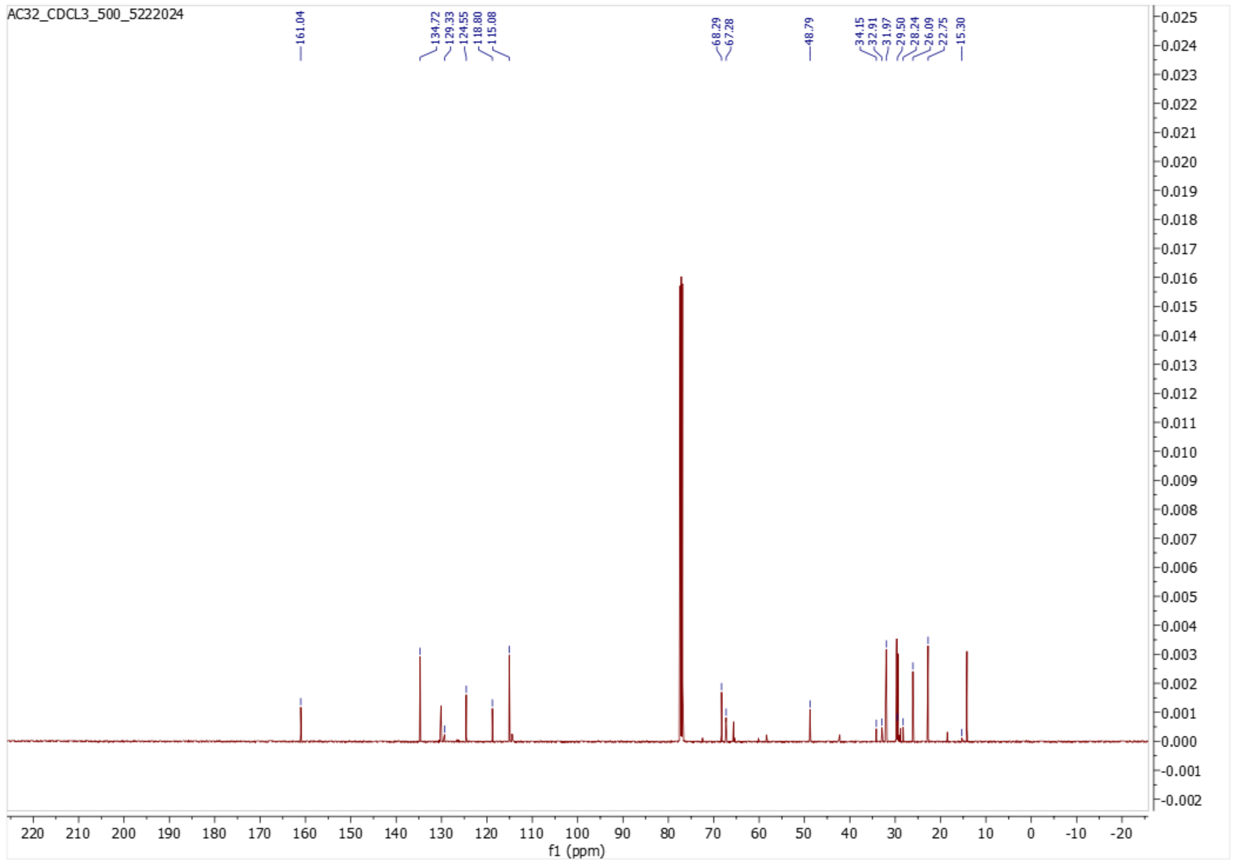

Figure S21: ^{13}C NMR (500 MHz) of **Allyl-pOC12Bn (AC32)** in $CDCl_3$.

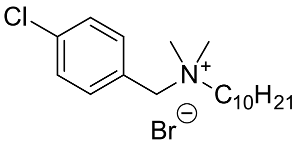

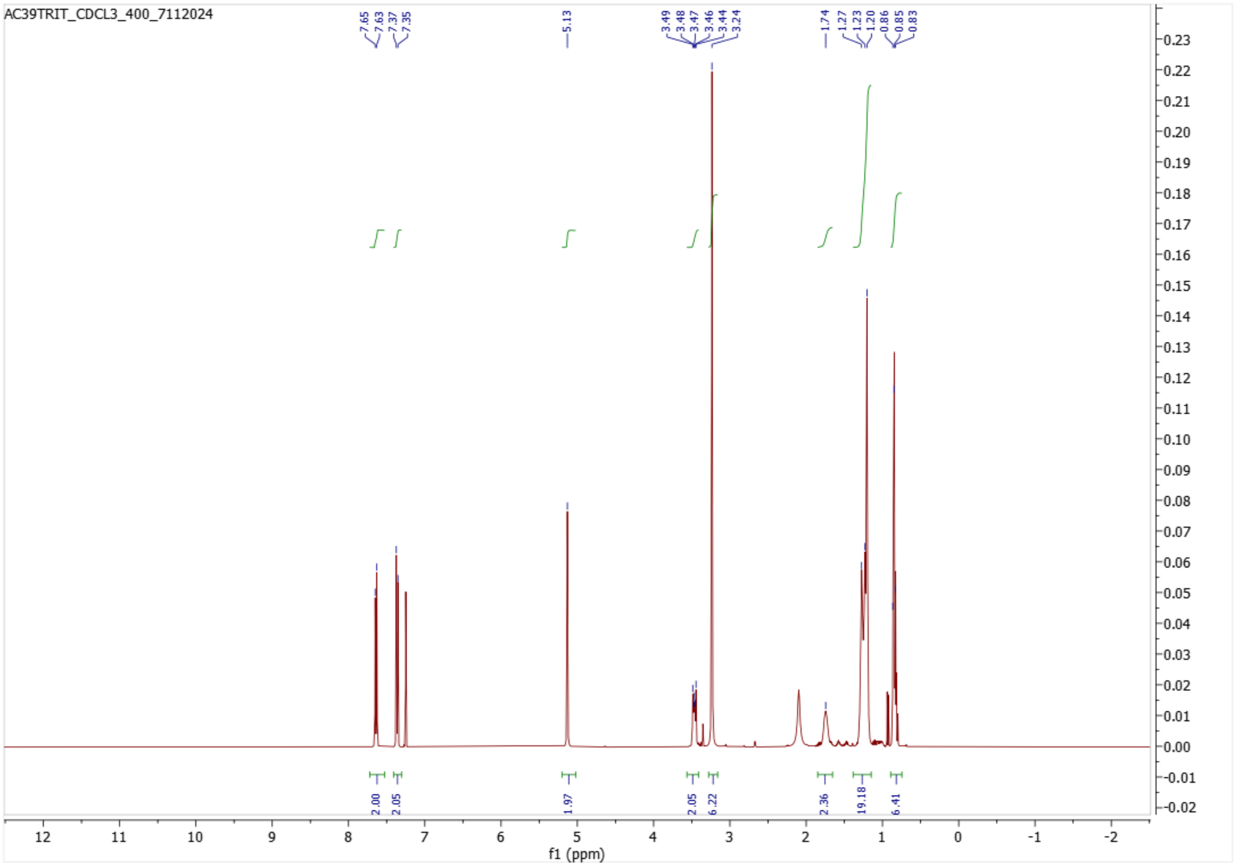

Figure S22: ^{1}H NMR (400 MHz) of **pCl-Bn-10 (AC39)** in $CDCl_3$.

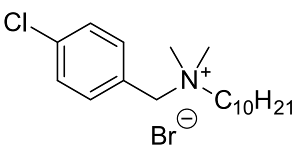

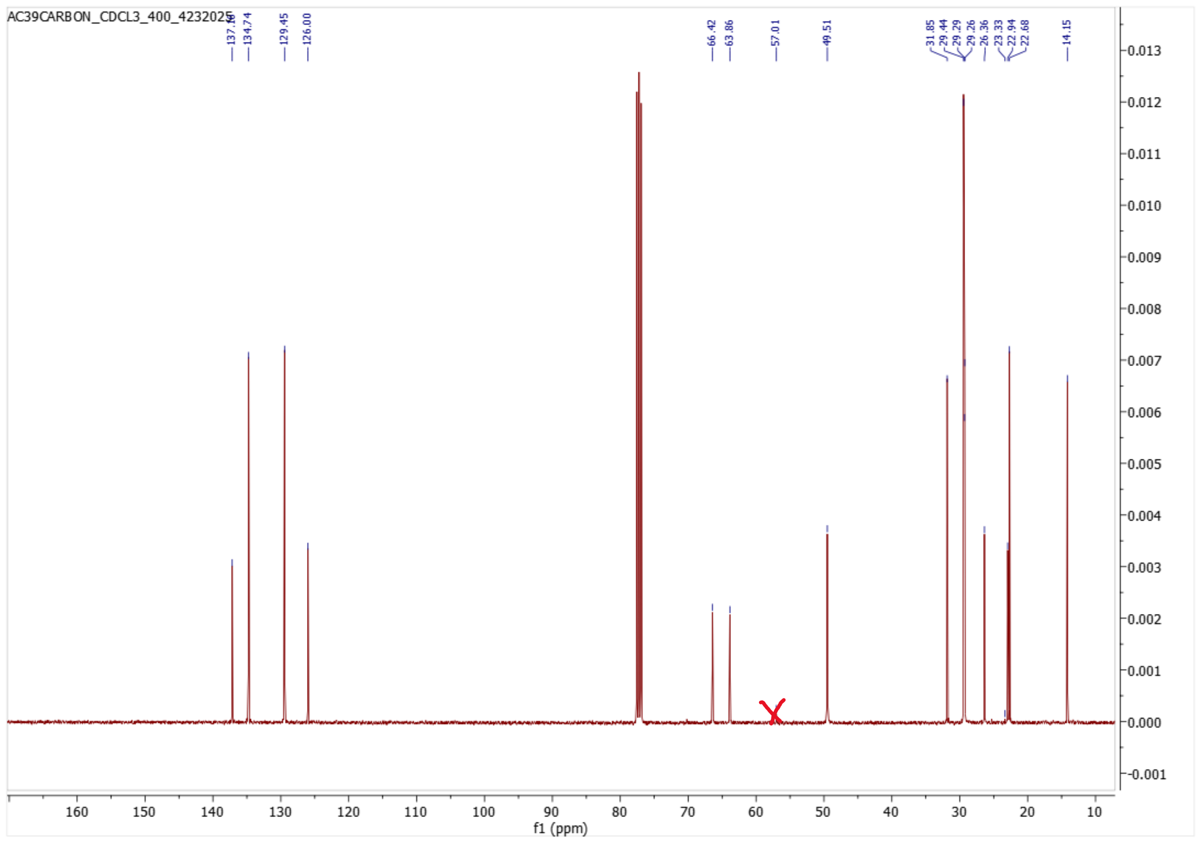

Figure S23: ^{13}C NMR (500 MHz) of **pCl-Bn-10** in $CDCl_3$.

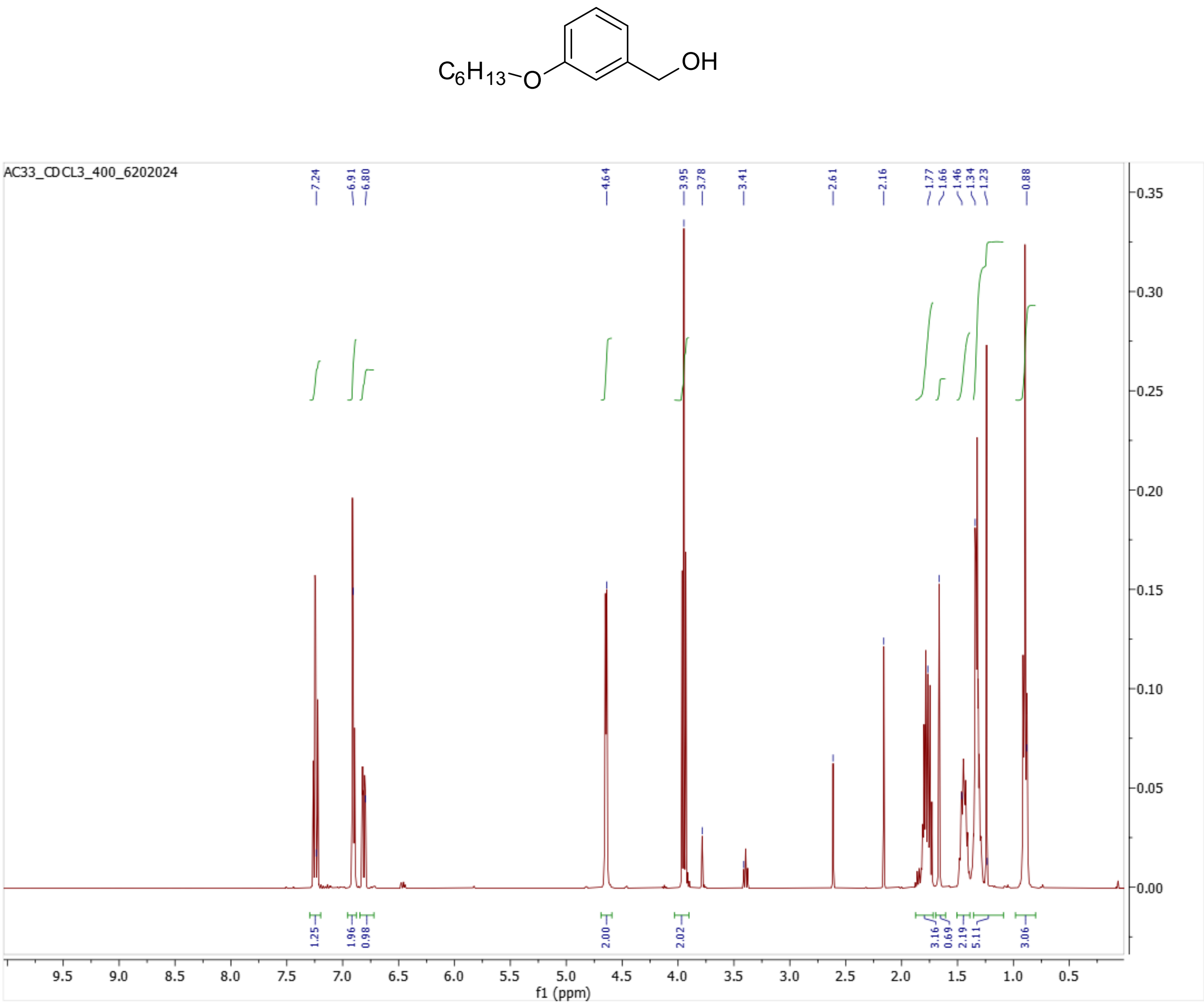

Figure S24: ^{1}H NMR (400 MHz) of **AC33** in $CDCl_3$.

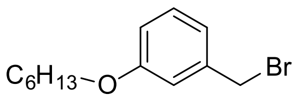

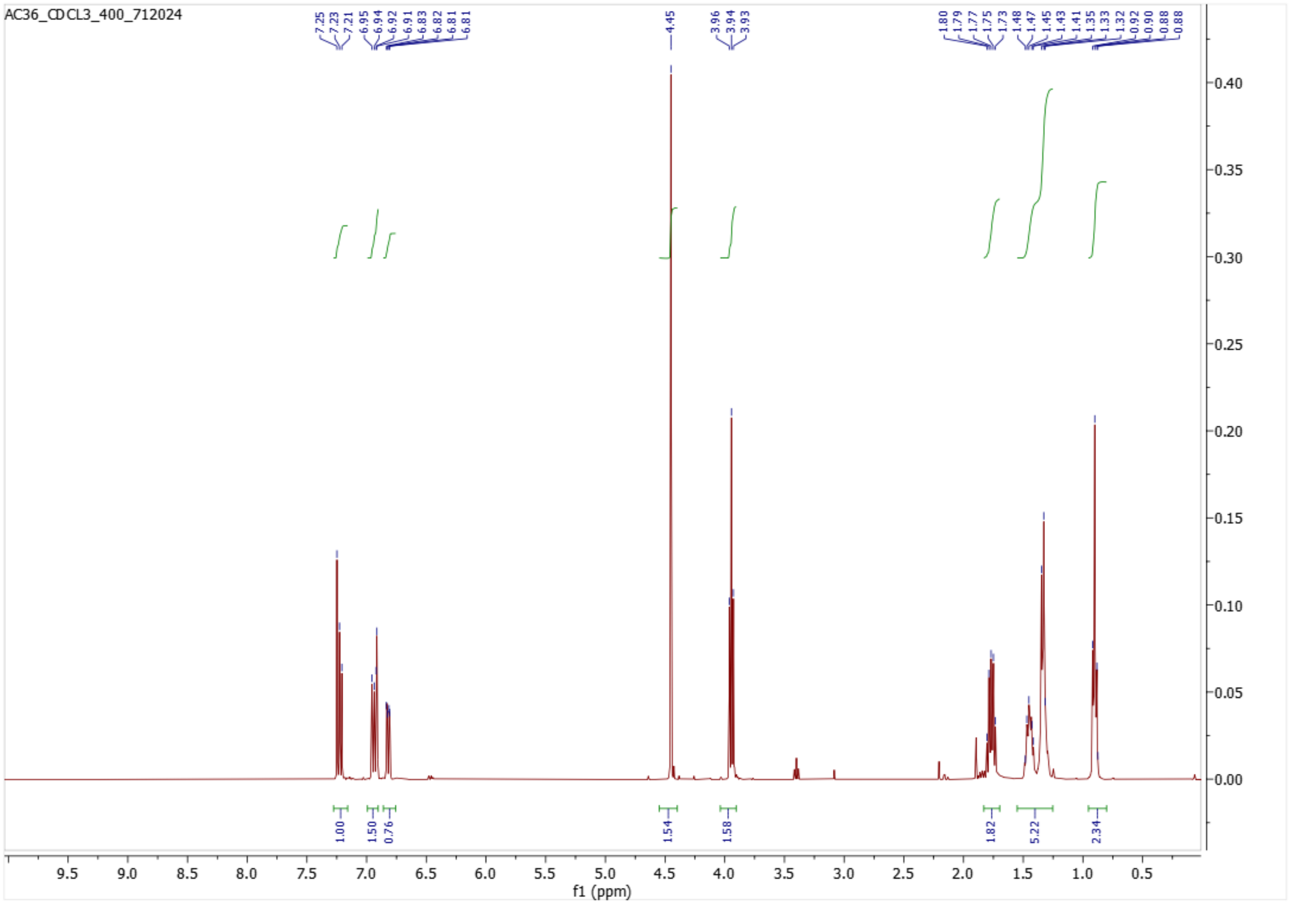

Figure S25: ^{1}H NMR (400 MHz) of **AC36** in $CDCl_3$.

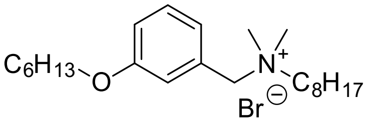

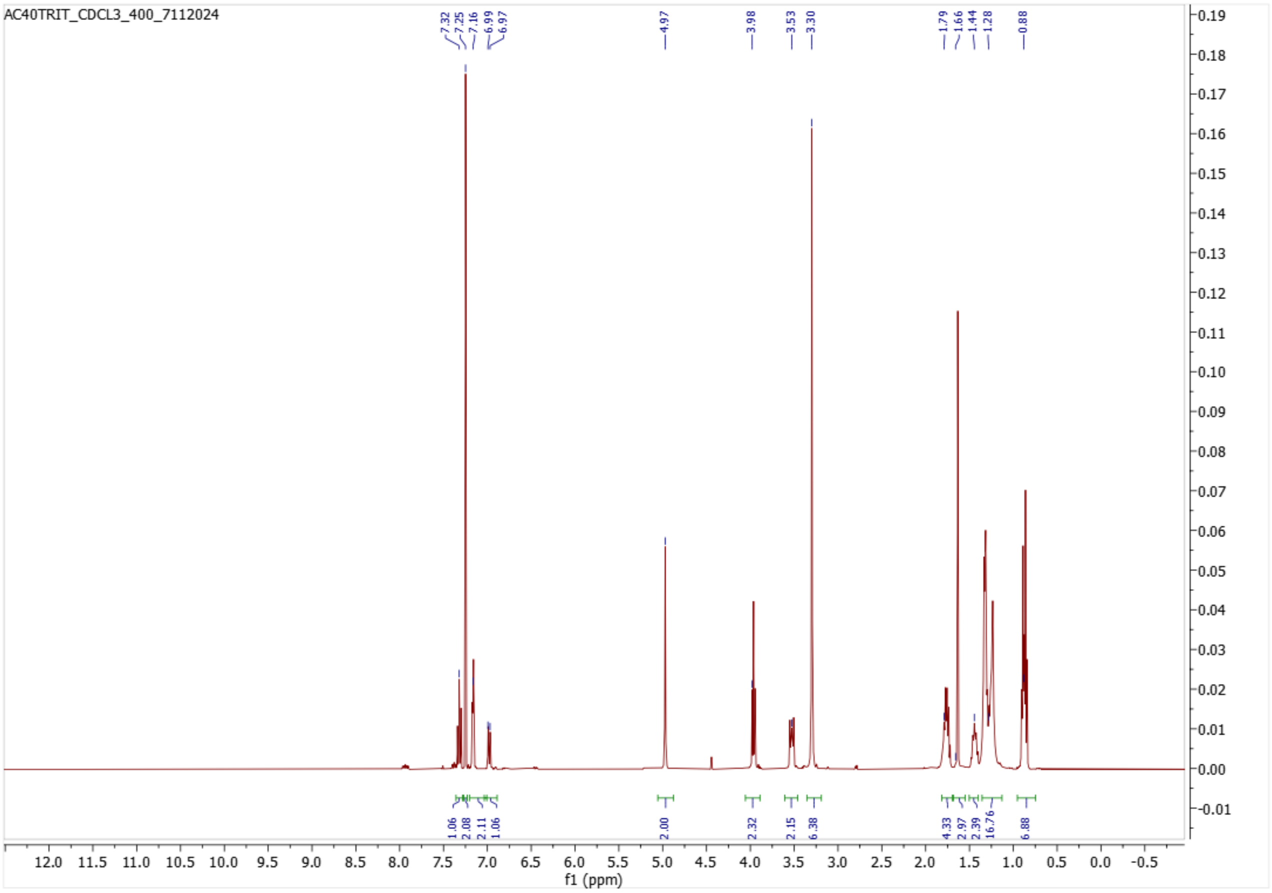

Figure S26: ^{1}H NMR (400 MHz) of **mOC6-Bn-8 (AC40)** in $CDCl_3$.

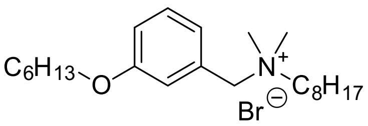

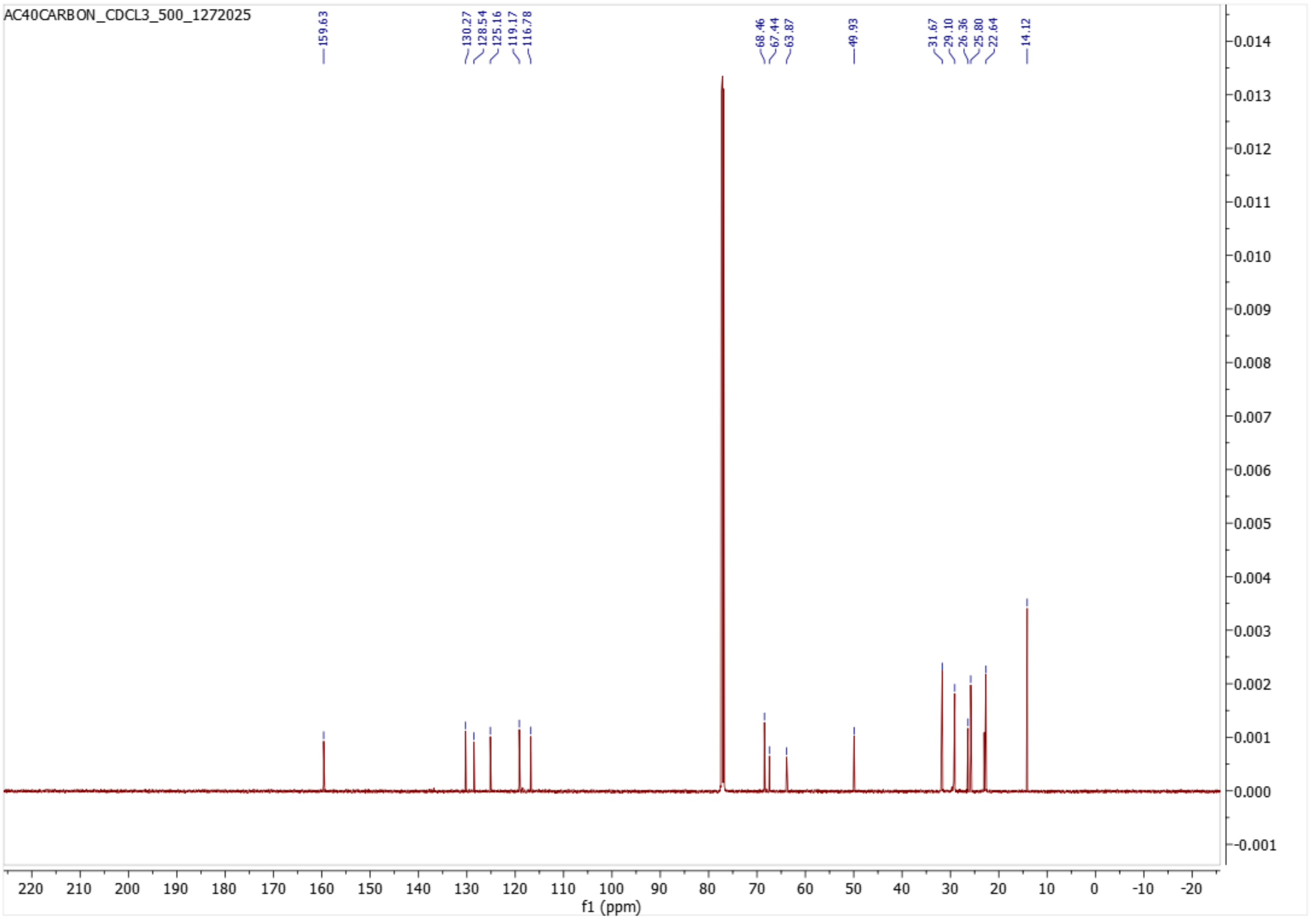

Figure S27: ^{13}C NMR (500 MHz) of **mOC6-Bn-8 (AC40)** in $CDCl_3$.

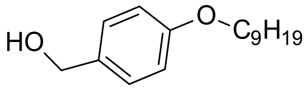

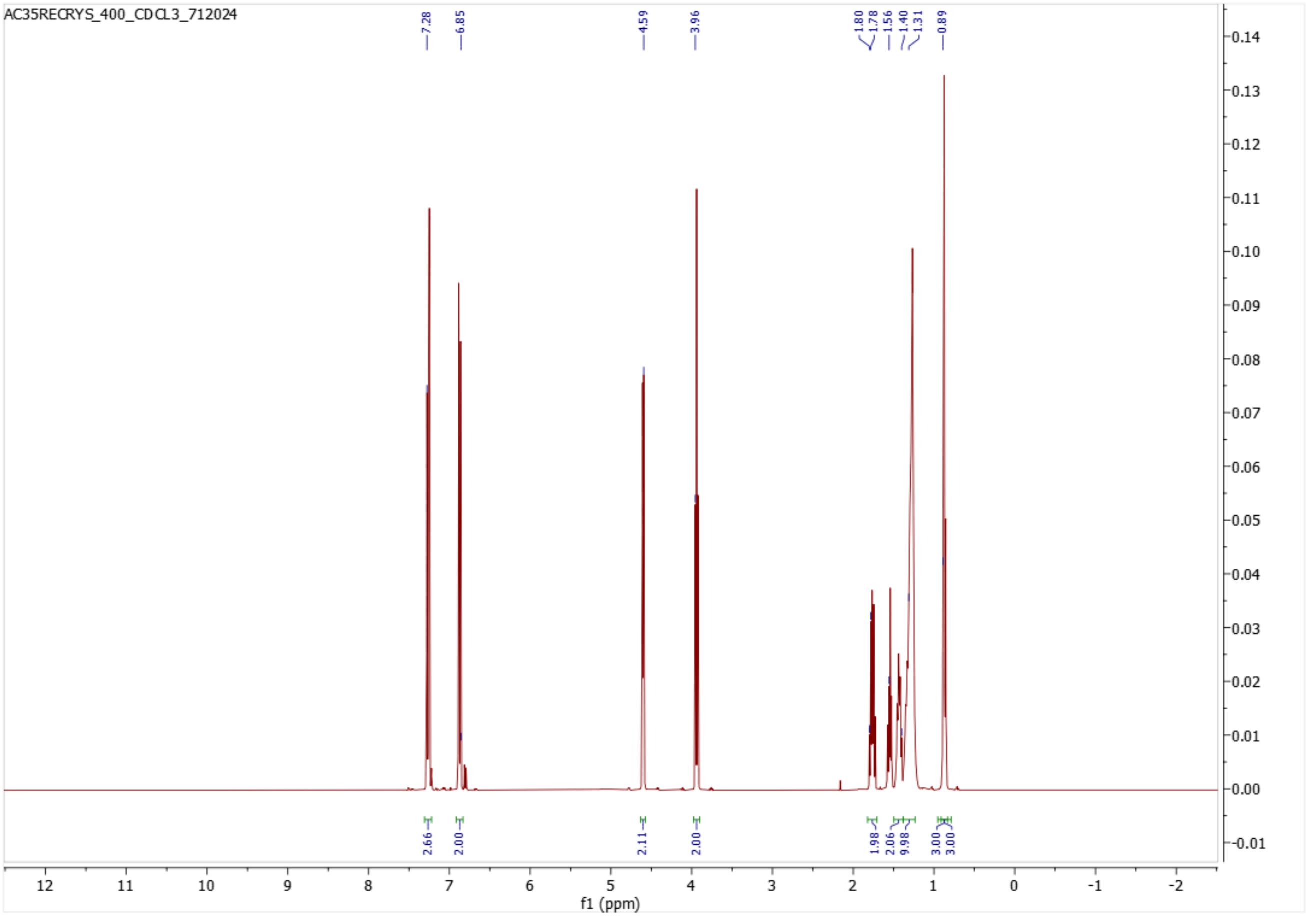

Figure S28: ^{1}H NMR (400 MHz) of **AC35** in $CDCl_3$.

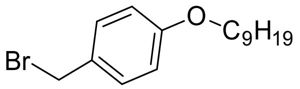

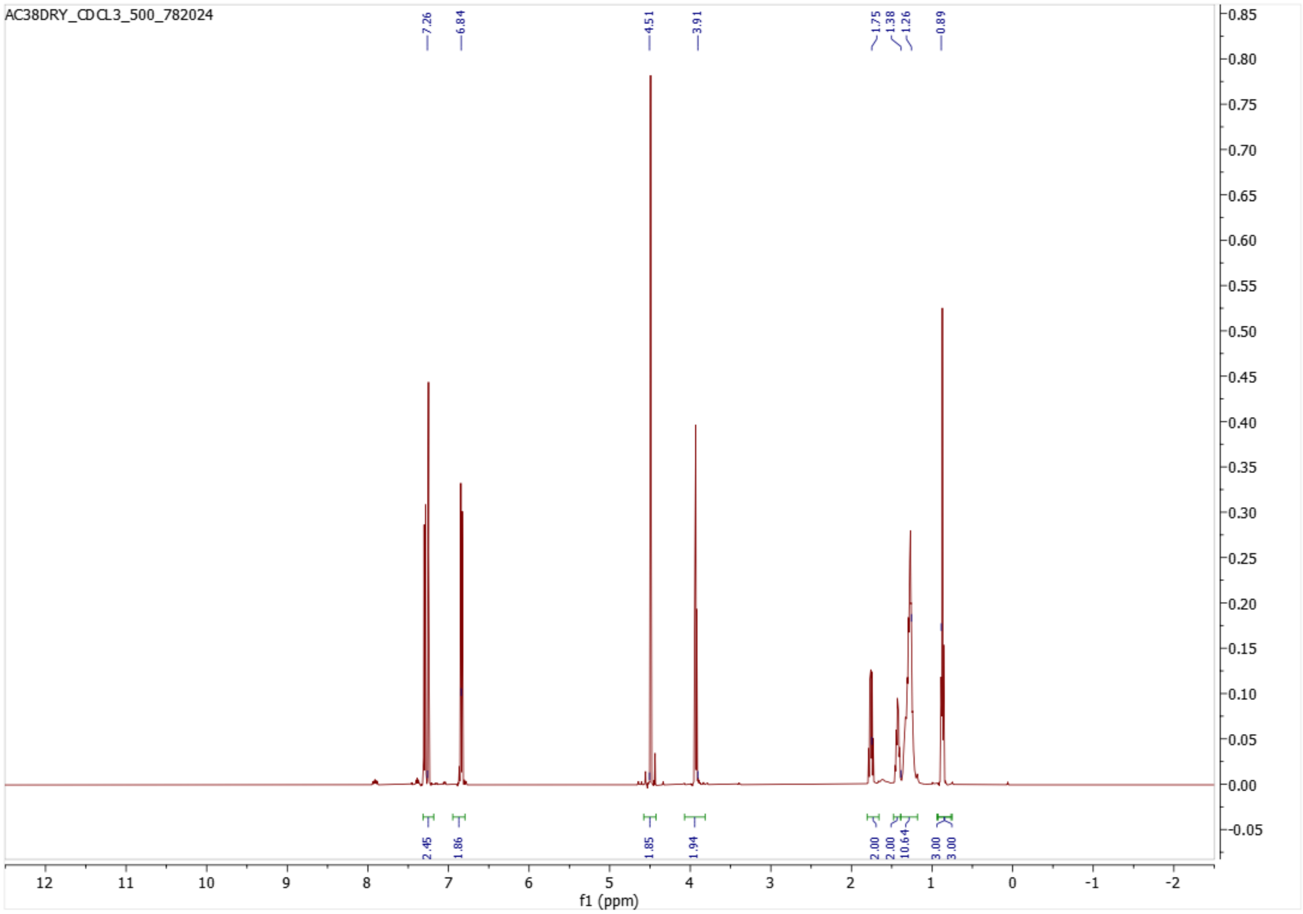

Figure S29: ^{1}H NMR (500 MHz) of **AC38** in $CDCl_3$.

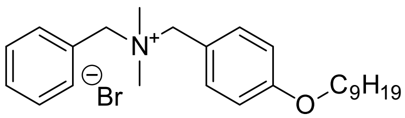

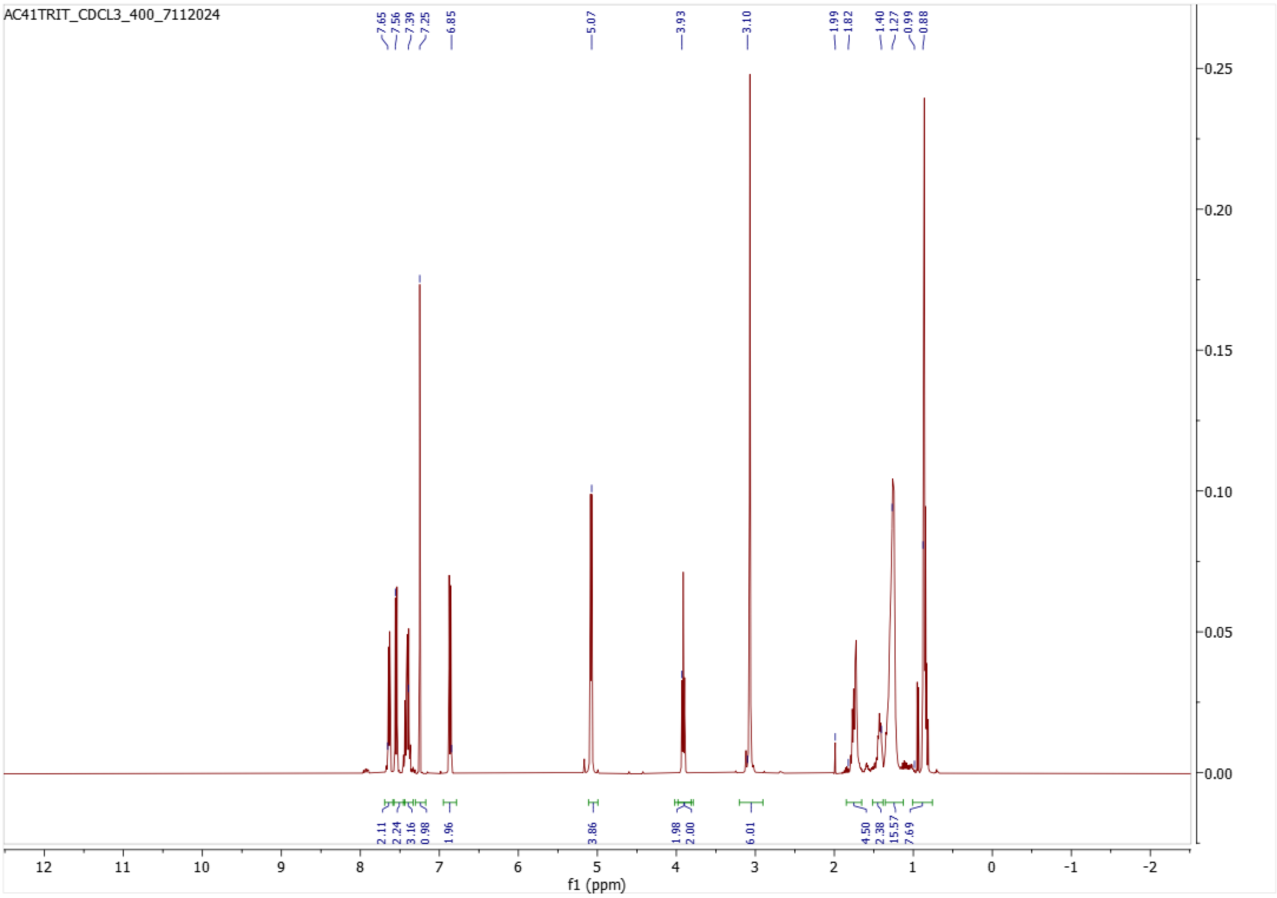

Figure S30: ^{1}H NMR (400 MHz) of **Bn-pOC9-Bn (AC41)** in $CDCl_3$.

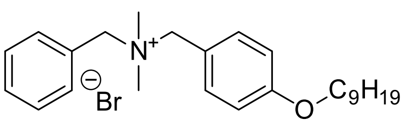

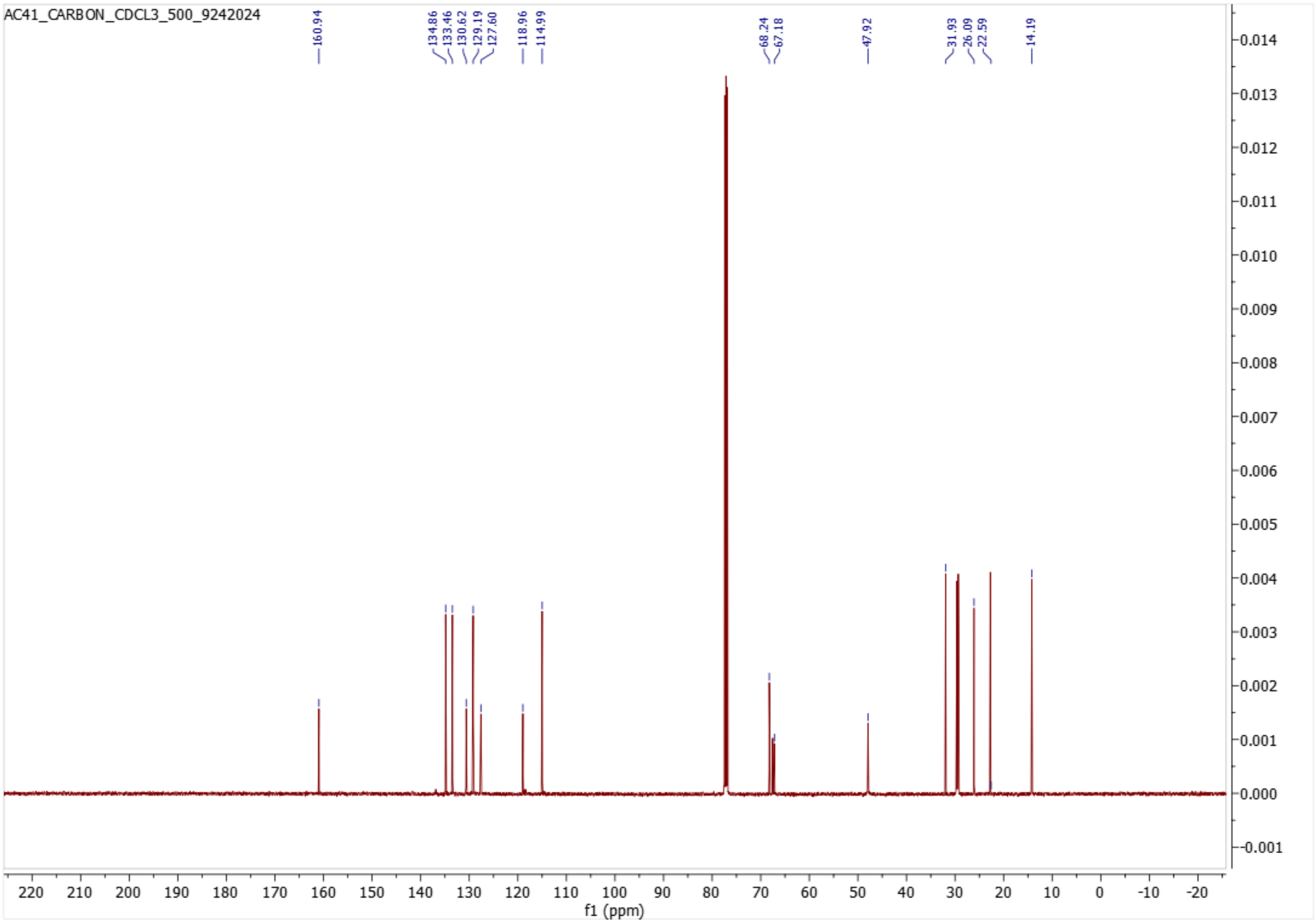

Figure S31: ^{13}C NMR (500 MHz) of **Bn-pOC9-Bn (AC41)** in $CDCl_3$.

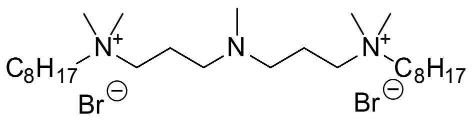

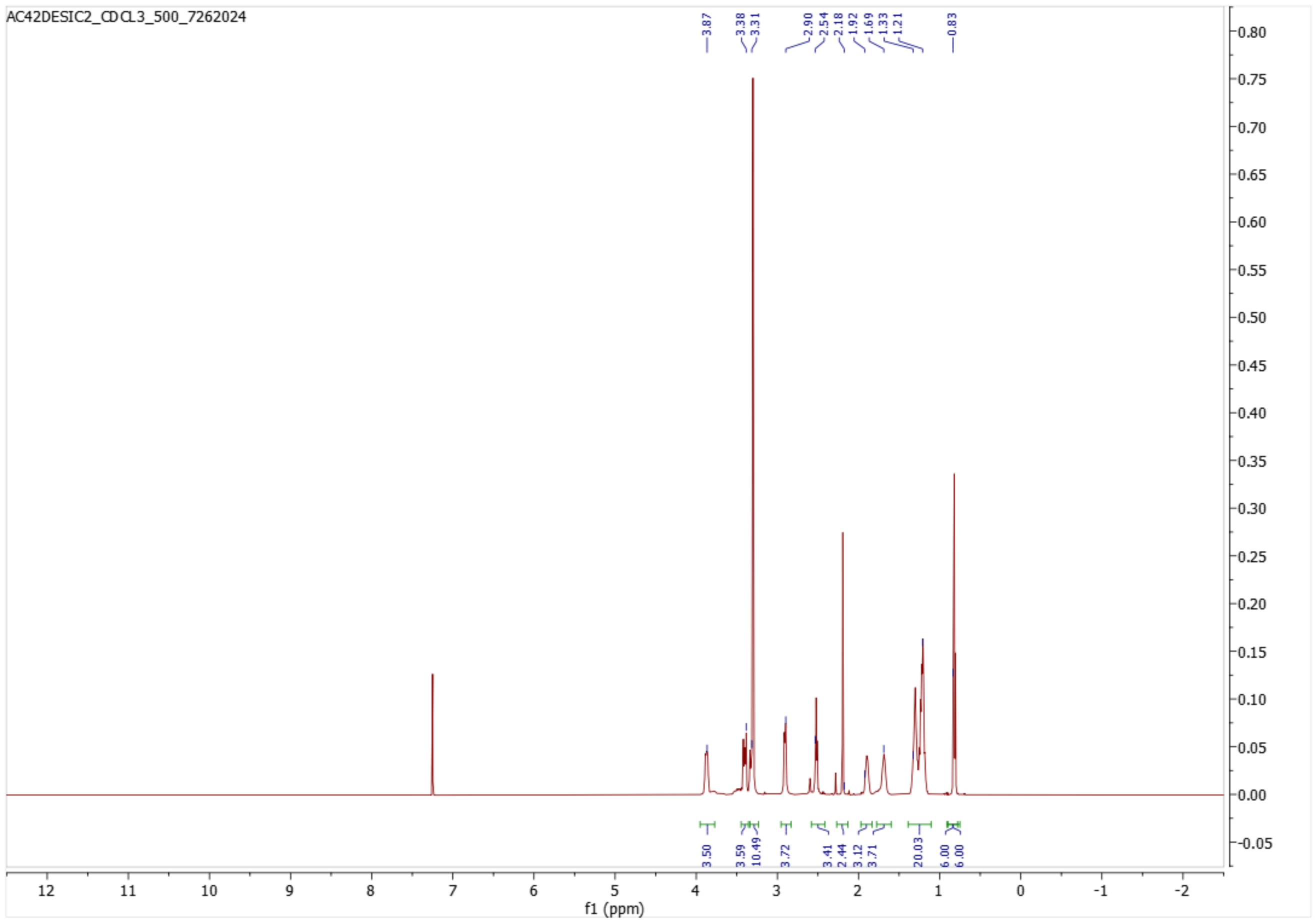

Figure S32: ^{1}H NMR (500 MHz) of **8(3)0(3)8 (AC42)** in $CDCl_3$.

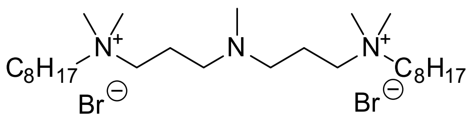

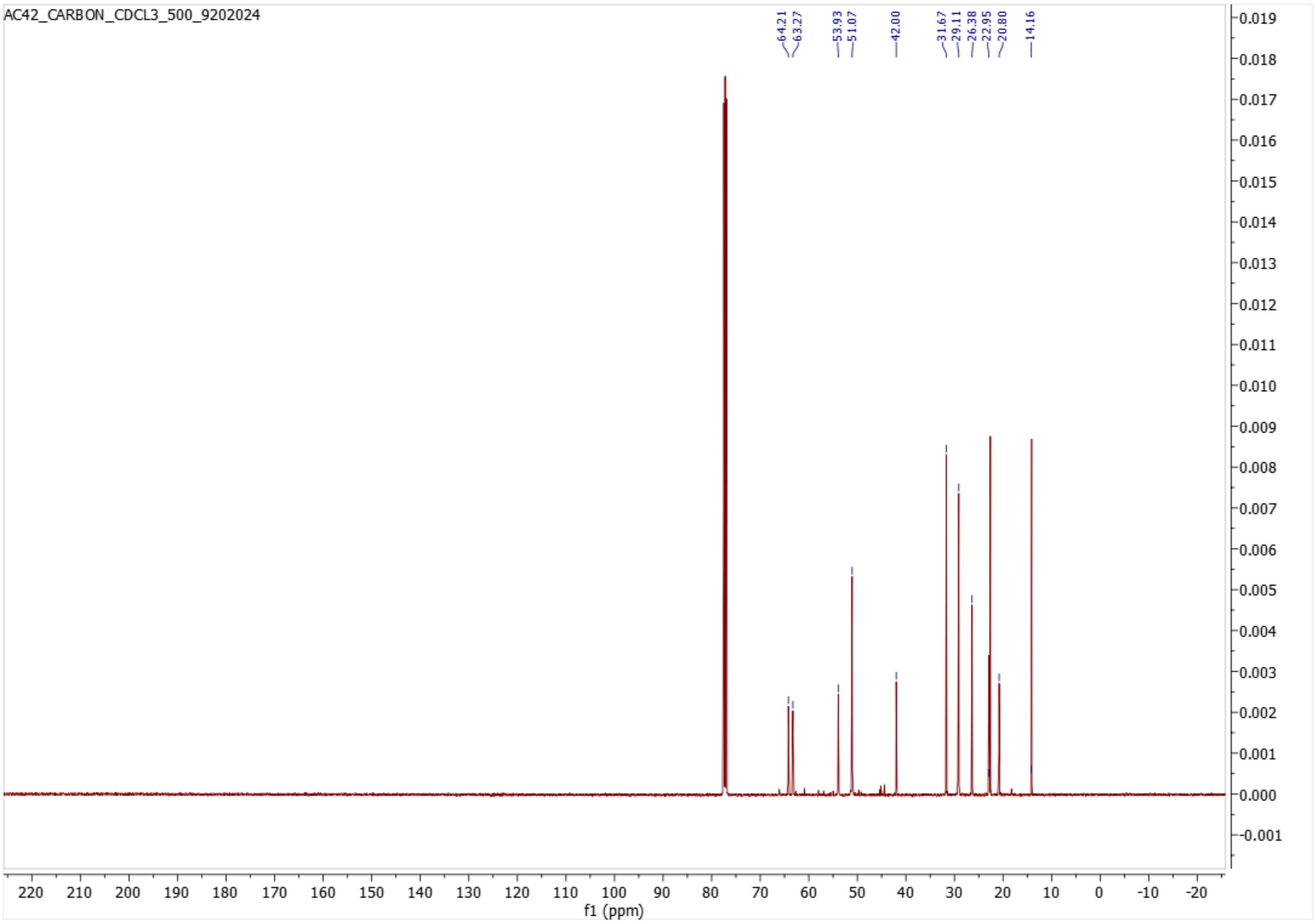

Figure S33: ^{13}C NMR (500 MHz) of **8(3)0(3)8 (AC42)** in $CDCl_3$.

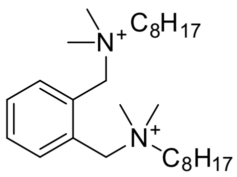

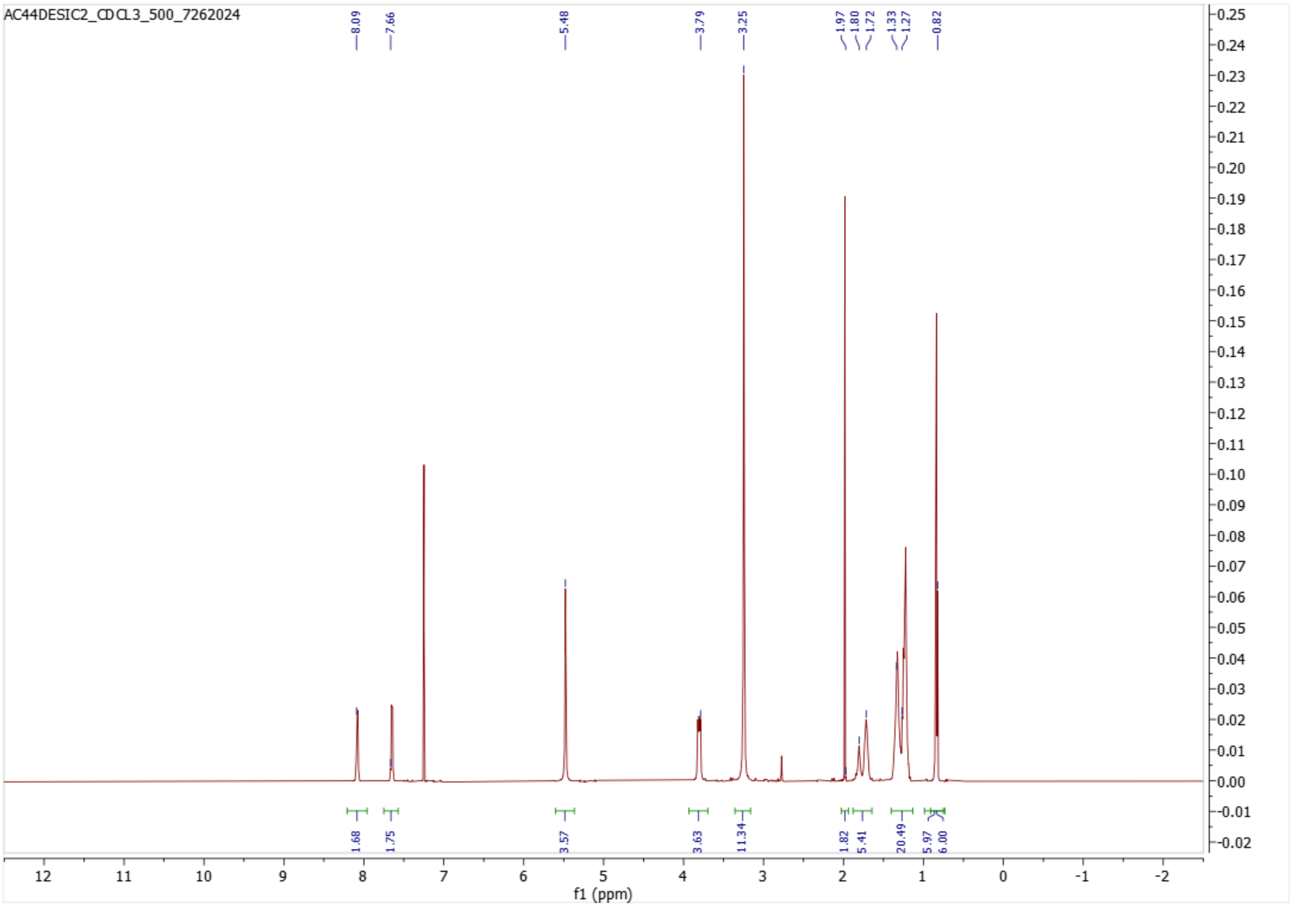

Figure S34: ^{1}H NMR (500 MHz) of **oX-8,8 (AC44)** in $CDCl_3$.

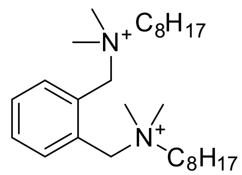

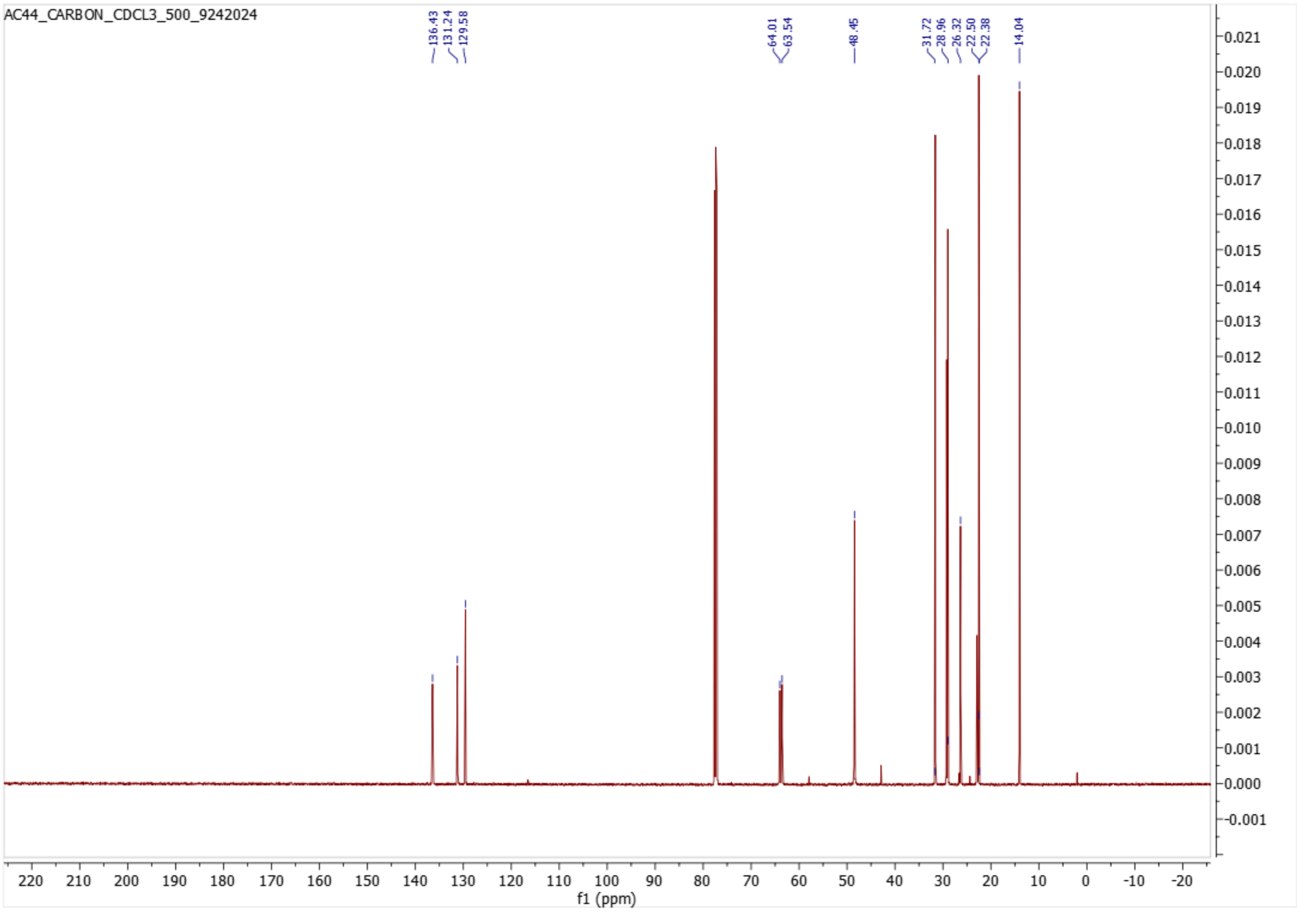

Figure S35: ^{13}C NMR (500 MHz) of **oX-8,8 (AC44)** in $CDCl_3$.

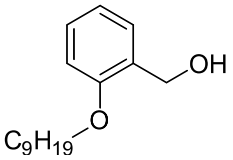

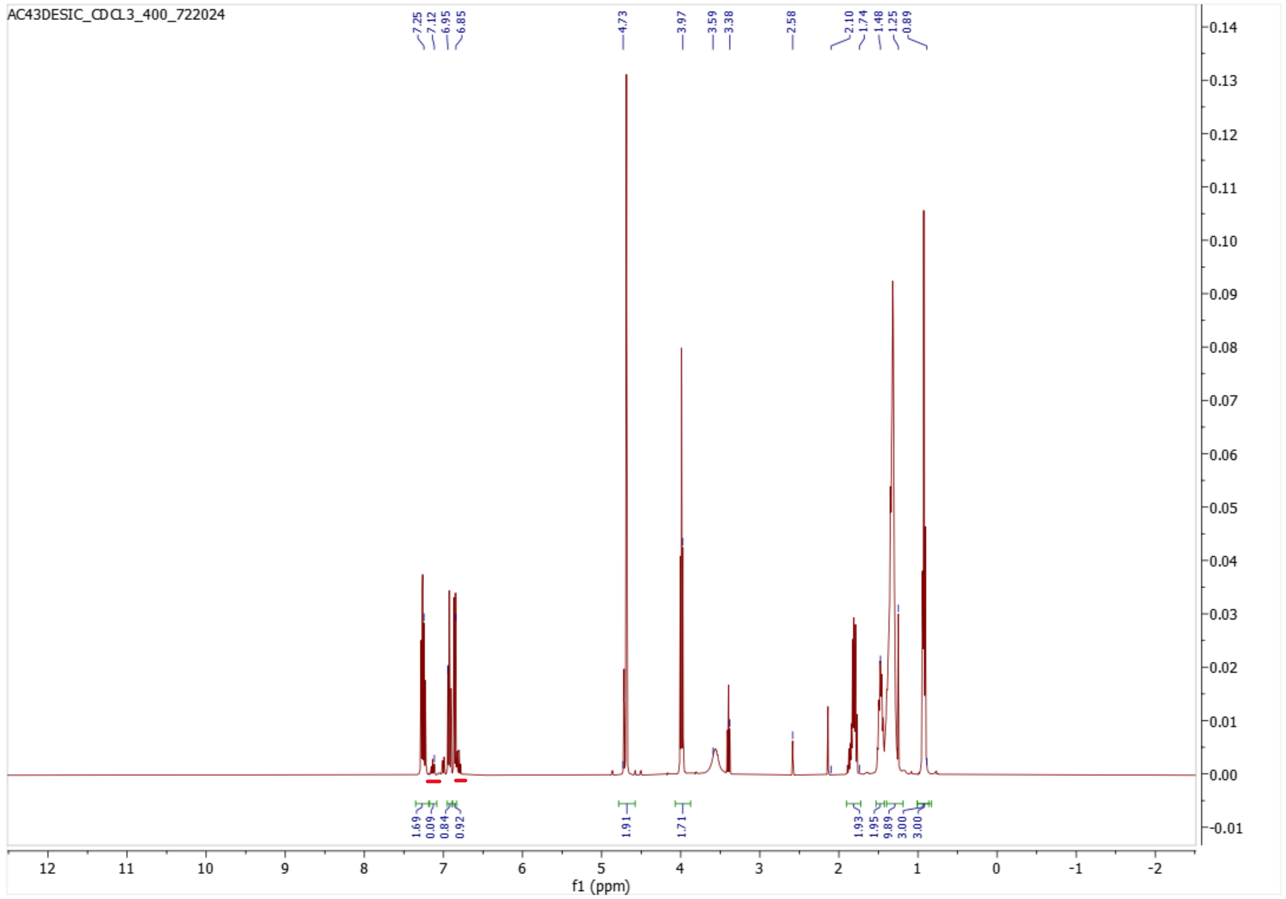

Figure S36: ^{1}H NMR (400 MHz) of **AC43** in $CDCl_3$.

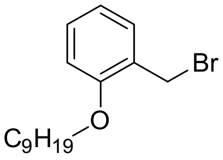

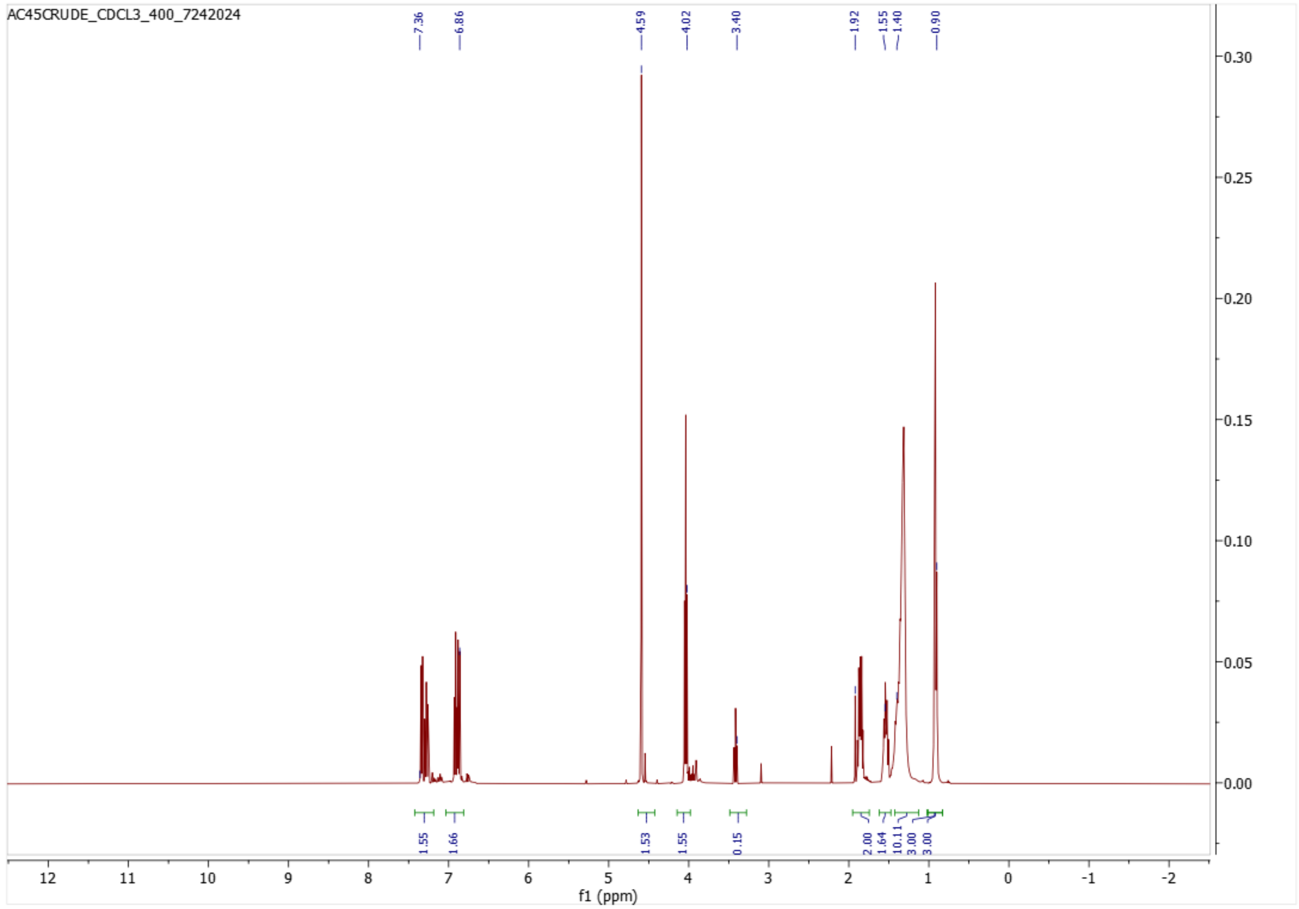

Figure S37: ^{1}H NMR (400 MHz) of **AC45** in $CDCl_3$.

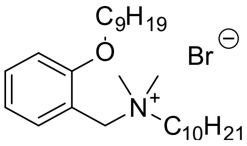

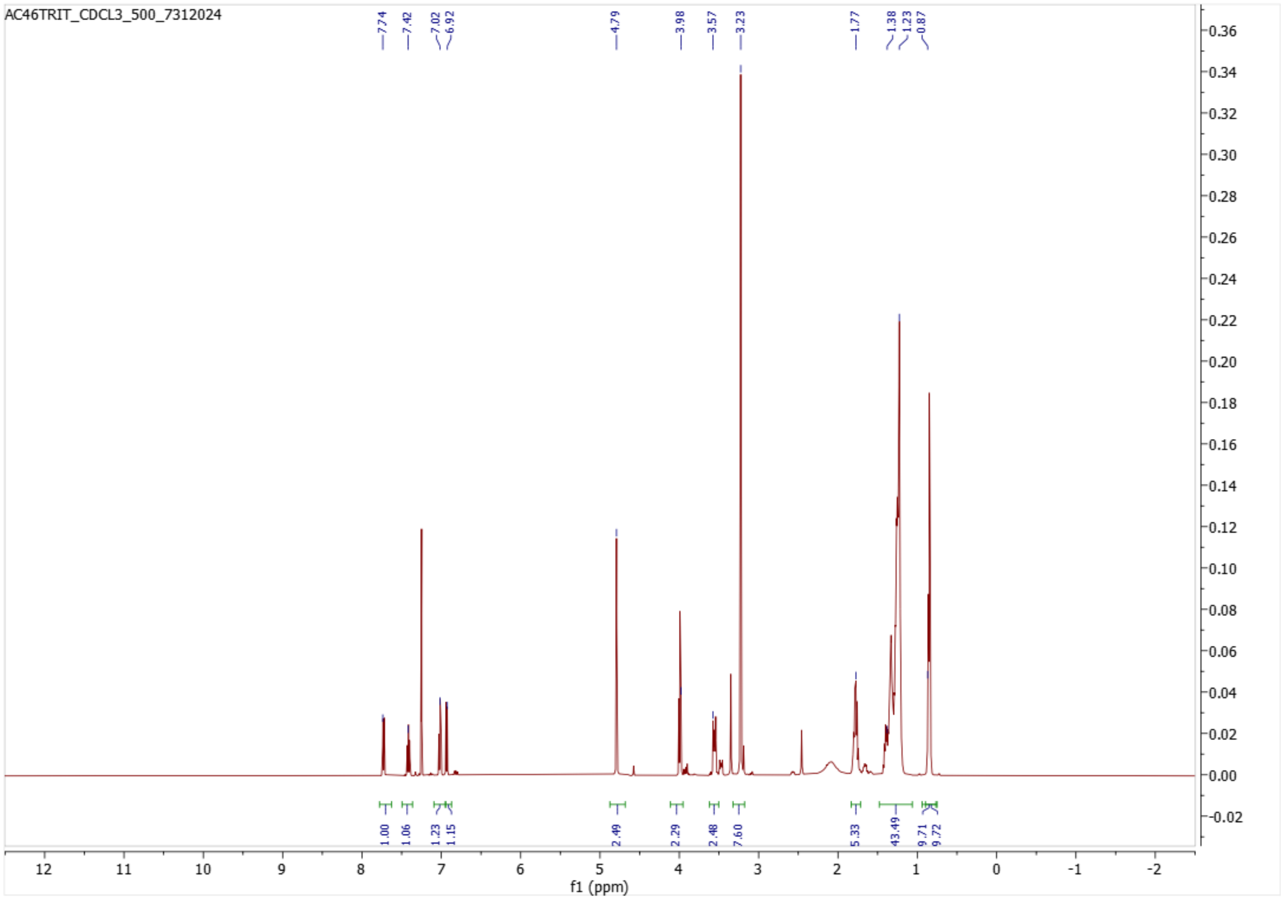

Figure S38: ^{1}H NMR (500 MHz) of **oOC9-Bn-10 (AC46)** in $CDCl_3$.

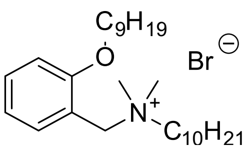

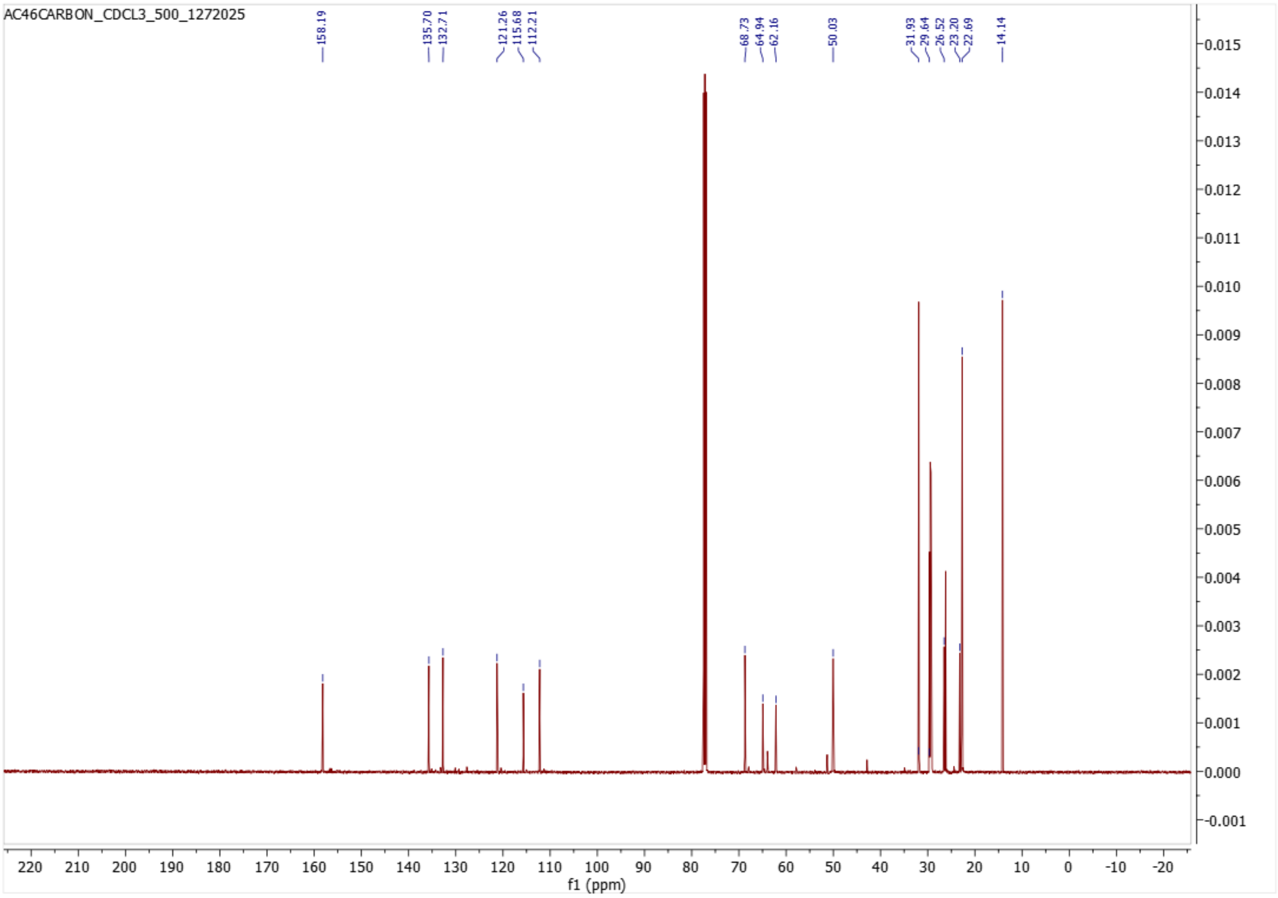

Figure S39: ^{13}C NMR (500 MHz) of **oOC9-Bn-10 (AC46)** in $CDCl_3$.

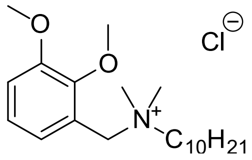

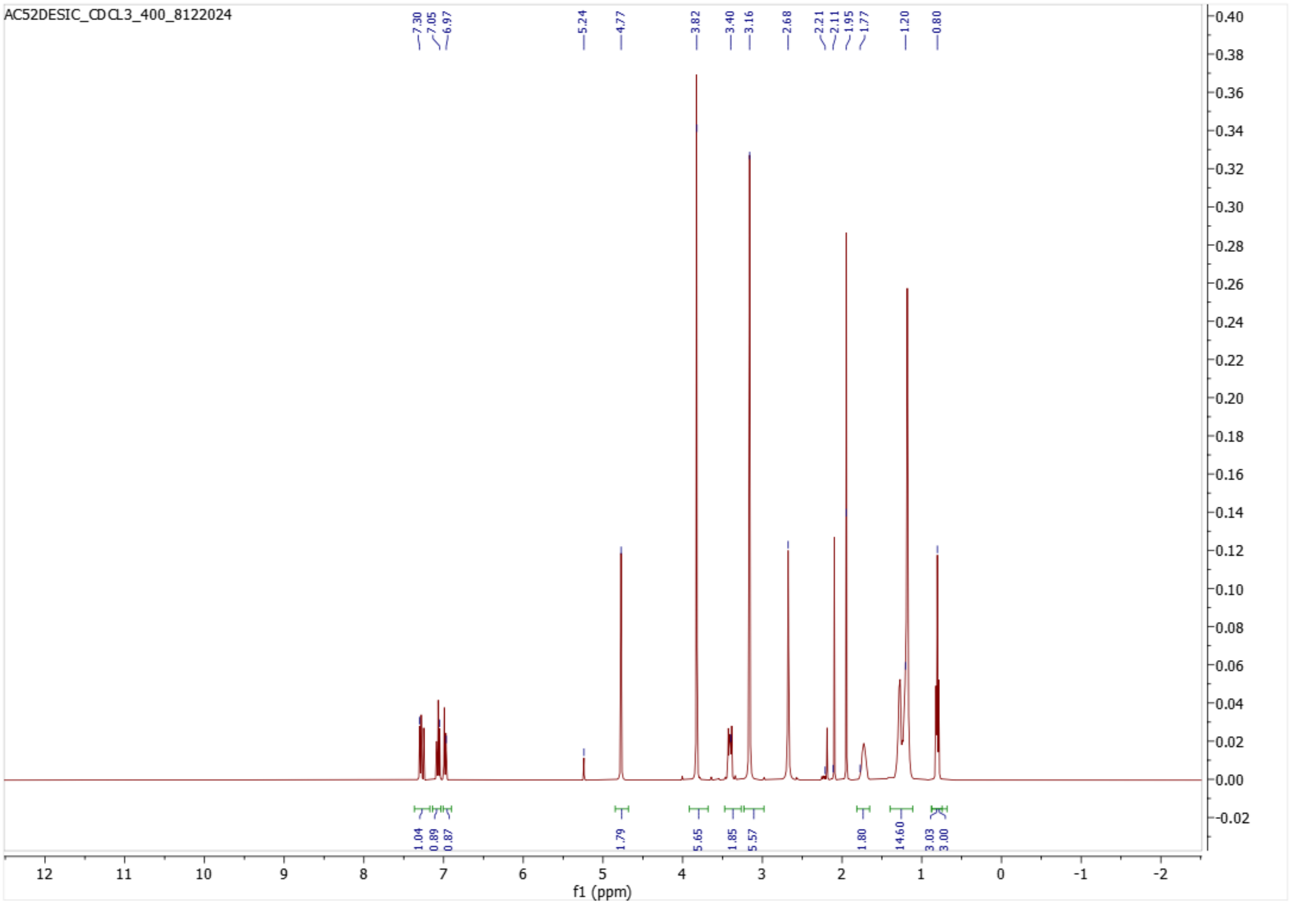

Figure S40: ^{1}H NMR (400 MHz) of **2,3methoxy-Bn10 (AC52)** in $CDCl_3$.

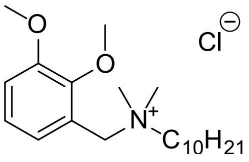

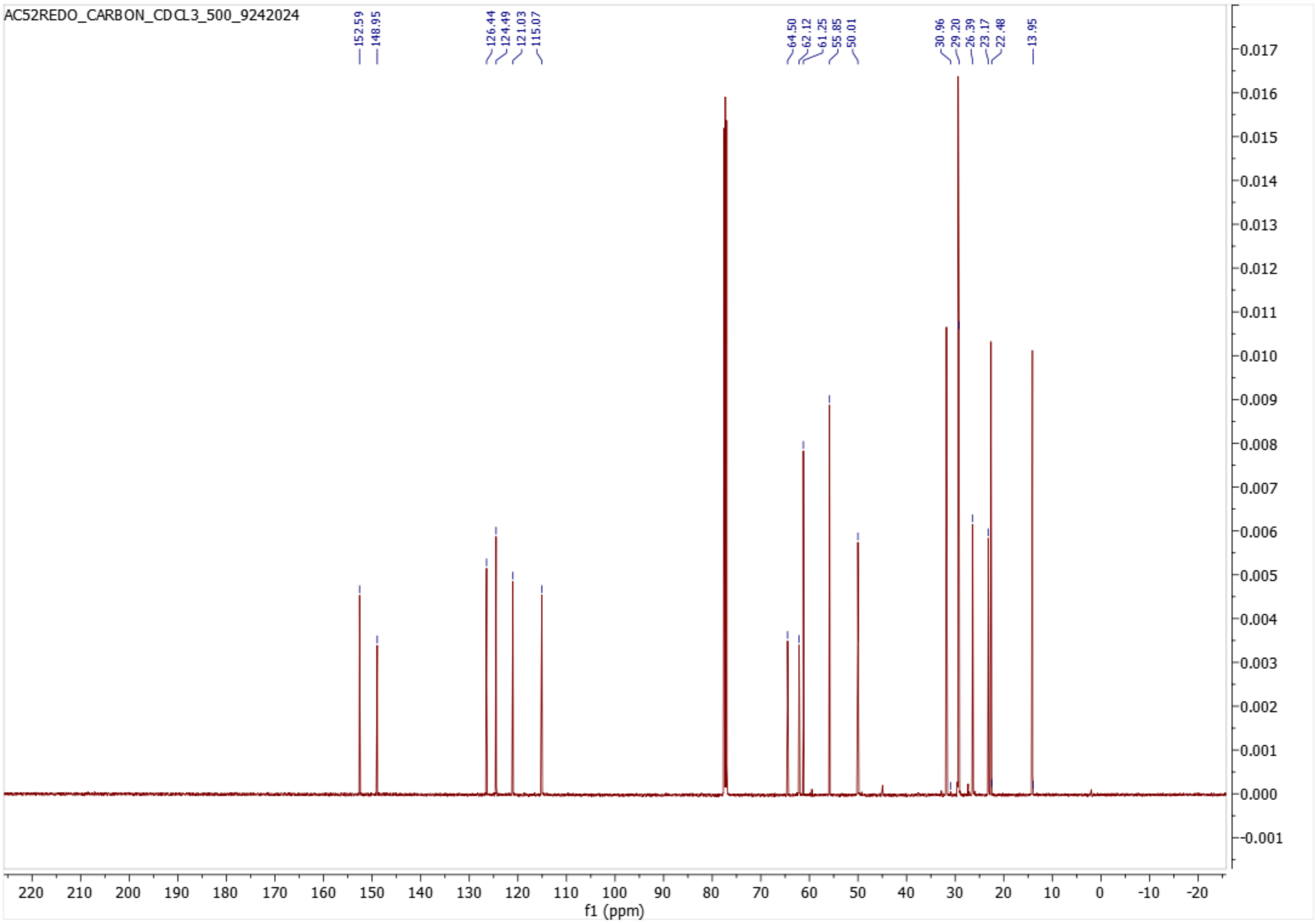

Figure S41: ^{13}C NMR (500 MHz) of **2,3methoxy-Bn10 (AC52)** in $CDCl_3$.

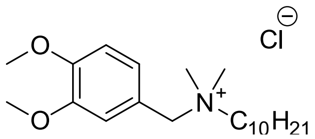

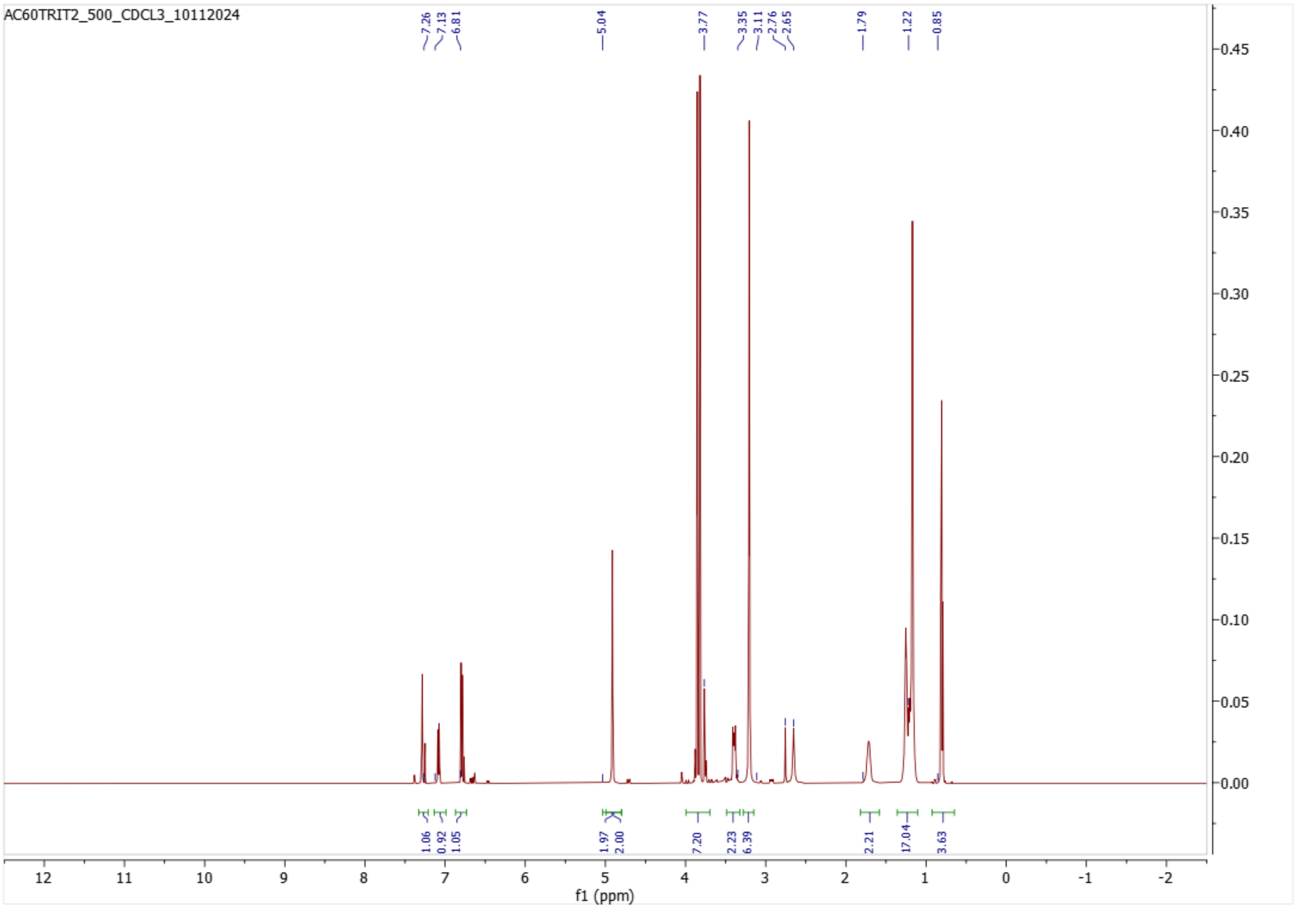

Figure S42: ^{1}H NMR (500 MHz) of **3,4methoxy-Bn10 (AC60)** in $CDCl_3$.

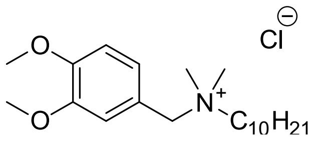

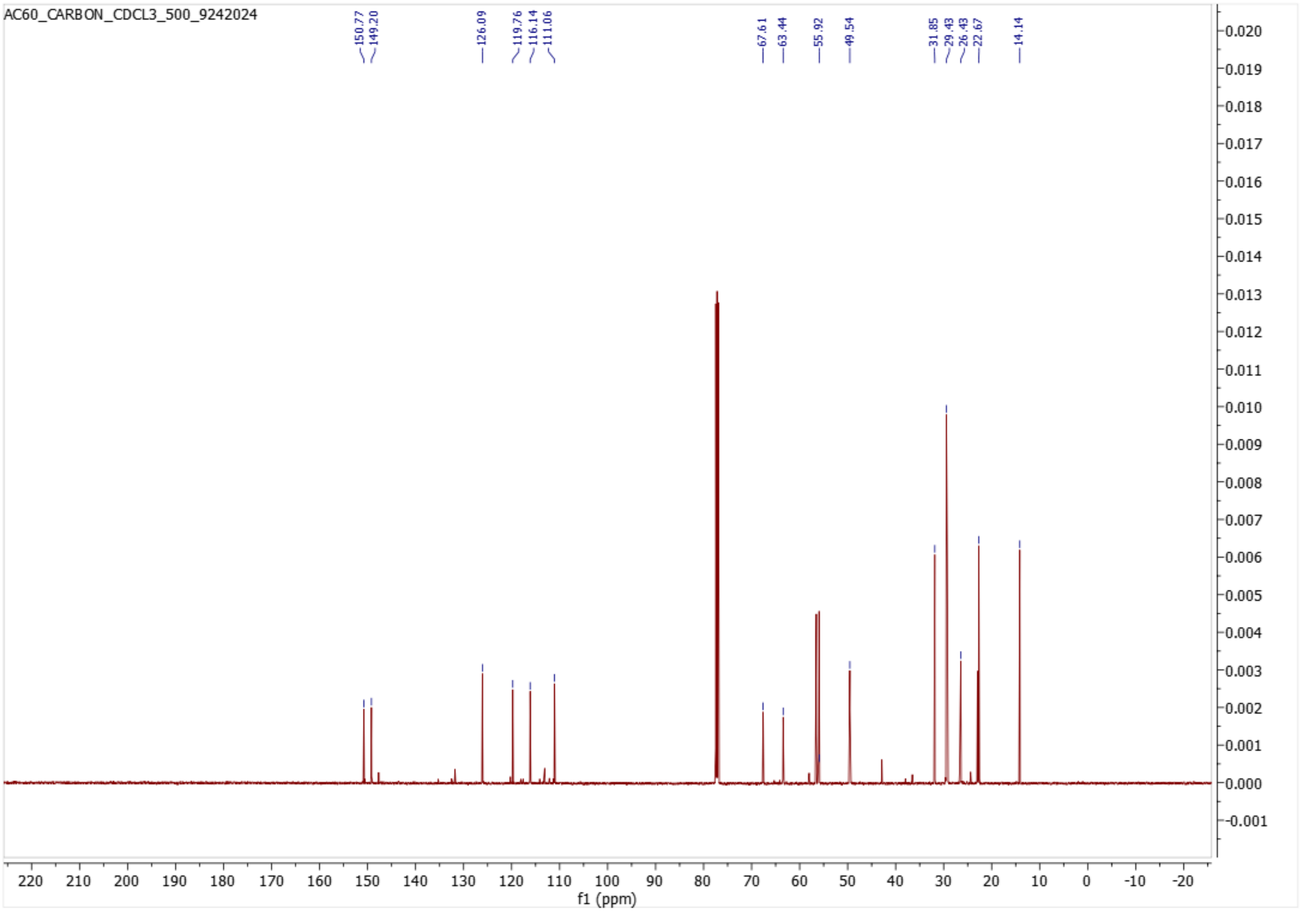

Figure S43: ^{13}C NMR (500 MHz) of **3,4methoxy-Bn10 (AC60)** in $CDCl_3$.

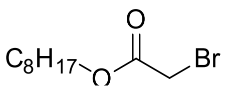

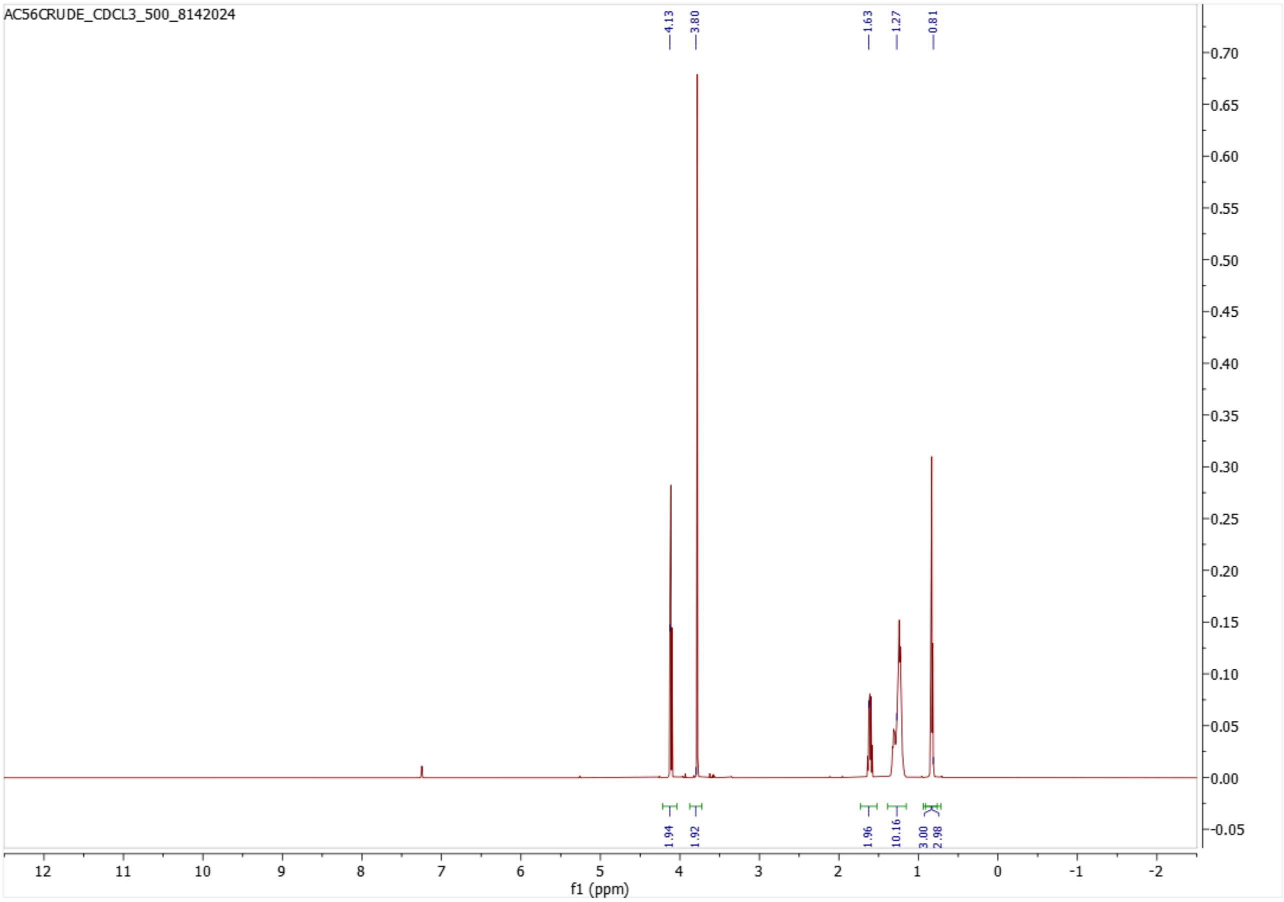

Figure S44: ^{1}H NMR (500 MHz) of **AC56** in $CDCl_3$.

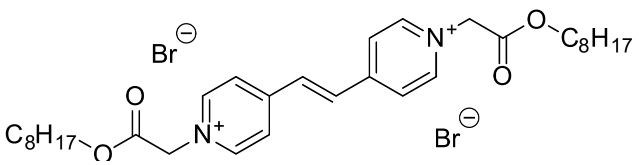

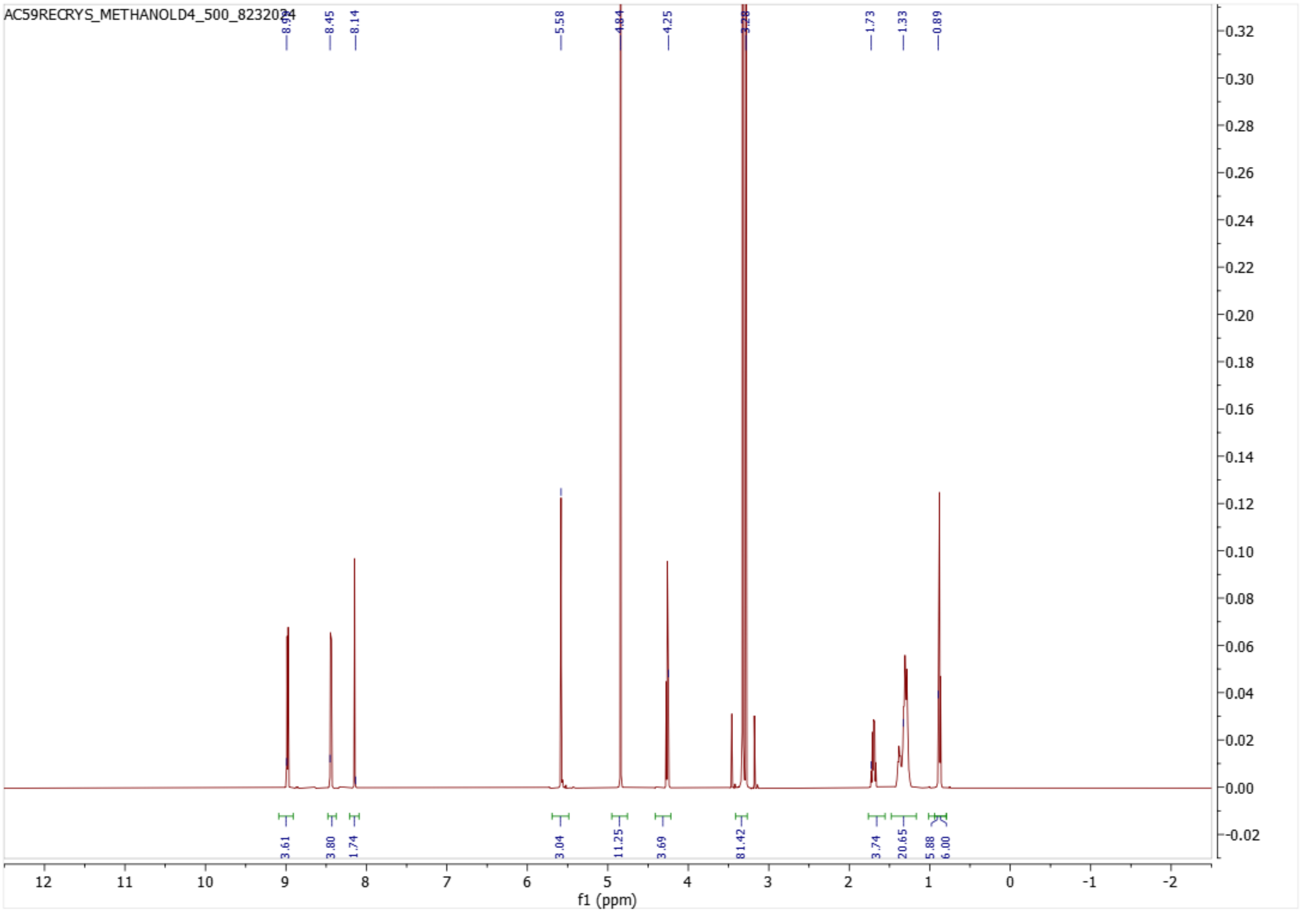

Figure S45: ^{1}H NMR (500 MHz) of **DPE-11E,11E (AC59)** in CD_3OD.

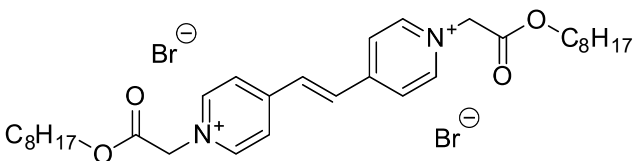

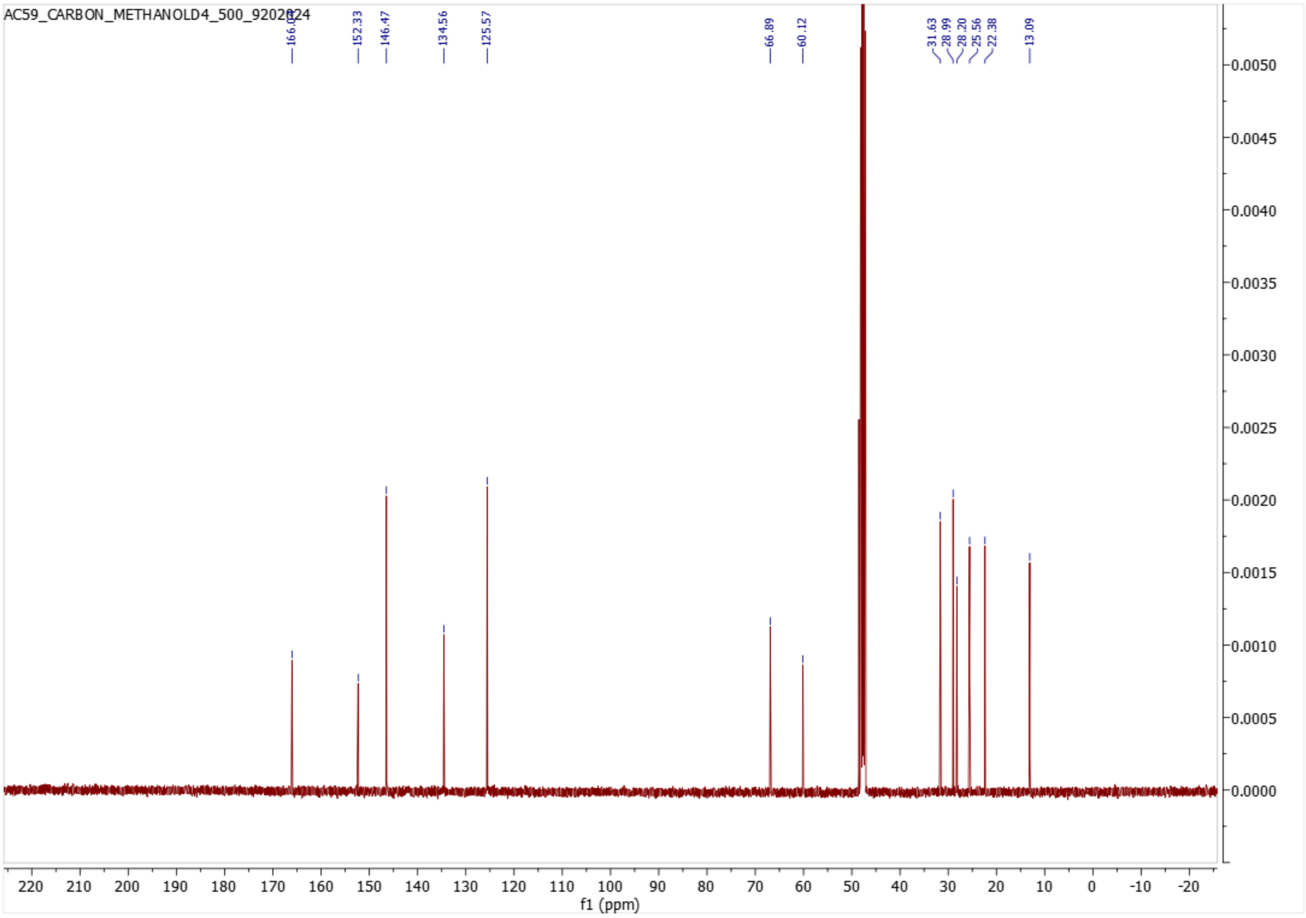

Figure S46: ^{13}C NMR (500 MHz) of **DPE-11E,11E** in CD_3OD.

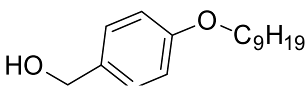

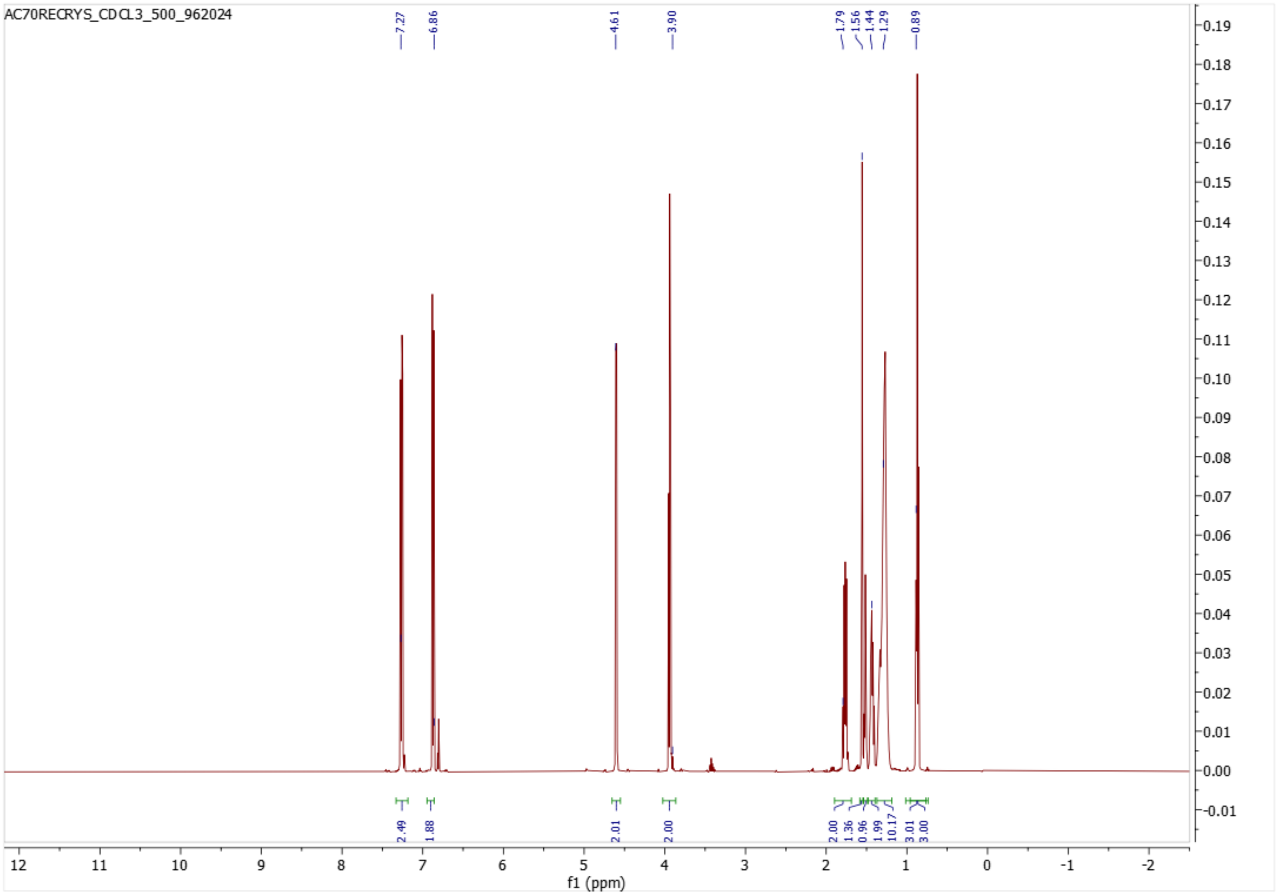

Figure S47: ^{1}H NMR (500 MHz) of **AC70** in $CDCl_3$.

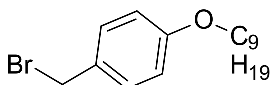

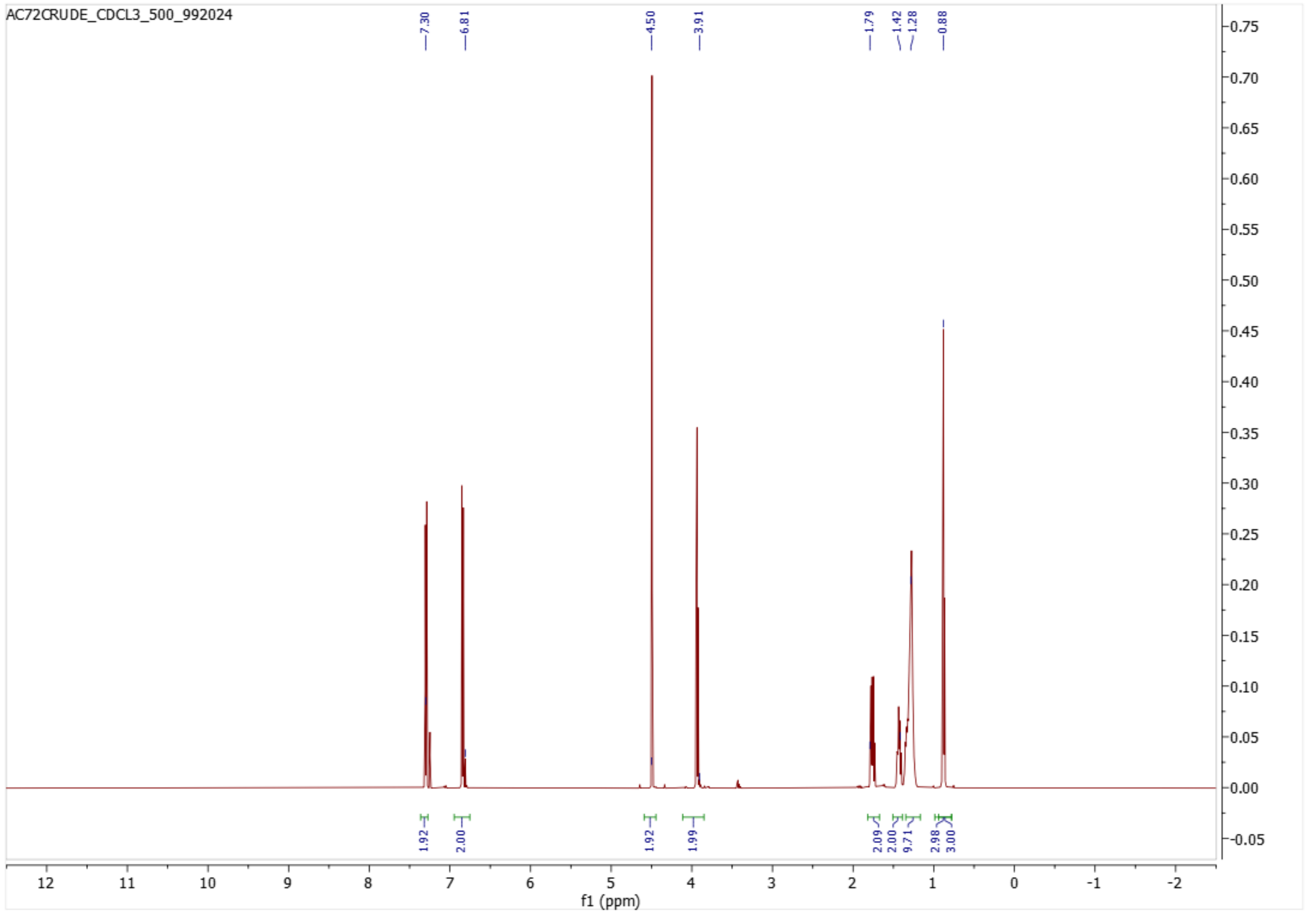

Figure S48: ^{1}H NMR (500 MHz) of **AC72** in $CDCl_3$.

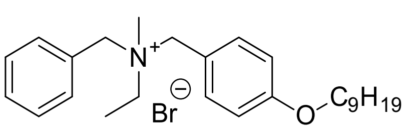

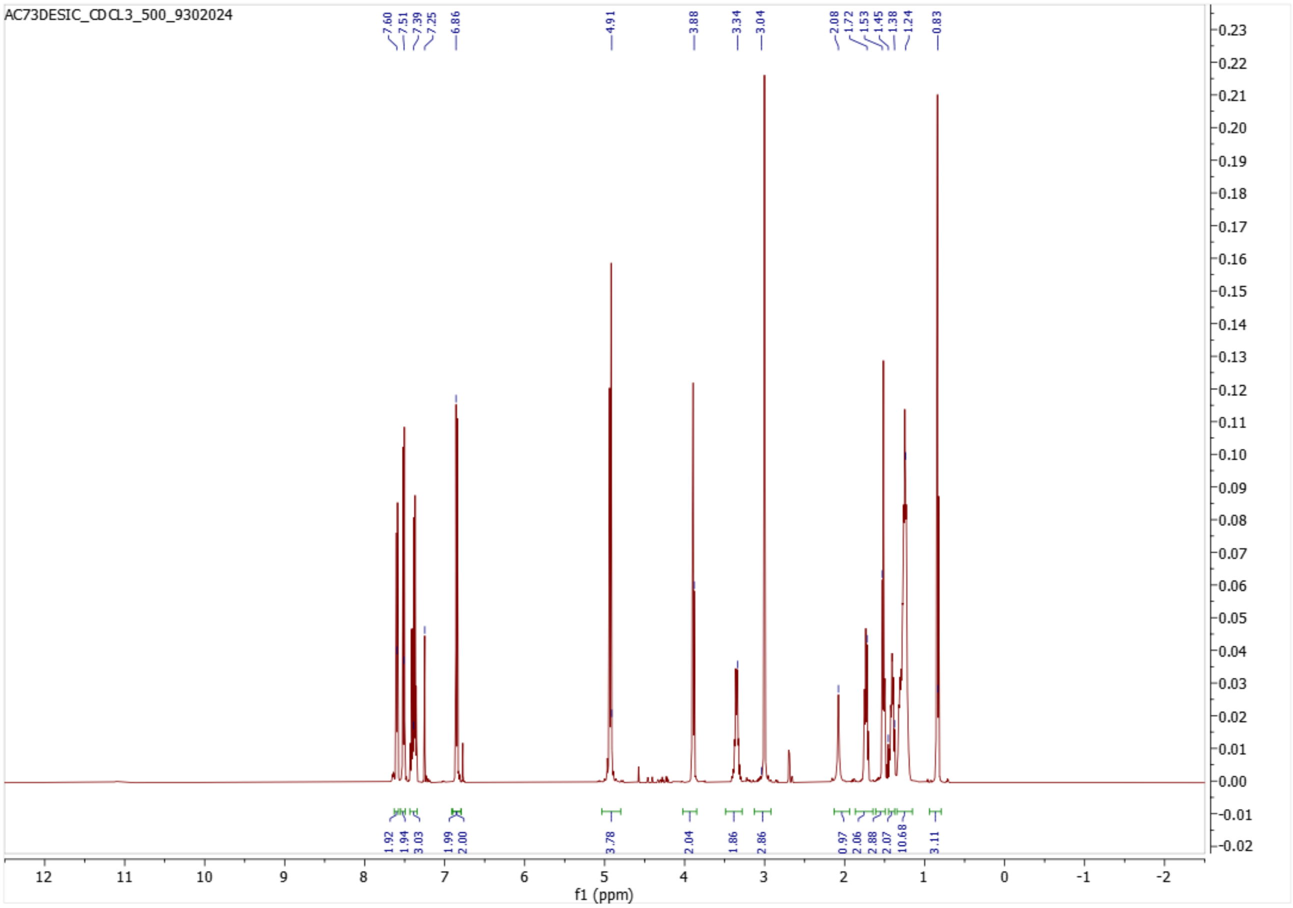

Figure S49: ^{1}H NMR (500 MHz) of **EtBn-pOC9Bn (AC73)** in $CDCl_3$.

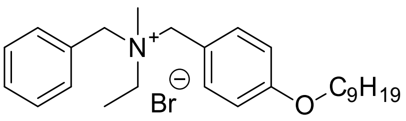

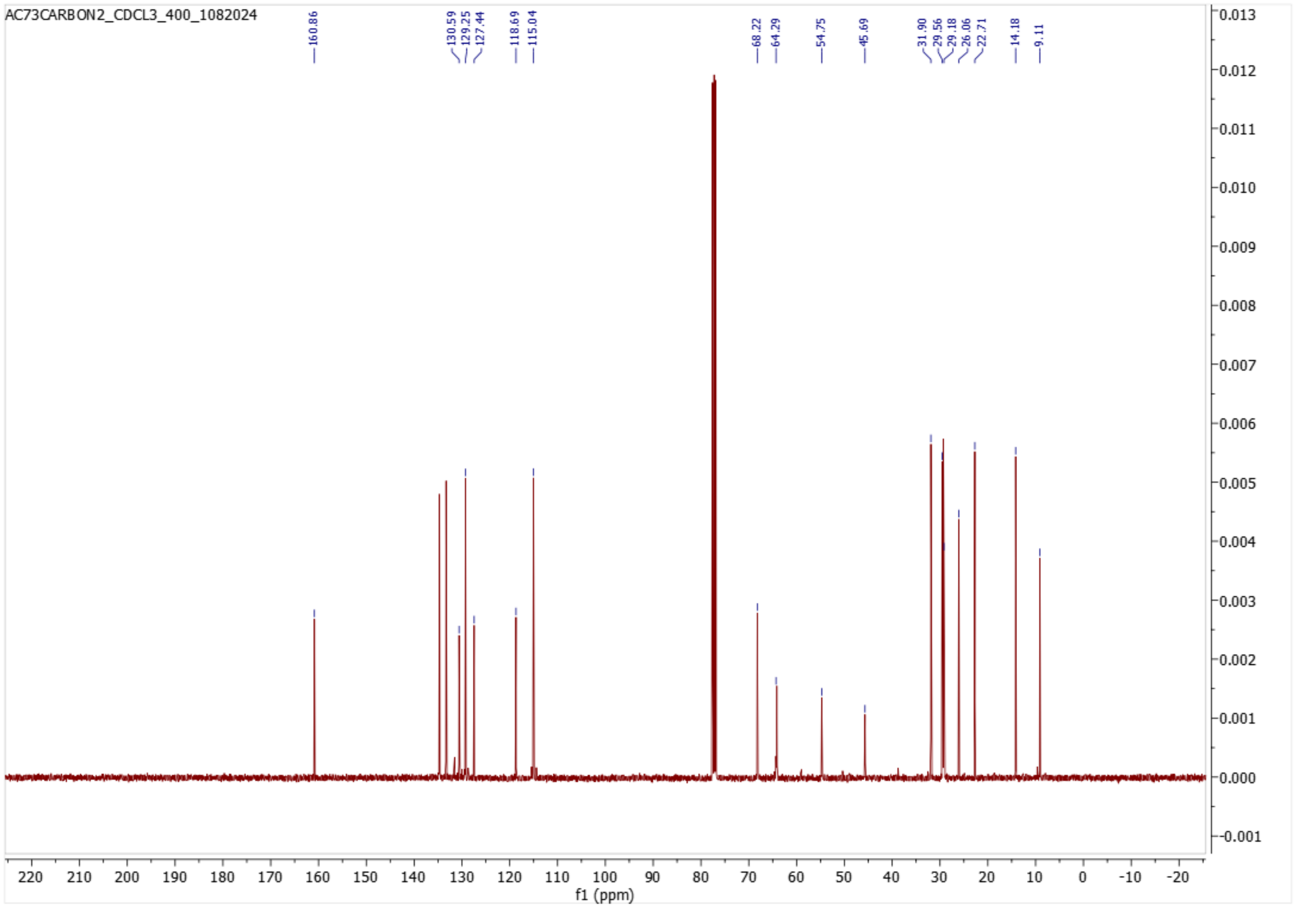

Figure S50: ^{13}C NMR (400 MHz) of **EtBn-pOC9Bn (AC73)** in $CDCl_3$.

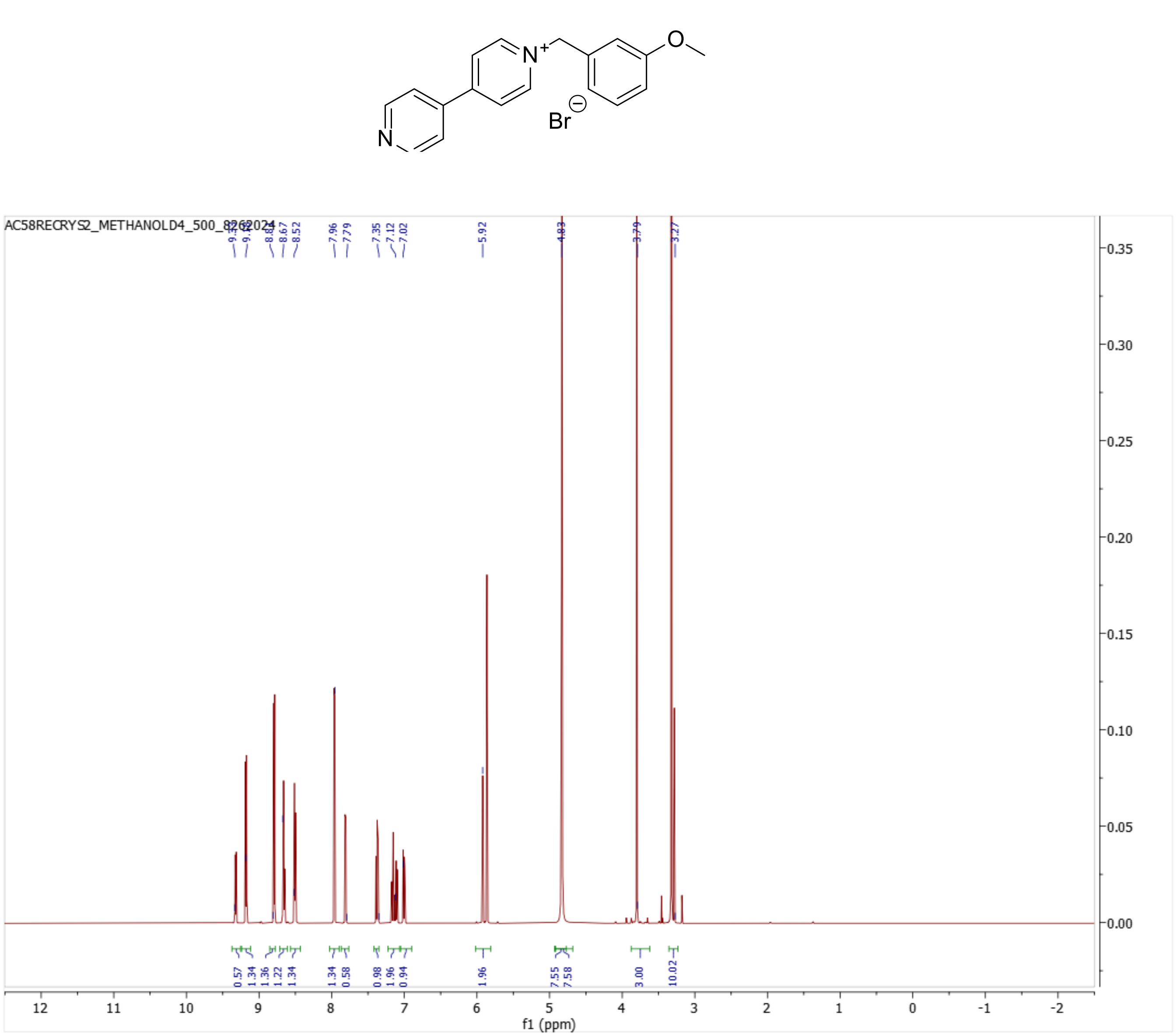

Figure S51: ^{1}H NMR (500 MHz) of **AC58** in CD_3OD.

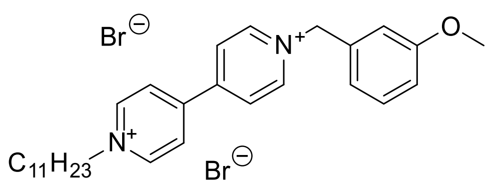

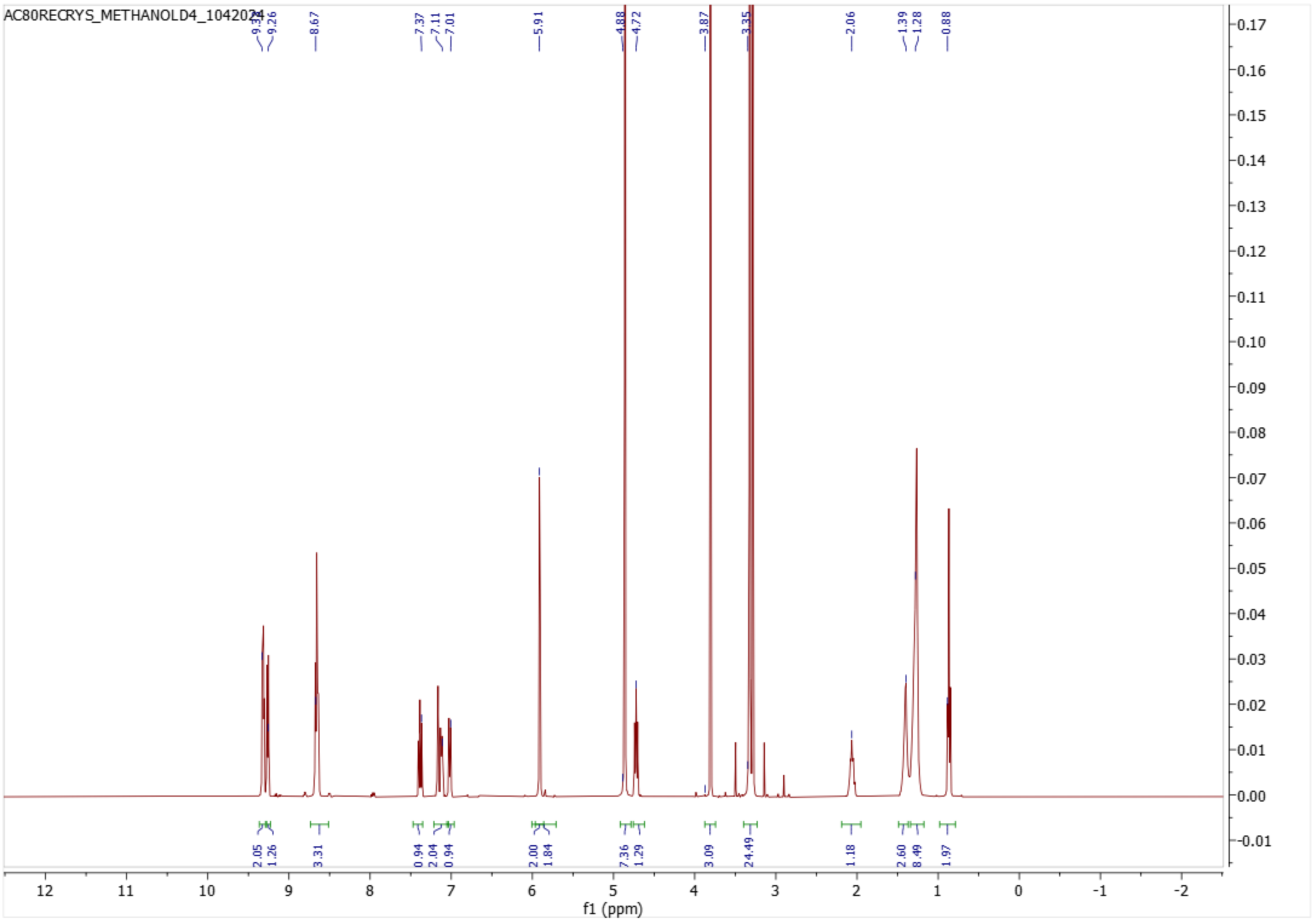

Figure S52: ^{1}H NMR (400 MHz) of **Paraquat-11,3-mOC1-Bn (AC80)** in CD_3OD.

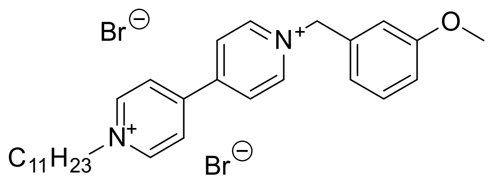

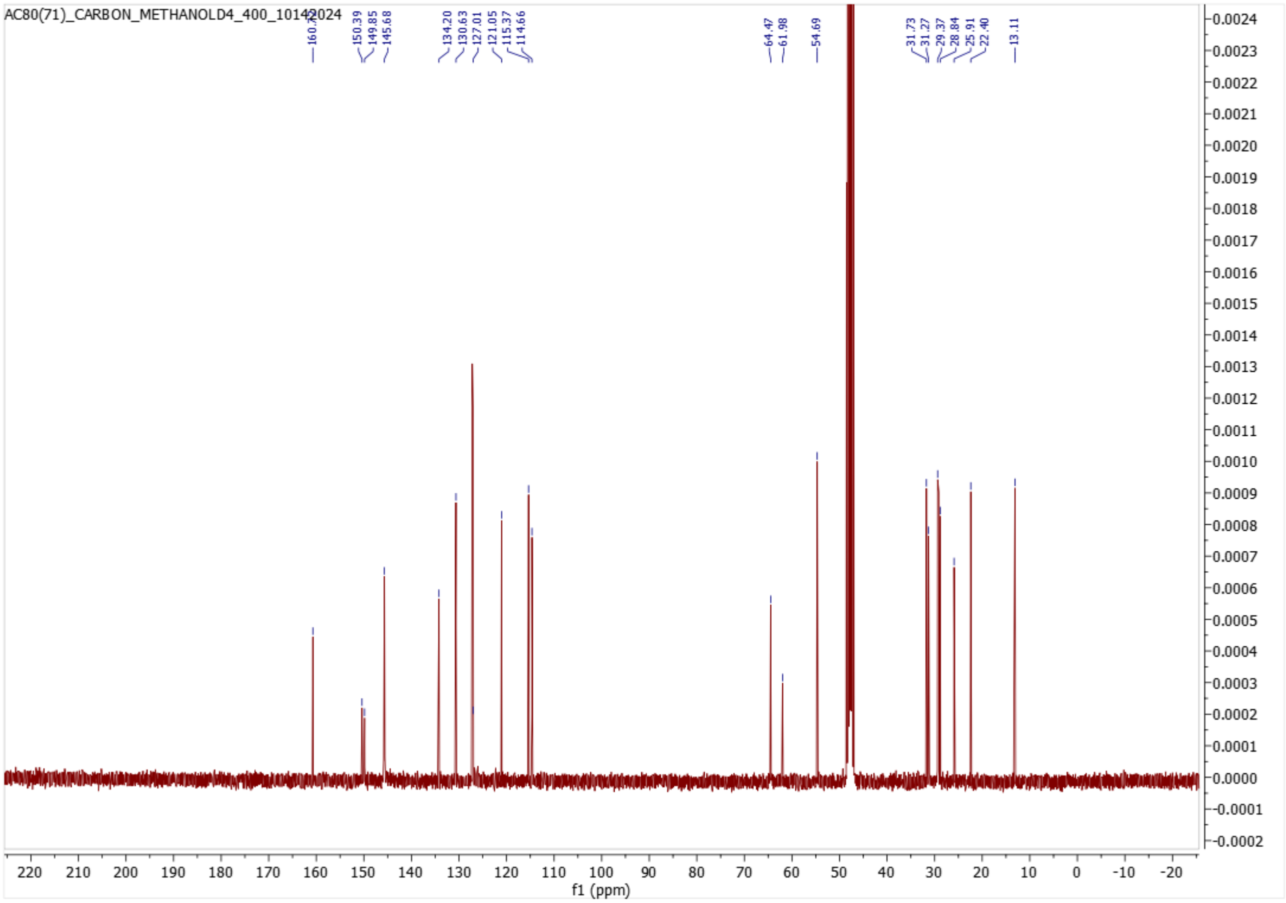

Figure S53: ^{13}C NMR (400 MHz) of **Paraquat-11,3-mOC1-Bn (AC80)** in CD_3OD.

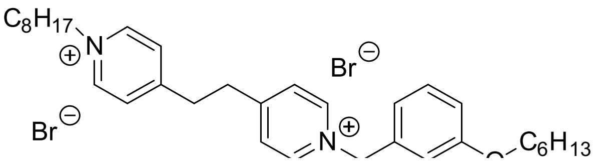

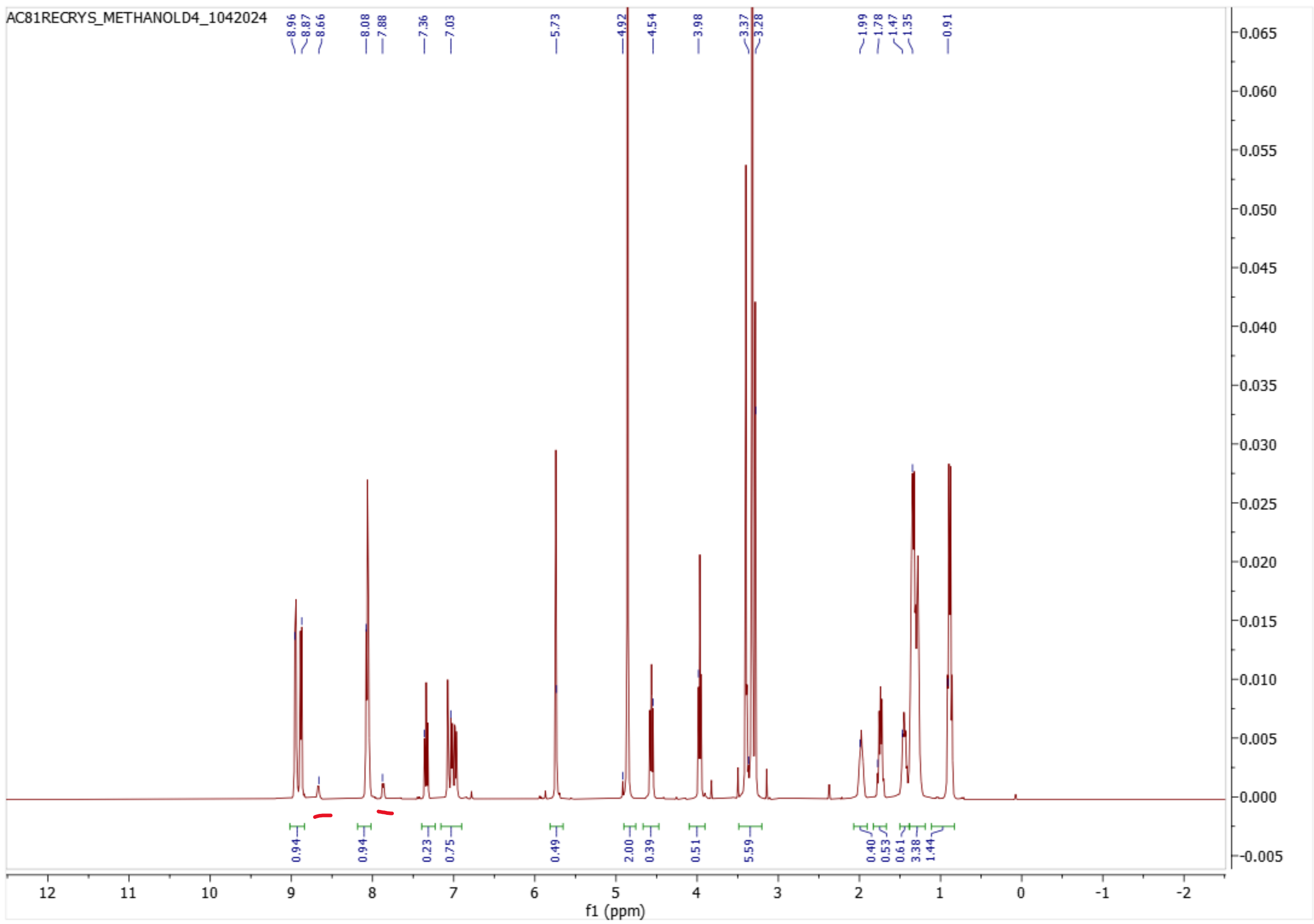

Figure S54: ^{1}H NMR (400 MHz) of **DPA-8,mOC6Bn (AC81)** in CD_3OD.

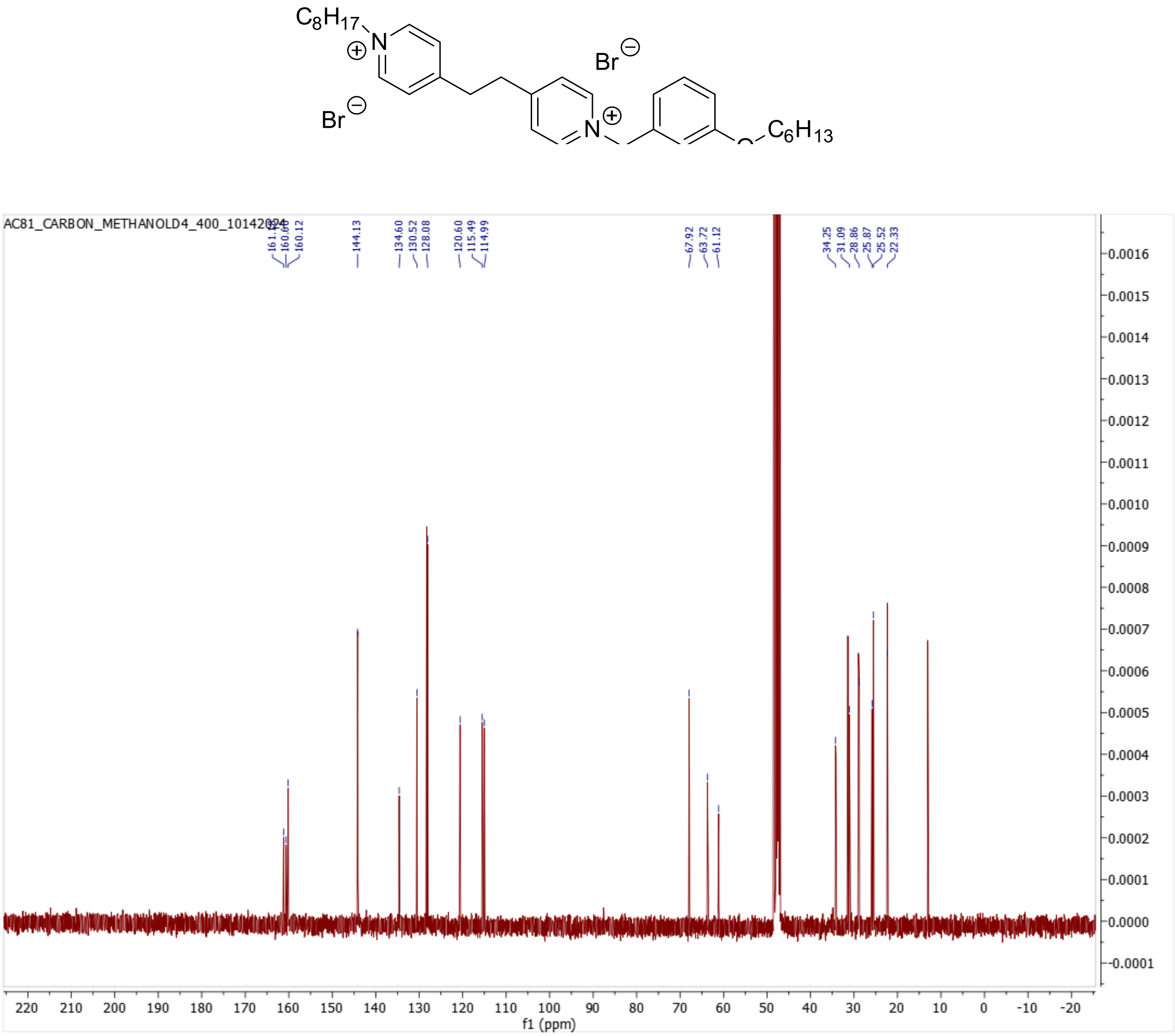

Figure S55: ^{13}C NMR (400 MHz) of **DPA-8,mOC6Bn (AC81)** in CD_3OD.

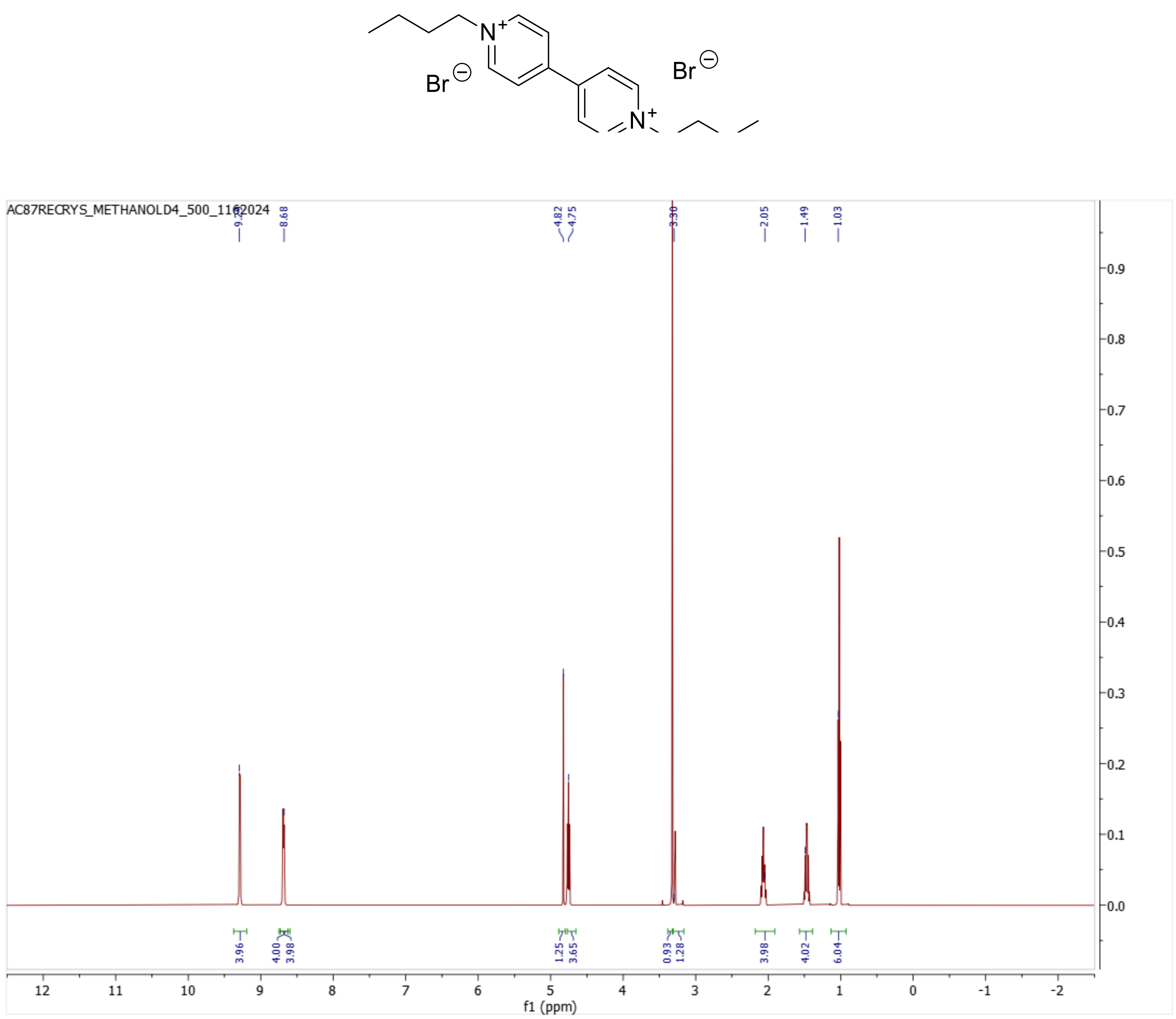

Figure S56: ^{1}H NMR (500 MHz) of **Paraquat-4,4 (AC87)** in CD_3OD.

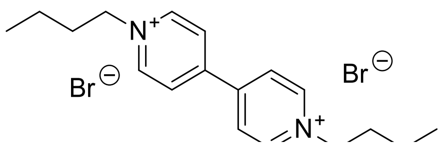

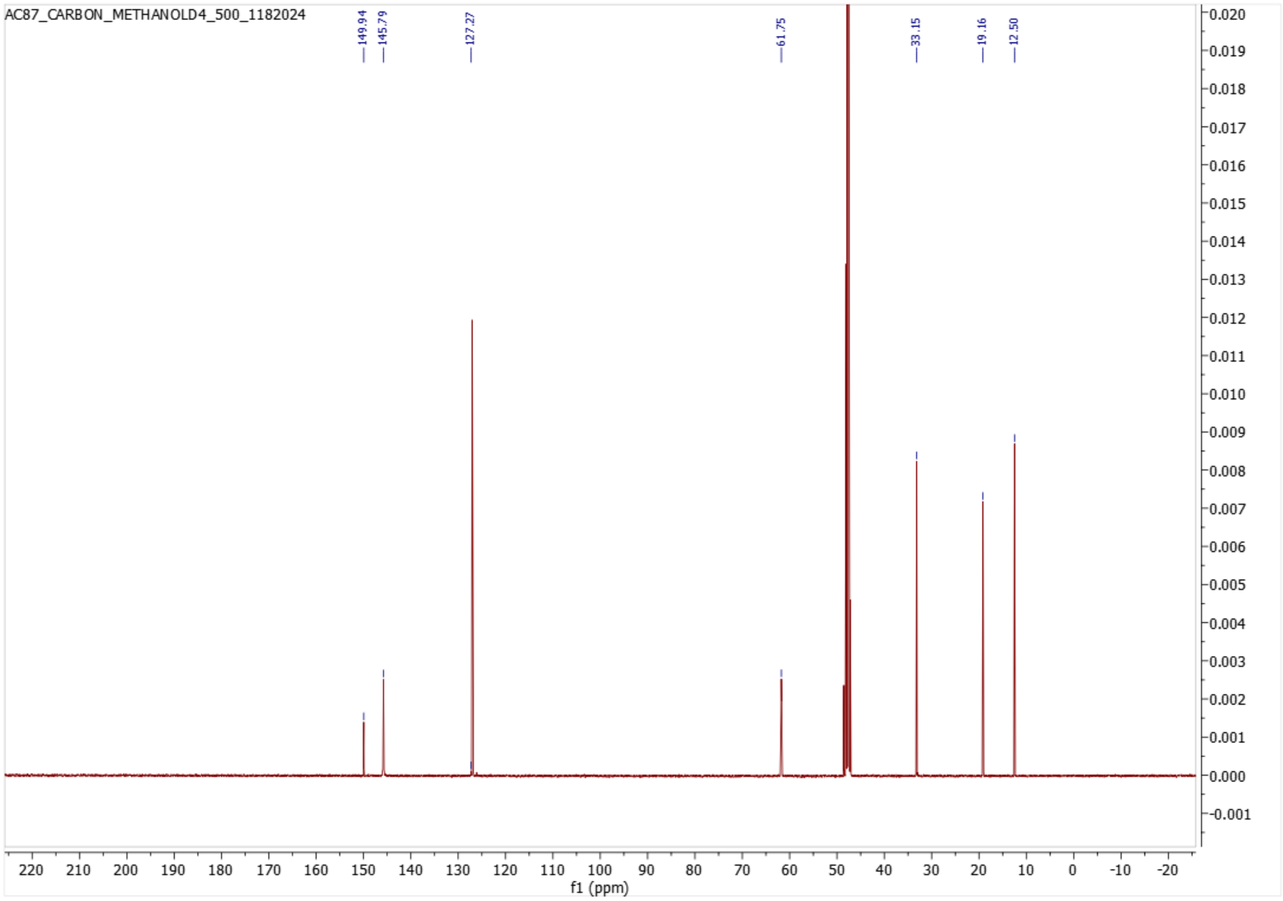

Figure S57: ^{13}C NMR (500 MHz) of **Paraquat-4,4 (AC87)** in CD_3OD.

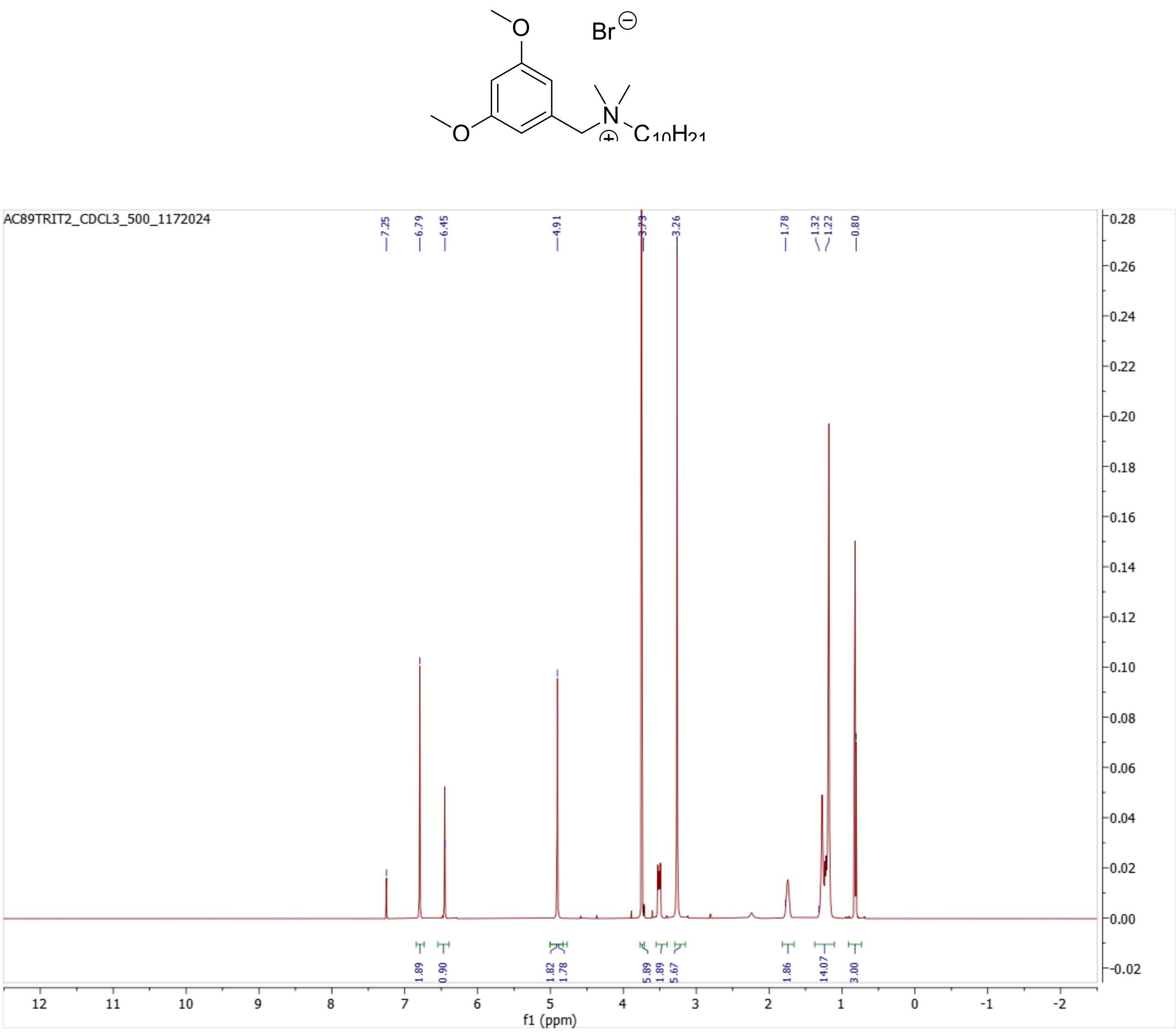

Figure S58: ^{1}H NMR (500 MHz) of **3,5methoxy-Bn-10 (AC89)** in $CDCl_3$.

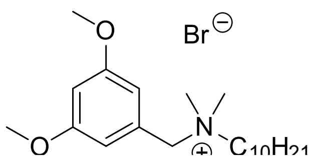

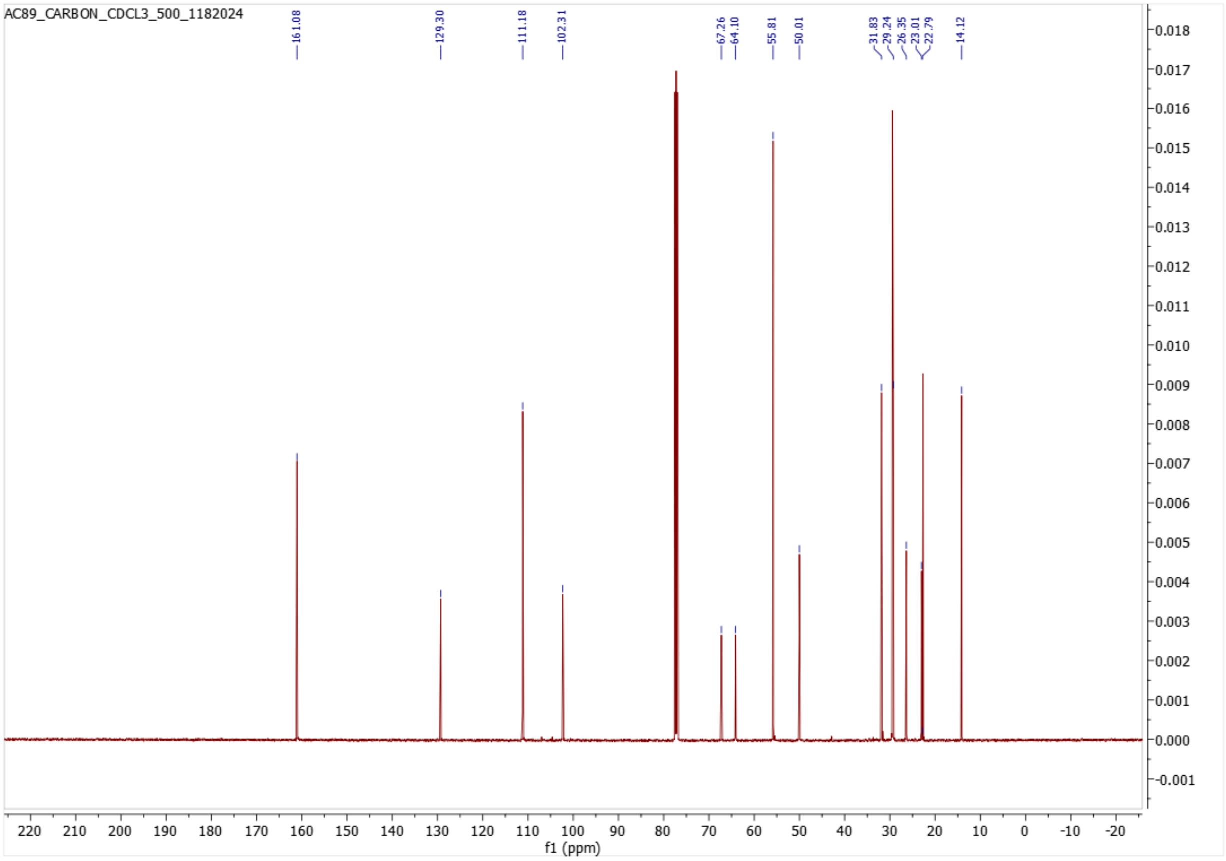

Figure S59: ^{13}C NMR (500 MHz) of **3,5methoxy-Bn-10 (AC89)** in $CDCl_3$.

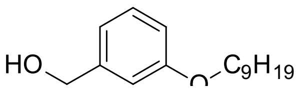

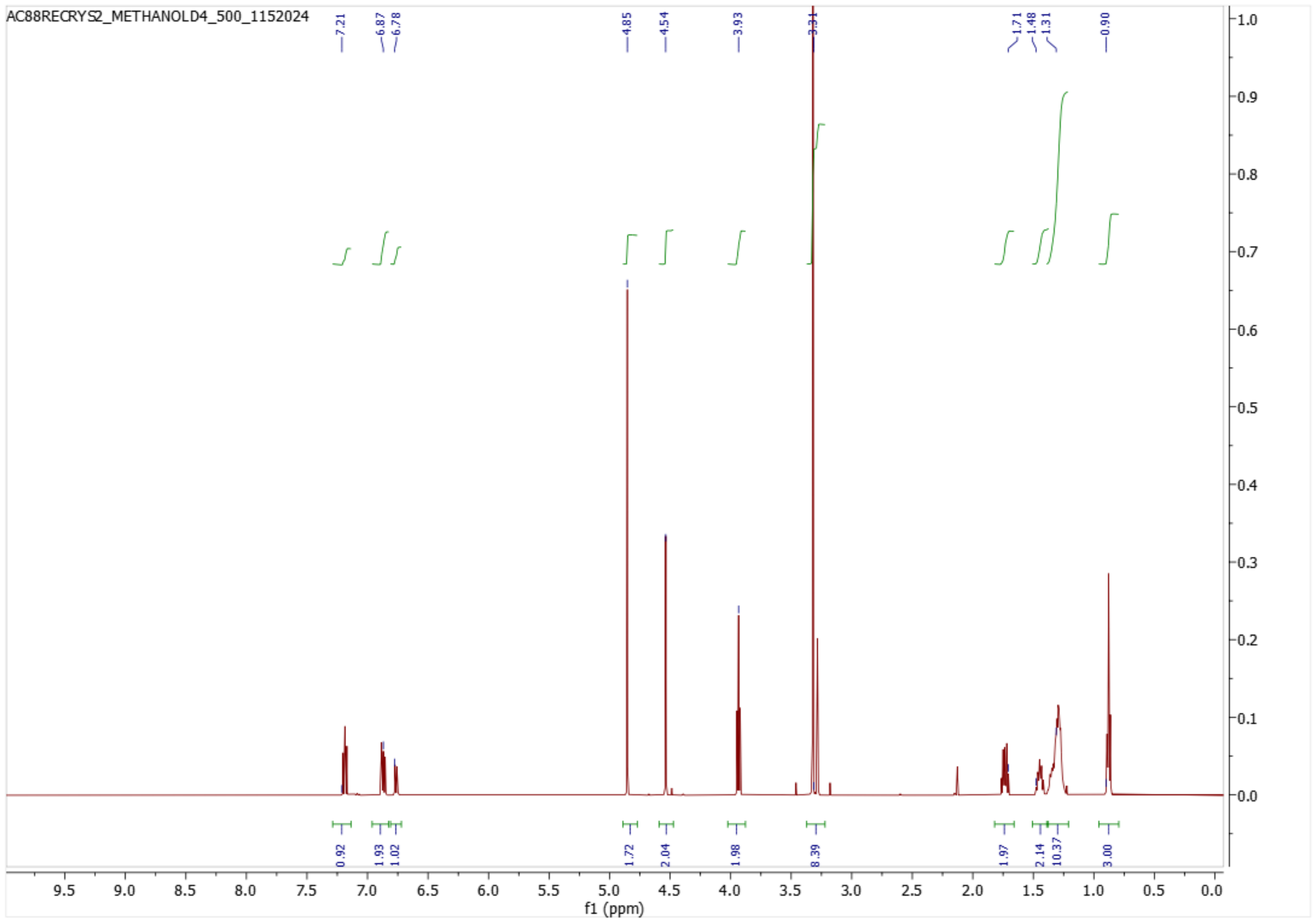

Figure S60: ^{1}H NMR (500 MHz) of **AC88** in CD_3OD.

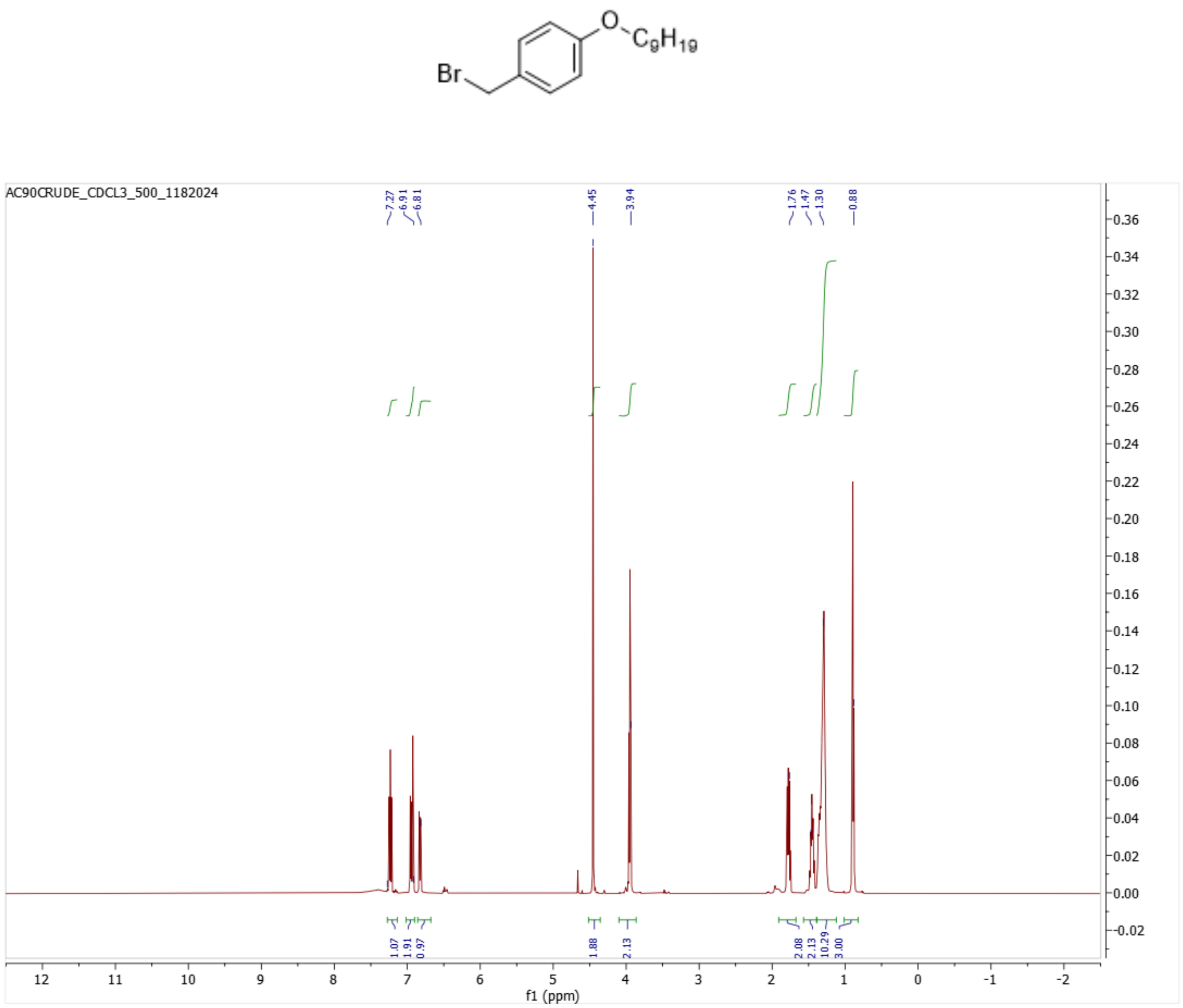

Figure S61: ^{1}H NMR (500 MHz) of **AC90** in $CDCl_3$.

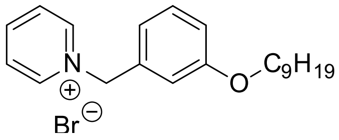

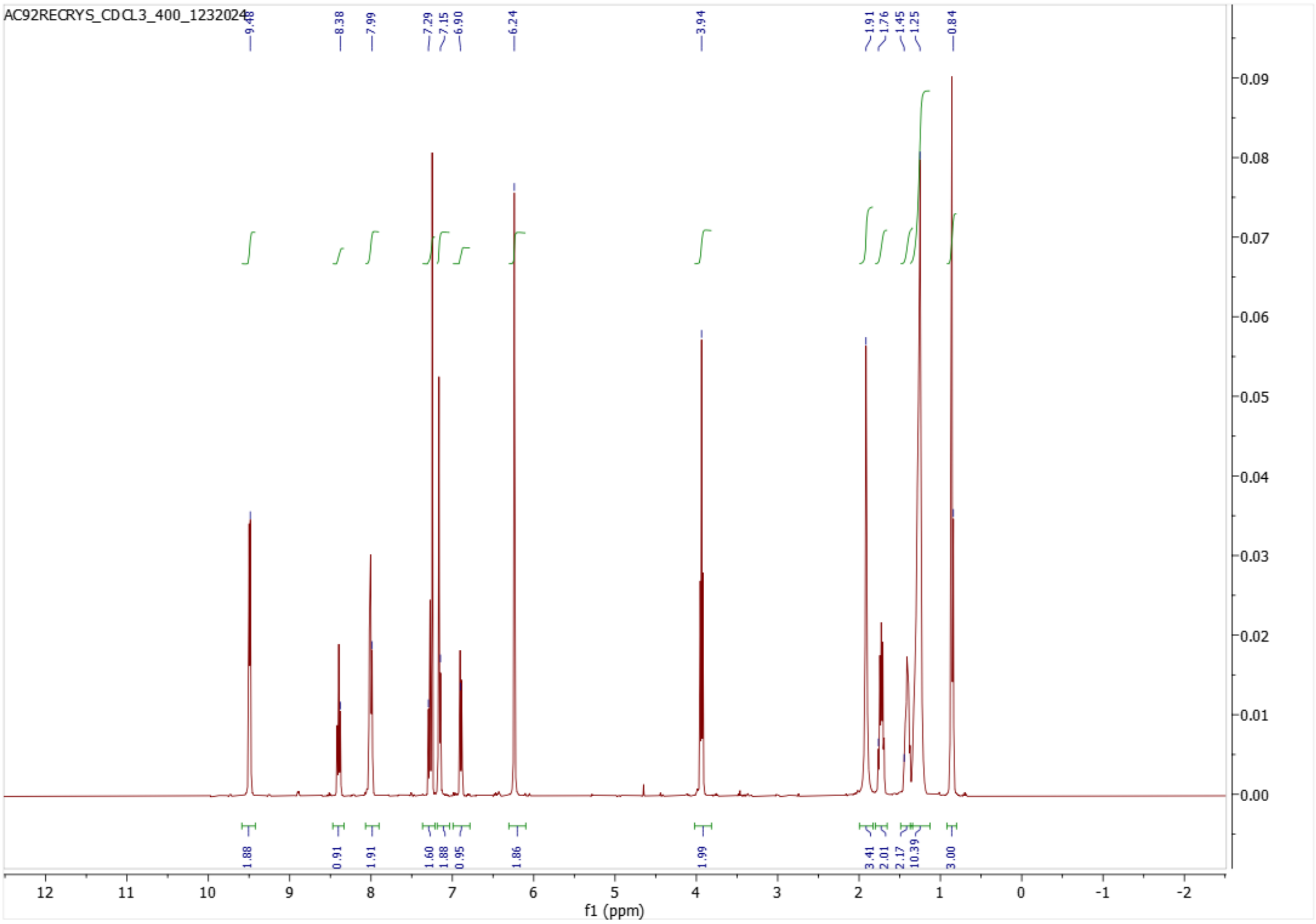

Figure S62: ^{1}H NMR (400 MHz) of **Pyr-mOC9 (AC92)** in $CDCl_3$.

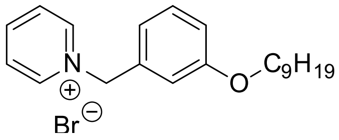

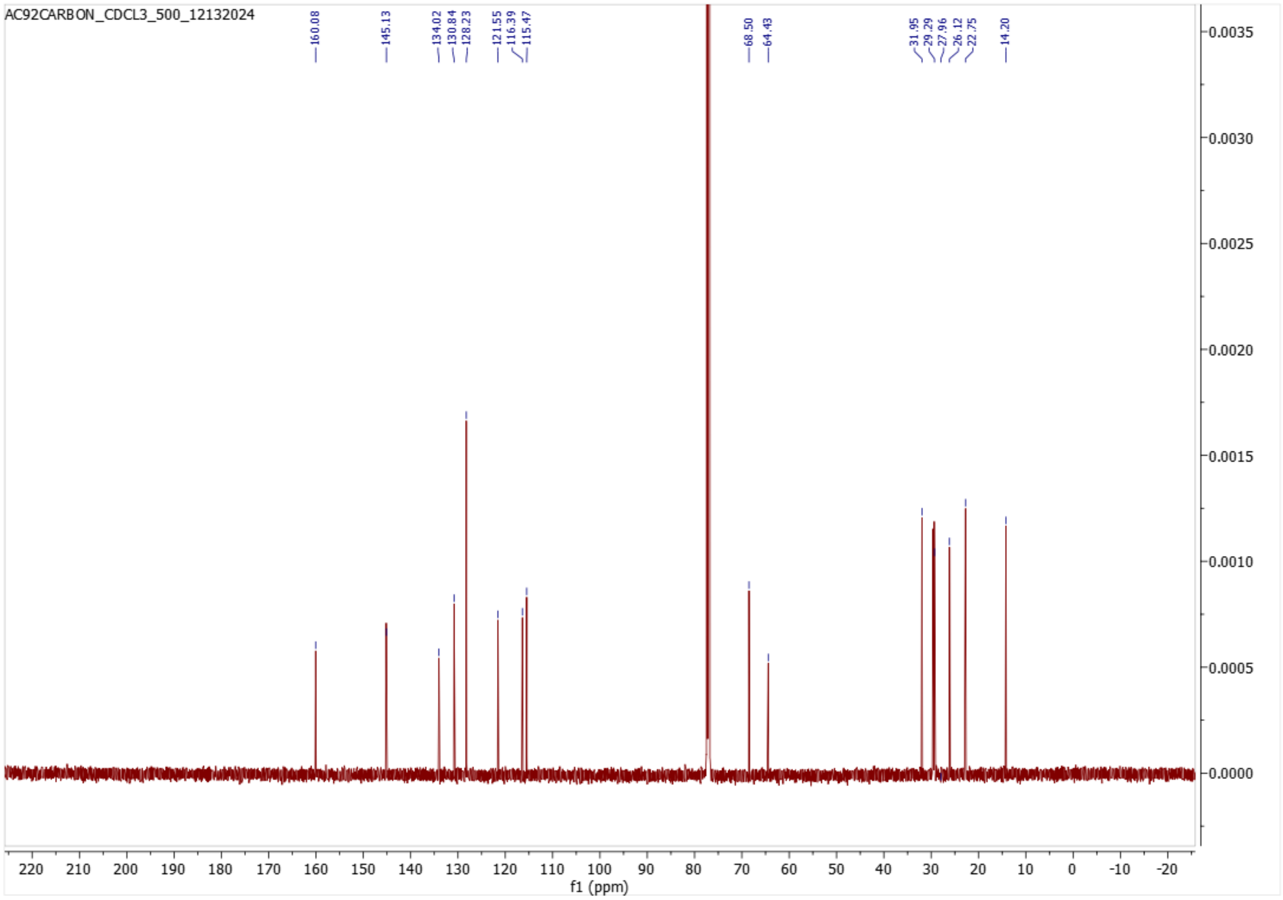

Figure S63:^{13}C NMR (500 MHz) of **Pyr-mOC9 (AC92)** in $CDCl_3$.

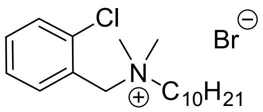

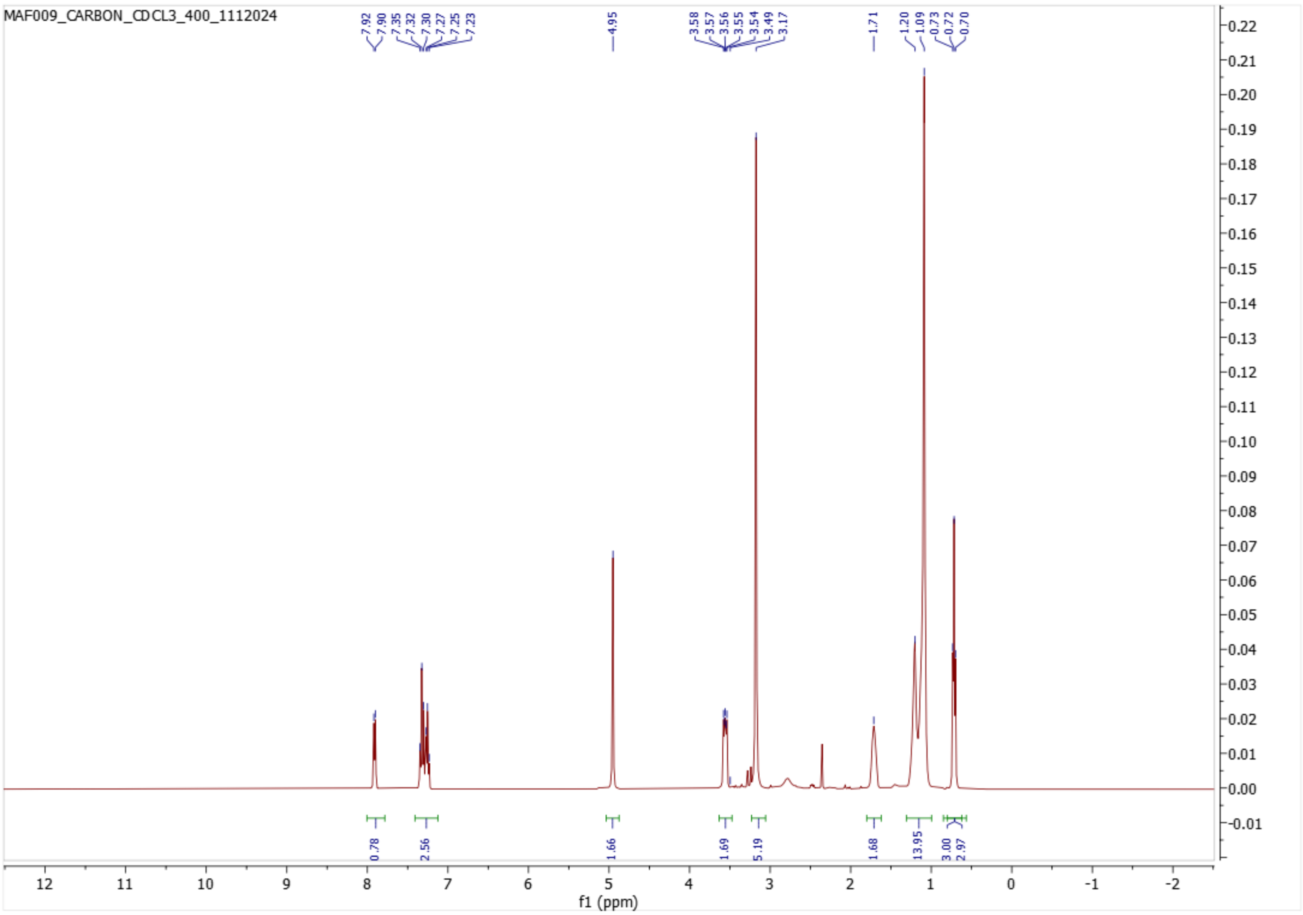

Figure S64:^{1}H NMR (400 MHz) of **oCl-Bn-10 (MAF-009)** in $CDCl_3$.

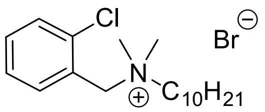

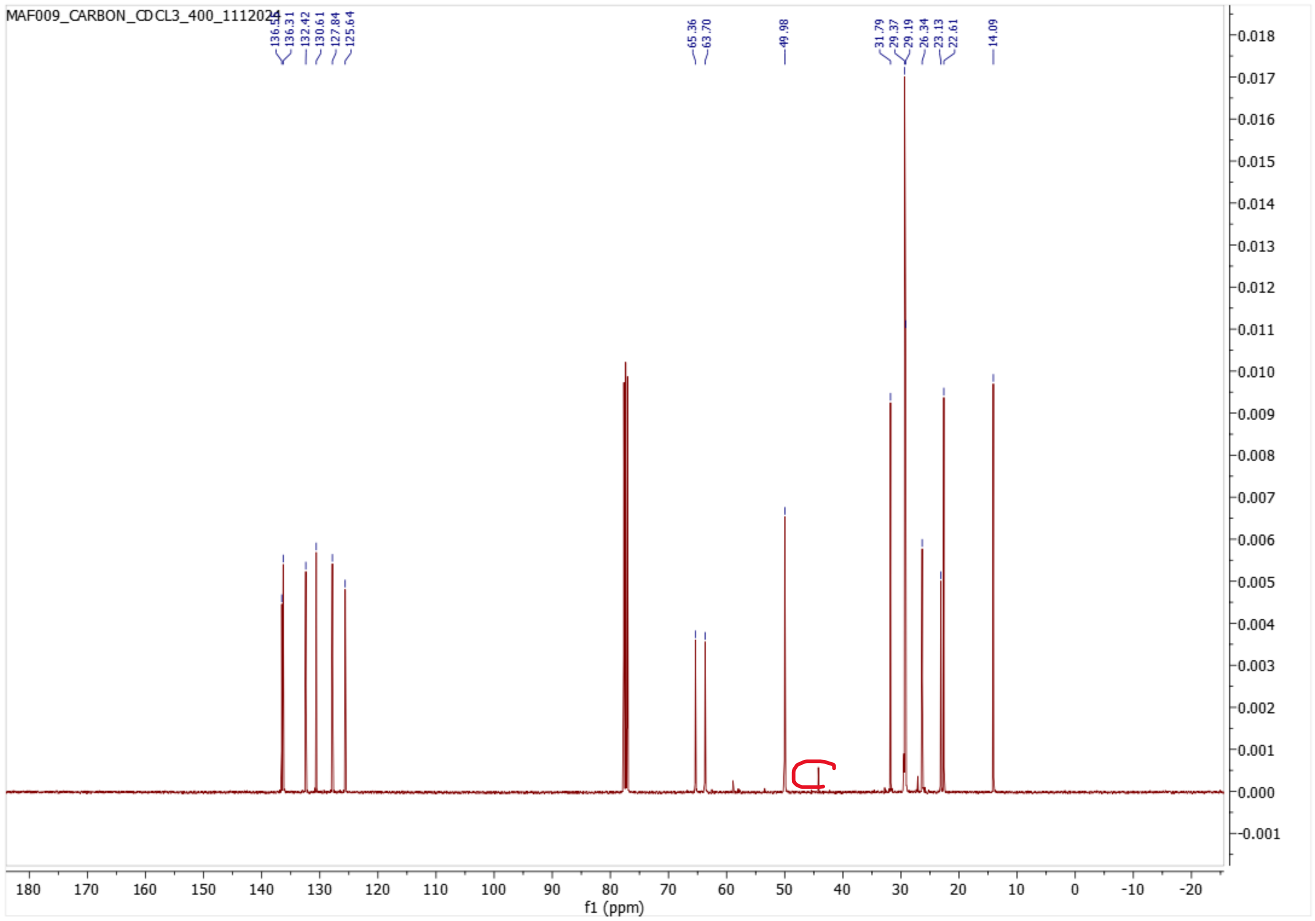

Figure S65: ^{13}C NMR (400 MHz) of **oCl-Bn-10 (MAF-009)** in $CDCl_3$.

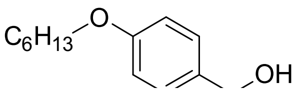

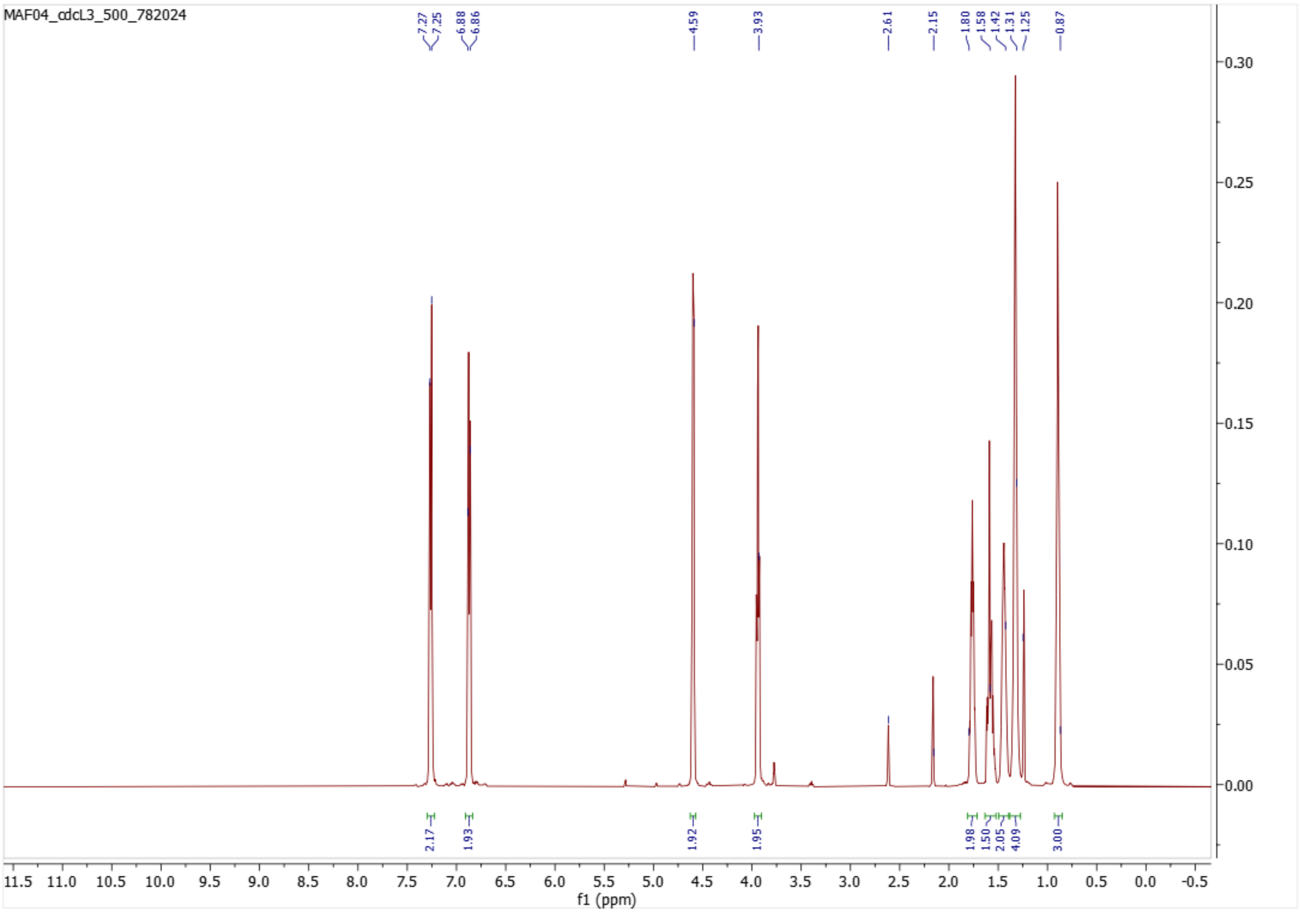

Figure S66: ^{1}H NMR (500 MHz) of **MAF-004** in $CDCl_3$.

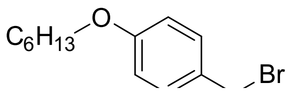

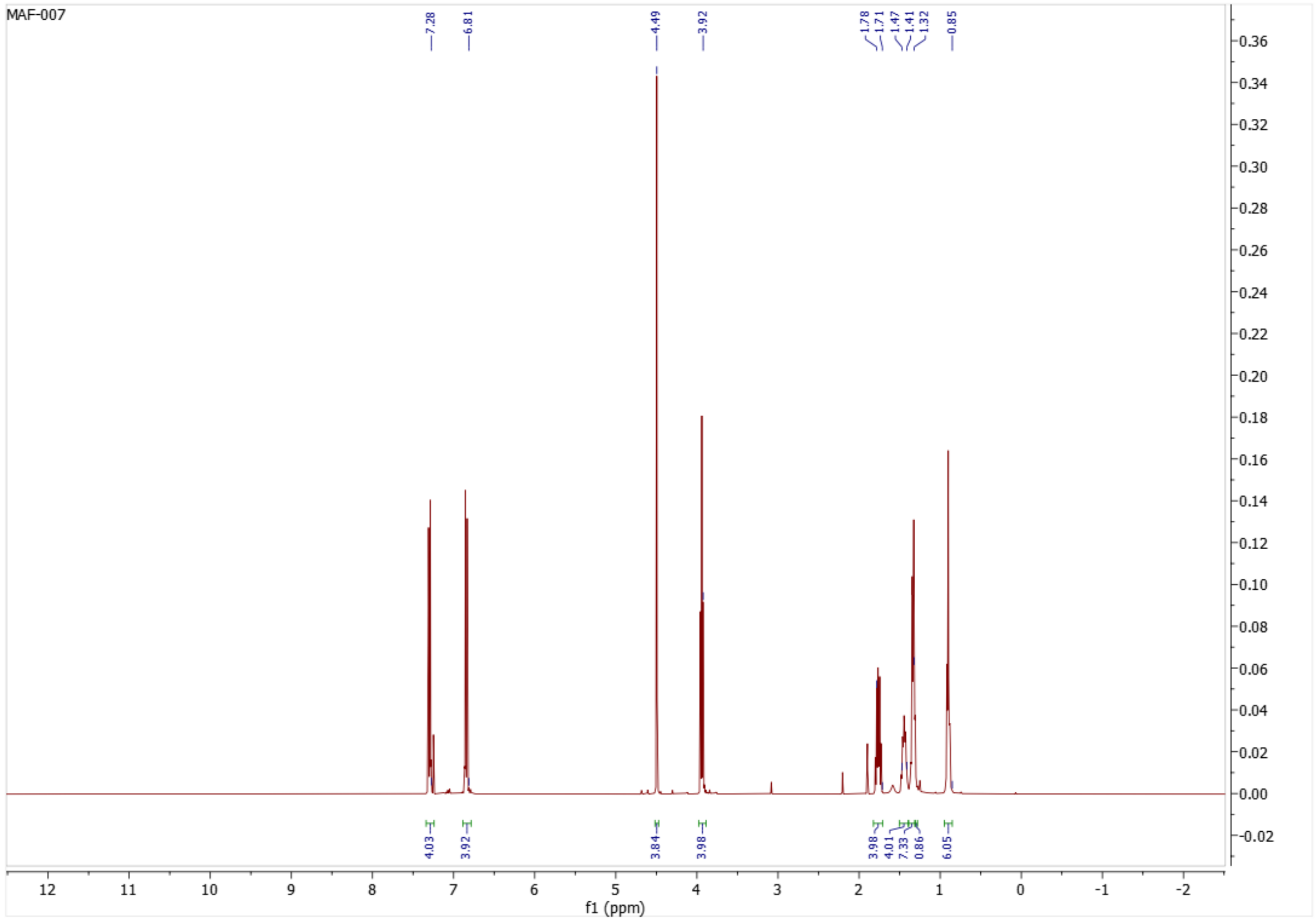

Figure S67: ^{1}H NMR (500 MHz) of **MAF-007** in $CDCl_3$.

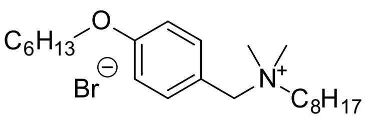

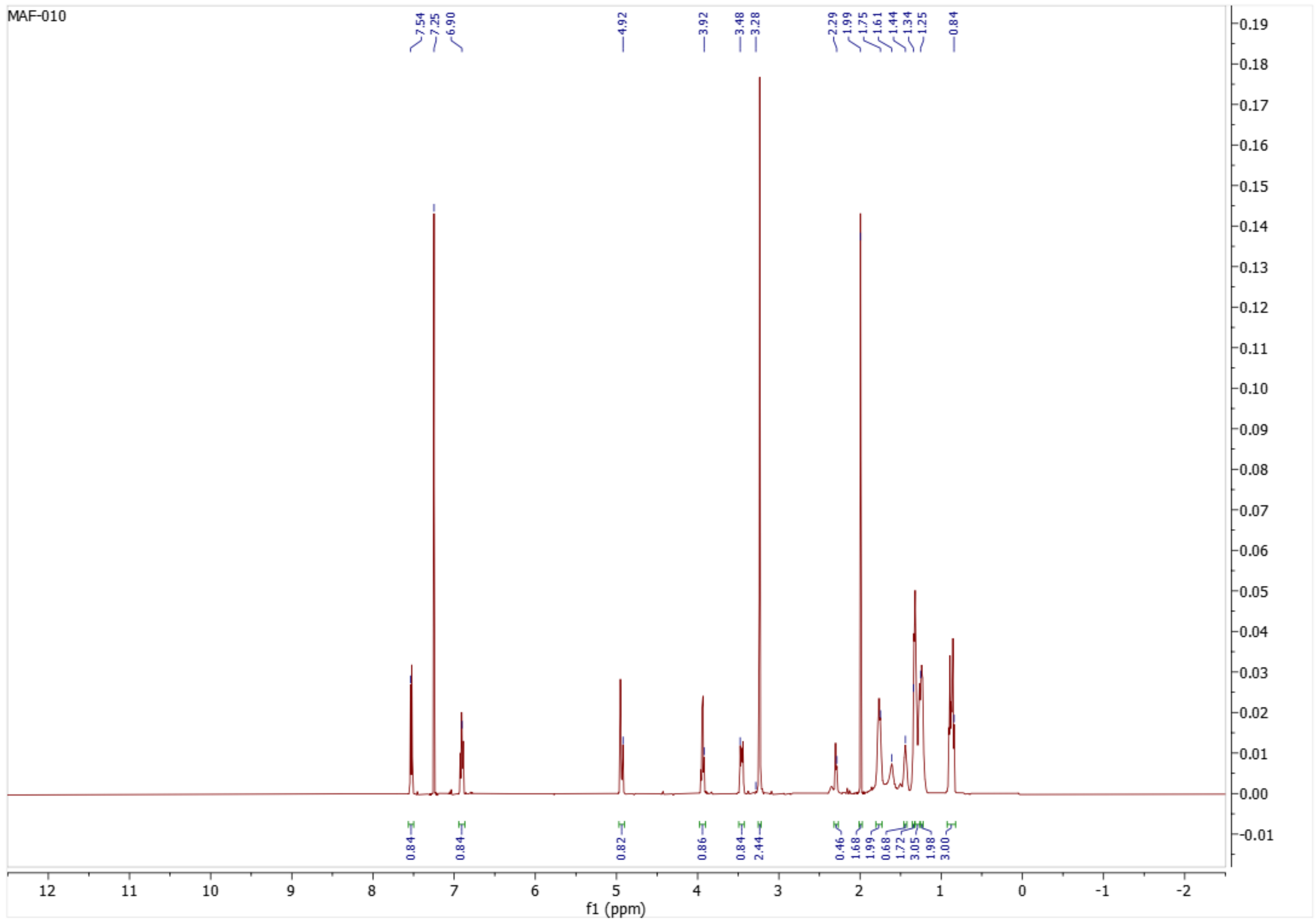

Figure S68: ^{1}H NMR (500 MHz) of **pOC6-Bn-8 (MAF-010)** in $CDCl_3$.

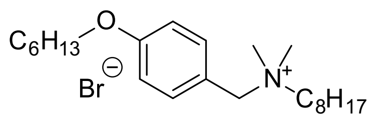

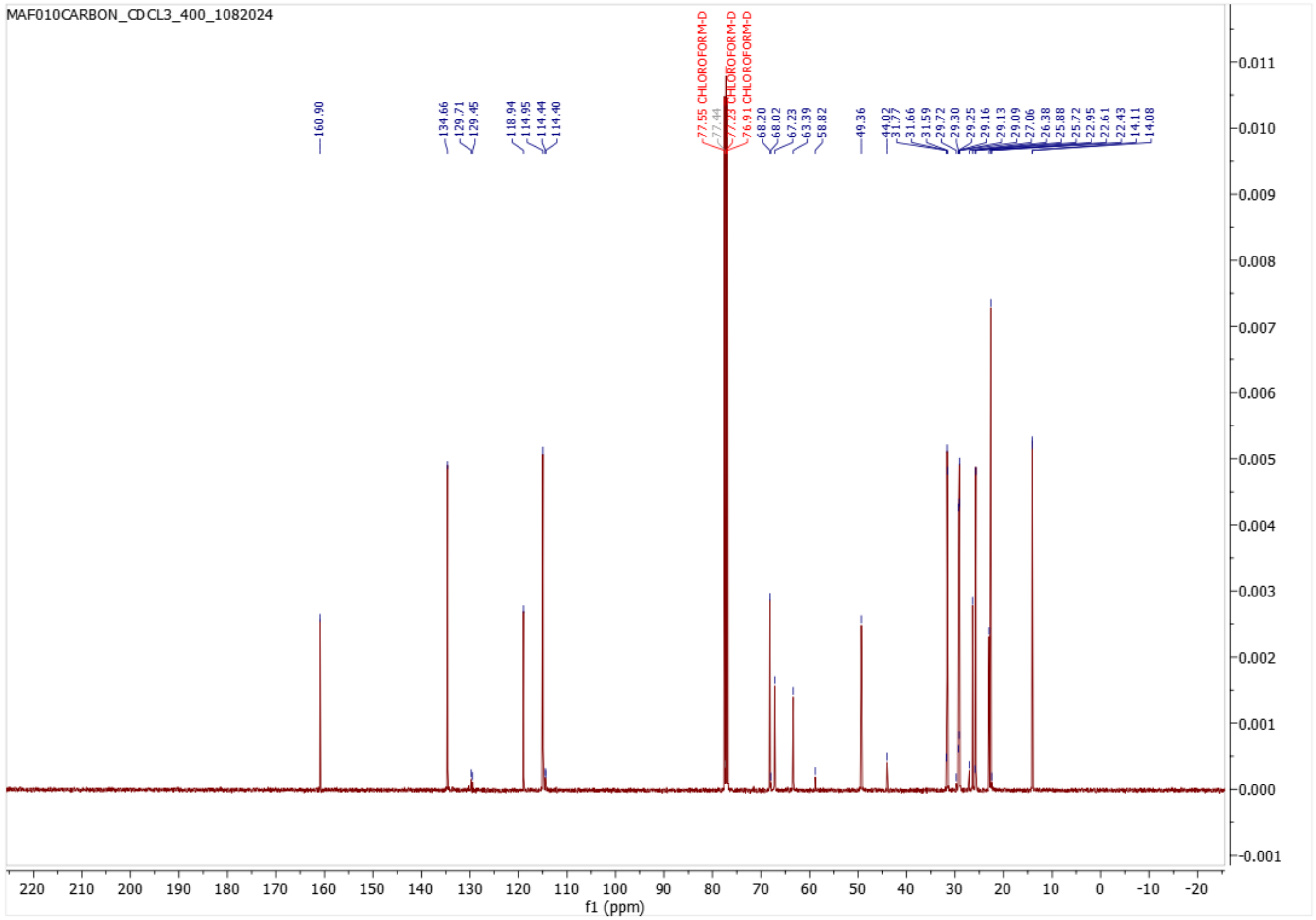

Figure S69: ^{13}C NMR (500 MHz) of **pOC6-Bn-8 (MAF-010)** in $CDCl_3$.

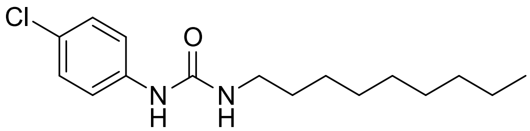

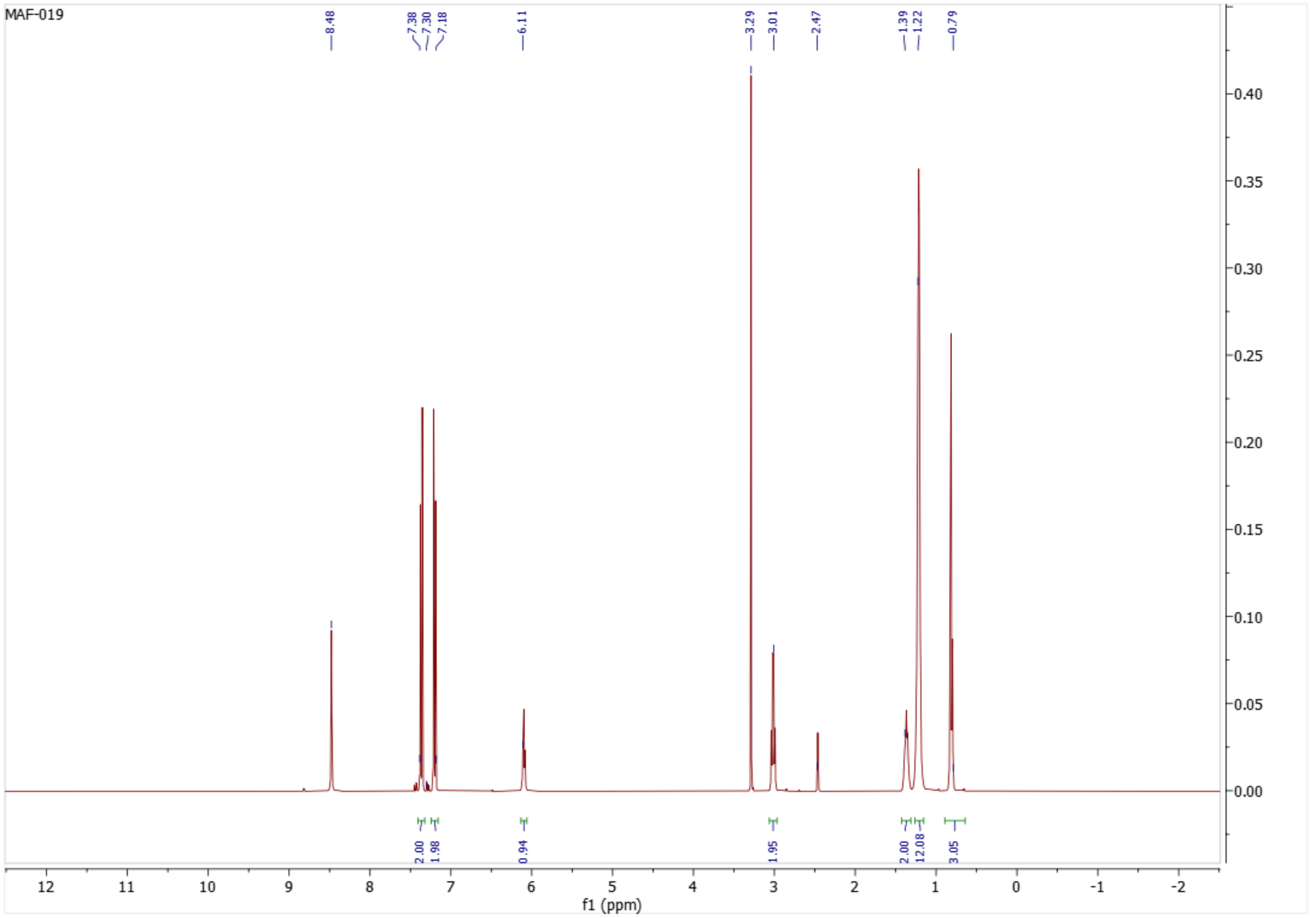

Figure S70: ^{1}H NMR (400 MHz) of **Urea-9,pClPh (MAF-019)** in DMSO-D_6.

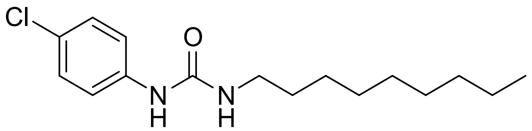

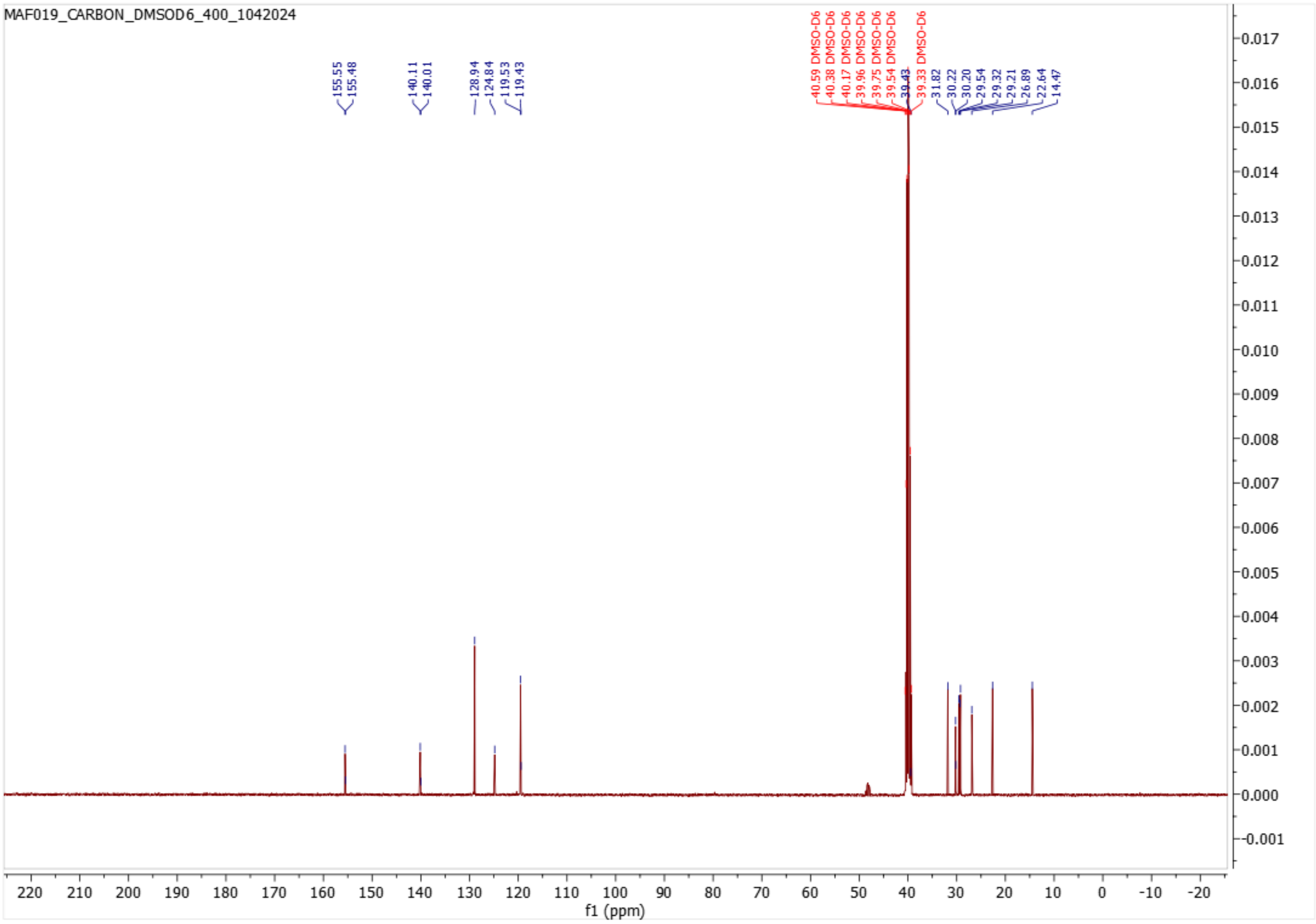

Figure S71: ^{13}C NMR (400 MHz) of **Urea-9,pClPh (MAF-019)** in DMSO-D$_6$.

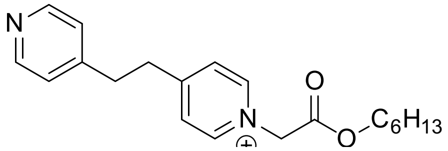

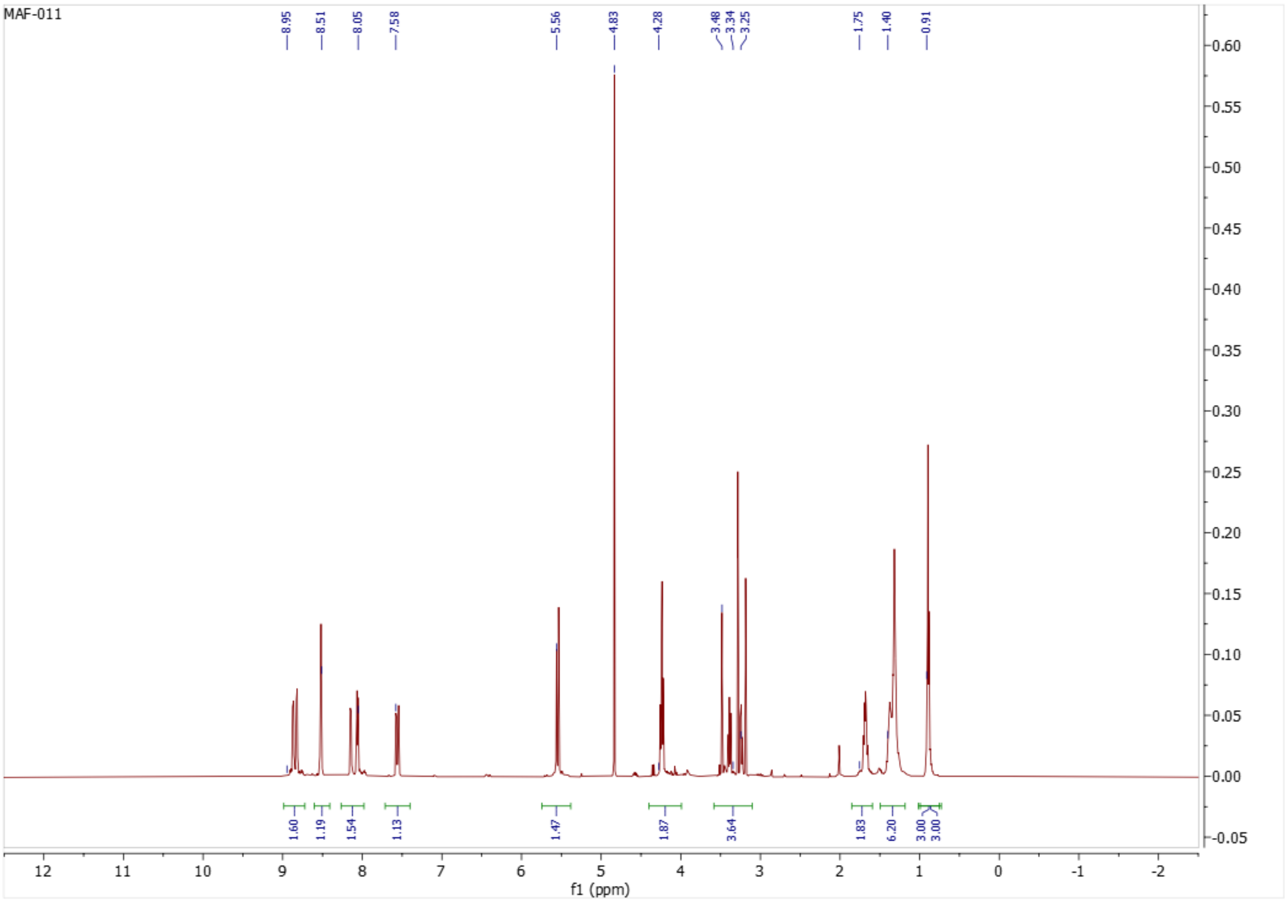

Figure S72: ^{1}H NMR (500 MHz) of **MAF-011** in CD_3OD.

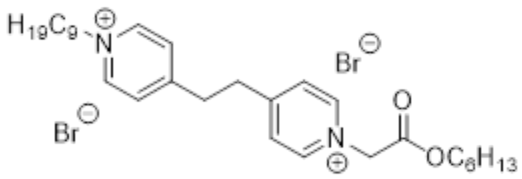

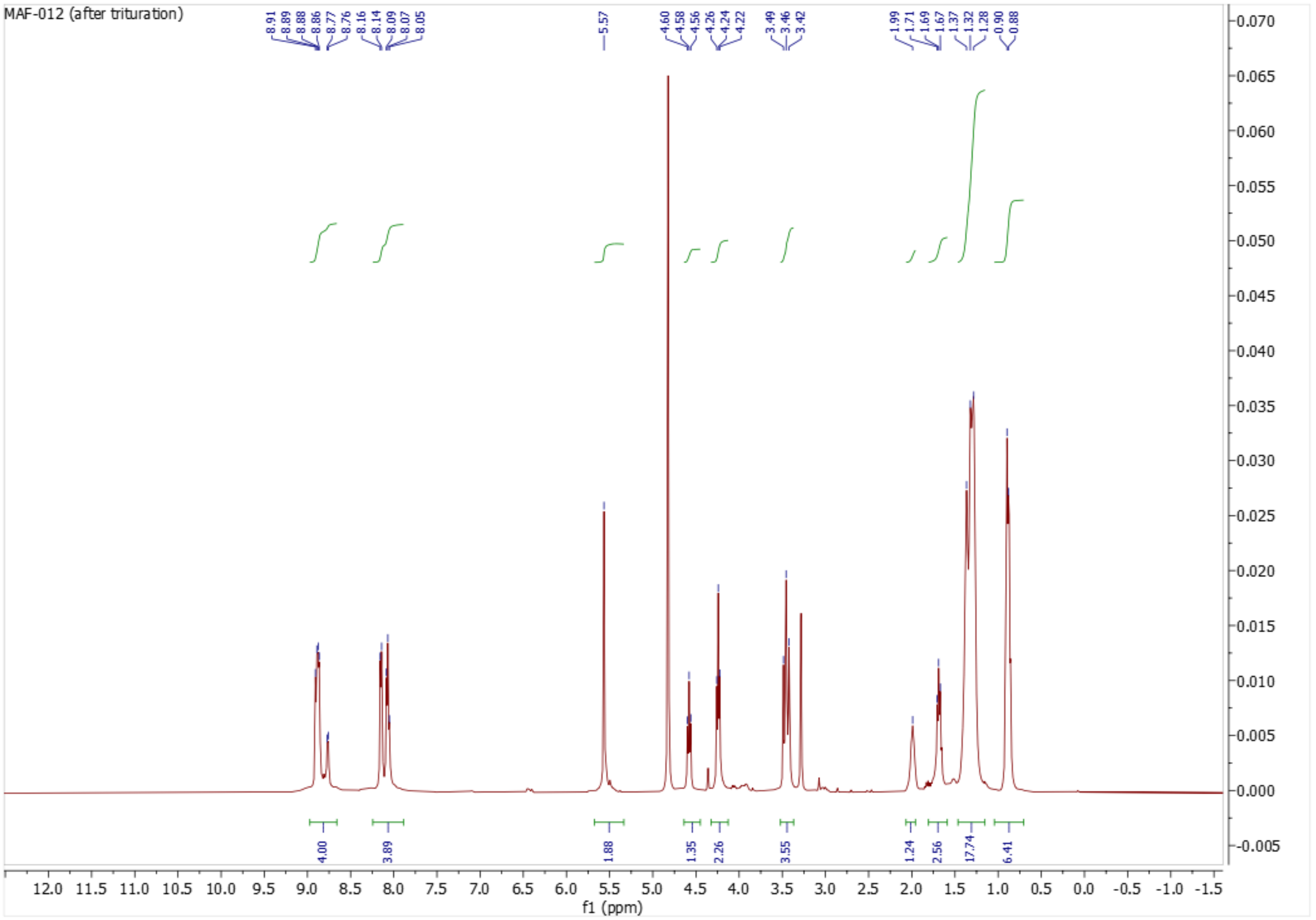

Figure S73: ^{1}H NMR (500 MHz) of **DPA-9E,9E (MAF-012)** in CD_3OD.

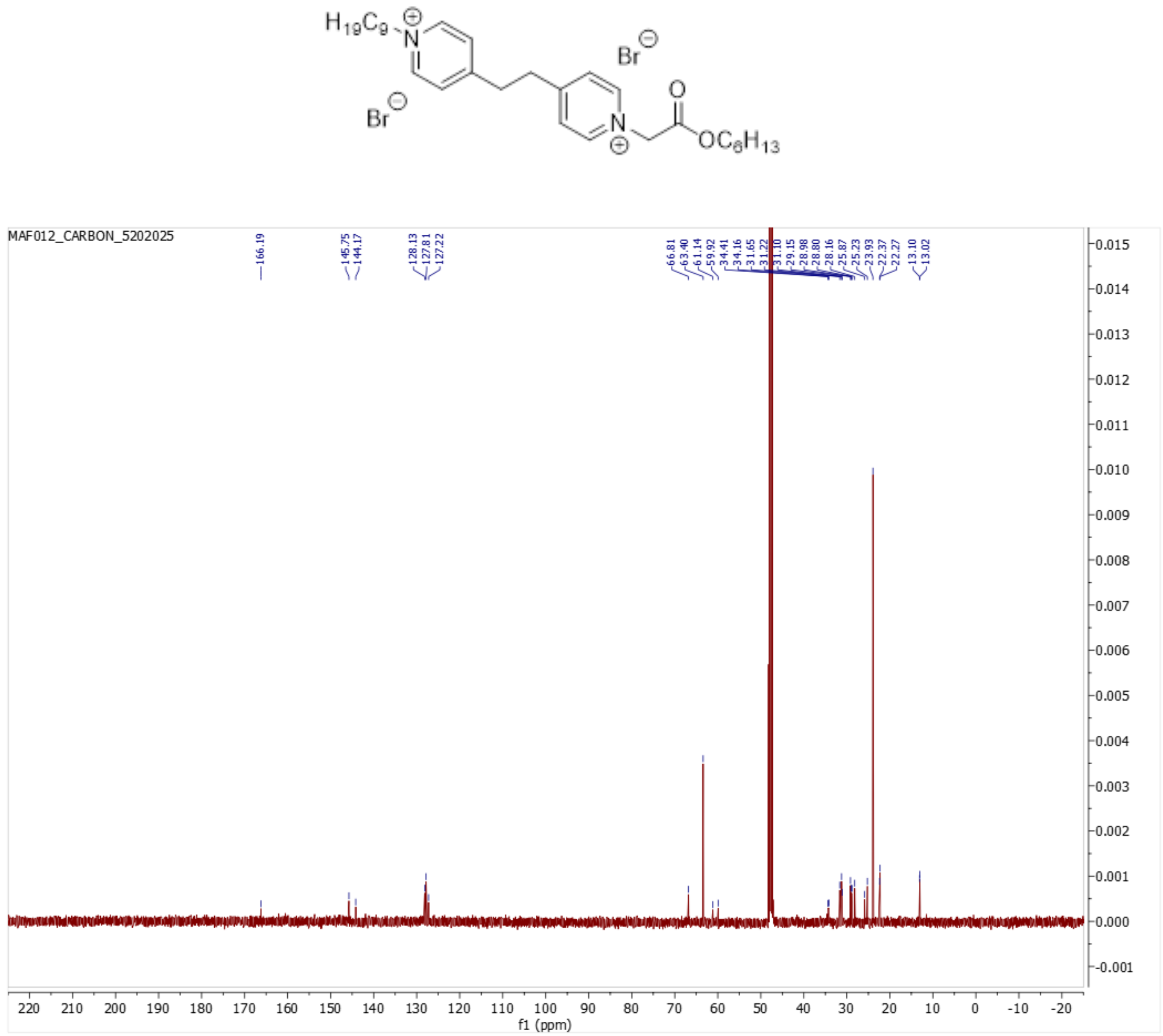

Figure S74: ^{13}C NMR (500 MHz) of **DPA-9E,9E (MAF-012)** in CD_3OD.

VII. References

(1) Paniak, T. J.; Jennings, M. C.; Shanahan, P. C.; Joyce, M. D.; Santiago, C. N.; Wuest, W. M.; Minbiole, K. P. C. The Antimicrobial Activity of Mono-, Bis-, Tris-, and Tetracationic Amphiphiles Derived from Simple Polyamine Platforms. *Bioorg Med Chem Lett* **2014**, *24* (24). https://doi.org/10.1016/j.bmcl.2014.10.018.

(2) Gan, L. H.; Deen, G. R.; Gan, Y. Y.; Chew, C. H. Synthesis and Properties of Piperazine Derivatives and Their Quaternary Ammonium Amphiphilic Salts. *J Colloid Interface Sci* **1996**, *183* (2). https://doi.org/10.1006/jcis.1996.0554.

(3) Hoque, J.; Akkapeddi, P.; Yarlagadda, V.; Uppu, D. S. S. M.; Kumar, P.; Haldar, J. Cleavable Cationic Antibacterial Amphiphiles: Synthesis, Mechanism of Action, and Cytotoxicities. *Langmuir* **2012**, *28* (33). https://doi.org/10.1021/la302303d.

(4) Eastoe, J.; Sharpe, D.; Heenan, R. K.; Egelhaaf, S. Rigidities of Cationic Surfactant Films in Microemulsions. *Journal of Physical Chemistry B* **1997**, *101* (6). https://doi.org/10.1021/jp962048y.

(5) Arnold, D. P.; Bartley, J. P.; James, D. A. Self-Aggregation of a Synthetic Amphiphilic Porphyrin. *Aust J Chem* **1992**, *45* (8). https://doi.org/10.1071/CH9921191.

(6) Hamed, F. H. Dephosphorylation and Aromatic Substitution in Functional Surfactants, Micelles in Ethylene Glycol-Water, and Microemulsions Containing Functional Surfactant, University of California, Santa Barbara, CA, 1981.

(7) Percec, V.; Heck, J. Liquid Crystalline Polymers Containing Mesogenic Units Based on Half-disc and Rod-like Moieties. I. Synthesis and Characterization of 4-(11-undecan-1-yloxy)-4′-[3,4,5-tri(P-n-dodecan-1-yloxybenzyloxy)Benzoate]Biphenyl Side Groups. *J Polym Sci A Polym Chem* **1991**, *29* (4). https://doi.org/10.1002/pola.1991.080290416.

(8) Nozary, H.; Piguet, C.; Tissot, P.; Bernardinelli, G.; Bünzli, J. C. G.; Deschenaux, R.; Guillon, D. Bent Tridentate Receptors in Calamitic Mesophases with Predetermined Photophysical Properties: New Luminescent Lanthanide-Containing Materials. *J Am Chem Soc* **1998**, *120* (47). https://doi.org/10.1021/ja982545n.

(9) Wang, Y.; Zhou, Q.; Guo, S.; Yang, K.; Mi, B.; Zhang, X.; Li, H.; Liu, X.; Han, M.; Deng, J. (P-Tetradecyloxybenzyl)Betaine Surfactant, Its Preparation Method from p-Hydroxybenzyl Alcohol and Application in Coal Seam Clean Fracturing Fluid. CN115286526, November 4, 2022.

(10) Li, B.; Wei, P.; de Leon, A.; Frey, T.; Pentzer, E. Polymer Composites with Photo-Responsive Phthalocyanine for Patterning in Color and Fluorescence. *Eur Polym J* **2017**, *89*. https://doi.org/10.1016/j.eurpolymj.2017.02.042.

(11) Wisastra, R.; Kok, P. A. M.; Eleftheriadis, N.; Baumgartner, M. P.; Camacho, C. J.; Haisma, H. J.; Dekker, F. J. Discovery of a Novel Activator of 5-Lipoxygenase from an Anacardic Acid Derived Compound Collection. *Bioorg Med Chem* **2013**, *21* (24). https://doi.org/10.1016/j.bmc.2013.10.015.

(12) Gathergood, N.; Garcia, M. T.; Scammells, P. J. Biodegradable Ionic Liquids: Part I. Concept, Preliminary Targets and Evaluation. *Green Chemistry* **2004**, *6* (3). https://doi.org/10.1039/b315270g.
